# Synthesis of Farnesyloxy- and Drimanyloxy-Arene Scaffold-Based Hybrid Molecules as Antifungals against *Botrytis cinerea*

**DOI:** 10.1021/acs.jafc.5c06584

**Published:** 2025-10-27

**Authors:** Antonio Ruano-González, Ana A. Pinto, Gabriela Mancilla, Rosario Sánchez-Maestre, Josefina Aleu, Rosario Hernández-Galán, Antonio J. Macías-Sánchez, Isidro G. Collado

**Affiliations:** † Departamento de Química Orgánica, Facultad de Ciencias, 16727 Universidad de Cádiz, Puerto Real, 11510 Cádiz, Spain; ‡ Instituto de Investigación en Biomoléculas (INBIO), Universidad de Cádiz, Puerto Real, 11510 Cádiz, Spain

**Keywords:** *Botrytis cinerea*, phytopathogenic fungi, antifungal, hybrid molecules, farnesyloxyarenes, drimanyloxyarenes

## Abstract

There is an increasing interest in the use of biopesticides, which implies the use of living microorganisms, plant and microbial extracts, and natural products isolated from the above-mentioned sources or closely related derivatives. In this regard, 10 farnesyloxy-arenes derivatives (**21**–**25**, (±)-**26**–(±)-**30**) and 12 drimanyloxyarenes derivatives ((±)-**12**–(±)-**14**, (±)-**31**–(±)-**40**) were synthesized and tested against the phytopathogenic fungus *Botrytis cinerea*. In general, the drimanyloxyarenes derivatives were more fungitoxic than their farnesyloxyarene precursors. The most active compounds were (±)-**12**, (±)-**31**, and (±)-**35**, which at a 391 × 10^–5^ mg/mL dose, were more active than azoxystrobin, while (±)-**12** presented an inhibition comparable to that of triclosan. Structure–activity relationships are discussed, as well as the correlation with calculated physicochemical properties such as logP and total polar surface area.

## Graphical abstract

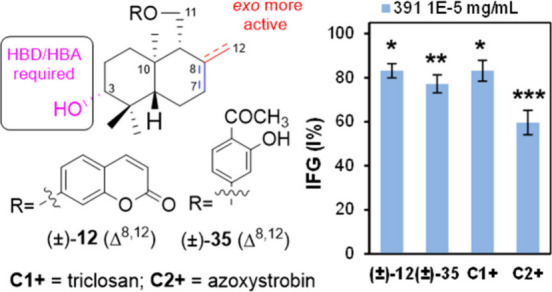

## 1 Introduction

*Botrytis* species are the causal agent of gray mold disease and cause important economic losses in a high number of commercial crops at all stages of their growth, including agricultural storage and transport.^1,2^ While most species are host specific, *B. cinerea* has a wide host range which involves 596 vascular plant genera,^2^ including wild plants as well as ornamental, greenhouse and field crops, such as tomatoes, vines, strawberries, tulips, and onions, among others.^3,4^

Infection is promoted by high humidity conditions, involving a variety of pathways to achieve infection and colonization.^5^ Chemical control based on the application of mostly synthetic fungicides constitutes the prevalent and reliable crop protection option for many crops.^6^ Nevertheless, actual concerns about the increasing impact on the environment,^7^ human health, and control sustainability invite to a smarter use of fungicides, avoiding the residue levels and aiming for an effective delivery,^8^ and to delay the ever-present acquired resistance phenomenon.^9,10^

On the other hand, in the context of integrated pest management,^11^ there is an increasing interest in the use of biopesticides,^12^ which comprises the use of living microorganisms and substances of natural origin, such as plant and microbial extracts, as well as natural products isolated from the above-mentioned sources or closely related derivatives.^13–16^

Therefore, taking into account the above, it is important to develop novel chemical control agents that can be used in the integrated control of *B. cinerea*. These should potentially act on selective biological targets in the infective cycle of the fungus, behaving as fungicidal or fungistatic agents against this phytopathogenic fungus.

Evidence of the protecting role of endophytic microorganisms against the attack of *B. cinerea* on relevant cultivars^17^ stresses the need for chemical control agents with selectivity against the phytopathogen, as they would reduce impact on the plant microbiome.

*B. cinerea* infects host cells by producing toxins and reactive oxygen species and triggering oxidative bursts.^18^ Two families of phytotoxins are produced by *B. cinerea*: (i) botrydial (**1**), dihydrobotrydial (**2**), and related botryanes, toxins with sesquiterpene skeleton and (ii) botcinic (**3**) and botcineric (**4**) acids, and related botcinins (**5**–**8**), with polyketide skeletons (Figure 1).^19^

**Figure 1:**
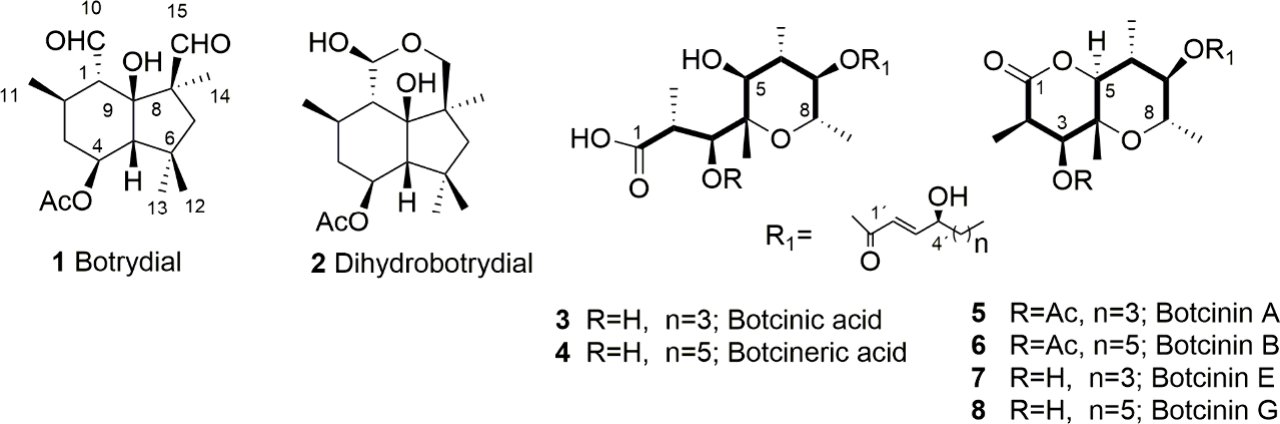
Some toxins excreted by *B. cinerea*.

Botrydial (**1**) is a characteristic phytotoxin of *B. cinerea* that produces chlorosis when applied to plant leaves at 1 ppm, and it is produced by the gray mold in soft rot regions of the infection site.^20^ Botryanes (**1**, **2**) are sesquiterpenes whose underlying carbon skeleton is an irregular sesquiterpene. Both were isolated for the first time by Fehlhaber et al., in 1974 ^21^ from a culture medium of *B. cinerea* (Figure 1).

The other previously mentioned family of phytotoxins is constituted by botcinic (**3**) and botcineric (**4**) acids, and their related botcinins (**5**–**8**), which possess a polyketide chain with eight carbon atoms (Figure 1). These compounds have been described as phytotoxins that caused chlorosis and necrosis on plants.^22–24^

A study of the aggressiveness and production of toxins in the wild population of *B. cinerea* suggests that the strains that produce both botrydial (**1**) and botcinic acid (**3**) are more virulent than the strains that produce only botrydial (**1**). Thus, synergistic behavior of these toxins has been observed in the infection mechanism of *B. cinerea*.^25^

In this context, Pinedo et al.^26^ showed that the fungus *B. cinerea* uses both families of toxins, botryanes and botcinins, as chemicals weapons during the infection process. Both toxins had a redundant function in highly virulent strains, where botcinic acid (**3**) and related toxins were able to compensate for the absence of botrydial (**1**) by an unknown regulatory mechanism, causing host cell death and facilitating invasion and the infective process by the fungus.^26^

On the other hand, Dalmais et al. confirmed the redundant role in virulence of both toxins.^27^ Deletion of the main genes bcbot2 and bcBOA6, involved in the biosynthesis of toxins **1** and **3**, resulted in null mutants which abolished the production of both toxins, and they were nonvirulent or with a decreased virulence.^27^

The above results have allowed us to make a rational approach to the selective control of this phytopathogenic fungus, through a strategy we have named the Fungicide Biosynthetic Design, which consists of the inhibition of biosynthetic pathways for the production of toxins involved in the infection mechanism, as a rational and selective alternative to the control of infections produced by *B. cinerea*.^28^

The inhibition of the biosynthetic pathways that lead to the production of these toxins, using analogues to biosynthetic intermediates, has allowed control of the fungus and its pathogenicity.^28^ The design, synthesis, and computational studies of hybrid molecules,^29,30^ bearing a potential sesquiterpene cyclase (STC) inhibitor and an inhibitor of polyketide synthases (PKS), have allowed us to obtain very efficient antifungal compounds against the phytopathogen *B. cinerea*.^28^

A review of the literature looking for new and more efficient hybrid molecules which displayed antifungal effects against *B. cinerea* ^31^ allowed us to select the molecules **9**–**15** as potential lead compounds.

The colletotrichins (**9**–**11**) are metabolites biosynthesized by the fungal species of the *Colletotrichum* genus, *C. nicotianae* and *C. capsici*. These metabolites are phytotoxins, and they have an important role in the infectious processes (Figure 2).^32–35^ Their structures are composed of a *nor*-diterpene linked to a polysubstituted γ-pyrone, which would meet the potential structural requirements to act as inhibitors of STC and PKS, respectively.

**Figure 2:**
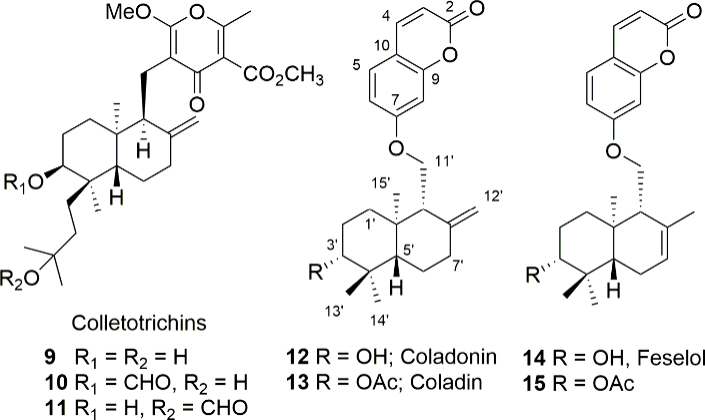
Colletotrichins (**9**–**11**), coladonin (**12**), and feselol (**14**) as natural products-based fungal growth inhibitors models.

On the other hand, coumarins and 4-substituted coumarins are a wide group of biologically active compounds, which have displayed interesting antifungal activity against *B. cinerea*.^36^ Among them, the sesquiterpene coumarins isolated from many plants and herbs^37^ have been reported for their wide range of biological activity, and some of them have been evaluated as squalene-hopene cyclase (SHC) inhibitors.^38^ All these reported data encouraged us to synthesize analogues of natural products which could be considered hybrid molecules, such as colletotrichins (**9**–**11**),^32–35^ coladonin (**12**), also named colladonin in the literature,^37,39,40^ and feselol (**14**)^37,41^ as potential antifungal agents (Figure 2).

Therefore, in this paper, we report the synthesis of racemic analogues of the hybrid molecules **9**–**14**, and the evaluation of their antifungal properties against *B. cinerea*.

## 2 Results and Discussion

### 2.1 Synthesis of Farnesyloxy-Arene, Epoxy Farnesyloxy-Arene and Drimanyloxy-Arene Compounds as Analogues of Hybrid Molecules **9**–**14**

In order to undertake the synthesis of analogues of the hybrid molecules **9**–**14**, as potential fungal control agents, designed on the hypothesis of a synergistic action due to the presence of fragments in the hybrid molecules aimed at interacting with the biosynthetic pathways of two virulence factors in *B. cinerea*,^26^ a biomimetic synthesis approach was adopted (Figure 3),^42^ where drimanyloxy-arenes were derived from a cyclization of distal epoxyfarnesyloxy-arenes, obtained, in turn, from farnesyloxy-arenes. To this end, phenol derivatives umbelliferone (**16**), 4-methyl-umbelliferone (**17**), 2,4-dihydroxy-acetophenone (**18**), 2-hydroxyacetophenone (**19**), and 2,2-biphenol (**20**) were etherified to (*E*,*E*)-farnesyl bromide, to yield farnesyloxy-arenes **21** to **25**, and which after conversion into distal epoxydes (±)-**26** to (±)-**30**, they were transformed into drimanyloxy-arene derivatives (±)-**12**, (±)-**14**, (±)-**31** to (±)-**38**, through Nugent’s reagent-mediated tandem radical cyclization.^43,44^ This biomimetic methodology allows the preparation of farnesyloxy-arene derivatives, and their distal epoxides, on the way to the targeted drimanyloxy-arene derivatives, so their antifungal activities could also be evaluated (Figures 4, 5, and 6).^37,45^

**Figure 3:**
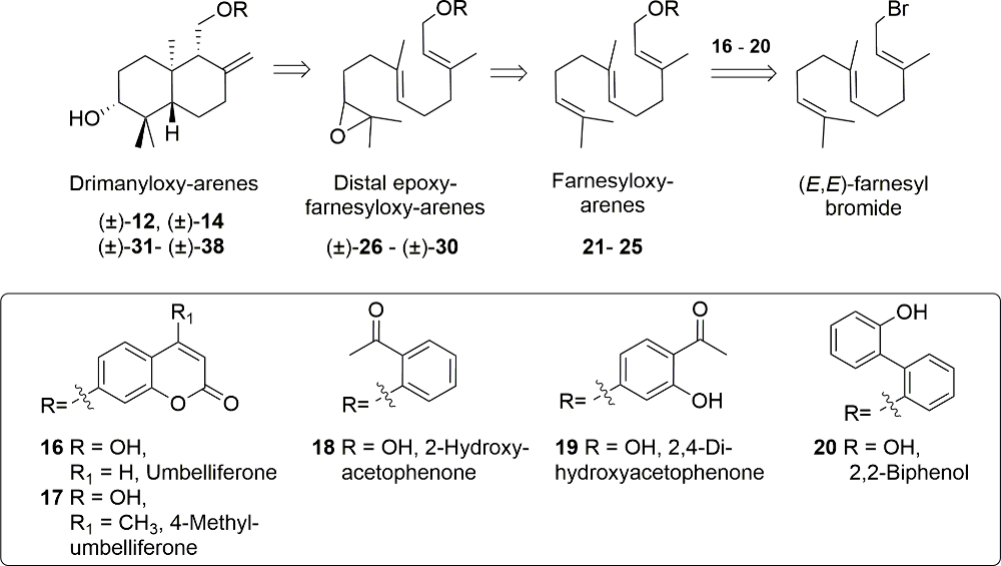
Retrosynthetic scheme to drimanyloxy-derivatives (±)-**12**, (±)-**14**, (±)-**31** to (±)-**38**, from phenol derivatives **16**–**20** and (*E*,*E*)-farnesyl bromide.

**Figure 4:**
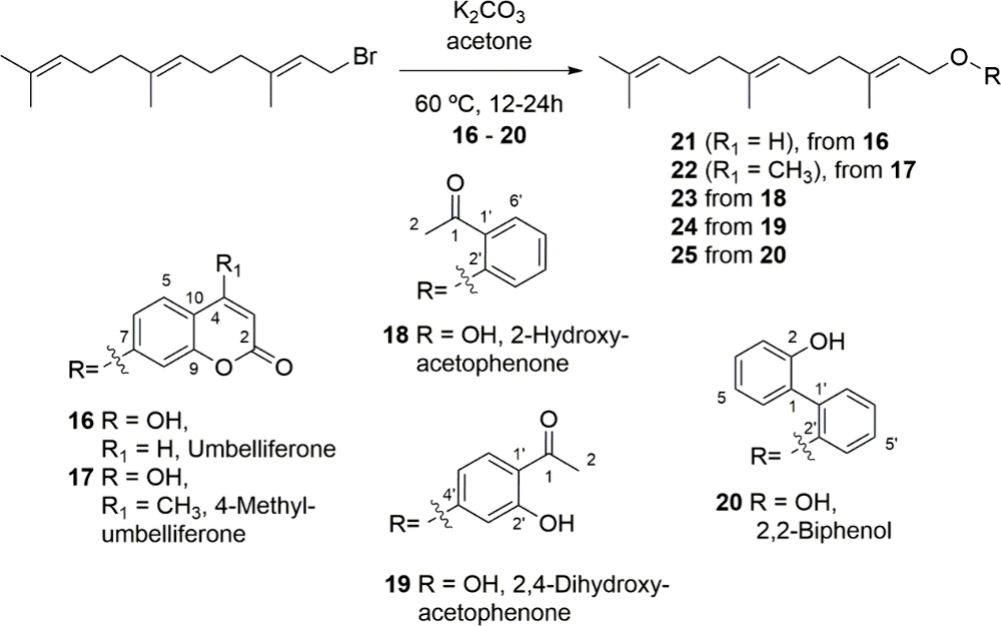
Synthesis of farnesyl derivatives **21** to **25**.

**Figure 5:**
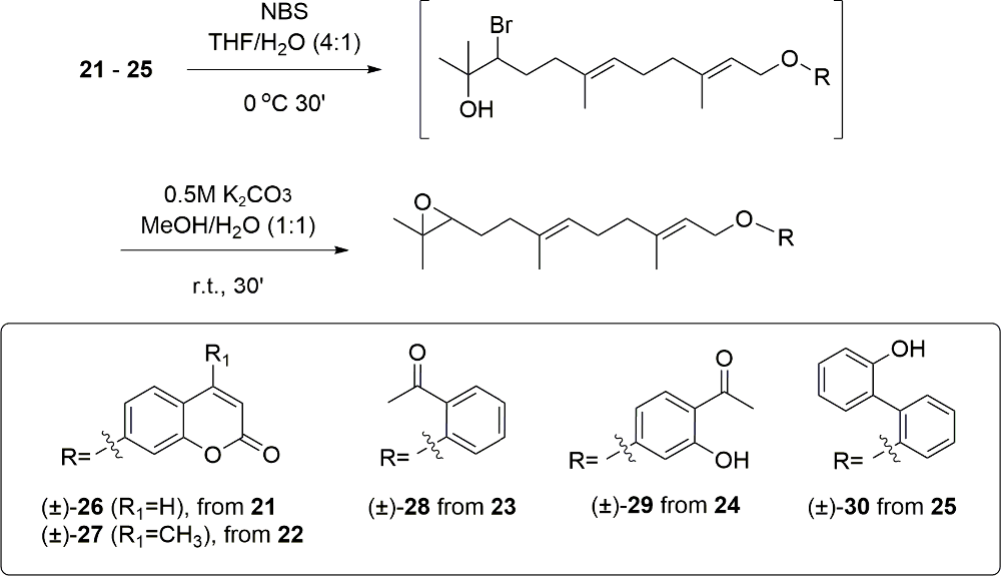
Synthesis of epoxyfarnesyl derivatives (±)-**26**–(±)-**30**.

**Figure 6:**
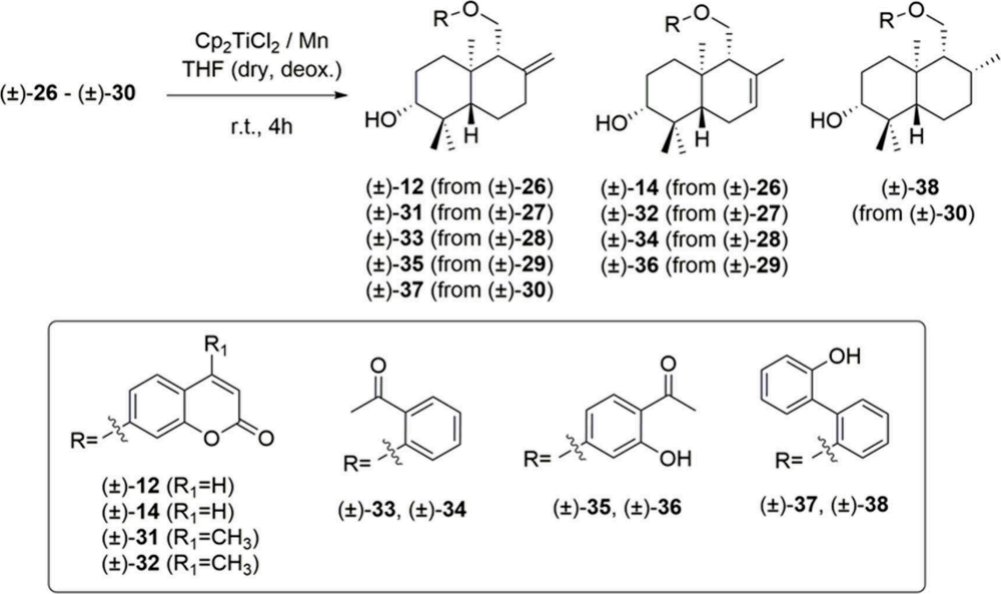
Synthesis of drimanyloxy arenes (±)-**12**, (±)-**14**, (±)-**31** to (±)-**38**.

Therefore, farnesyloxy-arene derivatives **21** to **25** were obtained by treatment of (*E*,*E*)-farnesyl bromide with potassium carbonate in acetone at 60 °C (Figure 4). Subsequent reaction with *N*-bromosuccinimide (NBS) at 0 °C for 30 min, followed by *in situ* reaction with potassium carbonate (0.5 M in MeOH/H_2_O (1:1)), yielded the corresponding epoxy derivatives (±)-**26** to (±)-**30**, via the corresponding bromohydrines (not isolated) (Figure 5).

The spectroscopic data for compounds **21** and **22** were consistent with those reported in the literature for umbelliprenin and 7-farnesyloxy-4-methylumbelliferone, respectively.^37,46,47^ Their updated ^1^H and ^13^C NMR data are shown in Tables [Table tbl1_1H] and [Table tbl2_1H] and Tables [Table tbl1_13C] and [Table tbl2_13C], respectively. The ^1^H and ^13^C NMR data for compounds **23** to **25**, (Table [Table tbl3_1H] to Tables [Table tbl4_1H] and Table [Table tbl3_13C] to Table [Table tbl4_13C]), showed patterns of signals similar to those of farnesyloxycoumarin derivatives **21** and **22**, except in the zone of aromatic signals, confirming the presence of a farnesyloxy chain attached to an aromatic moiety. Compound **23** displayed, as the main difference in its ^1^H NMR (Table [Table tbl3_1H]), two signals to δ_H_ 6.96 ppm (dd, *J* = 8.3, 0.8 Hz, 1H) and δ_H_ 7.74 ppm (dd, *J* = 7.5, 1.9 Hz, 1H) which were assigned to H-3′ and H-6′, protons of the aromatic ring. On the other hand, signals δ_H_ 7.43 ppm (ddd, *J* = 8.3, 7.5, 1.9, Hz, 1H) and δ_H_ 6.98 ppm (ddd, *J* = 8.3, 7.5, 0.8 Hz, 1H) corresponded to H-4′ and H-5′ protons. These signals in the aromatic region, along with a methyl resonance δ_H_ 2.63 ppm (s, 3H) in the ^1^H NMR, and a carbonyl group carbon resonance δ_C_ 200.1 ppm, in the ^13^C NMR (Table [Table tbl3_13C]), were consistent with the structure proposed for compound **23** as 1-(2’-(((2’’*E*,6’’*E*)-3,7,11-trimethyldodeca-2,6,10-trien-1-yl)­oxy)­phenyl)­ethanone. A molecular formula of C_23_H_32_O_2_, deduced from its molecular ion peak in its HRMS (ESI^+^), at *m*/*z* 363.2300 (C_23_H_32_O_2_Na [M + Na]^+^) confirmed the proposed structure for **23**.

**1 tbl1_1H:** ^1^H NMR Spectroscopic Data for **21**, (±)-**26**, (±)-**12**, and (±)-**14**

	**21**	(±)-**26**	(±)-**12**	(±)-**14**
H	δ_H_ [Table-fn t1_1Hfn1] mult. (*J* (Hz))	δ_H_ [Table-fn t1_1Hfn1] mult. (*J* (Hz))	δ_H_ [Table-fn t1_1Hfn2] mult. (*J* (Hz))	δ_H_ [Table-fn t1_1Hfn2] mult. (*J* (Hz))
3	6.24, d (9.5)	6.24, d (9.5)	6.25, d (9.6)	6.25, d (9.4)
4	7.63, d (9.5)	7.63, d (9.5)	7.63, d (9.6)	7.63, d (9.4)
5	7.36, d (8.5)	7.36, d (8.5)	7.36, d (9.4)	7.36, d (8.4)
6	6.84, dd (8.5, 2.4)	6.84, dd (8.5, 2.4)	6.82, m	6.84, dd (8.4, 2.5)
8	6.82, d (2.4)	6.82, d (2.4)	6.81, m	6.81, d (2.5)
1’	a, b: 4.61, d (6.0)	a, b: 4.60, d (6.6)	α: 1.82, m	a: 2.01, m
			β: 1.46, m	b: 1.33, td (13.3, 4.7)
2’	5.47, t (6.0)	5.46, td (6.6, 1.3)	a: 1.73, m	a, b: 1.63
			b: 1.63, m	
3′	-	-	β: 3.31, dd (11.8, 4.4)	β: 3.29 dd (11.2, 4.6)
4’	a, b: 2.10	a, b: 2.07	-	-
5′	a, b: 2.10	a, b: 2.16	β: 1.18, dd (12.6, 2.7)	β: 1.28, dd (11.4, 5.3)
6’	5.08, m	5.15, t (6.3)	a: 1.76, m	a: 2.04, m
			b: 1.46, m	b: 1.67, m
7’	-	-	α: 2.47 ddd (13.5, 4.3, 2.4)	5.55, s (br)
			β: 2.10 td (13.5, 5.2)	
8’	a, b: 1.96	a, b: 2.07	-	-
9’	a, b: 2.05	a, b: 1.58	β: 2.21, m	β: 2.22, s(br)
10’	5.08, m	2.69, t (6.2)	-	-
11’	-	-	a, b: 4.20	a: 4.16, dd (9.7, 3.4)
				b: 4.01, dd (9.7, 5.9)
12’	1.61, s	1.25, s	a: 4.92, sb: 4.54, s	1.69, s
13’	1.77, s	1.76, s	β: 1.03, s	β: 1.01, s
14’	1.59, s	1.62, s	α: 0.82, s	α: 0.89, s
15’	1.68, s	1.29, s	α: 0.85, s	α: 0.91, s

a CDCl_3_ (400 MHz);

b CDCl_3_ (500 MHz).

**2 tbl1_13C:** ^13^C NMR Spectroscopic Data for **21**, (±)-**26**, (±)-**12**, and (±)-**14**

	**21**	(±)-26	(±)-12	(±)-14
C	δC[Table-fn t1_13Cfn1], mult.	δC[Table-fn t1_13Cfn1], mult.	δC[Table-fn t1_13Cfn2], mult.	δC[Table-fn t1_13Cfn2], mult.
2	161.2, C	161.3, C	161.2, C	161.2, C
3	113.1, CH	113.0, CH	113.0, CH	113.1, CH
4	143.3, CH	143.4, CH	143.4, CH	143.4, CH
5	128.5, CH	128.7, CH	128.7, CH	128.7, CH
6	112.8, CH	113.2, CH	113.1, CH	113.0, CH
7	162.0, C	162.1, C	162.2, C	162.0, C
8	101.7, CH	101.5, CH	101.3, CH	101.3, CH
9	155.8, C	155.9, C	155.9, C	155.9, C
10	112.3, C	112.4, C	112.5, C	112.5, C
1’	65.5, CH_2_	65.5, CH_2_	37.2, CH_2_	37.8, CH_2_
2’	118.3, CH	118.5, CH	27.7, CH_2_	27.3, CH_2_
3′	142.2, C	142.2, C	78.5, CH	78.9, CH
4’	39.6, CH_2_	39.4, CH_2_	39.2, C	38.7, C
5′	25.7, CH_2_	26.1, CH_2_	54.8, CH	49.4, CH
6’	123.6, CH	124.1, CH	23.5, CH_2_	23.3, CH_2_
7’	135.6, C	134.7, C	37.4, CH_2_	123.7, CH
8’	39.7, CH_2_	36.3, CH_2_	146.2, C	132.3, C
9’	26.1, CH_2_	27.4, CH_2_	54.3, CH	53.8, CH
10’	124.4, CH	64.1, CH	38.8, C	35.9, C
11’	131.3, C	58.3, C	65.7, CH_2_	67.0, CH_2_
12’	17.7, CH_3_	18.7, CH_3_	107.8, CH_2_	21.6, CH_3_
13’	16.8, CH_3_	16.8, CH_3_	28.3, CH_3_	28.0, CH_3_
14’	16.1, CH_3_	16.0, CH_3_	15.3, CH_3_	15.3, CH_3_
15’	26.2, CH_3_	24.9, CH_3_	15.3, CH_3_	14.9, CH_3_

a CDCl_3_ (100 MHz).

b CDCl3 (125 MHz).

**3 tbl2_1H:** ^1^H NMR spectroscopic data for **22**, (±)-**27**, (±)-**31**, and (±)-**32**

	**22**	(±)-27	(±)-**31**	(±)-32
H	δ_H_ [Table-fn t2_1Hfn1] mult. (J (Hz))	δ_H_ [Table-fn t2_1Hfn2] mult. (J (Hz))	δ_H_ [Table-fn t2_1Hfn2] mult. (J (Hz))	δ_H_ [Table-fn t2_1Hfn2] mult. (J (Hz))
3	6.07, d (1.2)	6.12, s(br)	6.13, d (1.1)	6.13, d (1.1)
5	7.44, d (8.8)	7.48, d (8.8)	7.48, d (8.8)	7.48, d (8.7)
6	6.82, dd (8.8, 2.5)	6.86, dd (8.8, 2.4)	6.84, m	6.84, dd (8.7, 2.5)
7	-	-	-	-
8	6.76, d (2.5)	6.81, d (2.4)	6.83, m	6.81, d (2.5)
11	2.35, s	2.39, s	2.39, s	2.39, s
1’	a, b: 4.56, d (6.5)	a, b: 4.60, d (6.6)	a: 1.82, m	a: 2.02, m
			b: 1.45, m	b: 1.35, m
2’	5.43, td (6.5, 1.2)	5.46, t (6.6)	a: 1.73, m	a, b: 1.65
			b: 1.63, m	
3′	-	-	β: 3.30, dd (11.5, 4.4)	β: 3.29 d(br) (10.2)
4’	a, b: 2.10	a, b: 2.07	-	-
5′	a, b: 2.10	a, b: 2.14	β: 1.18, dd (12.6, 2.7)	β: 1.28, m
6’	5.04, m	5.15, t (6.3)	a: 1.76, m	a, b: 2.02
			b: 1.45, m	
7’	-	-	α: 2.47, ddd (13.4, 4.2, 2.4)	5.55, s(br)
			β: 2.10, td (13.4, 5.0)	
8’	a, b: 1.92	a, b: 2.07	-	-
9’	a, b: 2.01	a, b: 1.58	β: 2.21, m	β: 2.22, s(br)
10’	5.04, m	2.68, t (6.2)	-	-
11’	-	-	a, b: 4.20	a: 4.16, dd (9.7, 3.4), b: 4.01, dd (9.7, 6.0)
12’	1.57, s	1.25, s	a: 4.92, s (br)	1.69, s
			b: 4.55, s (br)	
13’	1.73, s	1.76, s	β: 1.03, s	β: 1.01, s
14’	1.55, s	1.62, s	α: 0.82, s	α: 0.89, s
15’	1.64, s	1.29, s	α: 0.85, s	α: 0.91, s

a CDCl_3_ (400 MHz);

b CDCl_3_ (500 MHz).^1^H NMR, ^13^C NMR, HRESIMS, HSQC and HMBC spectra can be found in Supporting Information for compounds (±)-**26** - (±)-**30** (Figures S6a–S10a, S6b–S10b, S6c–S10c, S6d–S10d and S6e–S10e).

**4 tbl2_13C:** ^13^C NMR Spectroscopic Data for **22**, (±)-**27**, (±)-**31**, and (±)-**32**

	22	(±)-27	(±)-**31**	(±)-32
C	δ_C_ [Table-fn t2_13Cfn1], mult.	δ_C_ [Table-fn t2_13Cfn1], mult.	δ_C_ [Table-fn t2_13Cfn2], mult.	δ_C_ [Table-fn t2_13Cfn2], mult.
2	161.4, C	161.1, C	161.3, C	161.3, C
3	111.8, CH	111.5, CH	111.9, CH	111.9, CH
4	152.6, C	152.4, C	152.2, C	152.5, C
5	125.4, CH	125.3, CH	125.5, CH	125.5, CH
6	112.9, CH	112.6, CH	112.8, CH	112.8, CH
7	161.9, C	161.7, C	162.0, C	161.8, C
8	101.6, CH	101.3, CH	101.3, CH	101.3, CH
9	155.2, C	155.0, C	155.3, C	155.3, C
10	113.4, C	113.2, C	113.5, C	113.5, C
11	18.6, CH_3_	18.4, CH_3_	18.7 CH_3_	18.7 CH_3_
1’	65.4, CH_2_	65.2, CH_2_	37.2 CH_2_	37.8 CH_2_
2’	118.5, CH	118.4, CH	27.7 CH_2_	27.3 CH_2_
3′	142.3, C	141.8, C	78.5 CH	78.9 CH
4’	39.5, CH_2_	39.2, CH_2_	39.2 C	38.7 C
5′	26.1, CH_2_	25.9, CH_2_	54.3 CH	49.4 CH
6’	123.5, CH	123.9, CH	23.4 CH_2_	23.3 CH_2_
7’	135.6, C	134.4, C	37.4 CH_2_	123.7 CH
8’	39.7, CH_2_	36.1, CH_2_	146.3 C	132.3 C
9’	26.7, CH_2_	27.2, CH_2_	54.8 CH	53.8 CH
10’	124.3, CH	63.9, CH	38.8 C	35.8 C
11’	131.3, C	58.1, C	65.6 CH_2_	66.9 CH_2_
12’	17.7, CH_3_	18.7, CH_3_	107.8 CH_2_	21.6 CH_3_
13’	16.8, CH_3_	16.6, CH_3_	28.3 CH_3_	28.0 CH_3_
14’	16.0, CH_3_	15.8, CH_3_	15.3 CH_3_	15.3 CH_3_
15’	25.7, CH_3_	24.7, CH_3_	15.5 CH_3_	14.9 CH_3_

a CDCl_3_ (100 MHz).

b CDCl_3_ (125 MHz).

**5 tbl3_1H:** ^1^H NMR Spectroscopic Data for **23**, (±)-**28**, (±)-**33**, and (±)-**34**

	23	(±)-28	(±)-33	(±)-34
H	δ_H_ [Table-fn t3_1Hfn1], mult.(J (Hz))	δ_H_ [Table-fn t3_1Hfn1], mult. (J (Hz))	δ_H_ [Table-fn t3_1Hfn2], mult. (J (Hz))	δ_H_ [Table-fn t3_1Hfn3], mult. (J (Hz))
2	2.63, s	2.62, s	2.54, s	2.61, s
3′	6.96, dd (8.3, 0.8)	6.95, dd (8.4, 0.6)	6.97, m	6.95, d (8.4)
4’	7.43, ddd (8.3, 7.5, 1.9)	7.43, ddd (8.4, 7.6, 1.8)	7.44, ddd (8.8, 7.3, 1.9)	7.45, t(br) (8.4)
5′	6.98, ddd (8.3, 7.5, 0.8)	6.99, dd (br) (7.6, 0.9)	6.98, m	6.98, t(br) (7.7)
6’	7.74, dd (7.5, 1.9)	7.73, dd (7.6, 1.8)	7.73, dd (7.7, 1.8)	7.70, dd (7.7, 1.7)
1’’	a, b: 4.64, d (6.5)	a, b: 4.64, d (6.0)	a: 1.84, m	a: 2.00, m
			b: 1.46, m	b: 1.29, m
2’’	5.51, t (6.5)	5.51, t (6.0)	a: 1.72, m	a, b: 1.65
			b: 1.63, m	
3′’	-	-	β: 3.31, dd (11.7, 4.1)	β: 3.30, m
4’’	a, b: 2.12	a, b: 2.11	-	-
5′’	a, b: 2.12	a: 2.16, m	β: 1.20, dd (12.5, 2.7)	β: 1.29, m
		b: 2.05, m		
6’’	5.09, m	5.16, t (6.2)	a: 1.78, m	a, b: 2.05
			b: 1.46, m	
7’’	-	-	a: 2.47, ddd (13.3, 4.2, 2.4)	5.57, s(br)
			b: 2.08, m	
8’’	a, b: 1.96	a, b: 2.16	-	-
9’’	a, b: 2.04	a, b: 1.59	β: 2.25, d (br) (7.0)	β: 2.24, s (br)
10’’	5.09, m	2.69, t (6.2)	-	-
11’’	-	-	a: 4.27, dd (9.4, 3.4)	a, b: 4.14
			b: 4.18, t (9.4)	
12’’	1.59, s	1.25, s	a: 4.93, s	1.72, s
			b: 4.57, s	
13’’	1.75, s	1.74, s	β: 1.03, s	β: 1.01, s
14’’	1.60, s	1.62, s	α: 0.82, s	α: 0.89, s
15’’	1.67 d, (1.1)	1.30, s	α: 0.84, s	α: 0.91, s

a CDCl_3_ (400 MHz).

b CDCl_3_ (500 MHz).

c CDCl_3_ (600 MHz).

**6 tbl3_13C:** ^13^C NMR Spectroscopic Data for **23**, (±)-**28**, (±)-**33**, and (±)-**34**

	**23**	(±)-**28**	(+)-**33**	(+)-**34**
C	δ_C_ [Table-fn t3_13Cfn1], mult.	δ_C_ [Table-fn t3_13Cfn1], mult.	δ_C_ [Table-fn t3_13Cfn2], mult	δ_C_ [Table-fn t3_13Cfn3], mult
1	200.1, C	200.0, C	200.1, C	200.2, C
2	32.0, CH_3_	32.0, CH_3_	32.1, CH_3_	32.0, CH_3_
1’	128.5, C	128.6, C	128.4, C	128.7, C
2’	158.3, C	158.3, C	158.3, C	157.9, C
3′	112.7, CH	112.7, CH	112.0, CH	112.0, CH
4’	133.5, CH	133.5, CH	133.6, CH	133.5, CH
5′	120.4, CH	120.4, CH	120.4, CH	120.5, CH
6’	130.4, CH	130.3, CH	130.4, CH	130.3, CH
1’’	65.3, CH_2_	65.4, CH_2_	37.3, CH_2_	37.9, CH_2_
2’’	119.0, CH	119.1, CH	27.7, CH_2_	27.2, CH_2_
3′’	141.6, C	141.5, C	78.5, CH	79.0, CH
4’’	39.4, CH_2_	39.4, CH_2_	39.2, C	38.7, C
5′’	26.1, CH_2_	26.2, CH_2_	54.3, CH	49.3, CH
6’’	123.5, CH	124.1, CH	23.5, CH_2_	23.2, CH_2_
7’’	135.5, C	134.6, C	37.3, CH_2_	123.8, CH
8’’	39.7, CH_2_	36.2, CH_2_	146.1, C	132.5, C
9’’	26.7, CH_2_	27.4, CH_2_	55.3, CH	54.3, CH
10’’	124.2, CH	64.1, CH	38.6, C	35.9, C
11’’	131.3, C	58.3, C	65.1, CH_2_	66.6, CH_2_
12’’	17.7, CH_3_	18.7, CH_3_	107.9, CH_2_	21.5, CH_3_
13’’	16.7, CH_3_	16.7, CH_3_	28.3, CH_3_	28.0, CH_3_
14’’	16.0, CH_3_	16.0, CH_3_	15.5, CH_3_	15.3, CH_3_
15’’	25.7, CH_3_	24.9, CH_3_	15.4, CH3	15.1, CH_3_

a CDCl_3_ (100 MHz).

b CDCl_3_ (125 MHz).

c CDCl_3_ (150 MHz).

The molecular formula for compound **24** was established as C_23_H_32_O_3_ based on an observed protonated molecular ion in its HRESIMS at *m*/*z* 357.2434 (calculated for C_23_H_33_O_3_ [M + H]^+^ 357.2430). This was consistent with ^13^C NMR (Table [Table tbl5_13C]) and HSQC data (Figure S4d). ^1^H NMR spectrum (Table [Table tbl5_1H]) presented, in addition to the signals corresponding to the farnesyl chain, a resonance at δ_H_ 6.41 ppm (d, *J* = 2.4 Hz, 1H) which was assigned to H-3′, and two additional resonances at δ_H_ 6.43 ppm (dd, *J* = 8.8, 2.4 Hz, 1H), and δ_H_ 7.60 ppm (d, *J* = 8.8 Hz, 1H), respectively, which were assigned to H-5′ and H-6′. These observations, together with presence of a methyl signal at δ_H_ 2.53 ppm (s, 3H) were consistent with the proposed structure for **24** as 1-(2’-hydroxy-4’-(((2’’*E*,6’’*E*)-3,7,11-trimethyldodeca-2,6,10-trien-1-yl)­oxy)­phenyl)­ethanone. This was confirmed by 2D NMR experiments. Thus, the observed correlation in its HMBC spectrum (Figure S4e), between H_2_-1́́ of the farnesyl chain and C-4′ of the aromatic ring, confirmed the connection between the farnesyl chain and the acetophenone moiety through an ether link.

**7 tbl4_1H:** ^1^H NMR Spectroscopic Data for **24**, (±)-**29**, (±)-**35**, and (±)-**36**

	**24**	(±)-**29**	(±)-**35**	(±)-**36**
H	δ_H_ [Table-fn t4_1Hfn1], mult. (J (Hz))	δ_H_ [Table-fn t4_1Hfn1], mult. (J (Hz))	δ_H_ [Table-fn t4_1Hfn2], mult. (J (Hz))	δ_H_ [Table-fn t4_1Hfn1], mult. (J (Hz)
2	2.53 s	2.53, s	2.54, s	2.55, s
3′	6.41, d (2.4)	6.40, d (2.5)	6.42, m	6.42, m
5′	6.43, dd (8.8, 2.4)	6.43, dd (8.9, 2.5)	6.41, m	6.41, m
6’	7.60, d (8.8)	7.60, d (8.9)	7.61, d (9.6)	7.61, d (8.1)
1’’	a, b: 4.56, d (6.1)	a, b: 4.55, d (6.6)	a: 1.77, m	a: 2.00, m
			b: 1.44, m	b: 1.33, m
2’’	5.45, t (6.1)	5.44, t (6.6)	a: 1.71, m	a, b: 1.63
			b: 1.62, m	
3′’	-	-	β: 3.29, dd (11.6, 4.0)	β: 3.28, dd (10.8, 4.3)
4’’	a, b: 2.10	a, b: 2.07	-	-
5′’	a, b: 2.15	a, b: 2.14	β: 1.17, dd (12.4, 1.7)	β: 1.27, m
6’’	5.09, m	5.14, t (6.1)	a: 1.77, m	a, b: 2.03
			b: 1.44, m	
7’’	-	-	a: 2.45, d(br) (13.0)	5.54, s (br)
			b: 2.09, td (13.0, 5.0)	
8’’	a, b: 1.96	a, b: 2.07	-	-
9’’	a, b: 2.04	a, b: 1.57	β: 2.18, m	β: 2.17, m
10’’	5.09, m	2.68, t (6.2)	-	-
11’’	-	-	a, b: 4.16, ddc	a: 4.13 dd (9.8, 3.3)
				b: 3.98, dd (9.8, 6.0)
12’’	1.59, s	1.24, s	a: 4.90, s	1.67, s
			b: 4.51, s	
13’’	1.74, s	1.72, s	β: 1.02, s	β: 1.00, s
14’’	1.59, s	1.61, s	α: 0.81, s	α: 0.88, s
15’’	1.67, s	1.28, s	α: 0.83, s	α: 0.88, s

a CDCl_3_ (400 MHz).

b CDCl_3_ (500 MHz).

**8 tbl4_13C:** ^13^C NMR Spectroscopic Data **24**, (±)-**29**, (±)-**35**, and (±)-**36**

	**24**	(±)-**29**	(±)-**35**	(±)-**36**
C	δ_C_ [Table-fn t4_13Cfn1], mult.	δ_C_ [Table-fn t4_13Cfn1], mult.	δ_C_ [Table-fn t4_13Cfn2], mult.	δ_C_ [Table-fn t4_13Cfn1], mult.
1	202.3, C	202.4, C	202.5, C	202.5, C
2	26.1, CH_3_	26.1, CH_3_	26.2, CH_3_	26.2, CH_3_
1’	113.6, C	113.7, C	113.8, C	113.8, C
2’	165.1, C	165.1, C	165.2, C	165.3, C
3′	101.4, CH	101.4, CH	101.2, CH	101.3, CH
4’	165.4, C	165.4, C	165.5, C	165.3, C
5′	108.0, CH	108.1, CH	108.2, CH	108.1, CH
6’	132.1, CH	132.2, CH	132.2, CH	132.2, CH
1’’	65.1, CH_2_	65.1, CH_2_	37.2, CH_2_	37.8, CH_2_
2’’	118.4, CH	118.5, CH	27.7, CH_2_	27.3, CH_2_
3′’	142.0, C	141.9, C	78.5, CH	78.9, CH
4’’	39.6, CH_2_	39.3, CH_2_	39.2, C	38.7, C
5′’	26.1, CH_2_	26.0, CH_2_	54.3, CH	49.4, CH
6’’	123.4, CH	124.1, CH	23.4, CH_2_	23.3, CH_2_
7’’	135.1, C	134.6, C	37.4, CH_2_	123.6, CH
8’’	39.4, CH_2_	36.2, CH_2_	146.2, C	132.4, C
9’’	26.6, CH_2_	27.4, CH_2_	54.7, CH	53.8, CH
10’’	124.2, CH	64.0, CH	38.8, C	35.8, C
11’’	131.2, C	58.2, C	65.4, CH_2_	66.7, CH_2_
12’’	17.6, CH_3_	18.7, CH_3_	107.8, CH_2_	21.6, CH_3_
13’’	16.6, CH_3_	16.6, CH_3_	28.3, CH_3_	28.0, CH_3_
14’’	15.9, CH_3_	16.0, CH_3_	15.3, CH_3_	14.8, CH_3_
15’’	25.6, CH_3_	24.8, CH_3_	15.5, CH_3_	15.25, CH_3_

a CDCl_3_ (100 MHz).

b CDCl_3_ (125 MHz).

**9 tbl5_1H:** ^1^H NMR Spectroscopic Data **25**, (±)-**30**, (±)-**37**, and (±)-**38**

	**25**	(±)-**30**	(±)-**37**	(±)-**38**
H	δ_H_ [Table-fn t5_1Hfn1], mult.(J (Hz))	δ_H_ [Table-fn t5_1Hfn2], mult.(J (Hz))	δ_H_ [Table-fn t5_1Hfn2], mult.(J (Hz))	δ_H_ [Table-fn t5_1Hfn2], mult.(J (Hz))
2	6.73, s (OH)	6.73, s (OH)	6,36, s (OH)	6,35, s (OH)
3	7.03 d (7.5)	7.03, d (7.5)	7.00, d (7.5)	7.00, d (7.5)
4	7.30, m	7.29, m	7.28, m	7.28, m
5	7.00, m	7.00, m	6.98, dd (br) (7.5, 1.5)	6.98, ddd (7.5, 1.2)
6	7.26, m	7.27, m	7.22, dd (7.5, 1.5)	7.22, dd (7.5, 1.5)
3′	7.06, m	7.06, d (8.6)	7.08, d (8.2)	7.08, d (8.3)
4’	7.37, m	7.36 m	7.37, ddd (8.2, 7.5, 1.7)	7.37, m
5′	7.12 t (br) (7.6)	7.12 td (7.6, 1.1)	7.12, td (7.5, 1.0)	7.11, td (7.5, 1.1)
6’	7.35 d (br) (7.6)	7.35, d (7.6)	7.34, dd (7.5, 1.7)	7.34, dd (7.5, 1.5)
1’’	a, b: 4.62 d (6.6)	a, b: 4.62 d (6.6)	a: 1.56, m	a: 1.54, m
			b: 1.17, m	b: 1.03, m
2’’	5.41 t (6.6)	5.40, td (6.6, 1.1)	a, b: 1.49	a, b: 1.49
3′’	-	-	β: 3.18, dd (11.2, 4.5)	β: 3.15, m
4”	a, b: 2.02	a, b: 2.03	-	-
5”	a, b: 2.07	a, b: 2.09	β: 1.10, dd (12.6, 2.7)	β: 0.80, d(br) (12.0)
6”	5.08, m	5.12 dd (7.2, 6.1)	a: 1.73, m	a, b: 1.40
			b: 1.38, m	
7”	-	-	a: 2.42, ddd (13.1, 4.3, 2.5)	a, b: 1.59
			b: 2.03, td (13.1, 5.2)	
8”	a, b: 1.96	a, b: 2.15	-	2.00, m
9”	a, b: 2.02	a, b: 1.60	β: 2.12, dd (7.0, 4.5)	β: 1.59, m
10’’	5.08, m	2.68, t (6.2)	-	-
11’’	-	-	a: 4.31, dd (9.6, 7.0)	a: 4.19, dd (9.4, 5.1)
			b: 4.11, dd (9.6, 4.5)	b: 3.99, t (9.4)
12”	1.59, s	1.25, s	a: 4.87, s	0.84, d (8.0)
			b: 4.54, s	
13”	1.64, s	1.64, s	β: 0.98, s	β: 0.96, s
14’’	1.58, s	1.56, s	α: 0.76, s	α: 0.75, s
15’’	1.68, s	1.29, s	α: 0.73, s	α: 0.86, s

a CDCl_3_ (400 MHz).

b CDCl_3_ (500 MHz).

**10 tbl5_13C:** ^13^C NMR Spectroscopic Data **25**, (±)-**30**, (±)-**37**, and (±)-**38**

	**25**	(±)-**30**	(±)-**37**	(±)-**38**
C	δ_H_ [Table-fn t5_13Cfn1], mult	δ_H_ [Table-fn t5_13Cfn2], mult.	δ_H_ [Table-fn t5_13Cfn2], mult.	δ_H_ [Table-fn t5_13Cfn2], mult.
1	126.7, C	126.7, C	126.8, C	126.5, C
2	154.0, C	154.0, C	153.9, C	153.7, C
3	117.9, CH	117.9, CH	117.8, CH	117.3, CH
4	129.1, CH	129.1, CH	129.0, CH	129.1, CH
5	120.9, CH	120.9, CH	120.9, CH	120.8, CH
6	131.3, CH	131.3, CH	131.4, CH	131.2, CH
1’	128.4, C	128.3, C	128.1, C	127.9, C
2’	154.5, C	154.5, C	155.0, C	155.2, C
3′	114.0, CH	114.0, CH	113.1, CH	113.6, CH
4’	129.0, CH	129.0, CH	129.1, CH	129.1, CH
5′	122.5, CH	122.5, CH	122.4, CH	122.3, CH
6’	132.6, CH	132.6, CH	132.6, CH	132.4, CH
1’’	66.7, CH_2_	66.6, CH_2_	36.9, CH_2_	38.0, CH_2_
2’’	118.5, CH	118.7, CH	27.6, CH_2_	26.9, CH_2_
3′’	142.5, C	142.2, C	78.5, CH	78.6, CH
4”	39.7, CH_2_	39.4, CH_2_	39.1, C	38.9, C
5”	26.2, CH_2_	26.1, CH_2_	54.0, CH	55.1, CH
6”	123.5, CH	124.2, CH	23.4, CH_2_	17.1, CH_2_
7”	135.5, C	134.6, C	37.3, CH_2_	34.1, CH_2_
8”	39.5, CH_2_	36.3, CH_2_	146.7, C	29.2, CH
9”	26.7, CH_2_	27.2, CH_2_	54.9, CH	52.1, CH
10”	124.3, CH	64.2, CH	38.6, C	37.1, C
11”	131.3, C	58.3, C	66.8, CH_2_	69.2, CH_2_
12’’	17.7, CH_3_	18.7, CH_3_	107.2, CH_2_	15.7, CH_3_
13’’	16.5, CH_3_	16.5, CH_3_	28.2, CH_3_	28.1, CH_3_
14’’	16.0, CH_3_	16.0, CH_3_	15.4, CH_3_	15.4, CH_3_
15’’	25.7, CH_3_	24.9, CH_3_	15.1, CH_3_	17.0, CH_3_

a CDCl_3_ (100 MHz).

b CDCl_3_ (125 MHz).

Compound **25** showed a molecular ion in its HRESIMS at *m*/*z* 391.2623 (calculated for C_27_H_35_O_2_ [M + H]^+^, 391.2637) which is consistent with a molecular formula of C_27_H_34_O_2_. ^1^H NMR and ^13^C NMR data (Tables [Table tbl4_1H] and [Table tbl4_13C]) showed a complex pattern of resonances in the aromatic zone that could be assigned using a combination of 2D NMR techniques. This, together with the observation of HMBC correlations (Figure S5e), which allows us to establish the connection of the farnesyl moiety at H-1′´ with one of the phenolic groups in 2,2-biphenol, leads to the confirmation of the structure of compound **25** as 2’-(((2’’*E*,6’’*E*)-3,7,11-trimethyldodeca-2,6,10-trien-1-yl)­oxy)-[1,1’-biphenyl]-2-ol.

The structures of the corresponding epoxidized derivatives (±)-**26** to (±)-**30** were established by extensive NMR studies, including 2D NMR techniques, and HESIRMS. The comparison of ^1^H NMR spectra for epoxides and parent olefins (see Tables [Table tbl1_1H] to [Table tbl4_1H], Figures S1a–S5a for parent olefins and Figures S6a–S10a for epoxides), showed the disappearance of the characteristic vinyl signal of the farnesyl chain at C-10 (for instance at δ_H_ 5.08 ppm in compound **21**, Table [Table tbl1_1H], Figure S1a), and the appearance of a signal corresponding to a proton bound to an epoxide function (for instance, at δ_H_ 2.69 ppm (t, *J* = 6.2 Hz, 1H) in compound (±)-**26**, Table [Table tbl1_1H], Figure S6a). On the other hand, comparison of ^13^C NMR spectra (see Tables [Table tbl1_13C] to [Table tbl4_13C], Figures S1b–S5b for parent olefins and Figures S6b–S10b for epoxides) showed disappearance of olefinic carbon resonances (for instance, signals at δ_C_ 124.4 ppm (CH) and δ_C_ 131.3 ppm (C) in compound **21**, Table [Table tbl1_13C], Figure S1b) and appearance of a methine and quaternary alkyl carbons linked to oxygen (for instance, signals at δ_C_ 64.1 ppm (CH) and δ_C_ 58.3 ppm (C) for compound (±)-**26**, Table [Table tbl1_13C], Figure S6b).

For the biomimetic synthesis of drimane skeleton derivatives, Nugent’s reagent (Cp_2_TiCl) was selected,^43^ following the methodology used previously by Barrero et al.,^48^ due to its mild reaction conditions, and the high efficiency shown in the synthesis of natural products.^44^ This method involves radical cyclization of the corresponding epoxypolyene ((±)-**26** to (±)-**30**) by treatment with Cp_2_TiCl_2_/Mn in strictly dry and deoxygenated THF (Figure 6).^49^ The cyclization reaction led to the preparation of the hybrid molecules (±)-**12**, (±)-**14**, and (±)-**31** to (±)-**38** (Figure 6) with moderate yields (see Materials and Methods). It is important to highlight that cyclization of (±)-**30** gave, in addition to the expected exocyclic derivative (±)-**37**, the saturated drimane derivative (±)-**38**, obtained probably due to the proximity at C-8′′ carbon, during the cyclization process, of an acidic proton from the hydroxyl group in the bicyclic aromatic system.^50^ On the other hand, this compound could originate from the presence of water in the reaction mixture, even though, remarkably, similar compounds were not observed in the cyclization of epoxides (±)-**26** to (±)-**29**.

Compounds (±)-**12** and- (±)-**14** were characterized by NMR spectroscopy, showing ^1^H and ^13^C NMR data (Tables [Table tbl1_1H] and [Table tbl1_13C]) consistent with those described in the literature for (−)-coladonin ((−)-**12**) and (+)-feselol ((+)-**14**) (see comparison in Tables S1-1H and S1-13C).^39–41,51^ Relative stereochemistry and resonance structural assignments for obtained compounds (±)-**12** and (±)-**14** were carried out by NOESY2D experiments (see Figure S11f–gand S13f–g, respectively). NOESY correlations between H-3′β/H_3_-13’β, H_3_-13’β/H-5′β, H-5′β/H-9’β, H-9’β/H_2_-11’, and between H_2_-11’/H-1’α, H_3_-15’α and H-12’b, for compound (±)-**12**, and between H-3′β/ H_3_-13’β, H_3_-13’β/H-5′β, H-5′β/H-9’β, H-9’β/H_2_-11’ and between H_2_-11’/ H_3_-15’α/H-12’b, for compound (±)-**14**, were consistent with relative configuration 3′*R*(*S*),5′*S*(*R*),9’*R*(*S*),10’*R*(*S*) for both compounds. On the other hand, this relative stereochemistry assignment can be also extended to compounds (±)-**31**-(±)-**37**, obtained under similar reaction conditions.

Treatment of epoxyfarnesyloxy-4-methyl-coumarin (±)-**27** with Nugent’s reagent,^43^ following the methodology described in the experimental section, yielded the novel 7-drimanyloxy-4-methylcoumarins (±)-**31** and (±)-**32** (Figure 6), with 38% and 16% yields, respectively. Both compounds showed patterns of signals in their ^1^H and ^13^C NMR spectra (Table [Table tbl2_1H] and Table [Table tbl2_13C]), identical to those of compounds (±)-**12** and (±)-**14** except for signals corresponding to the resonance of H-4, which had disappeared in compounds (±)-**31** and (±)-**32**. Instead, a methyl resonance (δ_H_ 2.39, s, 3H) was present in the ^1^H NMR spectra. On the other hand, an additional carbon resonance at δ_C_ 18.7 (CH_3_, C-11) was consistent with the presence of a 4-methyl-umbelliferone moiety. Further spectroscopic data for compounds (±)-**31** and (±)-**32**, along with the correlations observed in HSQC and HMBC spectra (Figures S14d–e, S15d–e) were consistent with the proposed structures. The observed molecular ions for (±)-**31** and (±)-**32**, [M + H]^+^ 397.2371 and 397.2382, which were consistent with molecular formula C_25_H_32_O_4_ for both compounds, finally confirmed the structures of both compounds as (±)-**7**-(3′*R*(*S*),5*’S*(*R*),9’*R*(*S*),10’*R*(*S*)-3′-hydroxydrim-8’(12’)-en-11’-yloxy)-4-methylcoumarin ((±)-**31**) and (±)-7-(3′*R*(*S*), 5′*S*(*R*),9’*R*(*S*), 10’*R*(*S*)-3′-hydroxydrim-7’-en-11’-yloxy)-4-methylcoumarin ((±)-**32**).

In a similar fashion, drimanyloxy-arene derivatives (±)-**33**-(±)-**38** were obtained as described above. On one hand, compounds (±)-**33**, (±)-**35**, and (±)-**37** showed in their ^1^H and ^13^C NMR spectra (Tables [Table tbl3_1H] to [Table tbl4_1H] and [Table tbl3_13C] to [Table tbl4_13C]) signals consistent with a 3-hydroxydrim-8(12)-en-11-yloxy moiety; on the other hand, compounds (±)-**34** and (±)-**36** showed in their ^1^H and ^13^C NMR spectra (Tables [Table tbl3_1H] to [Table tbl5_1H] and [Table tbl3_13C] to [Table tbl5_13C]) signals consistent with a 3-hydroxydrim-7-en-11-yloxy moiety. Examination of NOESY2D spectra (Figures S16f–S19f) for these compounds confirms stereochemistry of the sesquiterpenic moiety as 3*R­(S*),5*S*(*R*),9*R*(*S*),10*R*(*S*).

Isomers (±)-**33** and (±)-**34** showed molecular ions in their HRESIMS spectra, [M + H]^+^ 357.2435 for (±)-**33**, and 357.2420 for (±)-**34**, which were consistent for the molecular formula C_23_H_32_O_3_ for both compounds. Their ^1^H and ^13^C NMR spectra (Tables [Table tbl3_1H] and [Table tbl3_13C]) showed characteristic signals of a sesquiterpene moiety, as mentioned above, and a 2-hydroxyacetophenone fragment. The arene moiety was inferred from the signals observed in the aromatic zone, which were similar to those described for compound **23**. Two signals at δ_H_ 6.97 (m) and 7.73 (dd) ppm for (±)-**33**, and δ_H_ 6.95 (d) and 7.70 ppm (dd) ppm for (±)-**34**, were assigned to H-3′ and H-6′ protons. On the other hand, two signals at δ_H_ 7.44 and 6.98 ppm, assigned to H-4′ and H-5′ protons, were also observed in their ^1^H NMR spectra (Table [Table tbl3_1H]). These aromatic signals, along with a methyl signal at δ_H_ 2.54 ppm (s, 3H) for (±)-**33** and at δ_H_ 2.61 ppm (s, 3H) for (±)-**34**, and a carbonyl resonance at δ_C_ 200.1 for (±)-**33** and at 200.1 ppm for (±)-**34**, in their ^13^C NMR (Table [Table tbl3_13C]), were consistent with a 2-hydroxyacetophenone moiety in both compounds. These observations, along with analysis of the 2D-NMR spectroscopy (Figures S16d–f and S17d–f) led to the assignment of compound (±)-**33** as (±)-1-(2’-(*3′’R*(*S*),5′’*S*(*R*),9’’*R*(*S*),*10’’R*(S)-3′’-hydroxydrim-8’’(12’’)-en-11’’-yloxy)­phenyl)­ethanone and of compound (±)-**34** as (±)-1-(2’-(3′’*R*(*S*),5′’*S*(*R*),9’*’R*(*S*),10’’*R*(S)-3′’-hydroxydrim-7’’-en-11’’-yloxy)­phenyl)­ethanone.

The molecular formula for both isomers (±)-**35** and (±)-**36** was deduced as C_23_H_32_O_4_ from the protonated molecular ion in their HRESIMS spectra ([M + H]^+^ 373.2374 and 373.2382, respectively). This formula was consistent with 8 degrees of unsaturation, which is also consistent for both compounds with ^13^C NMR data (Table [Table tbl5_13C]), and HSQC spectra (Figures S18d and S19d), and suggests the presence of an arene moiety, in addition to the previously mentioned drimane one. The ^1^H NMR spectra for (±)-**35** and (±)-**36** (Table [Table tbl5_1H]) presented resonances at δ_H_ 6.42, 6.41, and 7.61 ppm corresponding to H-3′, H-5′, and H-6′ in a trisubstituted aromatic ring. These signals, along with a methyl signal at δ_H_ 2.55 ppm (s, 3H) and a carbonyl carbon resonance at δ_C_ 202.5 ppm in ^13^C NMR (Table [Table tbl5_13C]), were consistent with a 2,4-dihydroxyacetophenone moiety, as observed in compound **29**. Finally, HMBC correlations between C-4’ and H_2_-11” (see, for instance Figure S18e for compound (±)-**35**) led to the assignment of compound (±)-**35** as (±)-1-(2’-hydroxy-4’-(3′’*R*(*S*),5′’*S*(*R*),9’’*R*(*S*),10’’*R*(*S*)-3′’-hydroxydrim-8’’(12’’)-en-11’’-yloxy)­phenyl)­ethanone and of compound (±)-**36** as (±)-1-(2’-hydroxy-4’-(3′’*R*(*S*),5′’*S*(*R*),9’’*R*(*S*),10’’*R*(*S*)-3′’-hydroxydrim-7’’-en-11’’-yloxy)­phenyl)­ethanone.

The ^1^H and ^13^C NMR spectra of compounds (±)-**37** and (±)-**38** (Tables [Table tbl4_1H] and [Table tbl4_13C]) had a pattern of signals that were characteristic of a drimanyloxy derivative bound to a 2,2′-biphenol group. More specifically, for compound (±)-**37**, its ^1^H and ^13^C NMR spectra (Tables [Table tbl4_1H] and [Table tbl4_13C]) are consistent with a 3-hydroxydrim-8(12)-en-11-yloxy moiety. Therefore, structure 2’-(3′’*R*(*S*),5′’*S*(*R*),9’’*R*(*S*),10’’*R*(*S*)-3′’-hydroxydrim-8’’(12’’)-en-11’’-yloxy)-[1,1’-biphenyl]-2-ol was proposed for compound (±)-**37**. This structure was confirmed by HRESIMS where a molecular formula C_27_H_34_O_3_ was deduced from observed molecular ion ([M + Na]^+^ *m*/*z* 429.2396 (calcd. for C_27_H_34_O_3_Na 429.2406).

On the other hand, for compound (±)-**38**, a molecular formula C_27_H_36_O_3_ was deduced from the observed molecular ion [M + H]^+^ *m*/*z* 409.2733 in its HRESIMS (calcd. for C_27_H_37_O_3_ 409.2743), indicating the presence of an unsaturation degree less than that in compound (±)-**37**. While analysis of signals in its ^1^H and ^13^C NMR spectra suggests that a similar sesquiterpenic framework is present in both compounds, the principal difference with (±)-**37** was the absence of the double bond resonances in both ^1^H and ^13^C NMR spectra. Instead, a signal at δ_H_ 0.84 ppm (d, 3H) and resonances at δ_C_ 29.2 (CH, C-8”) and 15.7 (CH_3_, C-12”), were characteristic of a methyl group (C-12”) adjacent to a methine group (C-8”). Relative stereochemistry and resonance structural assignment for compound (±)-**38** was carried out by NOESY2D experiments (see Figure S21f–g). NOESY correlations between H-3”β, H3–13”β, H-5”β, H-9”β, H-8”β and H3–12”α confirmed structural assignment for (±)-**38** as 2’-(3′’*R*(*S*),5′’*S*(*R*),8’’*R*(*S*),9’’*R*(*S*),10’’*R*(*S*)-3′’-hydroxydriman-11’’-yloxy)-[1,1’-biphenyl]-2-ol. As mentioned above, the formation of this compound can be explained by a transfer of a hydrogen atom to the intermediate drimanyl radical formed at C-8”.^50^

#### 2.1.1 Chemical Transformations of (±)-Coladonin ((±)-**12**)

In order to study structure–activity relationships in the sesquiterpenic moiety, a certain number of derivatives of (±)-coladonin ((±)-**12**) were prepared, so their antifungal activity on the phytopathogenic fungus *B*. *cinerea* could be evaluated (Figure 7). Thus, the acetyl derivative (±)-**13** and the epoxidized derivative (±)-**39** were obtained, in 36% and 75% yields, respectively, from compound (±)-**12** following the procedures described in the Materials and Methods. Furthermore, treatment of (±)-coladonin ((±)-**12**) with triphenylphosphine (PPh_3_) and diisopropylazo-dicarboxylate (DIAD)^52^ caused the elimination of the hydroxyl group at C-3′ position, yielding the dehydroderivative (±)-**40** with a 44% yield.

**Figure 7:**
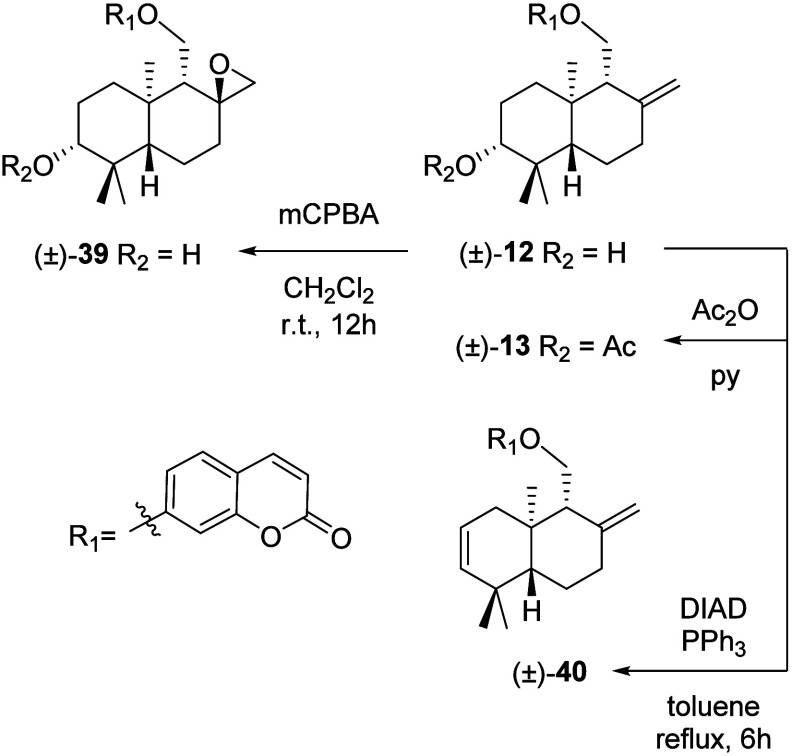
Synthesis of compounds (±)-**13**, (±)-**39**, and (±)-**40**.

Compound (±)-**13** showed a molecular ion in its HRESIMS at *m*/*z* 447.2149 [M + Na]^+^ (calcd. for C_26_H_32_O_5_Na 447.2147) which is consistent with a molecular formula C_26_H_32_O_5_ for this compound. Comparison with molecular formula for (±)-**12** (C_24_H_30_O_4_) suggests that a CH_3_CO group has been incorporated. Acetylation of compound (±)-**12** is further confirmed by the observation of the displacement of resonance for H-3′ from δ_H_ 3.31 ppm in (±)-**12** to 4.55 ppm in (±)-**13** and by the observations of resonances at δ_C_ 80.3 ppm (CH, C-3′), 21.3 ppm (CH_3_CO) and 170.9 ppm (CH_3_ CO) in the ^13^C NMR of (±)-**13**, which are consistent with an acetate moiety, substituted at C-3′.

Compound (±)-**39** showed a molecular ion in its HRESIMS at *m*/*z* 399.2178 [M + H]^+^ (calcd. for C_24_H_30_O_5_ 399.2171), which is consistent with a molecular formula C_24_H_30_O_5_ for this compound. Comparison with molecular formula for (±)-**12** (C_24_H_30_O_4_) suggests that an oxygen has been incorporated. Epoxidation of compound (±)-**12** is further confirmed by the observation of the absence of olefin resonances at ^1^H and ^13^C NMR spectra for (±)-**39** and by observation of a resonance for H_2_-12’ at δ_H_ 2.65 ppm and by the observations of resonances at δ_C_ 57.5 ppm (C, C-8’) and 51.9 ppm (CH_2_, C-12’) in the ^13^C NMR of (±)-**39**, which are consistent with an epoxide moiety on C-8’-C-12’. Relative stereochemistry and resonance structural assignment for compound (±)-**39** was carried out by NOESY-2D experiments (see Figure S22f–g). NOESY correlations between, on one hand, H-3”β, H3–13”β, H-5”β and H-6”β, and on the other, between H3–14”a, H-6”a, H3–15”a, H2–11”a and H2–12’α, confirmed the structural assignment for (±)-**39** as 7-(3′*R*(*S*),5′*S*(*R*),8’*S*(*R*), 9’*R*(*S*),10’*R*(*S*)-8’,12’-epoxy-3′-hydroxydriman-11’-yloxy)-coumarin.

Compound (±)-**40** showed a molecular ion in its HRESIMS at *m*/*z* 365.2115 [M + H]^+^ (calcd. for C_24_H_29_O_3_ 365.2117), which is consistent with a molecular formula of C_24_H_28_O_5_ for this compound. Comparison with the molecular formula for (±)-**12** (C_24_H_30_O_4_) suggests that a molecule of water has been lost. Hydroxyl group elimination for compound (±)-**12** is further confirmed by the observation for (±)-**40** of additional olefin resonances in ^1^H NMR spectrum at δ_H_ 5.39 (H-3′) and 5.49 (H-2’) ppm and at δ_C_ 137.8 (CH, C-3′) and 120.8 ppm (CH, C-2’) in the ^13^C NMR. This confirmed structural assignment for (±)-**40** as 7-(5′*S*(*R*),9’*R*(*S*),10’*R*(*S*)-drima-3′,8’(12’)-dien-11’-yloxy)-coumarin.

### 2.2 Antifungal Biological Assays

The antifungal properties of compounds (±)-**12**-(±)-**14**, **21**–**25**, (±)-**26-**(±)-**33**, (±)-**35**–(±)-**40** were evaluated against the growth of *B. cinerea*, using the broth microdilution method.^53,54^ The commercial fungicides triclosan^55^ and azoxystrobin^56^ were used as standards for comparison. 6250 × 10^–5^ mg/mL, 391 × 10^–5^ mg/mL (16-fold dilution), and 49 × 10^–5^ mg/mL (128-fold dilution) doses were evaluated, which respectively correspond to 215.9 × 10^–6^ M–147.2 × 10^–6^ M, 13.5 × 10^–6^ M–9.2 × 10^–6^ M and 1.7 × 10^–6^ M–1.2 × 10^–6^ M dose ranges for evaluated compounds, including triclosan and azoxystrobin (see concentration details for every evaluated compound in Table S2). Different inhibition levels were observed (Figure 8, Table S3, and Figures S24–S26).

**Figure 8:**
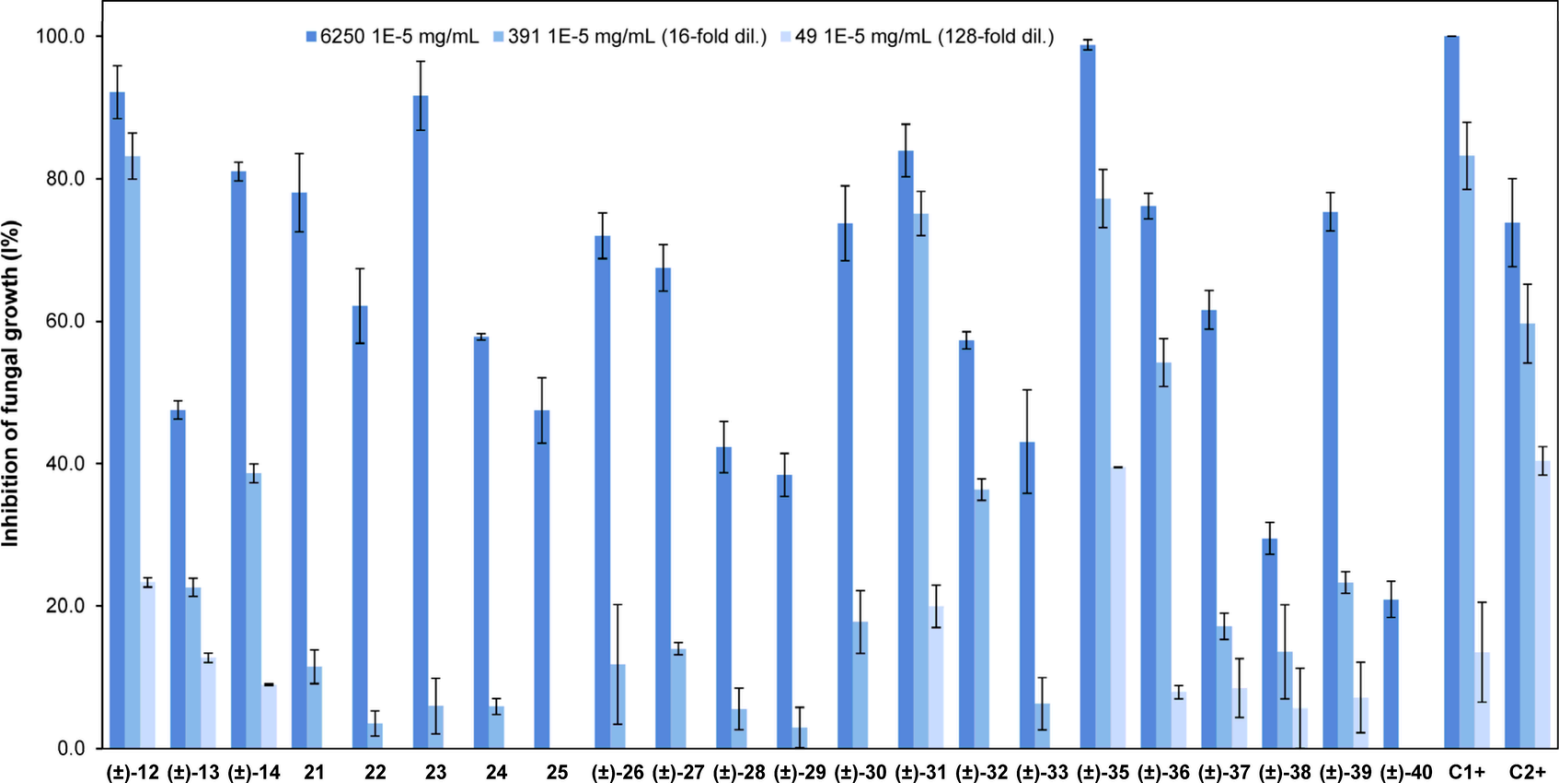
Comparison of inhibition of fungal growth percentage (IFG%) among compounds (±)-**12**-(±)-**14**, **21**–**25**, (±)-**26**–(±)-**33**, (±)-**35**–(±)-**40**, triclosan (C1^+^) and azoxystrobin (C2^+^) (*B. cinerea,* 6250 × 10^–5^ mg/mL, 391 × 10^–5^ mg/mL and 49 10–5 mg/mL doses; see molar doses for every compound in Table S2). Data are presented as mean ± standard deviation. See statistically significant differences between compounds, for every dose, in Tables S24–S26.

According to the *B. cinerea* inhibition percentages found at 391 × 10^–5^ mg/mL dose (Figure 8, Table S3 and Figure S25), most active compounds were exocyclic (±)-hydroxydrimenyloxyarene derivatives (±)-**12,** (±)-**31**, and (±)-**35**; which were more active than azoxystrobin (*p* < 0.05), while (±)-**12** presented an inhibition comparable to that of triclosan (83.2 ± 4.7). On the other hand, at the 49 × 10^–5^ mg/mL dose (Figure 8, Table S3 and Figure S26), where most compounds were inactive, (±)-**12**, (±)-**31**, and (±)-**35** were still more active than triclosan (*p* < 0.05), and (±)-**35** presented an inhibition percentage comparable to that of azoxystrobin (40.4 ± 2.0). Interestingly, compounds (±)-**12**, (±)-**31**, and (±)-**35** present similar polar fragments in the arene moiety, namely, a dihydro-2*H*-pyran-2-one fragment for (±)-**12** and (±)-**31** and ortho substituted hydroxyl and acetyl groups for (±)-**35** (Figures 6 and 9). On the other hand, significantly less active exocyclic (±)-hydroxydrimenyloxyarene derivatives (±)-**33** and (±)-**37**, even at the higher 6250 × 10^–5^ mg/mL dose (Figure 8, Tables S3 and S24), present, respectively, either acetyl or *O*-hydroxyphenyl fragments in the arene moiety.

**Figure 9:**
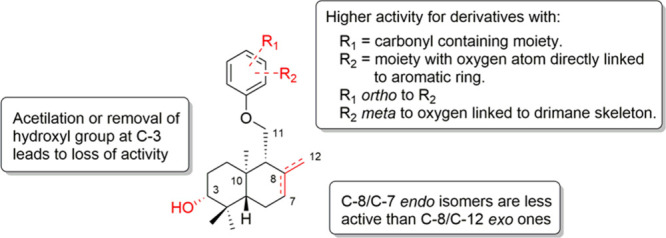
Structure–activity relationships for compounds (±)-**12–**(±)-**14**, (±)-**31**–(±)-**33**, (±)-**35**–(±)-**40**.

Other relevant structural features of compounds (±)-**12**, (±)-**31**, and (±)-**35** relate to their drimane moiety. As can be seen in the comparison of activities of these compounds with that of their synthetic precursors (farnesyloxy derivatives **21**, **22**, and **24** and epoxy derivatives (±)-**26**, (±)-**27**, and (±)-**29**, respectively; Figure 8, Table [Table tbl3_1H] and Figures S25–S26), the absence of a hydroxyl-drimenyloxy moiety render synthetic precursors **21**, **22**, **24**, (±)-**26**, (±)-**27**, and (±)-**29** inactive. Therefore, a rigidification strategy,^57,58^ through biomimetic cyclization of acyclic precursors has been successfully applied.

Regarding specific substituents on the drimane moiety, compounds (±)-**12**, (±)-**31**, and (±)-**35**, with and exocyclic C-8/C-12 double bond, are more active than their respective endocyclic C-7/C-8 double bond derivatives, compounds (±)-**14**, (±)-**32**, and (±)-**36** (see Figure 8 and Figures S24–S26, at all evaluated doses). Epoxidation of the exocyclic double bond in (±)-**12**, leading to (±)-**39**, also decreases activity (Figure 8 and Figures S24–S26, at all evaluated doses). Finally, the presence of a hydroxyl group at C-3 in the drimane moiety is a critical feature for activity, as acetylation decreases activity (see comparison of activities of compounds (±)-**12** and (±)-**13**, Figure 8 and Figures S24–26, at all evaluated doses); furthermore, elimination leads to an even greater degree of inactivation (see comparison of activities of compounds (±)-**12** and (±)-**40**, Figure 8 and Figures S24–26, at all evaluated doses). Structure–activity relationships are summarized in Figure 9.

Calculated physicochemical properties have been used to rationalize the structure–activity relationships of pharmaceuticals^59^ and agrochemicals.^60,61^ For instance, appropriate lipophilicity (evaluated as experimental or calculated logP) can facilitate transport through or even disruption of cell membrane.^62^ On the other hand, the topological polar surface area (sum of polar atom surfaces, TPSA)^63^ has been also correlated with transport through the membrane,^64^ either unaided^65^ or transporter mediated.^66^ Both parameters have been estimated for drimane skeleton compounds (±)-**12** to (±)-**14,** (±)-**31** to (±)-**33**, (±)-**35** to (±)-**40** using the Molinspiration software (https://www.molinspiration.com). Obtained values can be found in Table S4. Comparison of logP versus percentage of inhibition of fungal growth (IFG%) at 6250 10–5 mg/mL dose can be found in Figure S27; comparison of TPSA versus percentage of fungal growth inhibition (IFG%) at 6250 × 10^–5^ mg/mL dose can be found in Figure S28. In overall, antifungal activity correlates with logP, so lower values correspond to higher activities, as it has been described before for terpenoid derivatives.^29,67–69^ Higher activity compounds follow that correlation, such as (±)-**31** (logP = 5.68; IFG% = 84.0 ± 3.7), (±)-**12** (logP = 5.3; IFG% = 92.2 ± 3.7), and (±)-**35** (logP = 5.07; IFG% = 98.8 ± 0.7). Unfortunately, this correlation has exceptions, as hydroxydrymenyloxyphenylethanone (±)-**33**, with logP = 5.1 (IFG% = 43.1 ± 7.3), is less active than (±)-**35** and epoxide (±)-**39** (logP = 4.62, FGI% = 75.3 ± 2.7), with an even lower logP than (±)-**35**, it is also less active than the latter (Table S4, Figure S27). This fuzzy picture is compounded when TPSA values are also taken into consideration, as compounds with higher activity ((±)-**12**, (±)-**31**, and (±)-**35**), together with the relatively active C-7/C-8 endocyclic derivatives (±)-**14** and (±)-**36**, present TPSA values falling in the range between 59.67 and 66.76 Å^2^ (see Figure S28, Table S4).

Therefore, for this class of drimanyloxyarene derivatives evaluated as control agents of *B. cinerea*, and considering the present data, we propose that the inhibition of fungal growth increases as logP decreases, within an optimal range of 5.07 to 5.68. Simultaneously, active compounds should exhibit calculated TPSA values between 59.67 and 66.76 Å^2^. Within these limits, the percentage of inhibition of fungal growth (IFG%) for selected compounds ((±)-**12**, ((±)-**14**, (±)-**31**, (±)-**35**, and (±)-**36** ranges from 76% to 98%.

Interestingly, compounds falling within these limits possess four atoms capable of accepting hydrogen bond interactions and one hydroxyl group capable of donating hydrogen bond interactions. Removal of the hydroxyl group (as observed in compounds (±)-**13**, (±)-**39**, and (±)-**40**) leads to a decrease in activity. Similarly, a reduction of atoms capable of accepting hydrogen bond interactions (as observed in compounds (±)-**33**, (±)-**37** ,and (±)-**38**) also diminishes activity (see Figures S27–28, Table S4 and Figure 9).

In summary, a biomimetic synthesis of (±)-coladonin ((±)-**12**), (±)-feselol ((±)-**14**), and related derivatives was achieved, enabling the evaluation of their antifungal activity against *B. cinerea.* More active compounds, (±)-**12,** (±)-**31**, and (±)-**35**, showed antifungal activity comparable to commercial fungicides. The presence of a carbonyl group and an *ortho* substituted oxygen atom on the arene, along with an exocyclic (±)-hydroxydrimenyloxy fragment, is crucial for this activity, requiring at least four hydrogen bond acceptor atoms and one hydrogen bond donor group (Figure 9). The lipophilicity and TPSA values of the most active compounds align with known trends for antifungal compounds, suggesting the potential for further development, which would include in-depth mechanistic analysis of the mode of action and *in vivo* test data. So, future studies will focus on developing stable formulations for these compounds, as well as evaluating their phytotoxicity on relevant crops (such as strawberries and grapes) and their efficacy in controlled greenhouse trials and, eventually, in field trials. We are confident that this phase of research will confirm the potential of our hybrid molecules as a new generation of fungicides to combat the resistance of *Botrytis cinerea.*

## 3 Materials and Methods

### 3.1 General Experimental Procedures

Melting points were measured using a Reichert-Jung-Kofler block and are uncorrected. Optical rotations were determined on a Jasco P-2000 polarimeter. IR spectra were recorded on a PerkinElmer Spectrum Two FT-IR spectrophotometer. ^1^H and ^13^C NMR spectra were obtained on Varian INOVA 400, 500, and 600 MHz NMR spectrometers, with tetramethylsilane as an internal standard. NMR assignments were made using a combination of 1D and 2D techniques and by comparison with assignments for previously described compounds where applicable. High-resolution mass spectra were recorded on a Waters Synapt G2 QTOF spectrometer in ESI mode. Column chromatography was performed on silica gel (Merck, 60–200 mesh). Thin-layer chromatography (TLC) was performed on Merck Kiesegel 60 GF254 plates (0.2 mm thick). HPLC was performed with a VWR/Hitachi apparatus equipped with a Primaide 1110 pump, a Primaide 1410 UV–vis detector, and a Chromaster 5450 differential refractometer detector. Purification by HPLC was performed using a LiChroCART LiChrospher Si 60 (10 μm, 250 mm × 10 mm) or LiChroCART LiChrospher Si 60 (5 μm, 250 mm × 4 mm) column for normal-phase chromatography or a LiChroCART LiChrospher 100 (10 μm, 250 mm × 10 mm) column for reverse-phase chromatography.

### 3.2 Synthesis of Farnesyl Derivatives. General Procedure

Farnesyl bromide (3.5 mmol) and the corresponding phenol derivatives (**16**–**20**) (3.5 mmol) were dissolved in 5 mL of acetone. This solution was then treated with 3.12 mmol (0.432 g) of potassium carbonate (K_2_CO_3_) and stirred at 60 °C for 24 h.. Then, the reaction mixtures were diluted with ethyl acetate (EtOAc) (30 mL) and filtered through silica gel, and the solvent mixture was evaporated, yielding compounds **21** to **25** with high yields (90–99%) (Figure 4).

#### 3.2.1 7-(((2’*E*,6’*E*)-3,7,11-Trimethyldodeca-2,6,10-trien-1-yl)­oxy)-2*H*-chromen-2-one (**21**)

amorphous white solid (1280 mg, 3.49 mmol, 99%); IR ν_max_: 3405, 2953, 2402, 1750, 1600, 1567, 1444, 1350, 1203, 1013, 1001 cm^–1^; UV ν_max_: 270, 260 nm. ^1^H NMR: Table [Table tbl1_1H]; ^13^C NMR: Table [Table tbl1_13C]. HRESIMS *m*/*z* 367.2276 [M + H]^+^ (calcd. for C_24_H_31_O_3_ 367.2273).

#### 3.2.2 4-Methyl-7-(((2’*E*,6’*E*)-3,7,11-trimethyldodeca-2,6,10-trien-1-yl)­oxy)-2*H*-chromen-2-one (**22**)

amorphous white solid (1198 mg, 3.15 mmol, 90%); IR ν_max_: 3435, 2966, 2399, 1750, 1600, 1557, 1444, 1346, 1198, 1007, 1000 cm^–1^; UV ν_max_: 270, 260 nm. ^1^H NMR: Table[Table tbl2_1H]; ^13^C NMR: Table [Table tbl2_13C]. HRESIMS *m*/*z* 403.2252 [M + Na]^+^ (calcd. for C_25_H_32_O_3_Na 403.2249).

#### 3.2.3 1-(2’-(((2’’*E*,6’’*E*)-3,7,11-Trimethyldodeca-2,6,10-trien-1-yl)­oxy)­phenyl)­ethanone (**23**)

yellow oil (1191 mg, 3.5 mmol, 99%); IR ν_max_: 29226, 2855, 1736, 1670, 1596, 1482, 1357, 1300, 1270, 1162, 1124, 988, 756, cm^–1^; UV ν_max_: 260 nm. ^1^H NMR: Table [Table tbl3_1H]; ^13^C NMR: Table [Table tbl3_13C]. HRESIMS *m*/*z* 363.2300 [M + Na]^+^ (calcd. for C_23_H_32_O_2_Na 363.2300).

#### 3.2.4 1-(2’-Hydroxy-4’-(((2’’*E*,6’’*E*)-3,7,11-Trimethyldodeca-2,6,10-trien-1-yl)­oxy)­phenyl)­ethanone (**24**)

yellow oil (1150 mg, 3.22 mmol, 92%); IR ν_max_: 3630, 2962, 2922, 2855, 2361, 1633, 1505, 1461, 1269, 1191, 997, 800 cm^–1^; UV ν_max_: 280, 250 nm. ^1^H NMR: Table [Table tbl5_1H]; ^13^C NMR: Table [Table tbl5_13C]. HRESIMS *m*/*z* 357.2434 [M + H]^+^ (calc. for C_23_H_33_O_3_ 357.2430).

#### 3.2.5 2’-(((2’’*E*,6’’*E*)-3,7,11-Trimethyldodeca-2,6,10-trien-1-yl)­oxy)-[1,1’-biphenyl]-2-ol (**25**)

oil (1350 mg, 3.5 mmol, 99%); IR ν_max_: 3372, 2961, 2924, 1596, 1574, 1497, 1480, 1461, 1442, 1378, 1275, 1125, 984, 835, 752 cm^–1^; UV ν_max_: 260 nm. ^1^H NMR: Table [Table tbl4_1H]; ^13^C NMR: Table [Table tbl4_13C]. HRESIMS *m*/*z* 391.2623 [M + H]^+^ (calcd. for C_27_H_35_O_2_ 391.2637).

^1^H NMR, ^13^C NMR and HRESIMS and gHMBC spectra for compounds **21**–**25** are available in the Supporting Information as Figures S1a–S5a, S1b–S5b and S1c–S5c, S1e, S 2d and S3e–S5e, respectively, and gHSQC spectra for compounds **21**, **23**–**25** can be found as Figures S1d and S3d–S5d.

### 3.3 Synthesis of Epoxy-Farnesyl Derivatives. General Procedure

The synthesis of epoxy derivatives involved a two-step process starting with the corresponding bromohydrins via S_N_i reaction. First, farnesyl derivatives **21**–**25** were converted to their distal bromohydrins. Subsequently, treatment of these intermediate bromohydrin derivatives (without isolation) in a basic medium yielded epoxy derivatives (±)-**26**–(±)-**30**.

Each farnesyloxy arene (**21**–**25**) (1.75 mmol) was dissolved in 100 mL of THF/H_2_O (4:1, v/v), cooled at 0 °C, and treated with *N*-bromosuccinimide (1.75 mmol). After stirring for 30 min at 0 °C, each reaction mixture was extracted with EtOAc (50 mL, ×3). The combined organic phases were dried, filtered and the solvent evaporated, yielding a crude reaction mixture used directly in the next step without further purification.

Bromohydrin crudes were dissolved in a 0.5 M potassium carbonate solution in MeOH/H_2_O, (1:1, v/v, 30 mL) and stirred at room temperature for 30 min.. The resulting mixtures were extracted with EtOAc (50 mL × 3), the organic layers dried over anhydrous sodium sulfate, filtrated, and the solvent evaporated. The crude reaction mixtures were purified by silica gel column chromatography using increasing gradients of EtOAc in hexane, yielding compounds (±)-**26**–(±)-**30** with yields ranging from 29% to 53% (see below) (Figure 5).

#### 3.3.1 (±)-7-((2’*E*,6’*E*)-10,11-Epoxy-3,7,11-trimethyldodeca-2,6-dien-1-yloxy)-2*H*-chromen-2-one ((±)-**26**)

oil (270 mg, 0.71 mmol, 53%); IR ν_max_: 2959, 2923, 1731, 1750, 1613, 1556, 1508, 1445, 1388, 1143, 1069, 998, 875, 848 cm^–1^; UV ν_max_: 270, 260 nm. ^1^H NMR: Table [Table tbl1_1H]; ^13^C NMR: Table [Table tbl1_13C]. HRESIMS *m*/*z* 405.2026 [M + Na]^+^ (calcd. for C_24_H_30_O_4_Na 405.2042).

#### 3.3.2 (±)-7-((2’*E*,*6’E*)-10,11-Epoxy-3,7,11-trimethyldodeca-2,6-dien-1-yloxy)-4-methyl-2*H*-chromen-2-one ((±)-**27**)

amorphous solid (204 mg, 0.51 mmol, 48%); IR ν_max_: 3056, 2923, 2397, 1750, 1600, 1556, 1445, 1388, 1143, 1069, 1013, 848 cm^–1^; UV ν_max_: 270, 260 nm. ^1^H NMR: Table [Table tbl2_1H]; ^13^C NMR: see Table [Table tbl2_13C]. HRESIMS *m*/*z* 419.2193 [M + Na]^+^ (calcd. for C_25_H_32_O_4_Na 419.2198).

#### 3.3.3 (±)-1-(2’-((2’’*E*,6’’*E*)-10,11-Epoxy-3,7,11-trimethyldodeca-2,6-dien-1-yloxy)­phenyl)­ethanone ((±)-**28**)

oil (174 mg, 0.49 mmol, 28%); IR ν_max_: 2962, 2926, 2856, 1673, 1596, 1483, 1450, 1358, 1293, 1235, 1125, 989, 757, 593 cm^–1^; UV ν_max_: 260 nm. ^1^H NMR: Table [Table tbl3_1H]; ^13^C NMR: see Table [Table tbl3_13C]. HRESIMS *m*/*z* 379.2249 [M + Na]^+^ (calc. for C_23_H_32_O_3_Na 379.2249).

#### 3.3.4 (±)-1-(4’-((2’’*E*,6’’*E*)-10,11-Epoxy-3,7,11-trimethyldodeca-2,6-dien-1-yloxy)-2’-hydroxyphenyl) ethanone ((±)-**29**)

oil (245 mg, 0.66 mmol, 51%);. IR ν_max_: 2960, 2926, 2858, 1633, 1505, 1460, 1428, 1370, 1271, 1253, 1150, 1134, 1066, 998, 833, 803 cm^–1^; UV ν_max_: 260 nm. ^1^H NMR: Table [Table tbl5_1H]; ^13^C NMR: Table [Table tbl5_13C]. HRESIMS *m*/*z* 373.2384 [M + H]^+^ (calc. for C_23_H_33_O_4_ 373.2379).

##### 3.3.4.1 (±)-2’-((2’’*E*,6’’*E*)-10,11-Epoxy-3,7,11-trimethyldodeca-2,6-dien-1-yloxy)-[1,1’-biphenyl]-2-ol ((±)-**30**)

oil (305 mg, 0.75 mmol, 89%); IR ν_max_: 3359, 2961, 2924, 1666, 1609, 1596, 1574, 1497, 1461, 1579, 1442, 1378, 1225, 1125, 984, 835 cm^–1^; UV ν_max_: 260 nm. ^1^H NMR: Table [Table tbl4_1H]; ^13^C NMR: Table [Table tbl4_13C]. HRESIMS *m*/*z* 429.2407 [M + Na]^+^ (calc. for C_27_H_34_O_3_Na 429.2406).

### 3.4 Synthesis of Exo- and Endocyclic Drimanyl Derivatives

#### 3.4.1 General Procedure

A mixture of Cp_2_TiCl_2_ (127 mg, 0,51 mmol, 2,2 equiv) and manganese powder (10,2 mmol, 561 mg) in strictly dry and deoxygenated THF (100 mL), was stirred at room temperature until the red solution turned green (15 to 30 min approximately). This solution was then slowly added to the corresponding epoxide ((±)-**26**-(±)-**30**), 100 mg in 5 mL of deoxygenated THF) so that the solution maintained its green color. After stirring for 4 h. at room temperature, the reaction was quenched with HCl 2N (5 mL) and extracted with EtOAc (50 mL, x3). The organic phase was dried, filtered and solvent was evaporated. The crude product was purified by column chromatography yielding a mixture of endo- and exocyclic isomers, which were further purified by HPLC using normal phase chromatography (EtOAc/hexane 1:4, v/v) or reverse phase (linear gradient from AcN/H_2_O/MeOH 8:1:1 to AcN/H_2_O/MeOH 10:0:0, v/v/v) to obtain the target compounds (±)-**12**, (±)-**14**, (±)-**31** to (±)-**38** (Figure 6).

##### 3.4.1.1 (±)-7-(3′*R*(*S*),5′*S*(*R*),9’*R*(*S*),10’*R*(*S*)-3′-Hydroxydrim-8’(12’)-en-11’-yloxy)­coumarin ((±)-coladonin) ((±)-**12**)^37,38^

white needles, mp: 160 °C (27 mg, 0.070 mmol, 27%); IR ν_max_: 3446, 2927, 2871, 1710, 1620, 1504, 1258, 1174, 989 cm^–1^; UV ν_max_: 270, 260 nm. ^1^H NMR: Table [Table tbl1_1H]; ^13^C NMR: Table [Table tbl1_13C]. HRESIMS *m*/*z* 383.2219 [M + H]^+^ (calcd. for C_24_H_31_O_4_ 383.2222).

##### 3.4.1.2 (±)-7-(3′*R*(*S*),5′*S*(*R*),9’*R*(*S*),10’*R*(*S*)-3′-Hydroxydrim-7’-en-11’-yloxy)­coumarin ((+)-Feselol) ((±)-**14**)^39^

amorphous solid (12 mg, 0.031 mmol, 12%); IR ν_max_: 3446, 2930, 2869, 1710, 1620, 1504, 1258, 1176, 980 cm^–1^; UV ν_max_: 270, 260 nm. ^1^H NMR: Table [Table tbl1_1H]; ^13^C NMR: Table [Table tbl1_13C]. HRESIMS *m*/*z* 383.2222 [M + H]^+^ (calcd. for C_24_H_31_O_4_ 383.2222).

##### 3.4.1.3 (±)-7-(3′*R*(*S*),5′*S*(*R*),9’*R*(*S*),10’*R*(*S*)-3′-Hydroxydrim-8’(12’)-en-11’-yloxy)-4-methylcoumarin ((±)-**31**)

amorphous solid (36 mg, 0.091 mmol, 36%); IR ν_max_: 3476, 2940, 2870, 2446, 1714, 1600, 1511, 1470, 1389, 1146, 1071, 851, 754 cm^–1^; UV ν_max_: 270, 250 nm. ^1^H NMR: Table [Table tbl2_1H]; ^13^C NMR: Table [Table tbl2_13C]. HRESIMS *m*/*z* 397.2371 [M + H]^+^ (calcd. for C_25_H_33_O_4_ 397.2379).

##### 3.4.1.4 (±)-7-(3′*R*(*S*),5′*S*(*R*),9’*R*(*S*),10’*R*(*S*)-3′-Hydroxydrim-7’-en-11’-yloxy)-4-methylcoumarin ((±)-**32**)

amorphous solid (21 mg, 0.053 mmol, 21%); IR ν_max:_ 3456, 2929, 2852, 1713, 162, 1530, 1455, 1390, 1283, 1145, 1071, 1016, 850, 758 cm^–1^; UV ν_max_: 270, 250 nm. ^1^H NMR: Table [Table tbl2_1H]; ^13^C NMR: Table [Table tbl2_13C]. HRESIMS *m*/*z* 397.2382 [M + H]^+^ (calcd. for C_25_H_33_O_4_ 397.2379).

##### 3.4.1.5 (±)-1-(2’-(3*’’R*(*S*),5*’’S*(*R*),9*’’R*(*S*),10*’’R*(*S*)-3′’-Hydroxydrim-8’’(12’’)-en-11’’-yloxy)­phenyl) ethanone ((±)-**33**)

amorphous solid (30 mg, 0.084 mmol, 30%); IR ν_max:_ 3480, 2917, 2849, 1732, 1485, 1391, 1250, 1035, 906, 871, 759 cm^–1^; UV ν_max_: 275, 240 nm. ^1^H NMR: Table [Table tbl3_1H]; ^13^C NMR: Table [Table tbl3_13C]. HRESIMS *m*/*z* 357.2435 [M + H]^+^ (calcd. for C_23_H_33_O_3_ 357.2430).

##### 3.4.1.6 (±)-1-(2’-(*3′’R*(*S*),5′’*S*(*R*),9’’*R*(*S*),10’’*R*(*S*)-3′’-Hydroxydrim-7’’-en-11’’-yloxy)­phenyl)­ethanone ((±)-**34**)

amorphous solid (11 mg, 0.031 mmol, 11%); IR ν_max:_ 3476, 2967, 2940, 2868, 1640, 1622, 1505, 1472, 1371, 1256, 1135, 1032, 804, 754 cm^–1^; UV ν_max_: 275, 240 nm. ^1^H NMR: Table [Table tbl3_1H]; ^13^C NMR: Table [Table tbl3_13C]. HRESIMS *m*/*z* 357.2420 [M + H]^+^ (calc. for C_23_H_33_O_3_ 357.2430).

##### 3.4.1.7 (±)-1-(2’-Hydroxy-4’-(*3′’R*(*S*),5′’*S*(*R*),9’’*R*(*S*),10’’*R*(*S*)-3′’-hydroxydrim-8’’(12’’)-en-11’’-yloxy)­phenyl)­ethanone ((±)-**35**)

amorphous solid (38 mg, 0.102 mmol, 38%); IR ν_max_: 3497, 2916, 2849, 1738, 1633, 1485, 1445, 1385, 1249, 1215, 1034, 909, 763 cm^–1^; UV ν_max_: 260 nm. ^1^H NMR: Table [Table tbl5_1H]; ^13^C NMR: Table [Table tbl5_13C]. HRESIMS *m*/*z* 373.2374 [M + H]^+^ (calcd. for C_23_H_33_O_4_ 373.2379).

##### 3.4.1.8 (±)-1-(2’-Hydroxy-4’-(3′’*R*(*S*),5′’*S*(*R*),9’’*R*(*S*),10’’*R*(*S*)-3′’-hydroxydrim-7’’-en-11’’-yloxy)­phenyl)­ethanone ((±)-**36**)

amorphous solid (22 mg, 0.059 mmol, 22%); IR ν_max_: 3476, 2967, 2940, 2868, 1640, 1622, 1505, 1472, 1371, 1256, 1135, 1032, 804, 754 cm^–1^; UV ν_max_: 260 nm. ^1^H NMR: Table [Table tbl5_1H]; ^13^C NMR: Table [Table tbl5_13C]. HRESIMS *m*/*z* 373.2382 [M + H]^+^ (calcd. for C_23_H_33_O_4_ 373.2379).

##### 3.4.1.9 (±)-2’-(3′’*R*(*S*),5′’*S*(*R*),9’’*R*(*S*),10’’*R*(*S*)-3′’-Hydroxydrim-8’’(12’’)-en-11’’-yloxy)-[1,1’-biphenyl]-2-ol ((±)-**37**)

amorphous solid (32 mg, 0.079 mmol, 32%); IR ν_max_: 3395, 2965, 2939, 2871, 1716, 1696, 1651, 1596, 1455, 1442, 1263, 1226, 1029, 1009, 753 cm^–1^; UV ν_max_: 260 nm. ^1^H NMR: Table [Table tbl4_1H]; ^13^C NMR Table [Table tbl4_13C]. HRESIMS *m*/*z* 429.2396 [M + Na]^+^ (calcd. for C_27_H_34_O_3_Na 429.2406).

##### 3.4.1.10 (±)-2’-(3′’*R*(*S*),5′’*S*(*R*),8’’*R*(*S*),9’’*R*(*S*),10*’’R*(*S*)-3′’-Hydroxydriman-11’’-yloxy)-[1,1’-biphenyl]-2-ol ((±)-**38**)

amorphous solid (12 mg, 0.029 mmol, 12%); IR ν_max_: 3366, 2937, 2873, 1713, 1596, 1580, 1480, 1443, 1385, 1272, 1226, 1022, 847 cm^–1^; UV ν_max_: 260 nm. ^1^H NMR: Table [Table tbl4_1H]; ^13^C NMR: Table [Table tbl4_13C]. HRESIMS *m*/*z* 409.2739 [M + H]^+^ (calcd. for C_27_H_37_O_3_ 409.2743).

^1^H NMR, ^13^C NMR, and HRESIMS spectra can be found in Supporting Information for compounds (±)-**12** (Figure S11a–c), (±)-**14** (Figure S13a–c), (±)-**31**–(±)-**38** (Figures S14a–c, S15a–c, S16a–c, S17a–c, S18a–c, S19a–c, S20a–c. and S21a–c). gHSQC and gHMBC spectra can be found in Supporting Information for compounds (±)-**12** (Figure S11d–e), (±)-**14** (Figure S13d–e), (±)-**31**–(±)-**38** (Figure S14d–e, S15d-e, S16d-e, S17d-e, S18d-e, S19d-e, S20d-e and S21d-e). NOESY2D spectra can be found in Supporting Information for compounds (±)-**12** (Figure S11f–g), (±)-**14** (Figure S13f–g), (±)-**31**–(±)-**36** (Figures S14f, S15f, S16f, S17f, S18f, S19f) and (±)-**38** (Figure S21f–g).

### 3.5 Chemical Transformations of (±)-Coladonin ((±)-**12**)

#### 3.5.1 Acetyl Derivative, (±)-Coladin ((±)-**13**)

Compound (±)-**12** (35 mg) was dissolved in 15 μL of pyridine and 0.5 mL of acetic anhydride. The solution was stirred at room temperature for 12 h. The reaction mixture was diluted with 10 mL of toluene, and the solvent mixture was evaporated. Crude reaction mixture was dissolved in EtOAc, filtered through silica gel, and solvent evaporated under reduced pressure. The reaction crude was purified by HPLC chromatography (EtOAc/hexane, 1:4) to yield (±)-**13** (36%) (Figure 7).

##### 3.5.1.1 7-(3′*R*(*S*),5′*S*(*R*),9’*R*(*S*),10’*R*(*S*)-3′-Acetoxydrim-8’(12’)-en-11’-yloxy)­coumarin (Acetyl Coladonin, Coladin, (±)-**13**)

Amorphous solid (14 mg, 0.033 mmol, 36%); IR ν_max_: 2966, 2945, 2872, 1731, 1613, 1508, 1396, 1350, 1246, 1123, 1029, 835, 756 cm^–1^; UV ν_max_: 270, 260 nm. ^1^H NMR: Table [Table tbl6_1H]; ^13^C NMR: Table [Table tbl6_13C]. HRESIMS *m*/*z* 447.2149 [M + H]^+^ (calcd. for C_26_H_32_O_5_Na 447.2147).

**11 tbl6_1H:** ^1^H NMR Spectroscopic Data (±)-**13**, (±)-**39**, and (±)-**40**

	(±)-**13**	(±)-**39**	(±)-**40**
H	δ_H_ [Table-fn t6_1Hfn1] mult. (J (Hz))	δ_H_ [Table-fn t6_1Hfn1] mult. (J (Hz))	δ_H_ [Table-fn t6_1Hfn2] mult. (J (Hz))
3	6.24, d (9.5)	6.24, d (9.5)	6.23, d (9.5)
4	7.63, d (9.5)	7.62, d (9.5)	7.61, d (9.5)
5	7.35, d (8.7)	7.34, d (8.2)	7.34, d (9.5)
6	6.83, m	6.81, mc	6.83, m
8	6.81, m	6.80, m	6.82, m
1’	a: 1.80, ddd, (11.7, 7.9, 2.9)	a: 1.69, m	a, b: 2.09, m
	b: 1.46, m	b: 1.46, m	
2’	a: 1.75, m	a, b: 1.69m	5.49 ddd (10.1, 4.9, 3.1)
	b: 1.50, m		
3′	β: 4.55, dd (11.8, 4.3)	β: 3.30 dd (11.4, 4.2)	5.39 d (10.1)
5′	β: 1.27, dd (12.5, 2.5)	β: 1.13, dd (12.4, 2.7)	β: 1.46, dd (12.0, 3.7)
6’	a: 1.76, m	α: 1.61, m	a: 1.78, m
	b: 1.46, m	β: 1.86, m	b: 1.49, m
7’	α: 2.46 ddd (13.1, 4.2, 2.3)	α: 1.46, m	a: 2.47, ddd (13.0, 4.0, 2.6)
	β: 2.11 td (13.1, 4.7)	β: 1.98, m	b: 2.06, m
9’	β: 2.22, m	β: 1.98, m	β: 2.28, m
11’	a, b: 4.20	a: 3.85, dd (10.1, 6.0)b: 3.82, dd (10.1, 2.2)	a, b: 4.21
12’	a: 4.92, s	a, b: 2.65	a: 4.91, s (br)
	b: 4.54, s		b: 4.53, s (br)
13’	β: 0.90, s	β: 1.04, s	β: 0.98, s
14’	α: 0.89, s	α: 0.85, s	α: 0.87, s
15’	α: 0.87, s	α: 1.00, s	α: 0.83, s
CH_3_CO	2.06, s	-	-

a CDCl_3_ (500 MHz).

b CDCl_3_ (400 MHz).

**12 tbl6_13C:** ^13^C NMR Spectroscopic Data for (±)-**13**, (±)-**39**, and (±)-**40**

	(±)-**13**	(±)-**39**	(±)-**40**
C	δ_C_ [Table-fn t6_13Cfn1], mult.	δ_C_ [Table-fn t6_13Cfn1], mult.	δ_C_ [Table-fn t6_13Cfn1], mult.
2	161.2, C	161.2, C	161.2, C
3	113.0, CH	112.8, CH	113.0, CH
4	143.4, CH	143.3, CH	143.4, CH
5	128.7, CH	128.7, CH	128.7, CH
6	113.1, CH	112.5, CH	113.1, CH
7	162.1, C	161.7, C	162.3, C
8	101.3, CH	101.7, CH	101.4, CH
9	155.9, C	155.9, C	155.9, C
10	112.5, C	113.1, C	112.5, C
1’	39.9, CH_2_	36.8, CH_2_	39.0, CH_2_
2’	24.1, CH_2_	27.1, CH_2_	120.8, CH
3′	80.3, CH	78.5, CH	137.8 CH
4’	38.1, C	39.0, C	34.8, C
5′	54.4, CH	53.9, CH	51.2, CH
6’	23.3, CH_2_	21.3, CH_2_	25.1, CH_2_
7’	37.3, CH_2_	35.6, CH_2_	37.5, CH_2_
8’	146.1, C	57.5, C	146.5, C
9’	54.7, CH	52.7, CH	53.7, CH
10’	38.7, C	39.3, C	38.2, C
11’	65.7, CH_2_	62.7, CH_2_	65.7, CH_2_
12’	107.9, CH_2_	51.9, CH_2_	107.2, CH_2_
13’	28.3, CH_3_	28.3, CH_3_	31.9, CH_3_
14’	15.4, CH_3_	15.4, CH_3_	23.4, CH_3_
15’	14.0, CH_3_	15.4, CH_3_	14.9, CH_3_
CH_3_CO	21.3, CH_3_	-	-
CH_3_ CO	170.9, C	-	-

a CDCl_3_ (125 MHz).

#### 3.5.2 Synthesis of Epoxy-drimanyl Derivative (±)-**39**

Compound (±)-**12** (86 mg, 0.26 mmol) was dissolved in 5 mL of dichloromethane and treated with 67 mg of *m*-chloroperbenzoic acid. The reaction mixture was stirred for 12 h and then filtered through silica gel. Solvent was evaporated to yield a reaction crude which was purified by HPLC (EtOAc/hexane, 1:4) to obtain 47 mg of (±)-**39** (75%) (Figure 7).

##### 3.5.2.1 7-(3′*R*(*S*),5′*S*(*R*),8’ *S*(*R*),9’ *R*(*S*),10’ *R*(*S*))-8’,12’-Epoxy-3′-hydroxydriman-11’-yloxy)-coumarin ((±)-39)

Amorphous solid (47 mg, 0.118 mmol, 75%); IR ν_max_: 3430, 2962, 2939, 2869, 1729, 1612, 1555, 1508, 1400, 1282, 1230, 1125, 835, 753 cm^–1^; UV ν_max_: 270, 260 nm. ^1^H NMR: Table [Table tbl6_1H]; ^13^C NMR: Table [Table tbl6_13C]. HRESIMS *m*/*z* 399.2178 [M + H]^+^ (calcd. for C_24_H_31_O_5_ 399.2171).

#### 3.5.3 Synthesis of 3,4-Dehydrocoladonin ((±)-**40**)

Diisopropylazodicarboxylate (DIAD) (120 mg, 0.1 mL) was added dropwise to a solution of (±)-coladonin ((±)-**12**) (57 mg) and PPh_3_ (60 mg) in refluxed dry toluene (15 mL). After 6 h, the reaction mixture was allowed to cool to 25 °C. Methanol (25 mL) was added, and the mixture of solvents was evaporated under reduced pressure. The resulting crude reaction mixture was diluted with EtOAc and filtered through silica gel. Solvent was evaporated, and the partially purified reaction mixture was finally purified by normal phase HPLC (EtOAc/hexane, 1:4) to obtain product (±)-**40** (24 mg, 44%) (Figure 7).

##### 3.5.3.1 7-(5′*R*(*S*),9’ *R*(*S*),10’ *R*(*S*))-Drima-3′,8’(12’)-dien-11’-yloxy)-coumarin ((±)-40)

amorphous solid, (24 mg, 0.065 mmol, 44%); IR ν_max_: 2960, 2945, 2932, 2364, 1734, 1612, 1509, 1403, 1350, 1279, 1230, 1121, 834 cm^–1^; UV ν_max_: 270, 260 nm. ^1^H NMR: Table [Table tbl6_1H]; ^13^C NMR: Table [Table tbl6_13C]. HRESIMS *m/*z 365.2115 [M + H]^+^ (calcd. for C_24_H_29_O_3_ 365.2117).

### 3.6 General Procedure for Antifungal Assay

*In vitro* antifungal assays were carried out against the strain *Botrytis cinerea* UCA992, which was isolated from grapes at a Domecq vineyard, Jerez de la Frontera, Cádiz, Spain. This culture is deposited in the Universidad de Cadiz, Mycological Herbarium Collection (UCA). Broth microdilution assays for the measurement of inhibition of *B. cinerea* growth were carried out in 96-well microplates (Thermo Fischer Scientific, NunclonTM Δ, flat bottom, with lid, sterile) according to previous reports.^53,54^ Wells for the evaluation of each compound tested (ECWs) were prepared from stock solutions of each compound in DMSO and diluted in Sabouraud-glucose liquid medium (casein peptone, 5 g/L; meat peptone, 5 g/L; dextrose, 20 g/L; pH (25 °C) 5.4–5.8) to a volume of 100 μL. Then, an inoculum suspension of the fungus (100 μL, 5 × 104 spores) was added to each well (final volume in each well = 200 μL), so final treatment concentrations were 6250 × 10^–5^ mg/mL, 391 × 10^–5^ mg/mL (16-fold dilution), and 49 × 10^–5^ mg/mL (128-fold dilution). Maximum final concentration of DMSO in each treatment well was kept below 2%.

For each evaluated compound, fungal growth control wells (FCWs) were prepared, containing Sabourad-glucose liquid medium, inoculum, and the same amount of DMSO used in ECWs, but devoid of tested compounds. Medium control wells (MCWs) were also prepared, containing only Sabouraud-glucose liquid medium and the amount of DMSO used in ECWs, but without tested compounds. Finally, for each evaluated compound, blank control wells (BCWs) were also prepared, which included compound, Sabouraud-glucose liquid medium, and sterile water, instead of inoculum, to a final volume of 200 μL, with a maximum concentration of DMSO < 2%.

The 96-well microplates were incubated at 25 °C for 72 h. Fungal growth was evaluated by measuring absorbance of each well at 492 nm in a microplate reader (Thermo Fischer Scientific Multiskan FC, vers. 1.00.94).^70^ Triclosan (5-chloro-2-(2,4-dichlorophenoxy)­phenol; CAS number 3380-34-5)^55^ and azoxystrobin (methyl (2*E*)-2-(2-{[6-(2-cyanophenoxy)­pyrimidin-4-yl]­oxy}­phenyl)-3-methoxyprop-2-enoate, CAS number 131860-33-8)^56^ were used as a positive control. Tests were performed in triplicate. Growth inhibition for each compound concentration was calculated as Inhibition of Fungal Growth percentage (IFG%) = 100 – (((ABS492ECW–ABS492BCW) × 100)/(ABS492FCW–ABS492MCW)), where ABS492 is the measured absorbance for every well type defined above (ECWs, FCWs, MCWs and BCWs).

### 3.7 Statistical Analysis

Antifungal effects (inhibition of fungal growth percentage, IFG%) are expressed as the mean + standard deviation (SD). Analysis of variance for comparison of antifungal effects between evaluated compounds and control was achieved by one-way ANOVA. Comparison between treatment means was done by Tukey HSD test. Significance was set at *p* < 0.05. Analysis was carried out using Statgraphics (Centurion 19).

## Supplementary Material



## Data Availability

The data supporting this study are available in the published article and its Supporting Information.

## Notes

^1^H NMR, ^13^C NMR, HRESIMS and gHMBC spectra for compounds (±)-**12**–(±)-**14**, **21**–**25**, (±)-**26**–(±)-**40**; HSQC spectra for compounds (±)-**12**–(±)-**14**, **21**, **23**–**25**, (±)-**26**–(±)-**40**; NOESY2D spectra for compounds (±)-**12**, (±)-**14**, (±)-**31**–(±)-**36**, (±)-**38**, and (±)-**39**. Tables S1-^1^H and S1-^13^C. Tables S2–S4. Figures S24–S28 (PDF)

## Abbreviations

IFG%: inhibition of fungal growth percentage
ABS492: measured absorbance at 492 nm
ECW: evaluation of compound well
FCW: fungal growth control well
HMBC: heteronuclear multiple bond connectivity
HRESIMS: high resolution electrospray ionization mass spectrometry
HSQC: heteronuclear single quantum coherence
MCW: medium control well
NMR: nuclear magnetic resonance
NOESY2D: Bidimensional Nuclear Overhauser Effect SpectroscopY
TPSA: total polar surface area
