# Supplementary material for: Synthesis of Farnesyloxy- and Drimanyloxy-Arene Scaffold-Based Hybrid Molecules as Antifungals against Botrytis cinerea
Source: J Agric Food Chem. 2025 Oct 27;73(44):28005–20. doi: 10.1021/acs.jafc.5c06584 (PMC12593352; doi:10.1021/acs.jafc.5c06584)
Supplement: Supplementary file 1 [file jf5c06584_si_001.pdf]

Synthesis of Farnesyloxy- and Drimanyloxy-Arene Scaffold Based Hybrid Molecules as Antifungals against *Botrytis cinerea*.

Antonio Ruano-González,[1] Ana A. Pinto,[1] Gabriela Mancilla,[1] Rosario Sánchez-Maestre,[1] Josefina Aleu,[1,2] Rosario Hernández-Galán,[1,2] Antonio J. Macías-Sánchez[1,2,*] and Isidro G. Collado,[1,2,*]

[1] *Departamento de Química Orgánica, Facultad de Ciencias, Campus Universitario Puerto Real, Universidad de Cádiz, 11510 Puerto Real, Cádiz, Spain.*

[2] *Instituto de Investigación en Biomoléculas (INBIO), Universidad de Cádiz, 11510 Puerto Real, Cádiz, Spain.*

Corresponding Authors

Antonio J. Macías-Sánchez. *Departamento de Química Orgánica, Facultad de Ciencias, Campus Universitario Puerto Real, Universidad de Cádiz, 11510 Puerto Real, Cádiz, Spain; Instituto de Investigación en Biomoléculas (INBIO), Universidad de Cádiz, 11510 Puerto Real, Cádiz, Spain*; https://orcid.org/ 0000-0001-6002-4977; Tel: +34956012704; E-mail: antoniojose.macias@uca.es

Isidro G. Collado. *Departamento de Química Orgánica, Facultad de Ciencias, Campus Universitario Puerto Real, Universidad de Cádiz, 11510 Puerto Real, Cádiz, Spain; Instituto de Investigación en Biomoléculas (INBIO), Universidad de Cádiz, 11510 Puerto Real, Cádiz, Spain*; https://orcid.org/0000-0002-8612-059; Tel: +34956012768; E-mail: isidro.gonzalez@uca.es

Supplementary Materials

Table of Contents

Figure S1a. ^{1}H NMR spectrum (400 MHz) of 7-(((2′*E*,6′*E*)-3,7,11-trimethyldodeca-2,6,10-trien-1-yl)oxy)-2*H*-chromen-2-one (umbelliprenin) (**21**) in $CDCl_3$....................**9**

Figure S1b. ^{13}C NMR spectrum (100 MHz) of 7-(((2′*E*,6′*E*)-3,7,11-trimethyldodeca-2,6,10-trien-1-yl)oxy)-2*H*-chromen-2-one (umbelliprenin) (**21**) in $CDCl_3$....................**10**

Figure S1c. HRESIMS of 7-(((2′*E*,6′*E*)-3,7,11-trimethyldodeca-2,6,10-trien-1-yl)oxy)-2*H*-chromen-2-one (umbelliprenin) (**21**)....................**11**

Figure S1d. gHSQC spectrum of 7-(((2′*E*,6′*E*)-3,7,11-trimethyldodeca-2,6,10-trien-1-yl)oxy)-2*H*-chromen-2-one (umbelliprenin) (**21**) in $CDCl_3$....................**12**

Figure S1e. gHMBC spectrum of 7-(((2′*E*,6′*E*)-3,7,11-trimethyldodeca-2,6,10-trien-1-yl)oxy)-2*H*-chromen-2-one (umbelliprenin) (**21**) in $CDCl_3$....................**13**

Figure S2a. ^{1}H NMR spectrum (400 MHz) of 4-methyl-7-(((2′*E*,6′*E*)-3,7,11-trimethyldodeca-2,6,10-trien-1-yl)oxy)-2*H*-chromen-2-one (7-farnesyloxy-4-methylumbelliferone) (**22**) in $CDCl_3$....................**14**

Figure S2b. ^{13}C NMR spectrum (100 MHz) of 4-methyl-7-(((2′*E*,6′*E*)-3,7,11-trimethyldodeca-2,6,10-trien-1-yl)oxy)-2*H*-chromen-2-one (7-farnesyloxy-4-methylumbelliferone) (**22**) in $CDCl_3$....................**15**

Figure S2c. HRESIMS of 4-methyl-7-(((2′*E*,6′*E*)-3,7,11-trimethyldodeca-2,6,10-trien-1-yl)oxy)-2*H*-chromen-2-one (7-farnesyloxy-4-methylumbelliferone) (**22**)....................**16**

Figure S2d. gHMBC spectrum of 4-methyl-7-(((2′*E*,6′*E*)-3,7,11-trimethyldodeca-2,6,10-trien-1-yl)oxy)-2*H*-chromen-2-one (7-farnesyloxy-4-methylumbelliferone) (**22**) in $CDCl_3$. **17**

Figure S3a. ^{1}H NMR spectrum (400 MHz) of 1-(2′-(((2′′*E*,6′′*E*)-3,7,11-trimethyldodeca-2,6,10-trien-1-yl)oxy)phenyl)ethanone (**23**) in $CDCl_3$. **18**

Figure S3b. ^{13}C NMR spectrum (100 MHz) of 1-(2′-(((2′′*E*,6′′*E*)-3,7,11-trimethyldodeca-2,6,10-trien-1-yl)oxy)phenyl)ethanone (**23**) in $CDCl_3$. **19**

Figure S3c. HRESIMS of 1-(2′-(((2′′*E*,6′′*E*)-3,7,11-trimethyldodeca-2,6,10-trien-1-yl)oxy)phenyl)ethanone (**23**).....................**20**

Figure S3d. gHSQC spectrum of 1-(2′-(((2′′*E*,6′′*E*)-3,7,11-trimethyldodeca-2,6,10-trien-1-yl)oxy)phenyl)ethanone (**23**) in $CDCl_3$.....................**21**

Figure S3e. gHMBC spectrum of 1-(2′-(((2′′*E*,6′′*E*)-3,7,11-trimethyldodeca-2,6,10-trien-1-yl)oxy)phenyl)ethanone (**23**) in $CDCl_3$....................**22**

Figure S4a. ^{1}H NMR spectrum (400 MHz) of 1-(2′-hydroxy-4′-(((2′′*E*,6′′*E*)-3,7,11-trimethyldodeca-2,6,10-trien-1-yl)oxy)phenyl)ethanone (**24**) in $CDCl_3$....................**23**

Figure S4b. ^{13}C NMR spectrum (100 MHz) of 1-(2′-hydroxy-4′-(((2′′*E*,6′′*E*)-3,7,11-trimethyldodeca-2,6,10-trien-1-yl)oxy)phenyl)ethanone (**24**) in $CDCl_3$....................**24**

Figure S4c. HRESIMS of 1-(2′-hydroxy-4′-(((2′′*E*,6′′*E*)-3,7,11-trimethyldodeca-2,6,10-trien-1-yl)oxy)phenyl)ethanone (**24**) **25**

Figure S4d. gHSQC spectrum of 1-(2′-hydroxy-4′-(((2′′*E*,6′′*E*)-3,7,11-trimethyldodeca-2,6,10-trien-1-yl)oxy)phenyl)ethanone (**24**) in $CDCl_3$....................**26**

Figure S4e. gHMBC spectrum of 1-(2′-hydroxy-4′-(((2′′*E*,6′′*E*)-3,7,11-trimethyldodeca-2,6,10-trien-1-yl)oxy)phenyl)ethanone (**24**) in $CDCl_3$....................**27**

Figure S5a. ^{1}H NMR spectrum (400 MHz) of 2'-(((2′′*E*,6′′*E*)-3,7,11-trimethyldodeca-2,6,10-trien-1-yl)oxy)-[1,1'-biphenyl]-2-ol (**25**) in $CDCl_3$. **28**

Figure S5b. ^{13}C NMR spectrum (100 MHz) of 2'-(((2''*E*,6''*E*)-3,7,11-trimethyldodeca-2,6,10-trien-1-yl)oxy)-[1,1'-biphenyl]-2-ol (**25**) in $CDCl_3$..........**29**

Figure S5c. HRESIMS of 2'-(((2''*E*,6''*E*)-3,7,11-trimethyldodeca-2,6,10-trien-1-yl)oxy)-[1,1'-biphenyl]-2-ol (**25**)..........**30**

Figure S5d. gHSQC spectrum of 2'-(((2''*E*,6''*E*)-3,7,11-trimethyldodeca-2,6,10-trien-1-yl)oxy)-[1,1'-biphenyl]-2-ol (**25**) in $CDCl_3$..........**31**

Figure S5e. gHMBC spectrum of 2'-(((2''*E*,6''*E*)-3,7,11-trimethyldodeca-2,6,10-trien-1-yl)oxy)-[1,1'-biphenyl]-2-ol (**25**) in $CDCl_3$..........**32**

Figure S6a. ^{1}H NMR spectrum (400 MHz) of (±)-7-((2′*E*,6′*E*)-10,11-epoxy-3,7,11-trimethyldodeca-2,6-dien-1-yloxy)-2H-chromen-2-one ((±)-**26**) in $CDCl_3$..........**33**

Figure S6b. ^{1}H NMR spectrum (100 MHz) of 7-((2′*E*,6′*E*)-10,11-epoxy-3,7,11-trimethyldodeca-2,6-dien-1-yloxy)-2H-chromen-2-one ((±)-**26**) in $CDCl_3$..........**34**

Figure S6c. HRESIMS of (±)-7-((2′*E*,6′*E*)-10,11-epoxy-3,7,11-trimethyldodeca-2,6-dien-1-yloxy)-2H-chromen-2-one ((±)-**26**)..........**35**

Figure S6d. gHSQC spectrum of (±)-7-((2′*E*,6′*E*)-10,11-epoxy-3,7,11-trimethyldodeca-2,6-dien-1-yloxy)-2H-chromen-2-one ((±)-**26**) in $CDCl_3$..........**36**

Figure S6e. gHMBC spectrum of (±)-7-((2′*E*,6′*E*)-10,11-epoxy-3,7,11-trimethyldodeca-2,6-dien-1-yloxy)-2H-chromen-2-one ((±)-**26**) in $CDCl_3$. **37**

Figure S7a. ^{1}H NMR spectrum (500 MHz) of (±)-7-((2′*E*,6′*E*)-10,11-epoxy-3,7,11-trimethyldodeca-2,6-dien-1-yloxy)-4-methyl-2H-chromen-2-one ((±)-**27**) in $CDCl_3$..........**38**

Figure S7b. ^{13}C NMR spectrum (100 MHz) of (±)-7-((2′*E*,6′*E*)-10,11-epoxy-3,7,11-trimethyldodeca-2,6-dien-1-yloxy)-4-methyl-2H-chromen-2-one ((±)-**27**) in $CDCl_3$..........**39**

Figure S7c. HRESIMS of (±)-7-((2′*E*,6′*E*)-10,11-epoxy-3,7,11-trimethyldodeca-2,6-dien-1-yloxy)-4-methyl-2H-chromen-2-one ((±)-**27**) in $CDCl_3$..........**40**

Figure S7d. gHSQC spectrum of (±)-7-((2′*E*,6′*E*)-10,11-epoxy-3,7,11-trimethyldodeca-2,6-dien-1-yloxy)-4-methyl-2H-chromen-2-one ((±)-**27**) in $CDCl_3$..........**41**

Figure S7e. gHMBC spectrum of (±)-7-((2′*E*,6′*E*)-10,11-epoxy-3,7,11-trimethyldodeca-2,6-dien-1-yloxy)-4-methyl-2H-chromen-2-one ((±)-**27**) in $CDCl_3$..........**42**

Figure S8a. ^{1}H NMR spectrum (400 MHz) of (±)-1-(2′-((2''*E*,6''*E*)-10,11-epoxy-3,7,11-trimethyldodeca-2,6-dien-1-yloxy)phenyl)ethanone ((±)-**28**) in $CDCl_3$..........**43**

Figure S8b. ^{13}C NMR spectrum (100 MHz) of (±)-1-(2′-((2''*E*,6''*E*)-10,11-epoxy-3,7,11-trimethyldodeca-2,6-dien-1-yloxy)phenyl)ethanone ((±)-**28**) in $CDCl_3$..........**44**

Figure S8c. HRESIMS of (±)-1-(2′-((2''*E*,6''*E*)-10,11-epoxy-3,7,11-trimethyldodeca-2,6-dien-1-yloxy)phenyl)ethanone ((±)-**28**). **45**

Figure S8d. gHSQC spectrum of (±)-1-(2′-((2''*E*,6''*E*)-10,11-epoxy-3,7,11-trimethyldodeca-2,6-dien-1-yloxy)phenyl)ethanone ((±)-**28**) in $CDCl_3$..........**46**

Figure S8e. gHMBC spectrum of (±)-1-(2′-((2''*E*,6''*E*)-10,11-epoxy-3,7,11-trimethyldodeca-2,6-dien-1-yloxy)phenyl)ethanone ((±)-**28**) in $CDCl_3$..........**47**

Figure S9a. ^{1}H NMR spectrum (400 MHz) of (±)-1-(4′-((2''*E*,6''*E*)-10,11-epoxy-3,7,11-trimethyldodeca-2,6-dien-1-yloxy)-2′-hydroxyphenyl)ethanone ((±)-**29**) in $CDCl_3$..........**48**

Figure S9b. ^{13}C NMR spectrum (100 MHz) of (±)-1-(4′-((2''*E*,6''*E*)-10,11-epoxy-3,7,11-trimethyldodeca-2,6-dien-1-yloxy)-2′-hydroxyphenyl)ethanone ((±)-**29**) in $CDCl_3$..........**49**

Figure S9c. HRESIMS of (±)-1-(4′-((2''*E*,6''*E*)-10,11-epoxy-3,7,11-trimethyldodeca-2,6-dien-1-yloxy)-2′-hydroxyphenyl)ethanone ((±)-**29**)..........**50**

Figure S9d. gHSQC spectrum of (±)-1-(4′-((2''*E*,6''*E*)-10,11-epoxy-3,7,11-trimethyldodeca-2,6-dien-1-yloxy)-2′-hydroxyphenyl)ethanone ((±)-**29**) in $CDCl_3$ **51**

Figure S9e. gHMBC spectrum of (±)-1-(4′-((2''*E*,6''*E*)-10,11-epoxy-3,7,11-trimethyldodeca-2,6-dien-1-yloxy)-2′-hydroxyphenyl)ethanone ((±)-**29**) in $CDCl_3$ **52**

Figure S10a. ^{1}H NMR spectrum (500 MHz) of (±)-2'-((2''*E*,6''*E*)-10,11-epoxy-3,7,11-trimethyldodeca-2,6-dien-1-yloxy)-[1,1'-biphenyl]-2-ol ((±)-**30**) in $CDCl_3$ **53**

Figure S10b. ^{13}C NMR spectrum (125 MHz) of (±)-2'-((2''*E*,6''*E*)-10,11-epoxy-3,7,11-trimethyldodeca-2,6-dien-1-yloxy)-[1,1'-biphenyl]-2-ol ((±)-**30**) in $CDCl_3$ **54**

Figure S10c. HRESIMS of (±)-2'-((2''*E*,6''*E*)-10,11-epoxy-3,7,11-trimethyldodeca-2,6-dien-1-yloxy)-[1,1'-biphenyl]-2-ol ((±)-**30**) **55**

Figure S10d. gHSQC spectrum of (±)-2'-((2''*E*,6''*E*)-10,11-epoxy-3,7,11-trimethyldodeca-2,6-dien-1-yloxy)-[1,1'-biphenyl]-2-ol ((±)-**30**) in $CDCl_3$ **56**

Figure S10e. gHMBC spectrum of (±)-2'-((2''*E*,6''*E*)-10,11-epoxy-3,7,11-trimethyldodeca-2,6-dien-1-yloxy)-[1,1'-biphenyl]-2-ol ((±)-**30**) in $CDCl_3$ **57**

Figure S11a. ^{1}H NMR spectrum (500 MHz) of (±)-7-(3'*R*(*S*),5'*S*(*R*),9'*R*(*S*),10'*R*(*S*)-3'-hydroxydrim-8'(12')-en-11'-yloxy)-coumarin ((±)-coladonin ((±)-**12**)) in $CDCl_3$. **58**

Figure S11b. ^{13}C NMR spectrum (125 MHz) of (±)-7-(3'*R*(*S*),5'*S*(*R*),9'*R*(*S*),10'*R*(*S*)-3'-hydroxydrim-8'(12')-en-11'-yloxy)-coumarin ((±)-coladonin ((±)-**12**)) in $CDCl_3$ **59**

Figure S11c. HRESIMS of (±)-7-(3'*R*(*S*),5'*S*(*R*),9'*R*(*S*),10'*R*(*S*)-3'-hydroxydrim-8'(12')-en-11'-yloxy)-coumarin ((±)-coladonin ((±)-**12**)) in $CDCl_3$ **60**

Figure S11d. gHSQC spectrum of (±)-7-(3'*R*(*S*),5'*S*(*R*),9'*R*(*S*),10'*R*(*S*)-3'-hydroxydrim-8'(12')-en-11'-yloxy)-coumarin ((±)-coladonin ((±)-**12**)) in $CDCl_3$ **61**

Figure S11e. gHMBC spectrum of (±)-7-(3'*R*(*S*),5'*S*(*R*),9'*R*(*S*),10'*R*(*S*)-3'-hydroxydrim-8'(12')-en-11'-yloxy)-coumarin ((±)-coladonin ((±)-**12**)) in $CDCl_3$ **62**

Figure S11f. NOESY2D spectrum of (±)-7-(3'*R*(*S*),5'*S*(*R*),9'*R*(*S*),10'*R*(*S*)-3'-hydroxydrim-8'(12')-en-11'-yloxy)-coumarin ((±)-coladonin ((±)-**12**)) in $CDCl_3$ **63**

Figure S11g. Expansion of NOESY2D spectrum of (±)-7-(3'*R*(*S*),5'*S*(*R*),9'*R*(*S*),10'*R*(*S*)-3'-hydroxydrim-8'(12')-en-11'-yloxy)-coumarin ((±)-coladonin ((±)-**12**)) in $CDCl_3$. Selected NOESY2D correlations are annotated and highlighted on (±)-coladonin ((±)-**12**) structure **64**

Figure S21. ^{1}H NMR spectrum (500 MHz) of (±)-7-(3'*R*(*S*),5'*S*(*R*),9'*R*(*S*),10'*R*(*S*)-3'-acetoxydrim-8'(12')-en-11'-yloxy)-coumarin ((±)-coladin ((±)-**13**)) in $CDCl_3$ **65**

Figure S12b. ^{13}C NMR spectrum (125 MHz) of (±)-7-(3'*R*(*S*),5'*S*(*R*),9'*R*(*S*),10'*R*(*S*)-3'-acetoxydrim-8'(12')-en-11'-yloxy)-coumarin ((±)-coladin ((±)-**13**)) in $CDCl_3$ **66**

Figure S12c. HRESIMS of (±)-7-(3'*R*(*S*),5'*S*(*R*),9'*R*(*S*),10'*R*(*S*)-3'-acetoxydrim-8'(12')-en-11'-yloxy)-coumarin ((±)-coladin ((±)-**13**)) in $CDCl_3$ **67**

Figure S13a. ^{1}H NMR spectrum (500 MHz) of (±)-7-(3'*R*(*S*),5'*S*(*R*),9'*R*(*S*),10'*R*(*S*)-3'-hydroxydrim-7'-en-11'-yloxy)-coumarin ((±)-feselol ((±)-**14**)) in $CDCl_3$ **68**

Figure S13b. ^{13}C NMR spectrum (125 MHz) of (±)-7-(3'*R*(*S*),5'*S*(*R*),9'*R*(*S*),10'*R*(*S*)-3'-hydroyidrim-7'-en-11'-yloxy)-coumarin ((±)-feselol ((±)-**14**)) in $CDCl_3$ **69**

Figure S13c. HRESIMS of (±)-7-(3'*R*(*S*),5'*S*(*R*),9'*R*(*S*),10'*R*(*S*)-3'-hydroyidrim-7'-en-11'-yloxy)-coumarin ((±)-feselol ((±)-**14**)) in $CDCl_3$ **70**

Figure S13d. gHSQC spectrum of (±)-7-(3'*R*(*S*),5'*S*(*R*),9'*R*(*S*),10'*R*(*S*)-3'-hydroxydrim-7'-en-11'-yloxy)-coumarin ((±)-feselol ((±)-**14**)) in $CDCl_3$ **71**

Figure S13e. gHMBC spectrum of (±)-7-(3'*R*(*S*),5'*S*(*R*),9'*R*(*S*),10'*R*(*S*)-3'-hydroxydrim-7'-en-11'-yloxy)-coumarin ((±)-feselol ((±)-**14**)) in $CDCl_3$ **72**

Figure S13f. NOESY2D spectrum of (±)-7-(3'*R*(*S*),5'*S*(*R*),9'*R*(*S*),10'*R*(*S*)-3'-hydroxydrim-7'-en-11'-yloxy)-coumarin ((±)-feselol ((±)-**14**)) in $CDCl_3$...**73**

Figure S13g. Expansion of NOESY2D spectrum of (±)-7-(3'*R*(*S*),5'*S*(*R*),9'*R*(*S*),10'*R*(*S*)-3'-hydroxydrim-7'-en-11'-yloxy)-coumarin ((±)-feselol ((±)-**14**)) in $CDCl_3$. Selected NOESY2D correlations are annotated and highlighted on (±)-feselol ((±)-**14**) structure…................................**74**

Figure S14a. ^{1}H NMR spectrum (500 MHz) of 7-(3'*R*(*S*),5'*S*(*R*),9'*R*(*S*),10'*R*(*S*)-3'-hydroxydrim-8'(12')-en-11'-yloxy)-4-methycoumarin ((±)-**31**) in $CDCl_3$...**75**

Figure S14b. ^{13}C NMR spectrum (125 MHz) of 7-(3'*R*(*S*),5'*S*(*R*),9'*R*(*S*),10'*R*(*S*)-3'-hydroxydrim-8'(12')-en-11'-yloxy)-4-methycoumarin ((±)-**31**) in $CDCl_3$...**76**

Figure S14c. HRESIMS of 7-(3'*R*(*S*),5'*S*(*R*),9'*R*(*S*),10'*R*(*S*)-3'-hydroxydrim-8'(12')-en-11'-yloxy)-4-methycoumarin ((±)-**31**)...**77**

Figure S14d. gHSQC spectrum of 7-(3'*R*(*S*),5'*S*(*R*),9'*R*(*S*),10'*R*(*S*)-3'-hydroxydrim-8'(12')-en-11'-yloxy)-4-methycoumarin ((±)-**31**) in $CDCl_3$...**78**

Figure S14e. gHMBC spectrum of 7-(3'*R*(*S*),5'*S*(*R*),9'*R*(*S*),10'*R*(*S*)-3'-hydroxydrim-8'(12')-en-11'-yloxy)-4-methycoumarin ((±)-**31**) in $CDCl_3$...**79**

Figure S14f. NOESY2D spectrum of 7-(3'*R*(*S*),5'*S*(*R*),9'*R*(*S*),10'*R*(*S*)-3'-hydroxydrim-8'(12')-en-11'-yloxy)-4-methycoumarin ((±)-**31**) in $CDCl_3$...**80**

Figure S15a. ^{1}H NMR spectrum (500 MHz) of 7-(3'*R*(*S*),5'*S*(*R*),9'*R*(*S*),10'*R*(*S*)-3'-hydroxydrim-7'-en-11'-yloxy)-4-methylcoumarin ((±)-**32**) in $CDCl_3$...**81**

Figure S15b. ^{13}C NMR spectrum (125 MHz) of 7-(3'*R*(*S*),5'*S*(*R*),9'*R*(*S*),10'*R*(*S*)-3'-hydroxydrim-7'-en-11'-yloxy)-4-methylcoumarin ((±)-**32**) in $CDCl_3$...**82**

Figure S15c. HRESIMS of 7-(3'*R*(*S*),5'*S*(*R*),9'*R*(*S*),10'*R*(*S*)-3'-hydroxydrim-7'-en-11'-yloxy)-4-methylcoumarin ((±)-**32**)...**83**

Figure S15d. gHSQC spectrum of 7-(3'*R*(*S*),5'*S*(*R*),9'*R*(*S*),10'*R*(*S*)-3'-hydroxydrim-7'-en-11'-yloxy)-4-methylcoumarin ((±)-**32**) in $CDCl_3$...**84**

Figure S15e. gHMBC spectrum of 7-(3'*R*(*S*),5'*S*(*R*),9'*R*(*S*),10'*R*(*S*)-3'-hydroxydrim-7'-en-11'-yloxy)-4-methylcoumarin ((±)-**32**) in $CDCl_3$...**85**

Figure S15f. NOESY2D spectrum of 7-(3'*R*(*S*),5'*S*(*R*),9'*R*(*S*),10'*R*(*S*)-3'-hydroxydrim-7'-en-11'-yloxy)-4-methylcoumarin ((±)-**32**) in $CDCl_3$...**86**

Figure S16a. ^{1}H NMR spectrum (500 MHz) of 1-(2′-(3′′*R*(*S*),5''*S*(*R*),9''*R*(*S*),10''*R*(*S*)-3''-hydroxydrim-8''(12'')-en-11''-yloxy)phenyl)ethanone ((±)-**33**) in $CDCl_3$...**87**

Figure S16b. ^{13}C NMR spectrum (125 MHz) of 1-(2′-(3′′*R*(*S*),5''*S*(*R*),9''*R*(*S*),10''*R*(*S*)-3''-hydroxydrim-8''(12'')-en-11''-yloxy)phenyl)ethanone ((±)-**33**) in $CDCl_3$...**88**

Figure S16c. HRESIMS of 1-(2′-(3′′*R*(*S*),5''*S*(*R*),9''*R*(*S*),10''*R*(*S*)-3''-hydroxydrim-8''(12'')-en-11''-yloxy)phenyl)ethanone ((±)-**33**)...**89**

Figure S16d. gHSQC spectrum of 1-(2′-(3''*R*(*S*),5''*S*(*R*),9''*R*(*S*),10''*R*(*S*)-3''-hydroxydrim-8''(12'')-en-11''-yloxy)phenyl)ethanone ((±)-**33**) in $CDCl_3$...**90**

Figure S16e. gHMBC spectrum of 1-(2′-(3''*R*(*S*),5''*S*(*R*),9''*R*(*S*),10''*R*(*S*)-3''-hydroxydrim-8''(12'')-en-11''-yloxy)phenyl)ethanone ((±)-**33**) in $CDCl_3$...**91**

Figure S16f. NOESY2D spectrum of 1-(2′-(3′′*R*(*S*),5''*S*(*R*),9''*R*(*S*),10''*R*(*S*)-3''-hydroxydrim-8''(12'')-en-11''-yloxy)phenyl)ethanone ((±)-**33**) in $CDCl_3$...**92**

Figure S17a. ^{1}H NMR spectrum (500 MHz) of 1-(2′-(3''*R*(*S*),5''*S*(*R*),9''*R*(*S*),10''*R*(*S*)-3''-hydroxydrim-7''-en-11''-yloxy)phenyl)ethanone ((±)-**34**) in $CDCl_3$...**93**

Figure S17b. ^{13}C NMR spectrum (125 MHz) of 1-(2′-(3''*R*(*S*),5''*S*(*R*),9''*R*(*S*),10''*R*(*S*)-3''-hydroxydrim-7''-en-11''-yloxy)phenyl)ethanone ((±)-**34**) in $CDCl_3$...**94**

Figure S17c. HRESIMS of 1-(2′-(3''*R*(*S*),5''*S*(*R*),9''*R*(*S*),10''*R*(*S*)-3''-hydroxydrim-7''-en-11''-yloxy)phenyl)ethanone ((±)-**34**)....................95

Figure S17d. gHSQC spectrum of 1-(2′-(3''*R*(*S*),5''*S*(*R*),9''*R*(*S*),10''*R*(*S*)-3''-hydroxydrim-7''-en-11''-yloxy)phenyl)ethanone ((±)-**34**) in $CDCl_3$....................96

Figure S17e. gHMBC spectrum of 1-(2′-(3''*R*(*S*),5''*S*(*R*),9''*R*(*S*),10''*R*(*S*)-3''-hydroxydrim-7''-en-11''-yloxy)phenyl)ethanone ((±)-**34**) in $CDCl_3$....................97

Figure S17f. NOESY2D spectrum of 1-(2′-(3''*R*(*S*),5''*S*(*R*),9''*R*(*S*),10''*R*(*S*)-3''-hydroxydrim-7''-en-11''-yloxy)phenyl)ethanone ((±)-**34**) in $CDCl_3$....................98

Figure S18a. ^{1}H NMR spectrum (500 MHz) of 1-(2′-hydroxy-4′-(3''*R*(*S*),5''*S*(*R*),9''*R*(*S*),10''*R*(*S*)-3''-hydroxydrim-8''(12'')-en-11''-yloxy)phenyl)ethanone ((±)-**35**) in $CDCl_3$....................99

Figure S18b. ^{13}C NMR spectrum (125 MHz) of 1-(2′-hydroxy-4′-(3''*R*(*S*),5''*S*(*R*),9''*R*(*S*),10''*R*(*S*)-3''-hydroxydrim-8''(12'')-en-11''-yloxy)phenyl)ethanone ((±)-**35**) in $CDCl_3$....................100

Figure S18c. HRESIMS of 1-(2′-hydroxy-4′-(3''*R*(*S*),5''*S*(*R*),9''*R*(*S*),10''*R*(*S*)-3''-hydroxydrim-8''(12'')-en-11''-yloxy)phenyl)ethanone ((±)-**35**)....................101

Figure S18d. gHSQC spectrum of 1-(2′-hydroxy-4′-(3''*R*(*S*),5''*S*(*R*),9''*R*(*S*),10''*R*(*S*)-3''-hydroxydrim-8''(12'')-en-11''-yloxy)phenyl)ethanone ((±)-**35**) in $CDCl_3$....................102

Figure S18e. gHMBC spectrum of 1-(2′-hydroxy-4′-(3''*R*(*S*),5''*S*(*R*),9''*R*(*S*),10''*R*(*S*)-3''-hydroxydrim-8''(12'')-en-11''-yloxy)phenyl)ethanone ((±)-**35**) in $CDCl_3$....................103

Figure S18f. NOESY2D spectrum of 1-(2′-hydroxy-4′-(3''*R*(*S*),5''*S*(*R*),9''*R*(*S*),10''*R*(*S*)-3''-hydroxydrim-8''(12'')-en-11''-yloxy)phenyl)ethanone ((±)-**35**) in $CDCl_3$....................104

Figure S19a. ^{1}H NMR spectrum (400 MHz) of 1-(2′-hydroxy-4′-(3''*R*(*S*),5''*S*(*R*),9''*R*(*S*),10''*R*(*S*)-3''-hydroxydrim-7''-en-11''-yloxy)phenyl)ethanone ((±)-**36**) in $CDCl_3$....................105

Figure S19b. ^{13}C NMR spectrum (100 MHz) of 1-(2′-hydroxy-4′-(3''*R*(*S*),5''*S*(*R*),9''*R*(*S*),10''*R*(*S*)-3''-hydroxydrim-7''-en-11''-yloxy)phenyl)ethanone ((±)-**36**) in $CDCl_3$....................106

Figure S19c. HRESIMS of 1-(2′-hydroxy-4′-(3''*R*(*S*),5''*S*(*R*),9''*R*(*S*),10''*R*(*S*)-3''-hydroxydrim-7''-en-11''-yloxy)phenyl)ethanone ((±)-**36**)....................107

Figure S19d. gHSQC spectrum of 1-(2′-hydroxy-4′-(3''*R*(*S*),5''*S*(*R*),9''*R*(*S*),10''*R*(*S*)-3''-hydroxydrim-7''-en-11''-yloxy)phenyl)ethanone ((±)-**36**) in $CDCl_3$....................108

Figure S19e. gHMBC spectrum of 1-(2′-hydroxy-4′-(3''*R*(*S*),5''*S*(*R*),9''*R*(*S*),10''*R*(*S*)-3''-hydroxydrim-7''-en-11''-yloxy)phenyl)ethanone ((±)-**36**) in $CDCl_3$....................109

Figure S19f. NOESY2D spectrum of 1-(2′-hydroxy-4′-(3''*R*(*S*),5''*S*(*R*),9''*R*(*S*),10''*R*(*S*)-3''-hydroxydrim-7''-en-11''-yloxy)phenyl)ethanone ((±)-**36**) in $CDCl_3$....................110

Figure S20a. ^{1}H NMR spectrum (500 MHz) of 2'-(3''*R*(*S*),5''*S*(*R*),9''*R*(*S*),10''*R*(*S*)-3''-hydroxydrim-8''(12'')-en-11''-yloxy)-[1,1'-biphenyl]-2-ol ((±)-**37**) in $CDCl_3$....................111

Figure S20b. ^{13}C NMR spectrum (125 MHz) of 2'-(3''*R*(*S*),5''*S*(*R*),9''*R*(*S*),10''*R*(*S*)-3''-hydroxydrim-8''(12'')-en-11''-yloxy)-[1,1'-biphenyl]-2-ol ((±)-**37**) in $CDCl_3$....................112

Figure S20c. HRESIMS of 2'-(3''*R*(*S*),5''*S*(*R*),9''*R*(*S*),10''*R*(*S*)-3''-hydroxydrim-8''(12'')-en-11''-yloxy)-[1,1'-biphenyl]-2-ol ((±)-**37**)....................113

Figure S20d. gHSQC spectrum of 2'-(3''*R*(*S*),5''*S*(*R*),9''*R*(*S*),10''*R*(*S*)-3''-hydroxydrim-8''(12'')-en-11''-yloxy)-[1,1'-biphenyl]-2-ol ((±)-**37**) in $CDCl_3$....................114

Figure S20e. gHMBC spectrum of 2'-(3''*R*(*S*),5''*S*(*R*),9''*R*(*S*),10''*R*(*S*)-3''-hydroxydrim-8''(12'')-en-11''-yloxy)-[1,1'-biphenyl]-2-ol ((±)-**37**) in $CDCl_3$....................115

Figure S21a. ^{1}H NMR spectrum (500 MHz) of 2'-(3''*R*(*S*),5''*S*(*R*), 8''*R*(*S*),9''*R*(*S*),10''*R*(*S*)-3''-hydroxydriman-11''-yloxy)-[1,1'-biphenyl]-2-ol ((±)-**38**) in $CDCl_3$....................116

Figure S21b. ^{13}C NMR spectrum (125 MHz) of 2'-(3''*R*(*S*),5''*S*(*R*), 8''*R*(*S*),9''*R*(*S*),10''*R*(*S*)-3''-hydroxydriman-11''-yloxy)-[1,1'-biphenyl]-2-ol ((±)-**38**) in $CDCl_3$........**117**

Figure S21c. HRESIMS of 2'-(3''*R*(*S*),5''*S*(*R*), 8''*R*(*S*),9''*R*(*S*),10''*R*(*S*)-3''-hydroxydriman-11''-yloxy)-[1,1'-biphenyl]-2-ol ((±)-**38**)........**118**

Figure S21d. gHSQC spectrum of 2'-(3''*R*(*S*),5''*S*(*R*), 8''*R*(*S*),9''*R*(*S*),10''*R*(*S*)-3''-hydroxydriman-11''-yloxy)-[1,1'-biphenyl]-2-ol ((±)-**38**) in $CDCl_3$........**119**

Figure S21e. gHMBC spectrum of 2'-(3''*R*(*S*),5''*S*(*R*), 8''*R*(*S*),9''*R*(*S*),10''*R*(*S*)-3''-hydroxydriman-11''-yloxy)-[1,1'-biphenyl]-2-ol ((±)-**38**) in $CDCl_3$........**120**

Figure S21f. NOESY2D spectrum of 2'-(3''*R*(*S*),5''*S*(*R*), 8''*R*(*S*),9''*R*(*S*),10''*R*(*S*)-3''-hydroxydriman-11''-yloxy)-[1,1'-biphenyl]-2-ol ((±)-**38**) in $CDCl_3$........**121**

Figure S21g. Expansion of NOESY2D spectrum of 2'-(3''*R*(*S*),5''*S*(*R*), 8''*R*(*S*),9''*R*(*S*),10''*R*(*S*)-3''-hydroxydriman-11''-yloxy)-[1,1'-biphenyl]-2-ol ((±)-**38**) in $CDCl_3$. Selected NOESY2D correlations are annotated and highlighted on (±)-**38** structure........**122**

Figure S22a. ^{1}H NMR spectrum (500 MHz) of 7-(3'*R*(*S*),5'*S*(*R*), 8'*S*(*R*),9'*R*(*S*),10'*R*(*S*)-8',12'-epoxy-3'-hydroxydriman-11'-yloxy)-coumarin ((±)-**39**) in $CDCl_3$........**123**

Figure S22b. ^{13}C NMR spectrum (125 MHz) of 7-(3'*R*(*S*),5'*S*(*R*),8'*S*(*R*),9'*R*(*S*),10'*R*(*S*)-8',12'-epoxy-3'-hydroxydriman-11'-yloxy)-coumarin ((±)-**39**) in $CDCl_3$........**124**

Figure S22c. HRESIMS of 7-(3'*R*(*S*),5'*S*(*R*), 8'*S*(*R*),9'*R*(*S*),10'*R*(*S*)-8',12'-epoxy-3'-hydroxydriman-11'-yloxy)-coumarin ((±)-**39**)........**125**

Figure S22d. gHSQC spectrum of 7-(3'*R*(*S*),5'*S*(*R*),8'*S*(*R*),9'*R*(*S*),10'*R*(*S*)-8',12'-epoxy-3'-hydroxydriman-11'-yloxy)-coumarin ((±)-**39**) in $CDCl_3$........**126**

Figure S22e. gHMBC spectrum of 7-(3'*R*(*S*),5'*S*(*R*),8'*S*(*R*),9'*R*(*S*),10'*R*(*S*)-8',12'-epoxy-3'-hydroxydriman-11'-yloxy)-coumarin ((±)-**39**) in $CDCl_3$........**127**

Figure S22f. NOESY2D spectrum of 7-(3'*R*(*S*),5'*S*(*R*),8'*S*(*R*),9'*R*(*S*),10'*R*(*S*)-8',12'-epoxy-3'-hydroxydriman-11'-yloxy)-coumarin ((±)-**39**) in $CDCl_3$........**128**

Figure S22g. Expansion of NOESY2D spectrum of 7-(3'*R*(*S*),5'*S*(*R*),8'*S*(*R*),9'*R*(*S*),10'*R*(*S*)-8',12'-Epoxy-3'-hydroxydriman-11'-yloxy)-coumarin ((±)-**39**) in $CDCl_3$. Selected NOESY2D correlations are annotated and highlighted on (±)-**39** structure........**129**

Figure S23a. ^{1}H NMR spectrum (400 MHz) of 7-(5'*R*(*S*),9'*R*(*S*),10'*R*(*S*)-drima-3',8'(12')-dien-11'-iloxy)-coumarin ((±)-**40**) in $CDCl_3$........**130**

Figure S23b. ^{13}C NMR spectrum (100 MHz) of 7-(5'*R*(*S*),9'*R*(*S*),10'*R*(*S*)-drima-3',8'(12')-dien-11'-iloxy)-coumarin ((±)-**40**) in $CDCl_3$........**131**

Figure S23c. HRESIMS of 7-(5'*R*(*S*),9'*R*(*S*),10'*R*(*S*)-drima-3',8'(12')-dien-11'-iloxy)-coumarin ((±)-**40**)........**132**

Figure S23d. gHSQC spectrum of 7-(5'*R*(*S*),9'*R*(*S*),10'*R*(*S*)-drima-3',8'(12')-dien-11'-iloxy)-coumarin ((±)-**40**) in $CDCl_3$........**133**

Figure S23e. gHMBC spectrum of 7-(5'*R*(*S*),9'*R*(*S*),10'*R*(*S*)-drima-3',8'(12')-dien-11'-iloxy)-coumarin ((±)-**40**) in $CDCl_3$........**134**

Table S1-^{1}H. Comparison of ^{1}H-NMR spectroscopic data for (±)-**12** versus (-)-**12** and (±)-**14** versus (+)-**14**........**135**

Table S1-^{13}C. Comparison of ^{13}C-NMR spectroscopic data for (±)-**12** versus (-)-**12** and (±)-**14** versus (+)-**14**........**136**

Table S2. Molar doses for broth microdilution assays against *B. cinerea* for compounds (±)-**12**-(±)-**14**, **21**-**25**, (±)-**26**-(±)-**33**, (±)-**35**-(±)-**40**, triclosan and azoxystrobin (10^{-5} mg/mL versus 10^{-6} M)........**137**

Table S3. Inhibition of fungal growth percentage (IFG%) against *B. cinerea* for compounds (±)**-12**-(±)**-14**, **21-25**, (±)**-26**-(±)**-33**, (±)**-35**-(±)**-40**, triclosan (**C1+**) and azoxystrobin (**C2+**). (6250 10^{-5} mg/mL, 391 10^{-5} mg/mL and 49 10^{-5} mg/mL doses; see molar doses for every compound in **Table S2**)……………………………………………………………………………**138**

Figure S24. Comparison of inhibition of fungal growth percentage (IFG%) among compounds (±)**-12**-(±)**-14**, **21-25**, (±)**-26**-(±)**-33**, (±)**-35**-(±)**-40**, triclosan (**C1+**) and azoxystrobin (**C2+**) (*B. cinerea*, 6250 10^{-5} mg/mL dose; see molar doses for every compound in **Table S2**). Data are presented as mean ± standard deviation. Differences in IFG% between compounds are presented in uppercase Latin: different letters represent statistically significant differences between compounds; p<0.05……………………………………………………………………………**139**

Figure S25. Comparison of inhibition of fungal growth percentage (IFG%) among compounds (±)**-12**-(±)**-14**, **21-25**, (±)**-26**-(±)**-33**, (±)**-35**-(±)**-40**, triclosan (**C1+**) and azoxystrobin (**C2+**) (*B. cinerea*, 391 10^{-5} mg/mL dose - 16-fold dilution from 6250 10^{-5} mg/mL dose -; see molar doses for every compound in **Table S2**); Data are presented as mean ± standard deviation. Differences in IFG% between compounds are presented in uppercase Latin: different letters represent statistically significant differences between compounds; p<0.05……………………………………**139**

Figure S26. Comparison of inhibition of fungal growth percentage (IFG%) among compounds (±)**-12**-(±)**-14**, **21-25**, (±)**-26**-(±)**-33**, (±)**-35**-(±)**-40**, triclosan (**C1+**) and azoxystrobin (**C2+**) (*B. cinerea*, 49 10^{-5} mg/mL dose - 128-fold dilution from 6250 10^{-5} mg/mL dose -; see molar doses for every compound in **Table S2**). Data are presented as mean ± standard deviation. Differences in IFG% between compounds are presented in uppercase Latin: different letters represent statistically significant differences between compounds; p<0.05……………………………………………………………………………**140**

Table S4. Calculated (www.molinspiration.com) logP, total polar surface area (TPSA), hydrogen bond donor atoms (HBA) and hydroxyl groups (OH) for compounds (±)**-12**-(±)**-14**, (±)**-31 -** (±)**-33**, (±)**-35 -** (±)**-40**……………………………………………………………………………**140**

Figure S27. Comparison of inhibition of fungal growth percentage (IFG%) (*B. cinerea*, 6250 10^{-5} mg/mL dose; see molar doses for every compound in table S2) versus LogP for compounds (±)**-12**-(±)**-14**, (±)**-31**-(±)**-33**, (±)**-35**-(±)**-40**. In shaded blue, compounds falling in the logP range 5.68-5.07; circled in orange, compounds also displaying TPSA values within range 66.76 -59.67 Å^2 (see Figure S28)……………………………………………………………………………**141**

Figure S28. Comparison of inhibition of fungal growth percentage (IFG%) (*B. cinerea*, 6250 10^{-5} mg/mL dose; see molar doses for every compound in table S2) versus total polar surface area (TPSA) for compounds (±)**-12**-(±)**-14**, (±)**-31**-(±)**-33**, (±)**-35**-(±)**-40**. In shaded orange, compounds falling in the TPSA range 66.76 -59.67 Å^2; circled in blue, compounds also displaying logP values within range 5.68-5.07 (see Figure S27)……………………………………………**141**

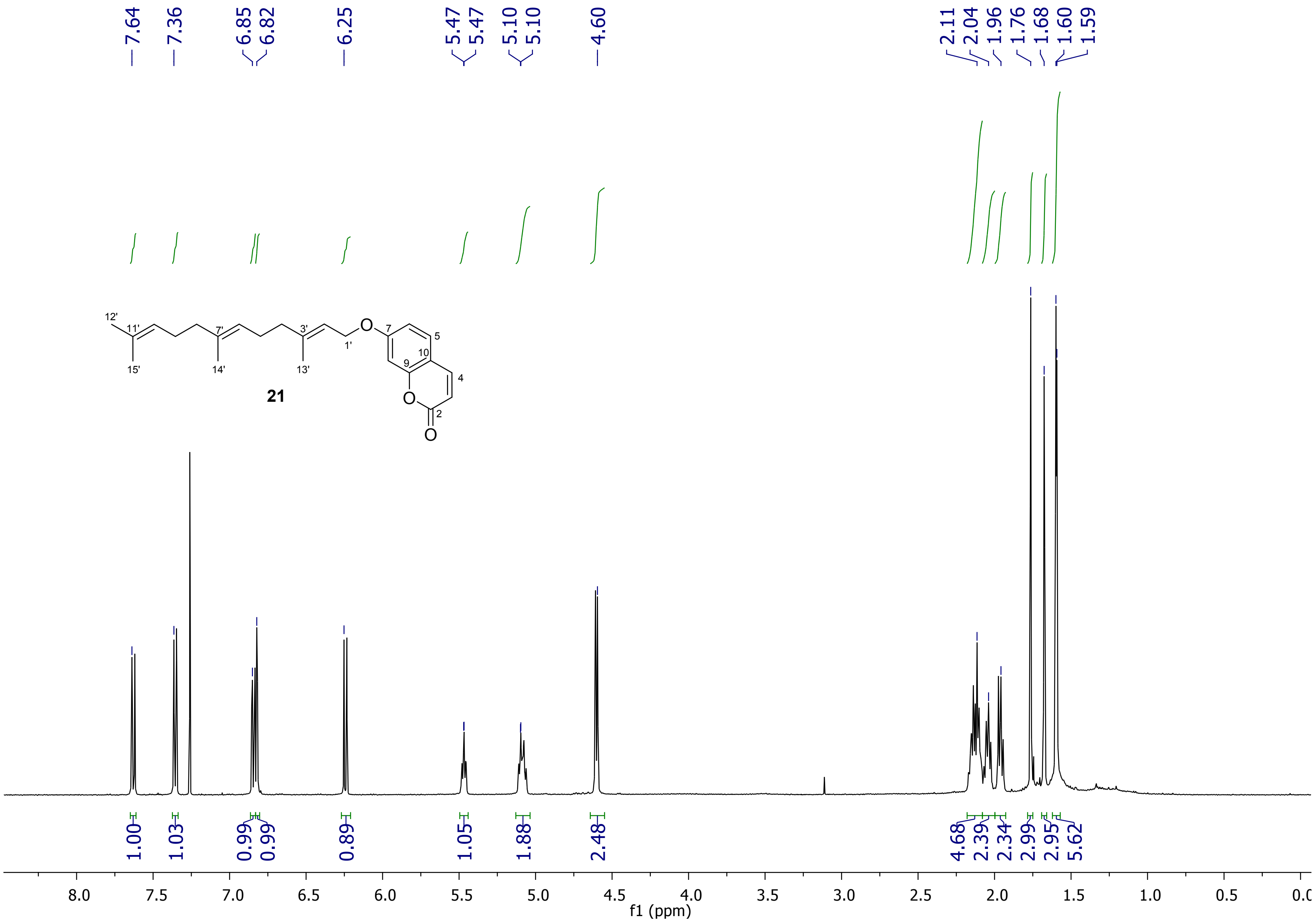

Figure S1a. ^{1}H NMR spectrum (400 MHz) of 7-(((2'*E*,6'*E*)-3,7,11-trimethyldodeca-2,6,10-trien-1-yl)oxy)-2*H*-chromen-2-one (umbelliprenin) (**21**) in $CDCl_3$.

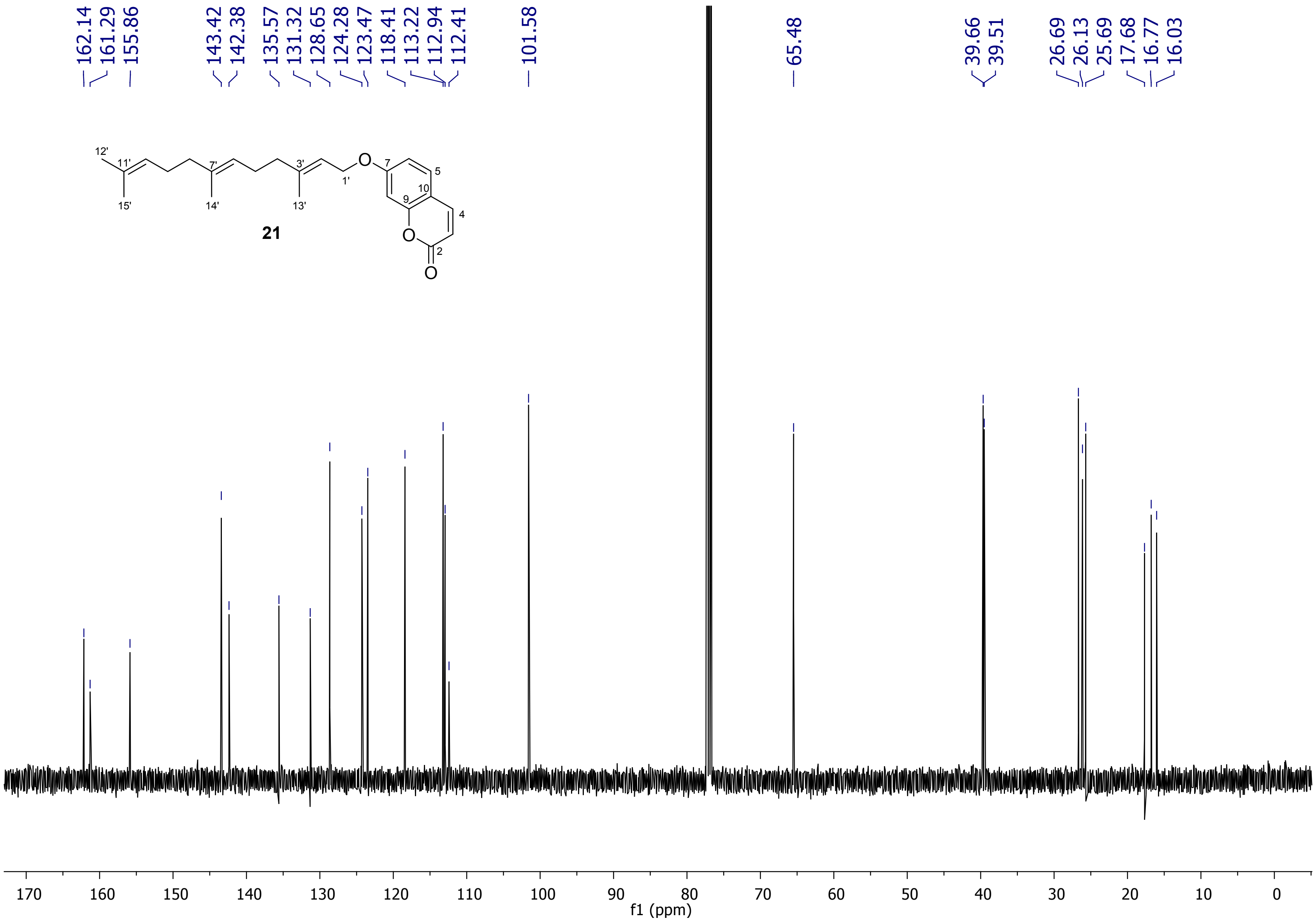

Figure S1b. ^{1}H NMR spectrum (100 MHz) of 7-(((2'*E*,6'*E*)-3,7,11-trimethyldodeca-2,6,10-trien-1-yl)oxy)-2*H*-chromen-2-one (umbelliprenin) (**21**) in $CDCl_3$.

Monoisotopic Mass, Even Electron Ions
141 formula(e) evaluated with 2 results within limits (up to 5 best isotopic matches for each mass)
Elements Used:
C: 0-50 H: 0-150 O: 0-20 Na: 0-1

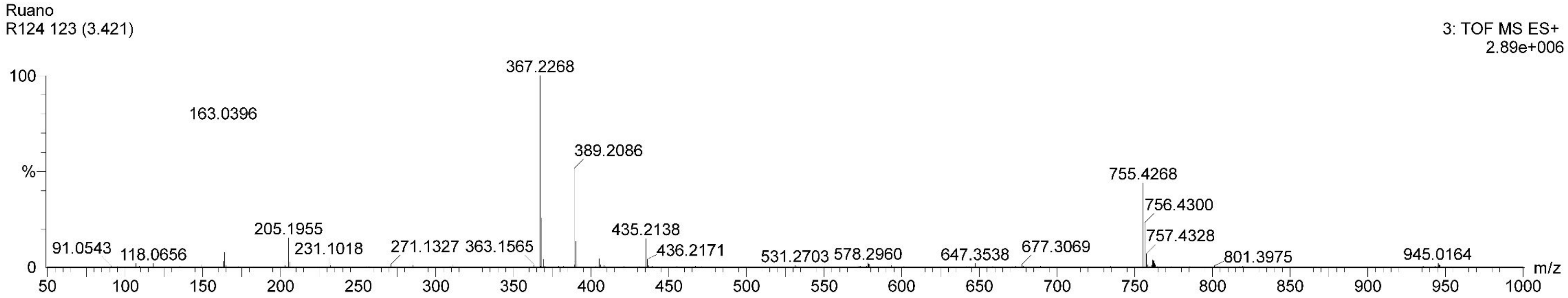

Minimum:				-1.5				
Maximum:		2.0	50.0	80.0				
Mass	Calc. Mass	mDa	PPM	DBE	i-FIT	Norm	Conf(%)	Formula
367.2268	367.2273	-0.5	-1.4	9.5	1917.5	0.002	99.79	C24 H31 O3
	367.2249	1.9	5.2	6.5	1923.6	6.150	0.21	C22 H32 O3 Na

Figure S1c. HRESIMS of 7-(((2'*E*,6'*E*)-3,7,11-trimethyldodeca-2,6,10-trien-1-yl)oxy)-2*H*-chromen-2-one (umbelliprenin) (**21**).

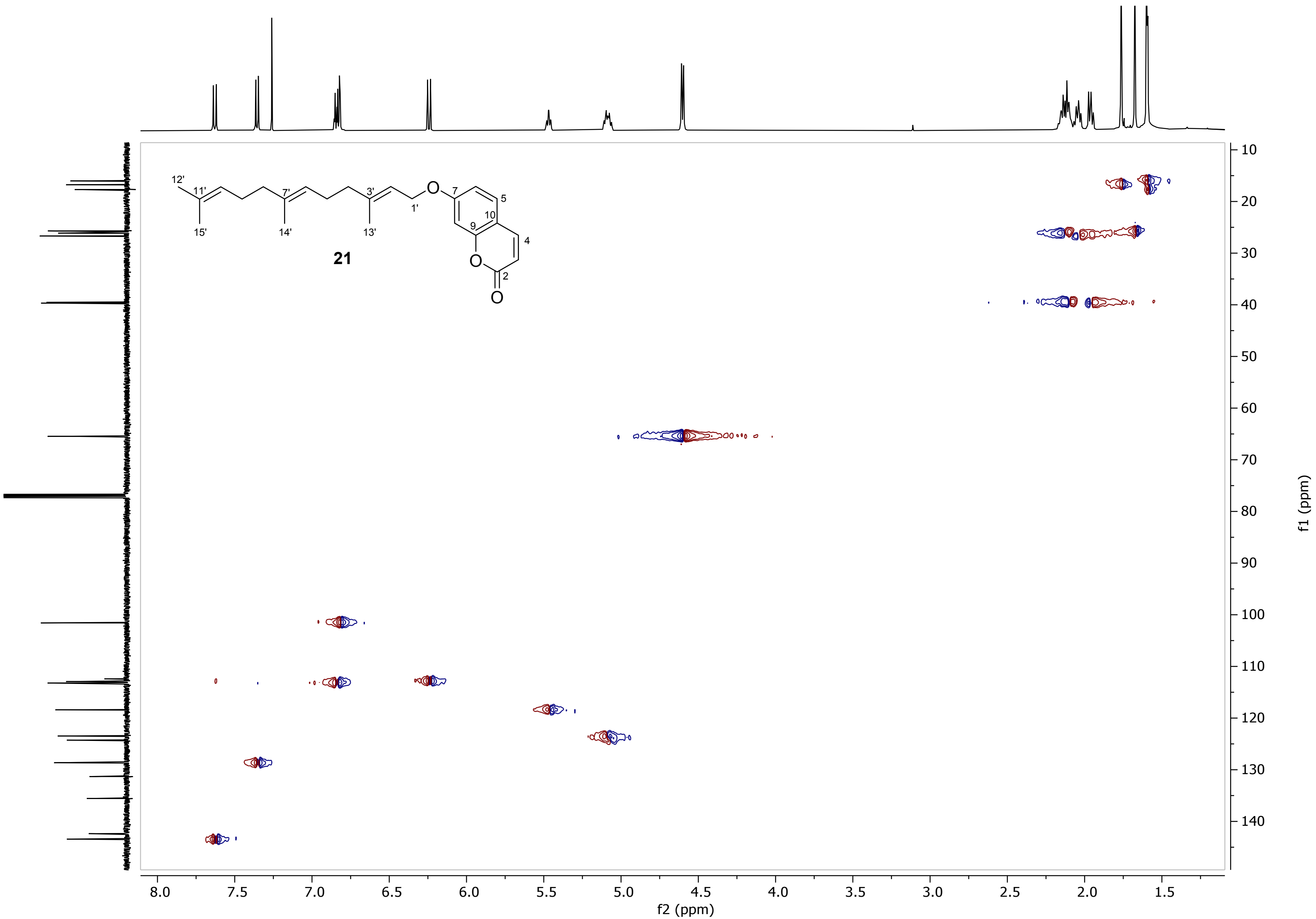

Figure S1d. gHSQC spectrum of 7-(((2'*E*,6'*E*)-3,7,11-trimethyldodeca-2,6,10-trien-1-yl)oxy)-2*H*-chromen-2-one (umbelliprenin) (**21**) in $CDCl_3$.

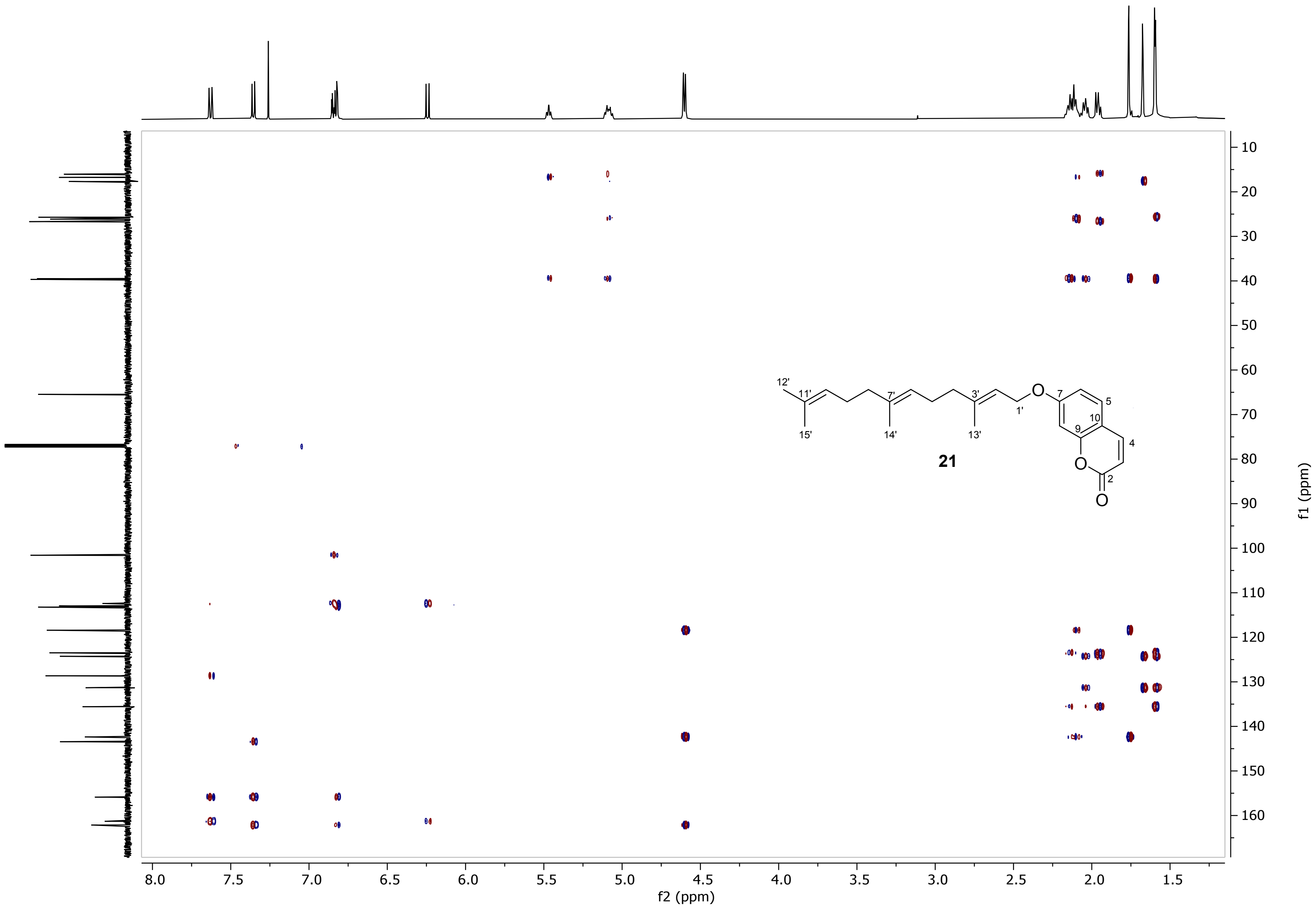

Figure S1e. gHMBC spectrum of 7-(((2'*E*,6'*E*)-3,7,11-trimethyldodeca-2,6,10-trien-1-yl)oxy)-2*H*-chromen-2-one (umbelliprenin) (**21**) in $CDCl_3$.

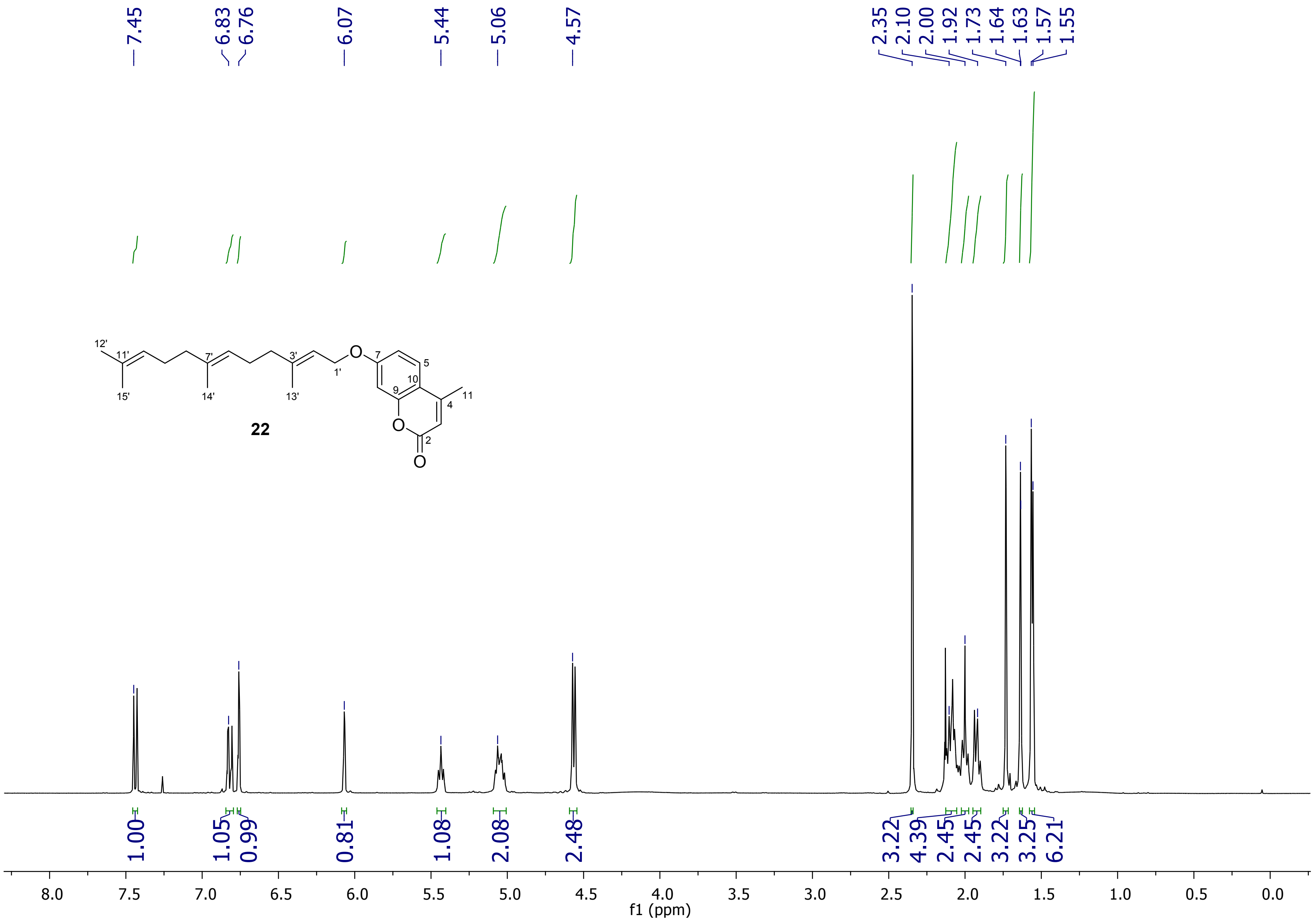

Figure S2a. ^{1}H NMR spectrum (400 MHz) of 4-methyl-7-(((2’*E*,6’*E*)-3,7,11-trimethyldodeca-2,6,10-trien-1-yl)oxy)-2*H*-chromen-2-one (7-farnesyloxy-4-methylumbelliferone) (**22**) in $CDCl_3$.

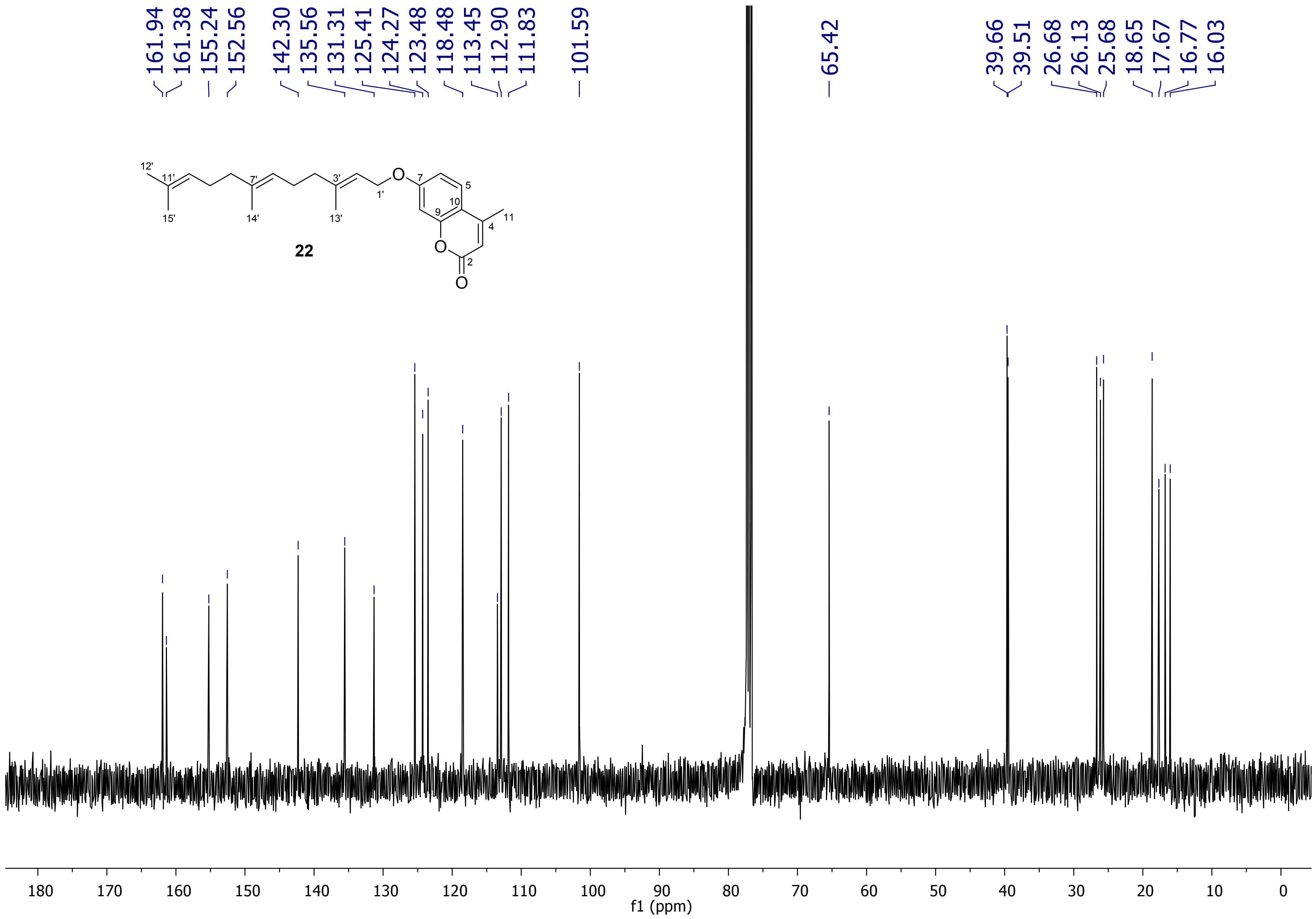

Figure S2b. ^{13}C NMR spectrum (100 MHz) of 4-methyl-7-(((2'*E*,6'*E*)-3,7,11-trimethyldodeca-2,6,10-trien-1-yl)oxy)-2*H*-chromen-2-one (7-farnesyloxy-4-methylumbelliferone) (**22**) in $CDCl_3$.

Monoisotopic Mass, Even Electron Ions
328 formula(e) evaluated with 3 results within limits (up to 50 best isotopic matches for each mass)
Elements Used:
C: 0-500 H: 0-1000 O: 0-200 Na: 0-1 Si: 0-1
AJM-114 224 (4.344)
4: TOF MS ES+

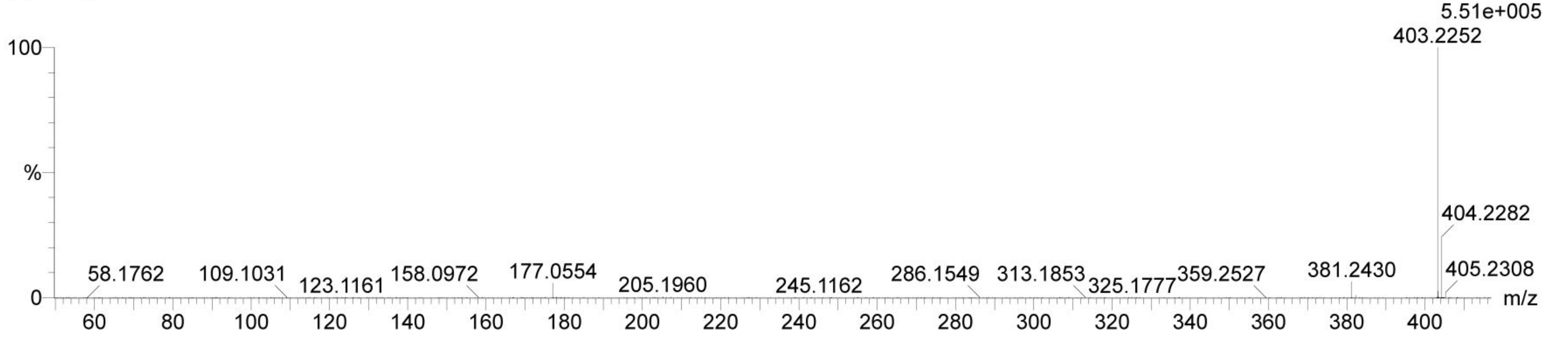

Mass	Calc. Mass	mDa	PPM	DBE	i-FIT	Norm	Conf(%)	Formula
Minimum:				-1.5				
Maximum:		5.0	10.0	50.0				
403.2252	403.2249	0.3	0.7	9.5	217.0	0.133	87.55	C25 H32 O3 Na
	403.2273	-2.1	-5.2	12.5	219.0	2.084	12.45	C27 H31 O3
	403.2281	-2.9	-7.2	4.5	226.6	9.719	0.01	C21 H36 O4 Na Si

Figure S2c. HRESIMS of 4-methyl-7-(((2'*E*,6'*E*)-3,7,11-trimethyldodeca-2,6,10-trien-1-yl)oxy)-2*H*-chromen-2-one (7-farnesyloxy-4-methylumbelliferone) (**22**).

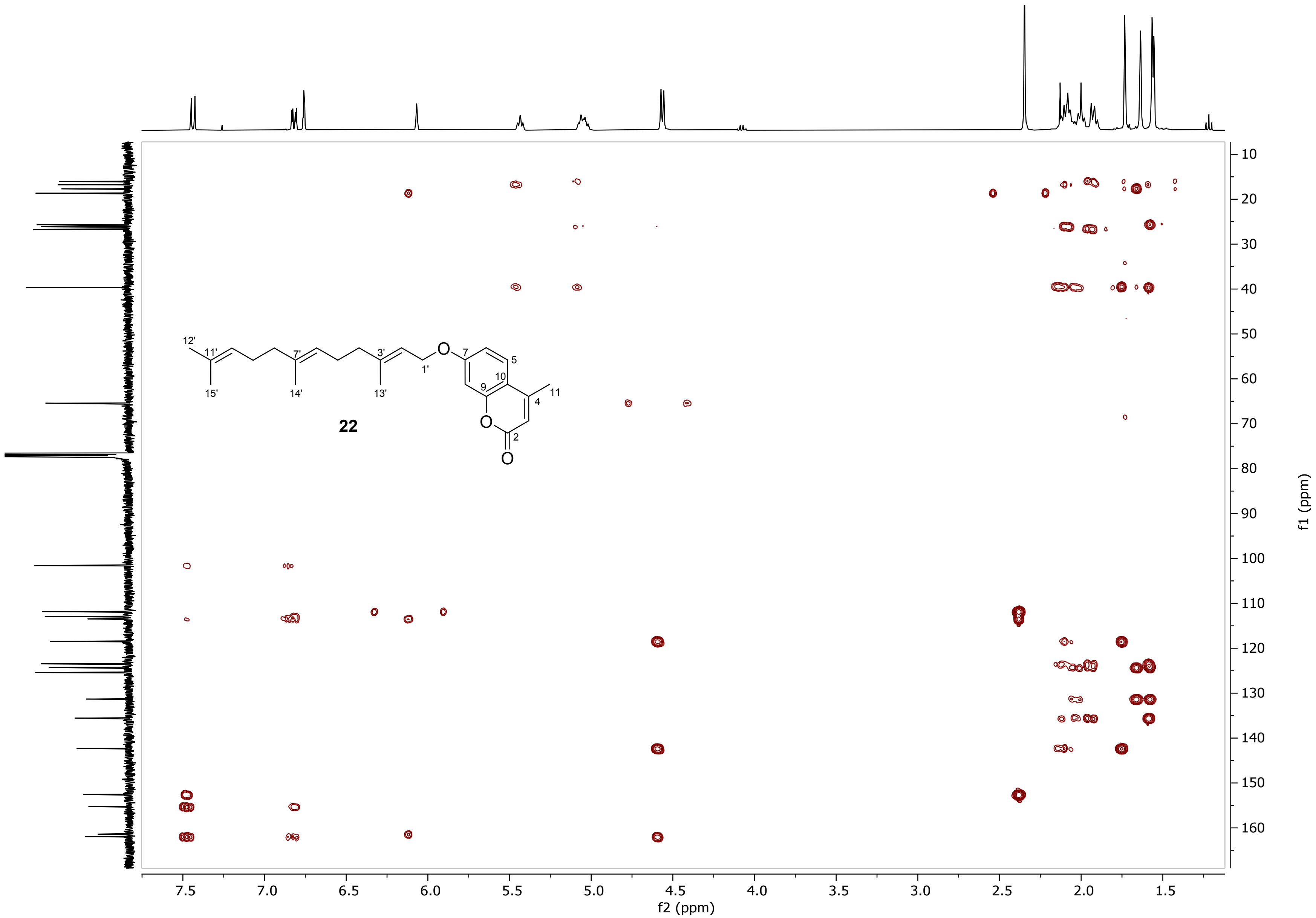

Figure S2d. gHMBC spectrum of 4-methyl-7-(((2’*E*,6’*E*)-3,7,11-trimethyldodeca-2,6,10-trien-1-yl)oxy)-2*H*-chromen-2-one (7-farnesyloxy-4-methylumbelliferone) (**22**) in $CDCl_3$.

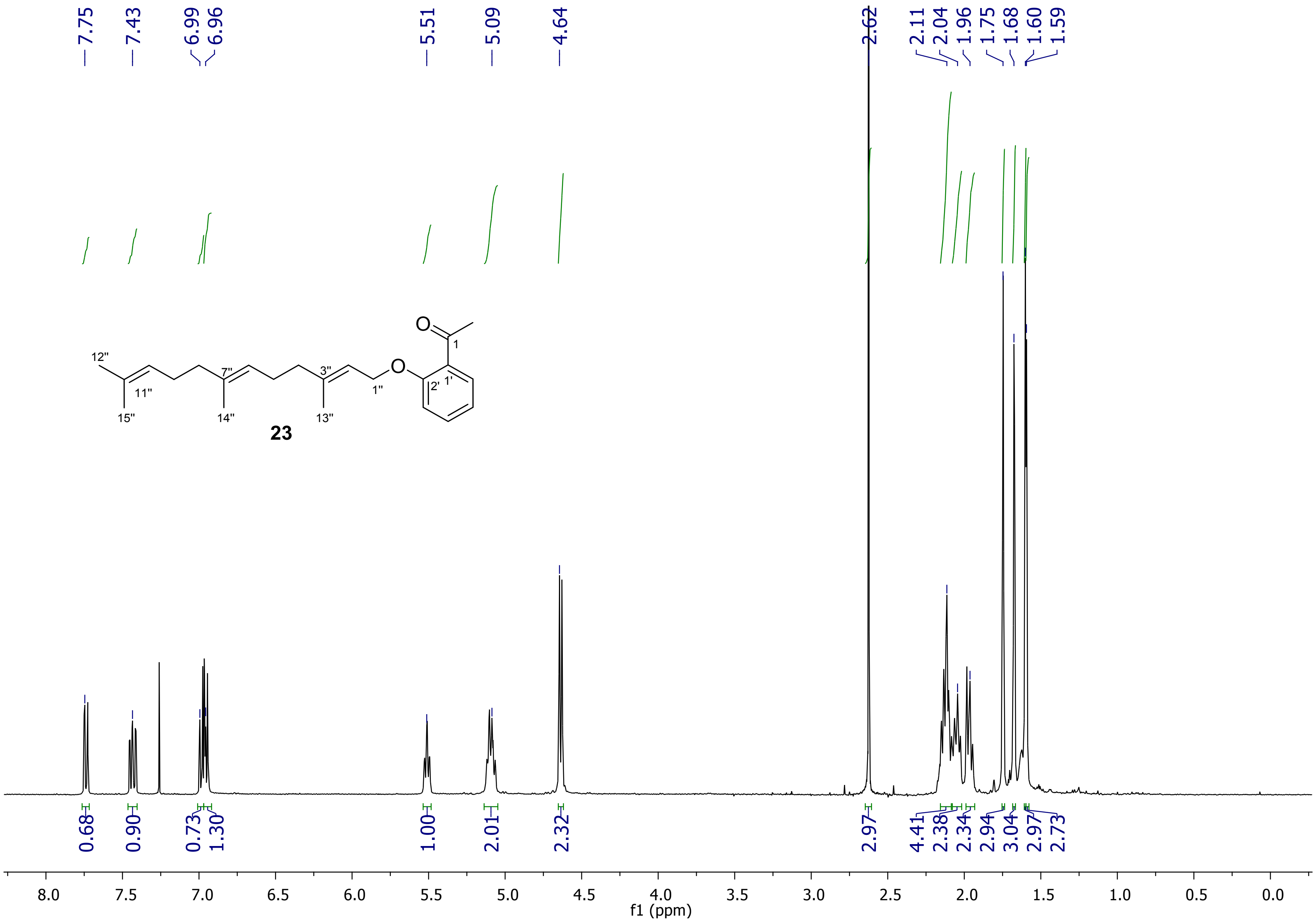

Figure S3a. ^{1}H NMR spectrum (400 MHz) of 1-(2’-(((2"*E*,6"*E*)-3,7,11-trimethyldodeca-2,6,10-trien-1-yl)oxy)phenyl)ethanone (**23**) in $CDCl_3$.

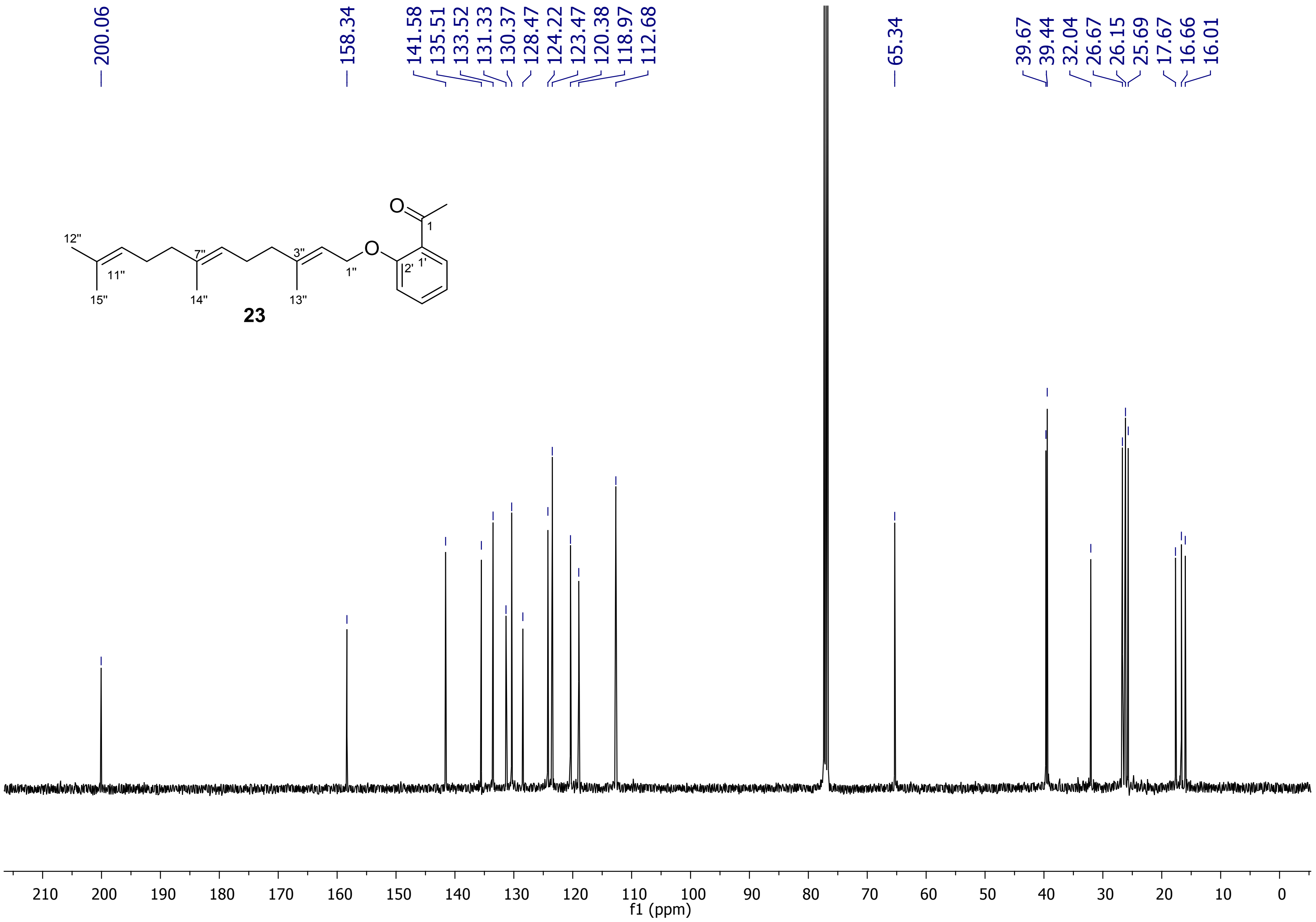

Figure S3b. ^{13}C NMR spectrum (100 MHz) of 1-(2'-(((2"*E*,6"*E*)-3,7,11-trimethyldodeca-2,6,10-trien-1-yl)oxy)phenyl)ethanone (**23**) in $CDCl_3$.

Monoisotopic Mass, Even Electron Ions
273 formula(e) evaluated with 3 results within limits (up to 50 best isotopic matches for each mass)
Elements Used:
C: 0-500 H: 0-1000 O: 0-200 Na: 0-1 Si: 0-1
AJM-15-1 220 (4.273)
5: TOF MS ES+

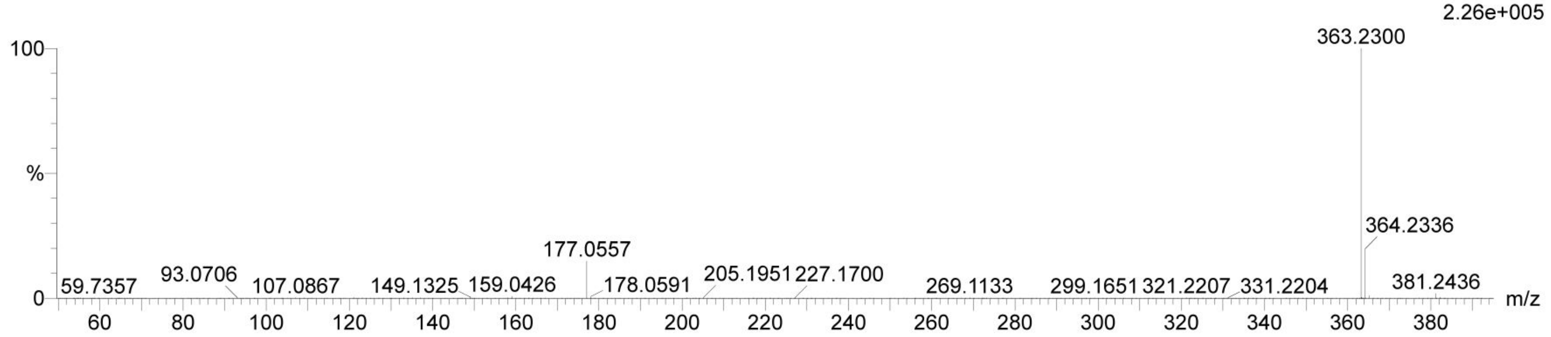

Minimum:				-1.5				
Maximum:		5.0	10.0	50.0				
Mass	Calc. Mass	mDa	PPM	DBE	i-FIT	Norm	Conf(%)	Formula
363.2300	363.2300	0.0	0.0	7.5	142.2	0.215	80.64	C23 H32 O2 Na
	363.2324	-2.4	-6.6	10.5	143.6	1.642	19.36	C25 H31 O2
	363.2331	-3.1	-8.5	2.5	153.1	11.138	0.00	C19 H36 O3 Na Si

Figure S3c. HRESIMS of 1-(2'-(((2''*E*,6''*E*)-3,7,11-trimethyldodeca-2,6,10-trien-1-yl)oxy)phenyl)ethanone (**23**)

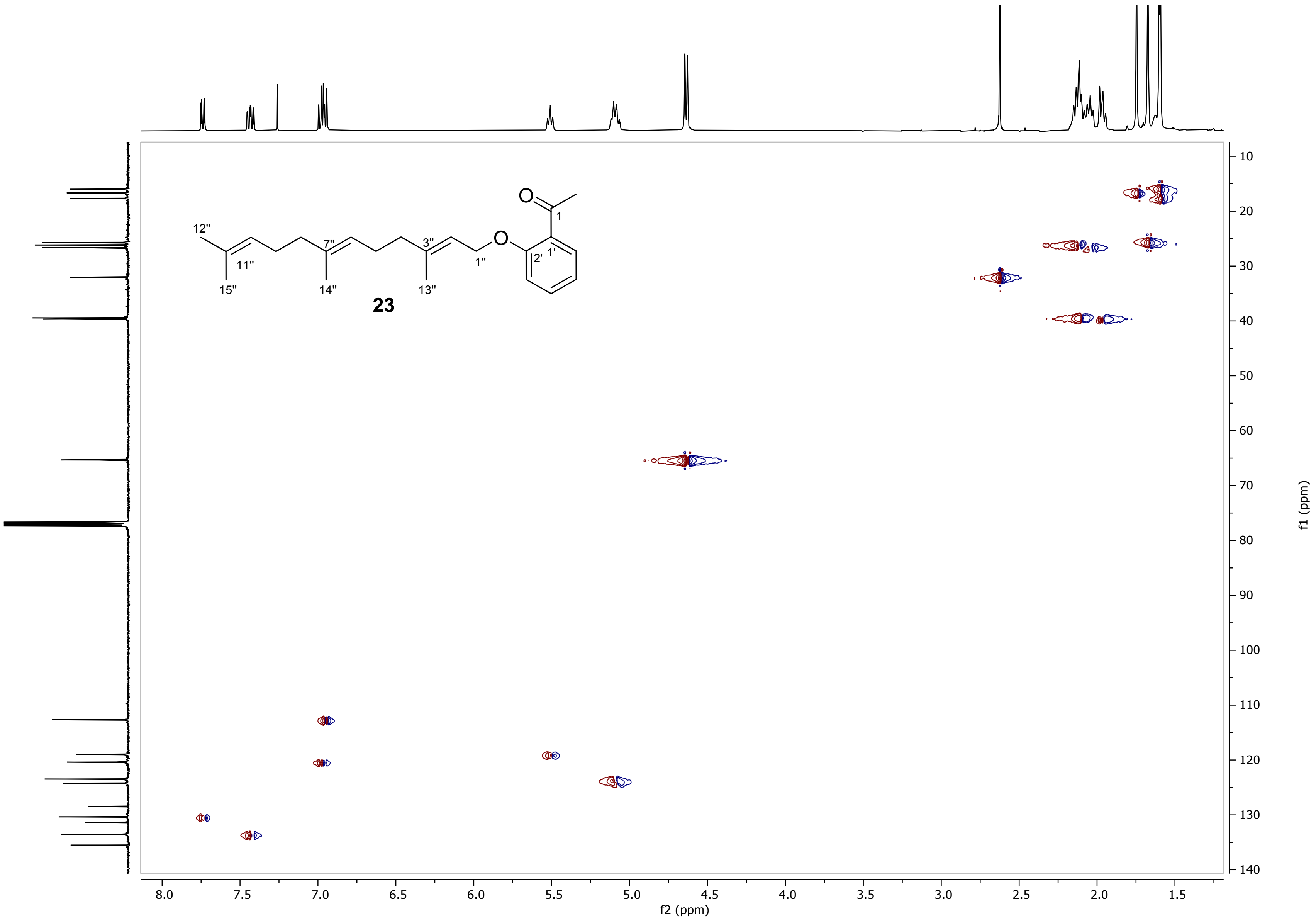

Figure S3d. gHSQC spectrum of 1-(2'-(((2"*E*,6"*E*)-3,7,11-trimethyldodeca-2,6,10-trien-1-yl)oxy)phenyl)ethanone (**23**) in $CDCl_3$.

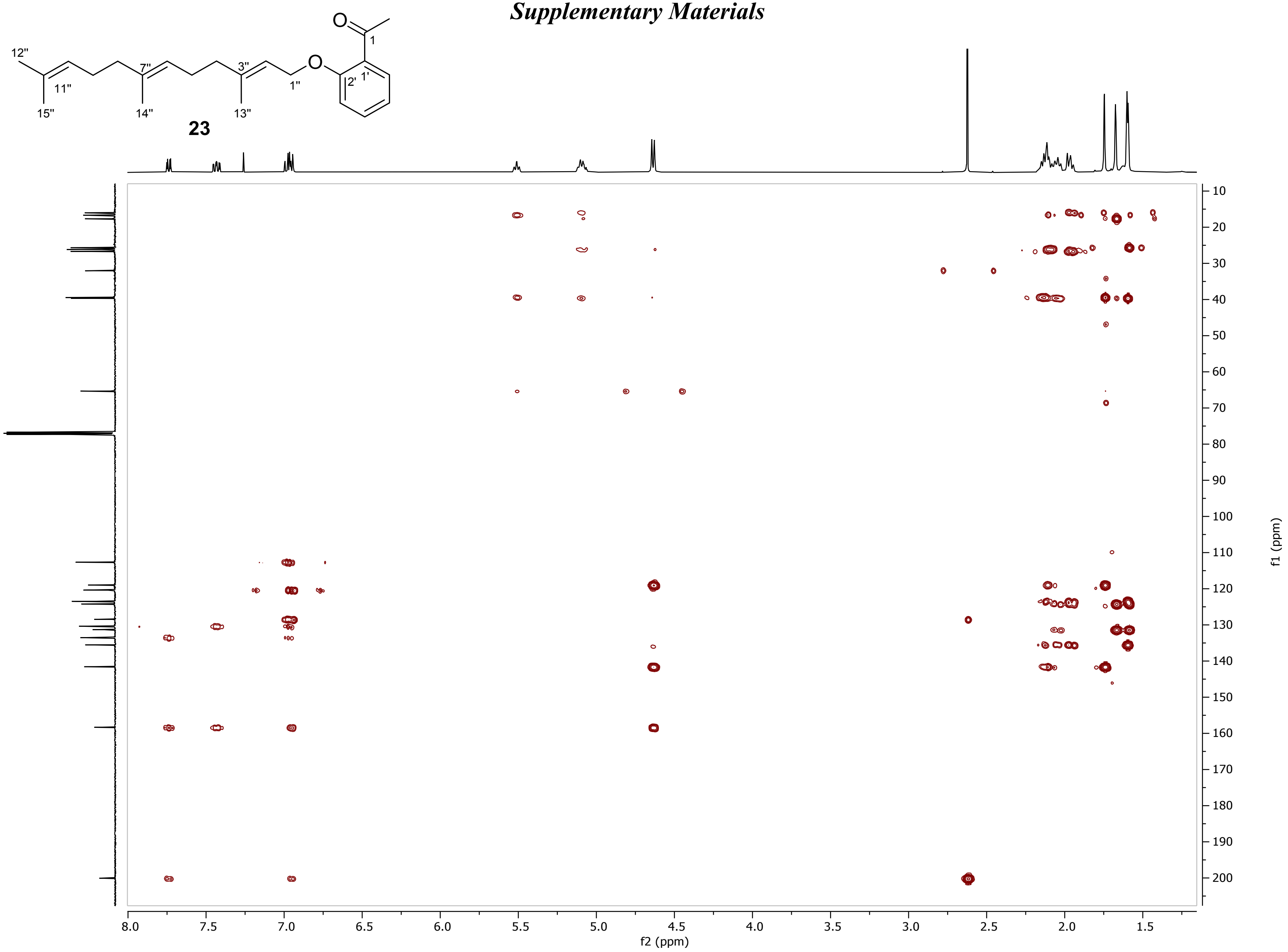

Figure S3e. gHMBC spectrum of 1-(2'-(((2"*E*,6"*E*)-3,7,11-trimethyldodeca-2,6,10-trien-1-yl)oxy)phenyl)ethanone (**23**) in $CDCl_3$.

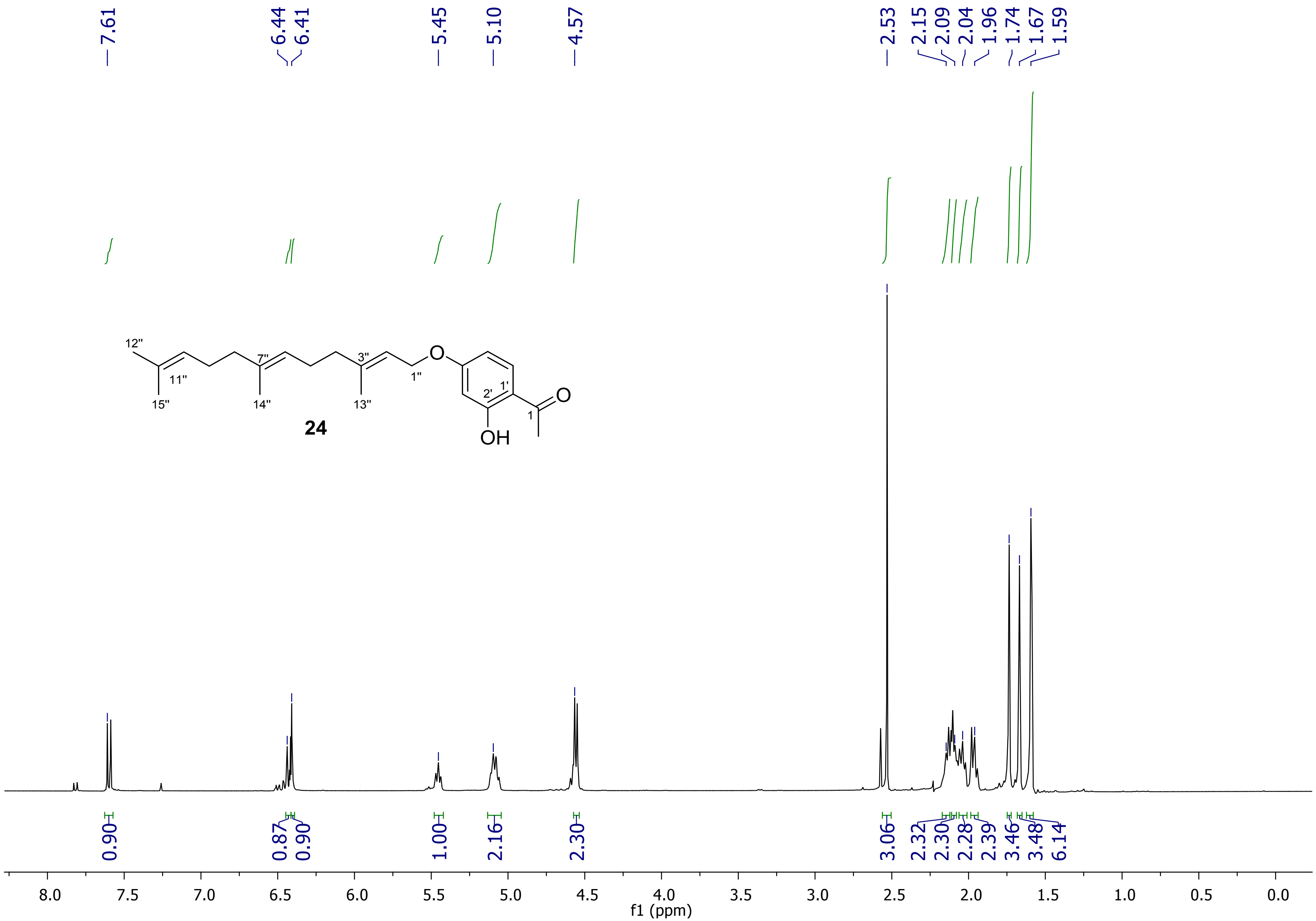

Figure S4a. ^1H NMR spectrum (400 MHz) of 1-(2'-hydroxy-4'-(((2''*E*,6''*E*)-3,7,11-trimethyldodeca-2,6,10-trien-1-yl)oxy)phenyl)ethanone (**24**) in $CDCl_3$.

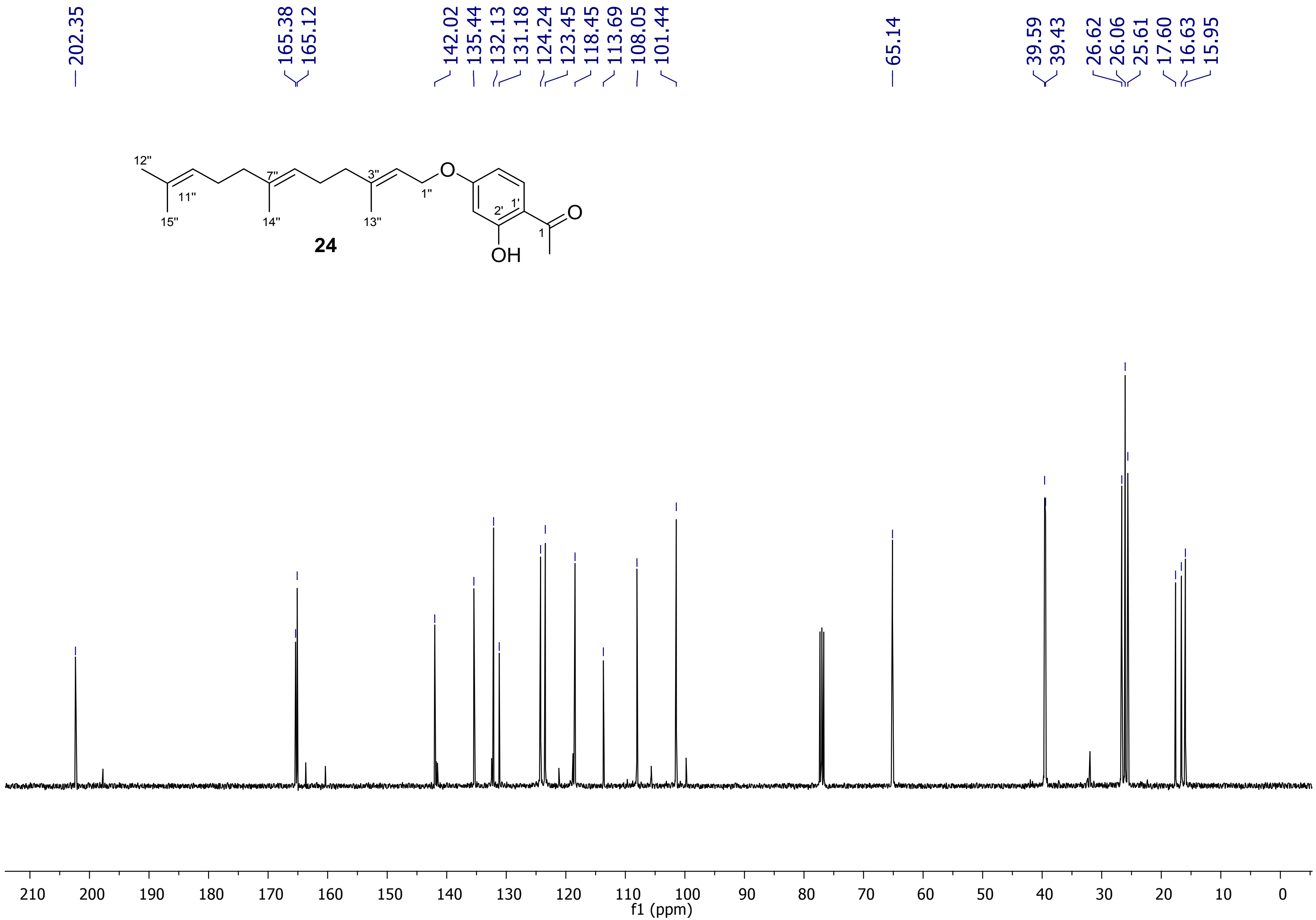

Figure S4b. ^{13}C NMR spectrum (100 MHz) of 1-(2’-hydroxy-4’-(((2"*E*,6"*E*)-3,7,11-trimethyldodeca-2,6,10-trien-1-yl)oxy)phenyl)ethanone (**24**) in $CDCl_3$.

Monoisotopic Mass, Even Electron Ions
127 formula(e) evaluated with 2 results within limits (up to 10 best isotopic matches for each mass)
Elements Used:
C: 1-500 H: 0-1000 O: 0-200 K: 0-1
AJM-118-1 223 (4.322)
3: TOF MS ES+

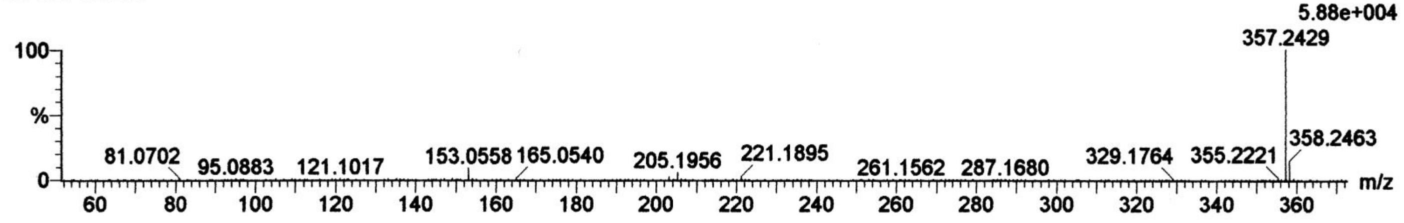

Mass	Calc. Mass	mDa	PPM	DBE	i-FIT	Norm	Conf(%)	Formula
Minimum:				-1.5				
Maximum:		5.0	10.0	50.0				
357.2429	357.2430	-0.1	-0.3	7.5	116.6	0.000	100.00	C23 H33 O3
	357.2407	2.2	6.2	-0.5	129.7	13.094	0.00	C18 H38 O4 K

Figure S4c. HRESIMS of 1-(2’-hydroxy-4’-(((2''*E*,6''*E*)-3,7,11-trimethyldodeca-2,6,10-trien-1-yl)oxy)phenyl)ethanone (**24**).

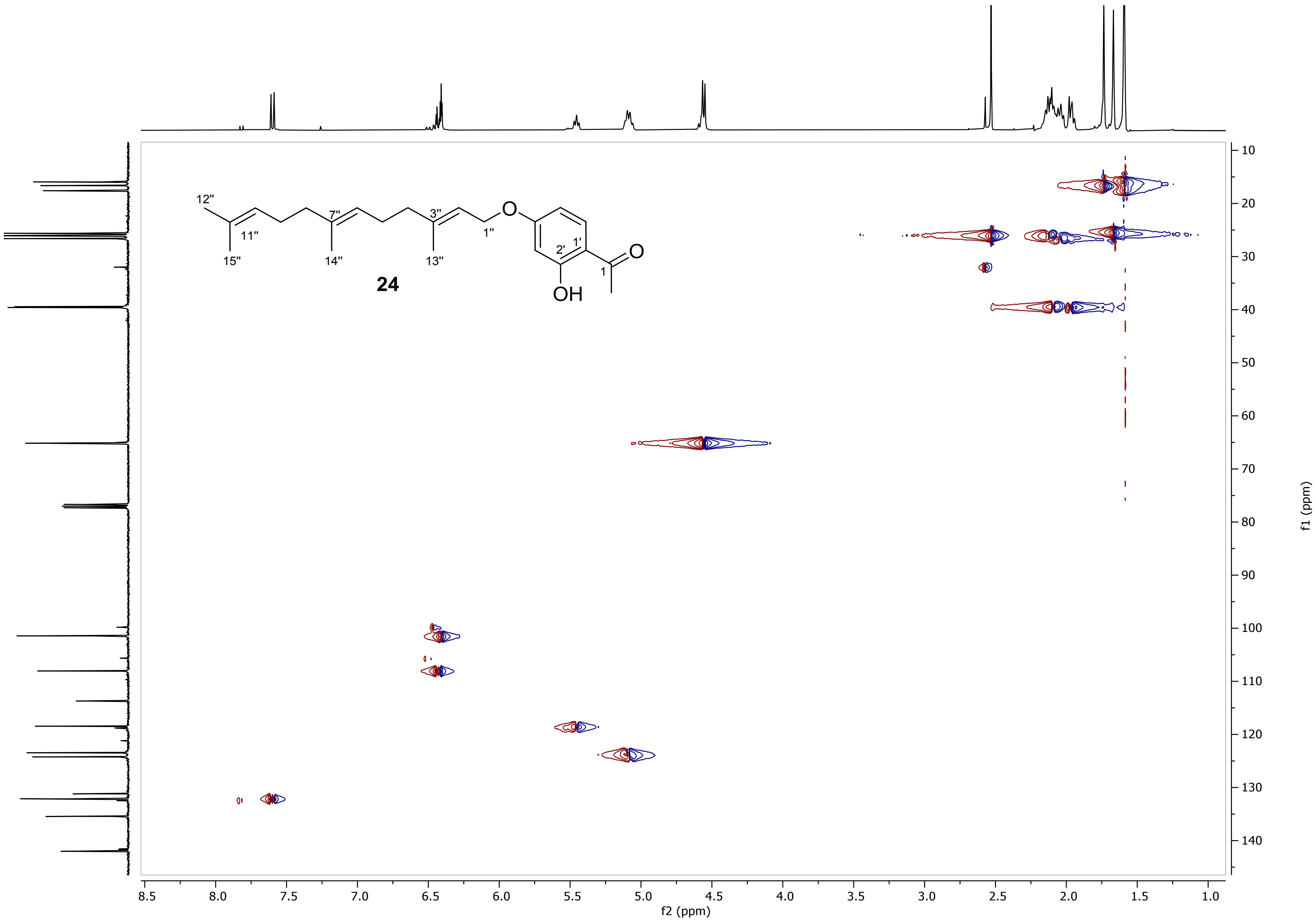

Figure S4d. gHSQC spectrum of 1-(2'-hydroxy-4'-(((2''*E*,6''*E*)-3,7,11-trimethyldodeca-2,6,10-trien-1-yl)oxy)phenyl)ethanone (**24**) in $CDCl_3$.

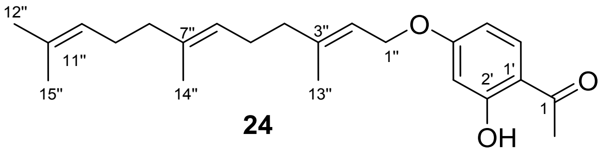

24

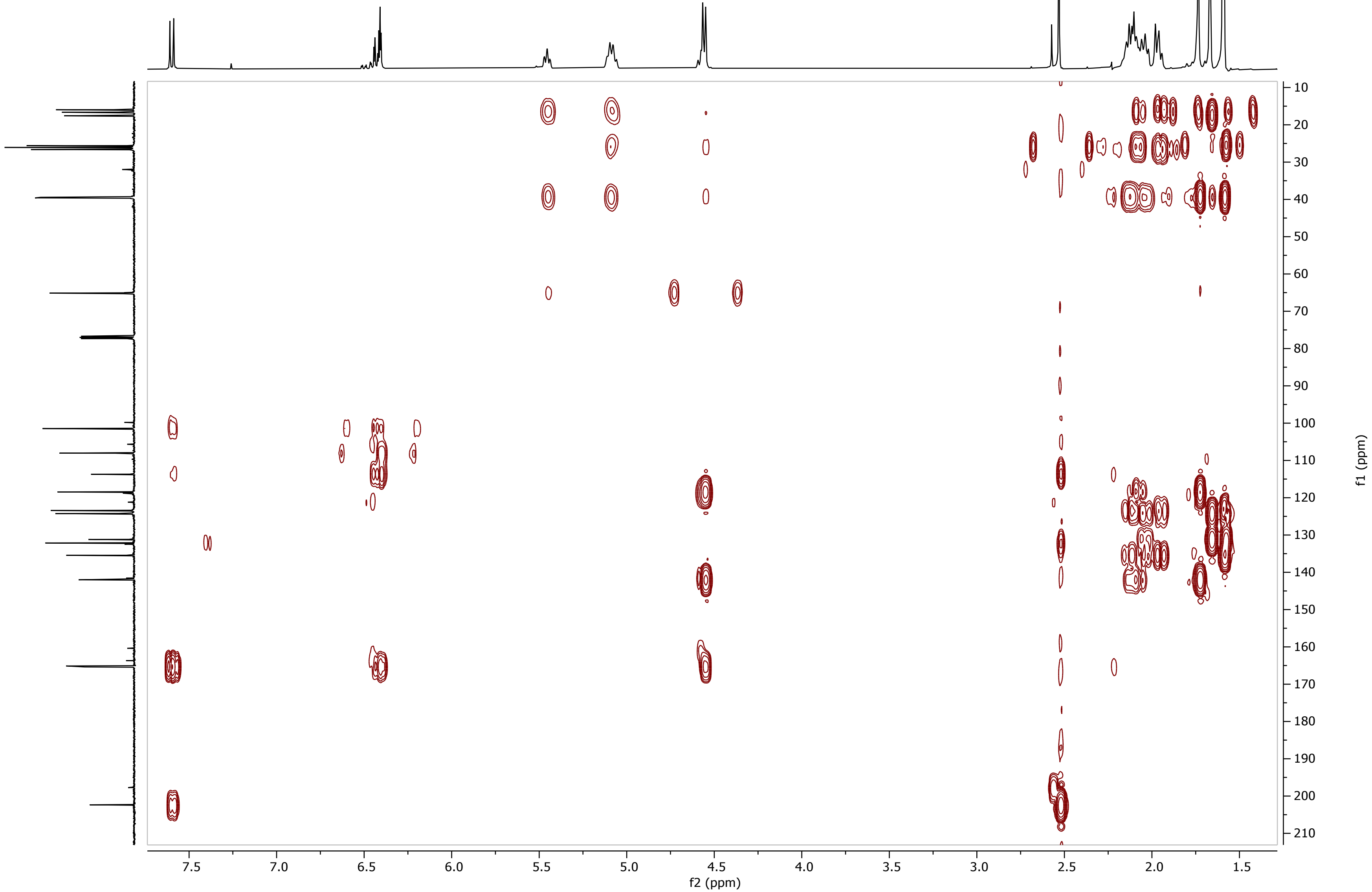

Figure S4e. gHMBC spectrum of 1-(2’-hydroxy-4’-(((2"*E*,6"*E*)-3,7,11-trimethyldodeca-2,6,10-trien-1-yl)oxy)phenyl)ethanone (**24**) in $CDCl_3$.

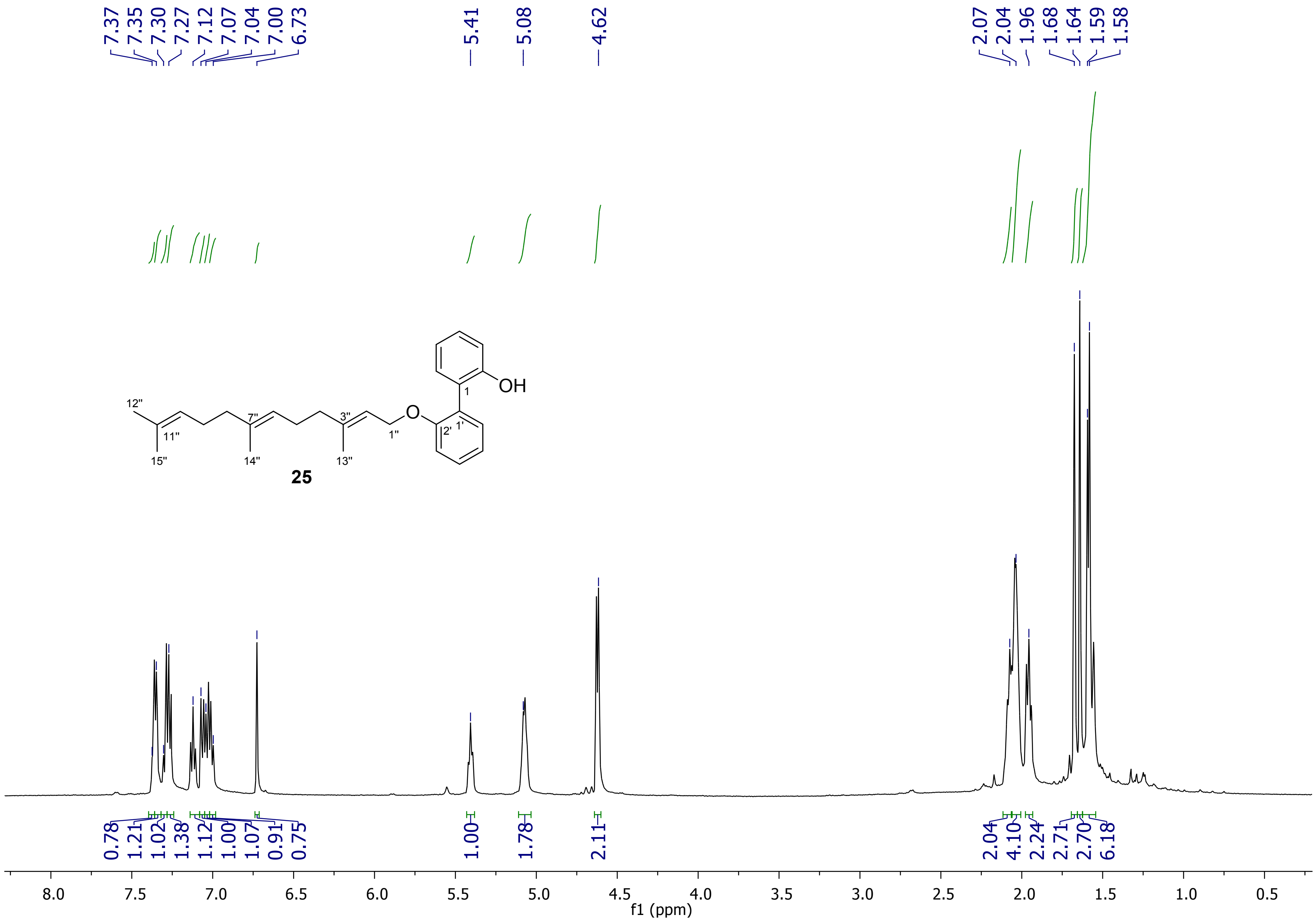

Figure S5a. ^{1}H NMR spectrum (400 MHz) of 2'-(((2''*E*,6''*E*)-3,7,11-trimethyldodeca-2,6,10-trien-1-yl)oxy)-[1,1'-biphenyl]-2-ol (**25**) in $CDCl_3$.

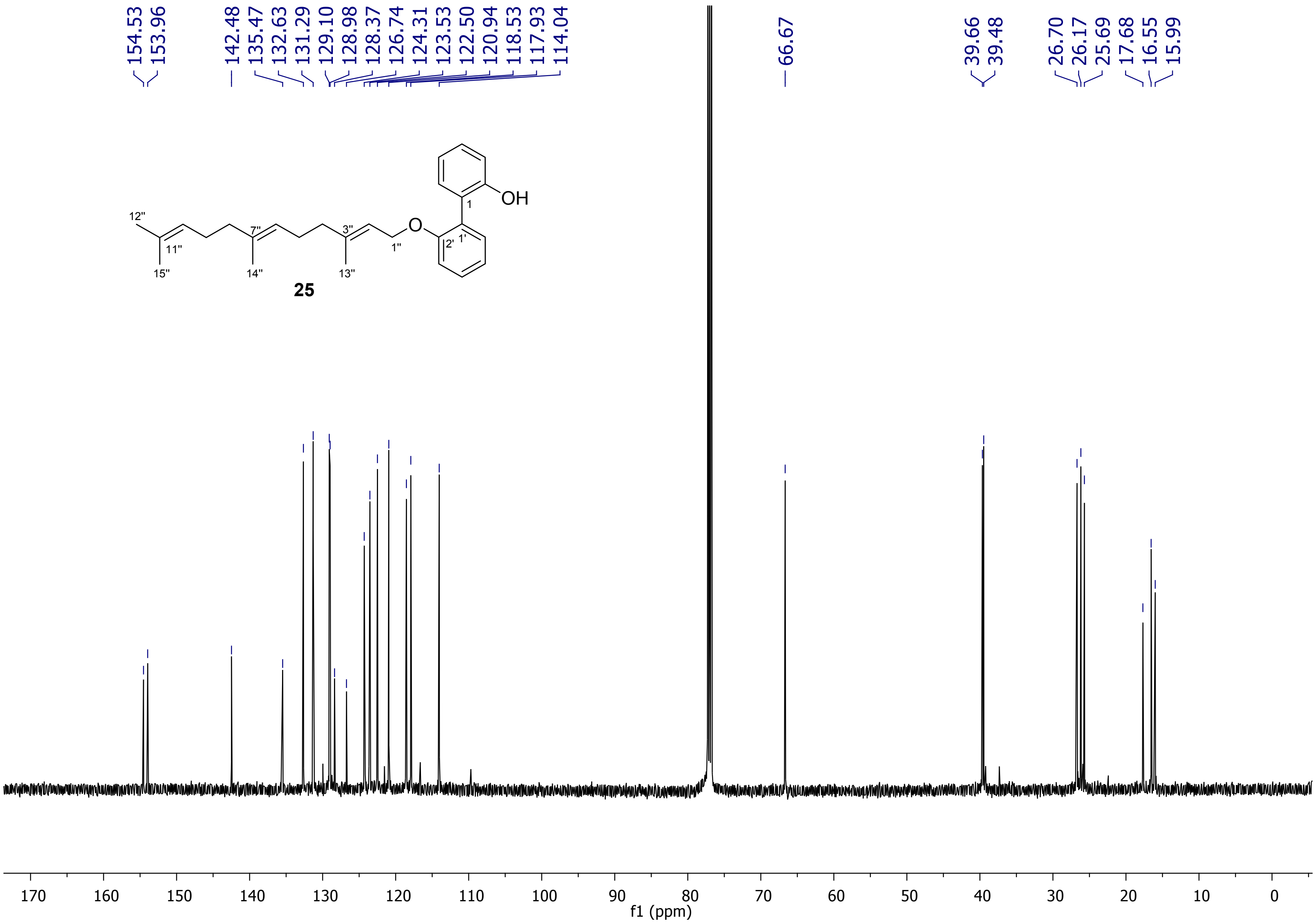

Figure S5b. ^{13}C NMR spectrum (100 MHz) of 2'-(((2"*E*,6"*E*)-3,7,11-trimethyldodeca-2,6,10-trien-1-yl)oxy)-[1,1'-biphenyl]-2-ol (**25**) in $CDCl_3$.

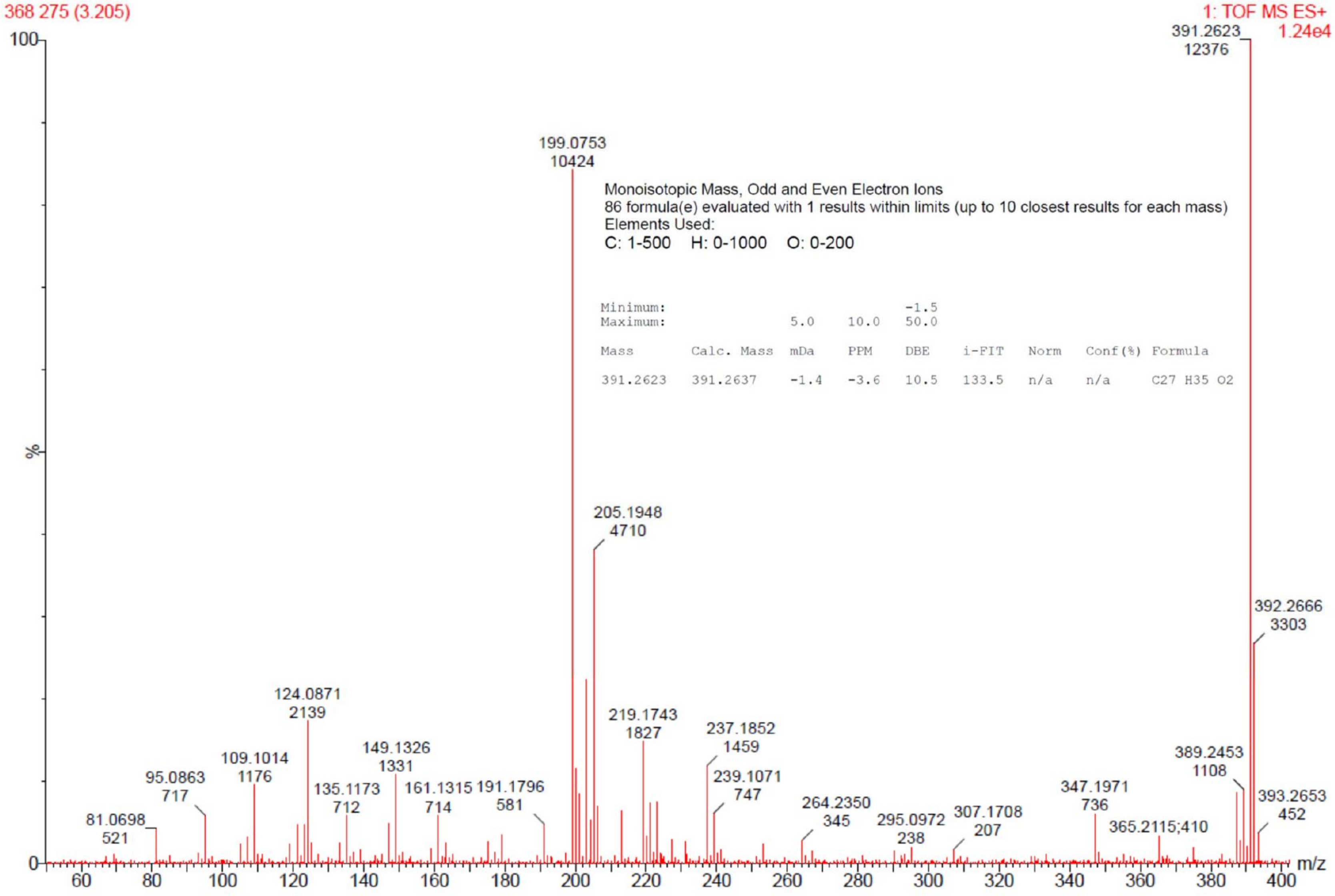

Figure S5c. HRESIMS of 2'-(((2*E*,6*E*)-3,7,11-trimethyldodeca-2,6,10-trien-1-yl)oxy)-[1,1'-biphenyl]-2-ol (**25**).

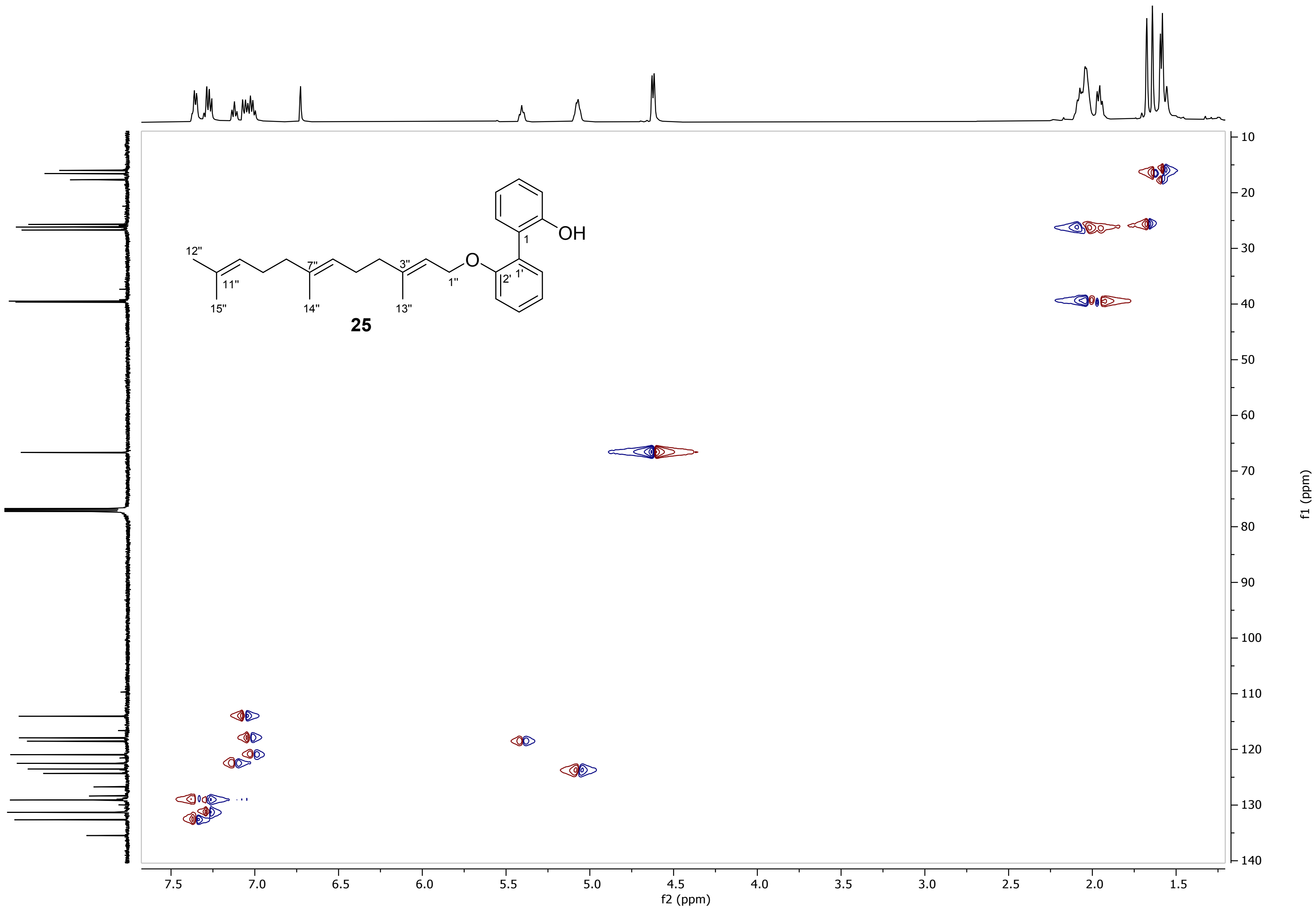

Figure S5d. gHSQC spectrum of 2'-(((2''*E*,6''*E*)-3,7,11-trimethyldodeca-2,6,10-trien-1-yl)oxy)-[1,1'-biphenyl]-2-ol (**25**) in $CDCl_3$.

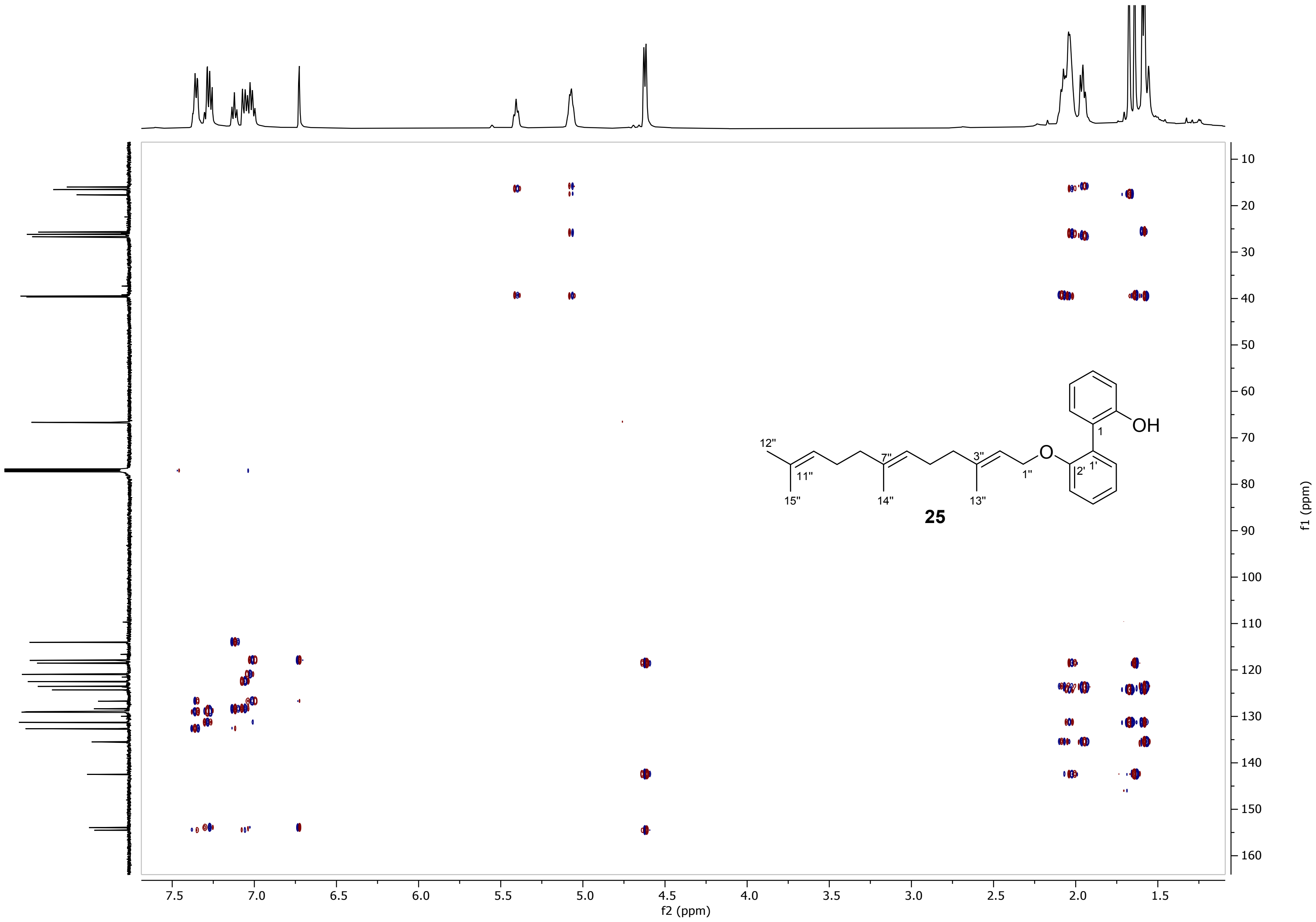

Figure S5e. gHMBC spectrum of 2'-(((2"*E*,6"*E*)-3,7,11-trimethyldodeca-2,6,10-trien-1-yl)oxy)-[1,1'-biphenyl]-2-ol (**25**) in $CDCl_3$.

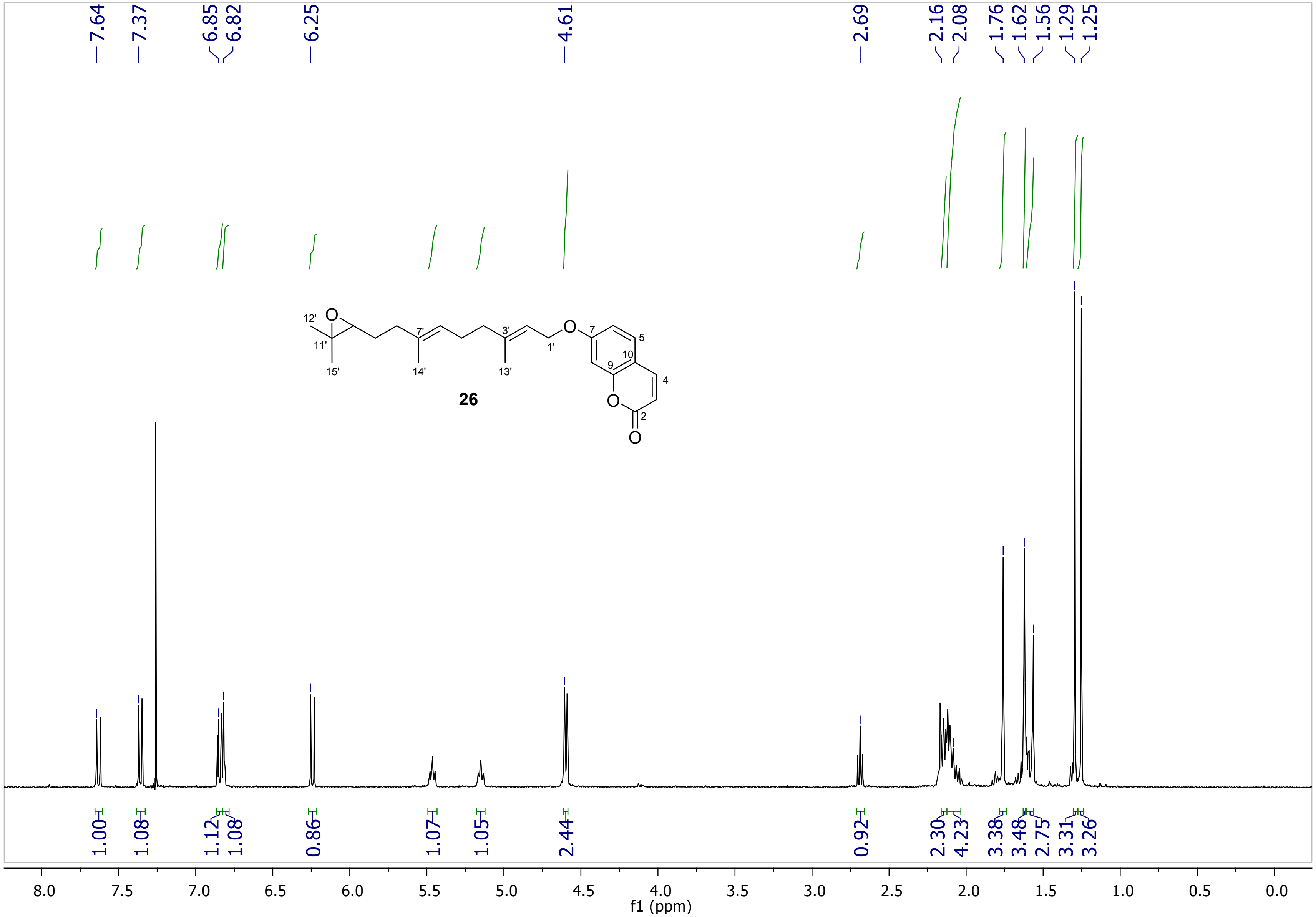

Figure S6a. ^{1}H NMR spectrum (400 MHz) of (±)-7-((2'*E*,6'*E*)-10,11-epoxy-3,7,11-trimethyldodeca-2,6-dien-1-yloxy)-2H-chromen-2-one ((±)-**26**) in $CDCl_3$.

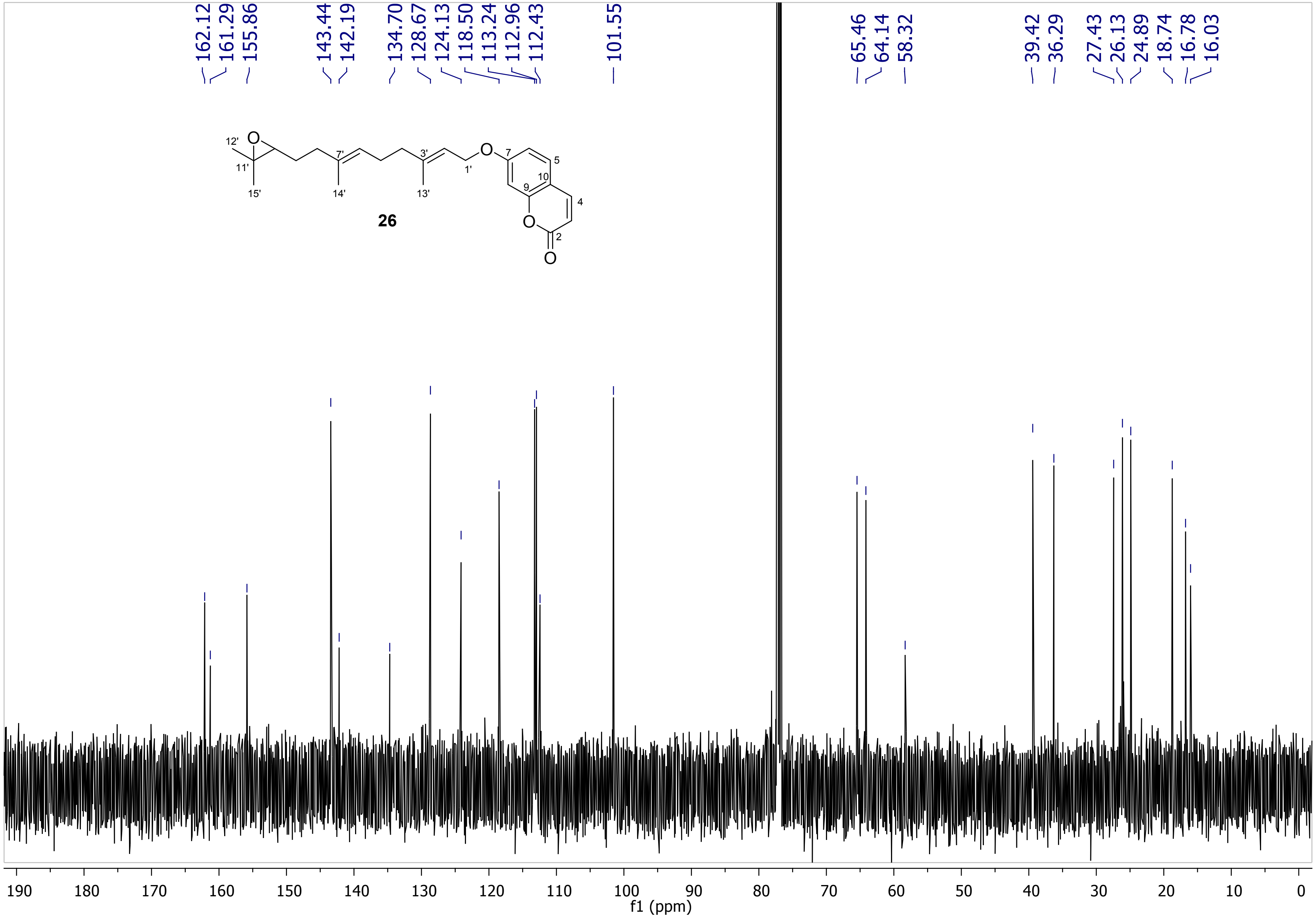

Figure S6b. ^{1}H NMR spectrum (100 MHz) of (±)-7-((2'*E*,6'*E*)-10,11-epoxy-3,7,11-trimethyldodeca-2,6-dien-1-yloxy)-2H-chromen-2-one ((±)-**26**) in $CDCl_3$.

Monoisotopic Mass, Even Electron Ions
163 formula(e) evaluated with 2 results within limits (up to 10 closest results for each mass)
Elements Used:
C: 1-500 H: 0-1000 O: 0-200 Na: 0-1
ARuan_Epo Far N 86 (1.676)
4: TOF MS ES+

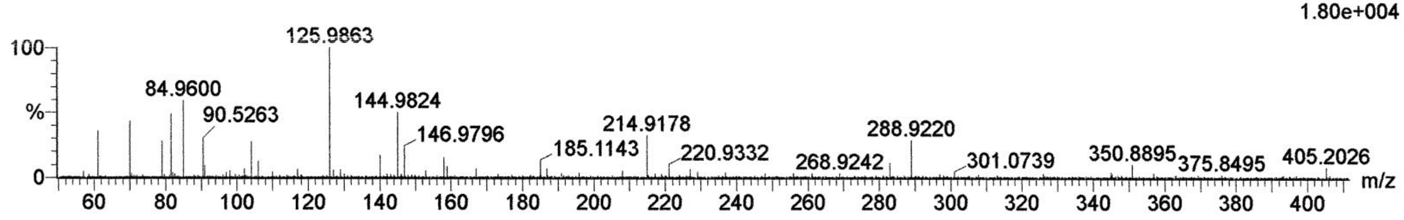

Minimum:				-1.5				
Maximum:		5.0	10.0	50.0				
Mass	Calc. Mass	mDa	PPM	DBE	i-FIT	Norm	Conf(%)	Formula
405.2026	405.2042	-1.6	-3.9	9.5	277.0	0.399	67.11	C24 H30 O4 Na
	405.2066	-4.0	-9.9	12.5	277.7	1.112	32.89	C26 H29 O4

Figure S6c. HRESIMS of (±)-7-((2’*E*,6’*E*)-10,11-epoxy-3,7,11-trimethyldodeca-2,6-dien-1-yloxy)-2H-chromen-2-one ((±)-**26**).

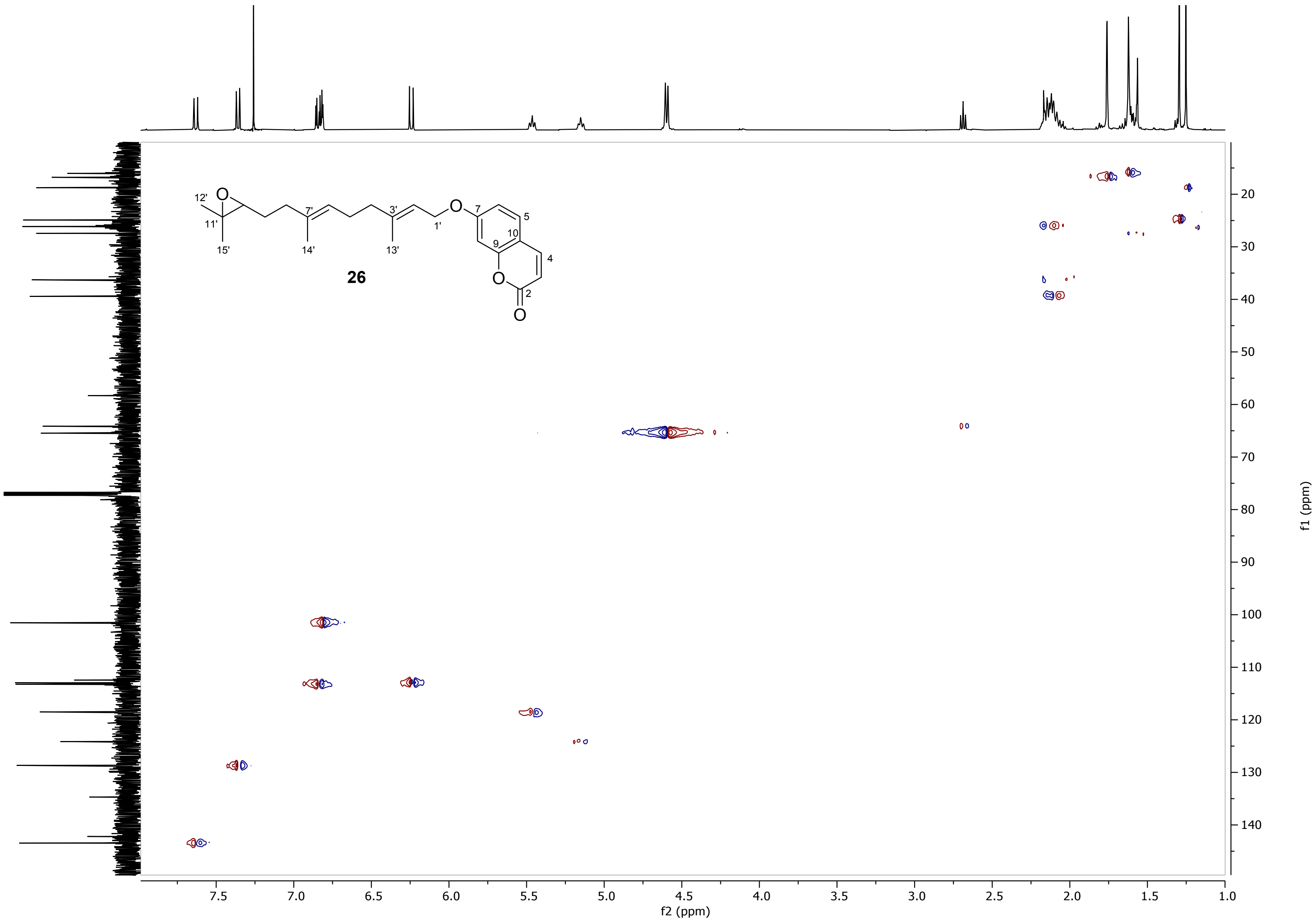

Figure S6d. gHSQC spectrum of (±)-7-((2'*E*,6'*E*)-10,11-epoxy-3,7,11-trimethyldodeca-2,6-dien-1-yloxy)-2H-chromen-2-one ((±)-**26**) in $CDCl_3$.

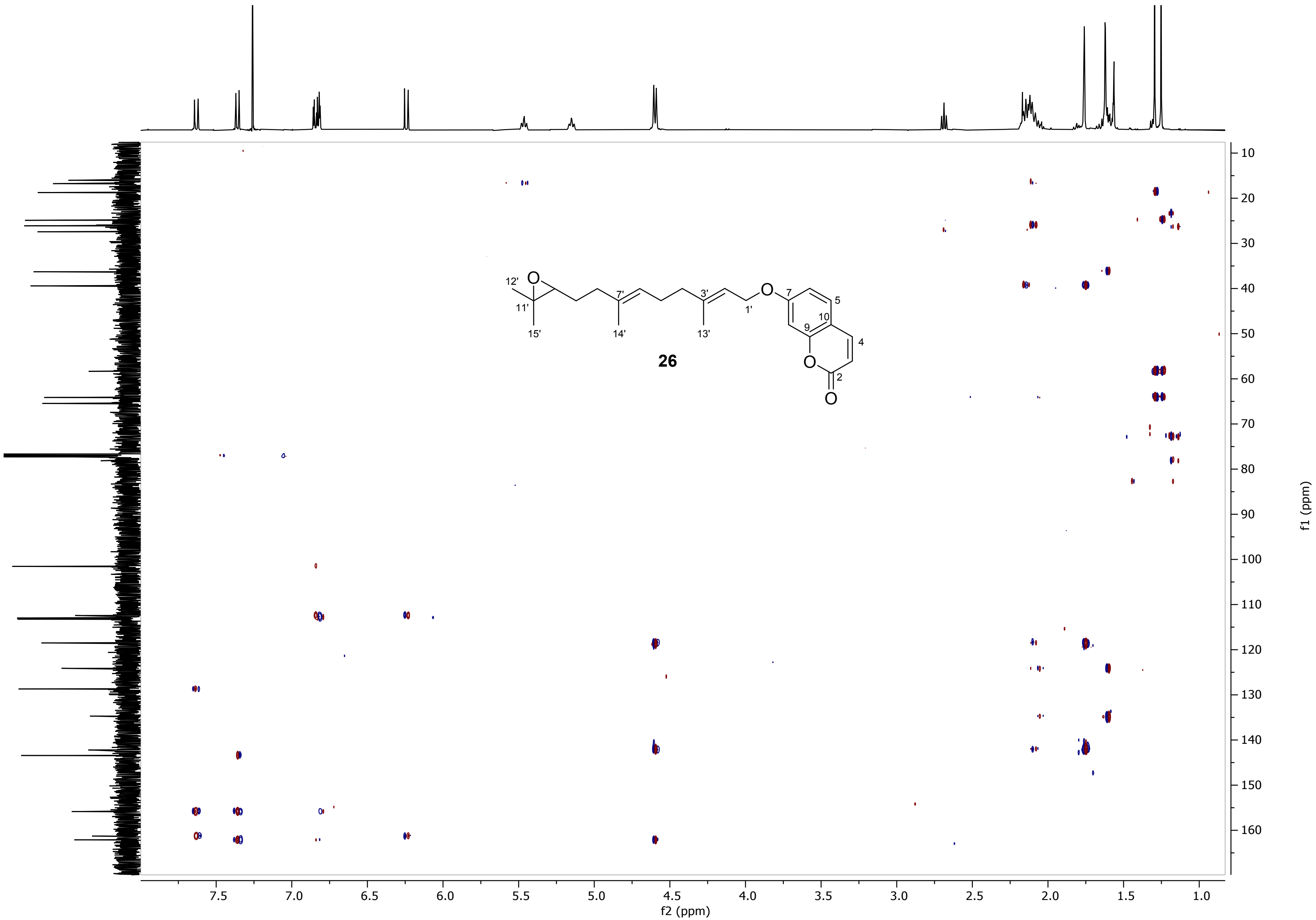

Figure S6e. gHMBC spectrum of (±)-7-((2'*E*,6'*E*)-10,11-epoxy-3,7,11-trimethyldodeca-2,6-dien-1-yloxy)-2H-chromen-2-one ((±)-**26**) in $CDCl_3$.

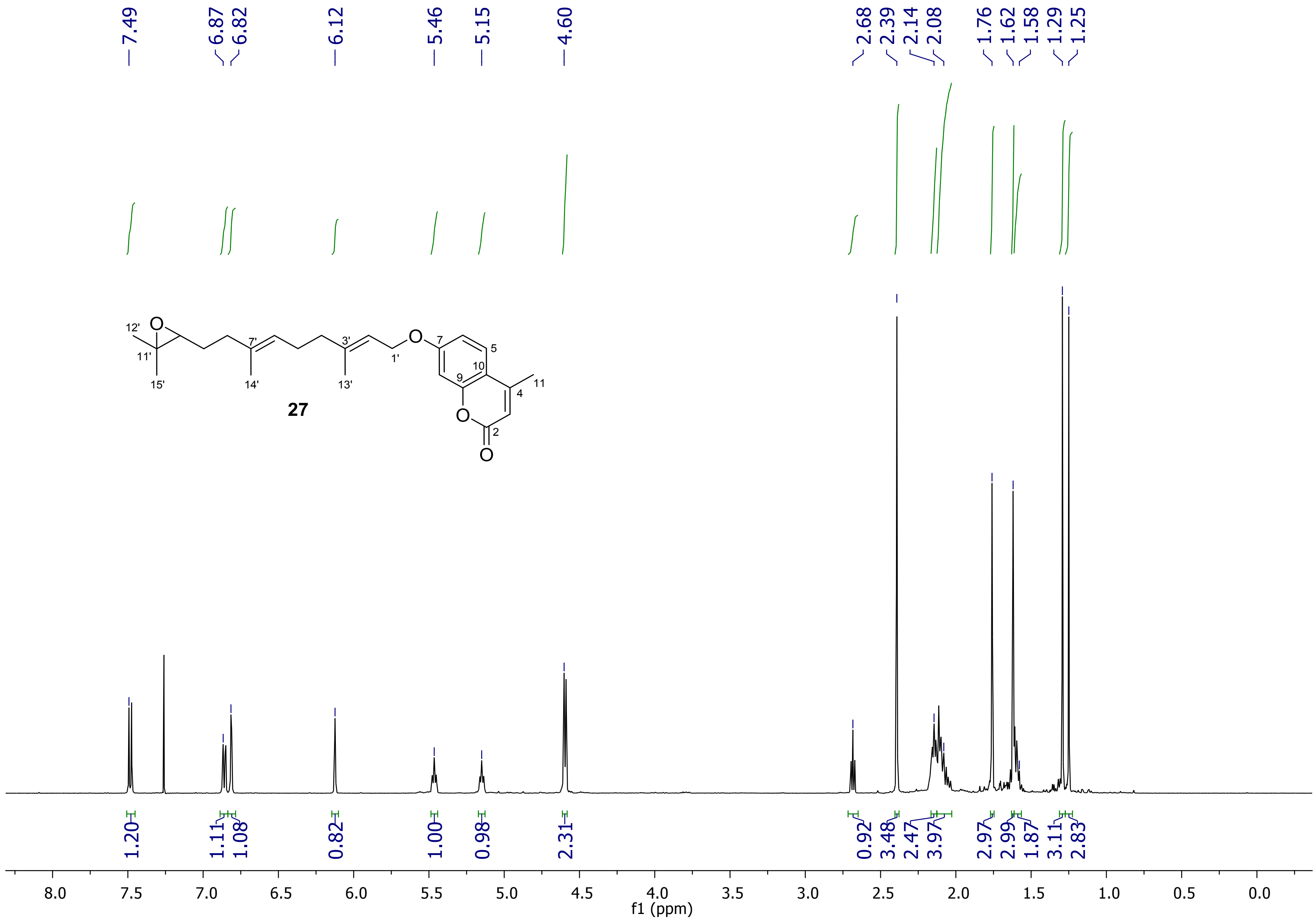

Figure S7a. ^{1}H NMR spectrum (500 MHz) of (±)-7-((2'*E*,6'*E*)-10,11-epoxy-3,7,11-trimethyldodeca-2,6-dien-1-yloxy)-4-methyl-2H-chromen-2-one ((±)-**27**) in $CDCl_3$.

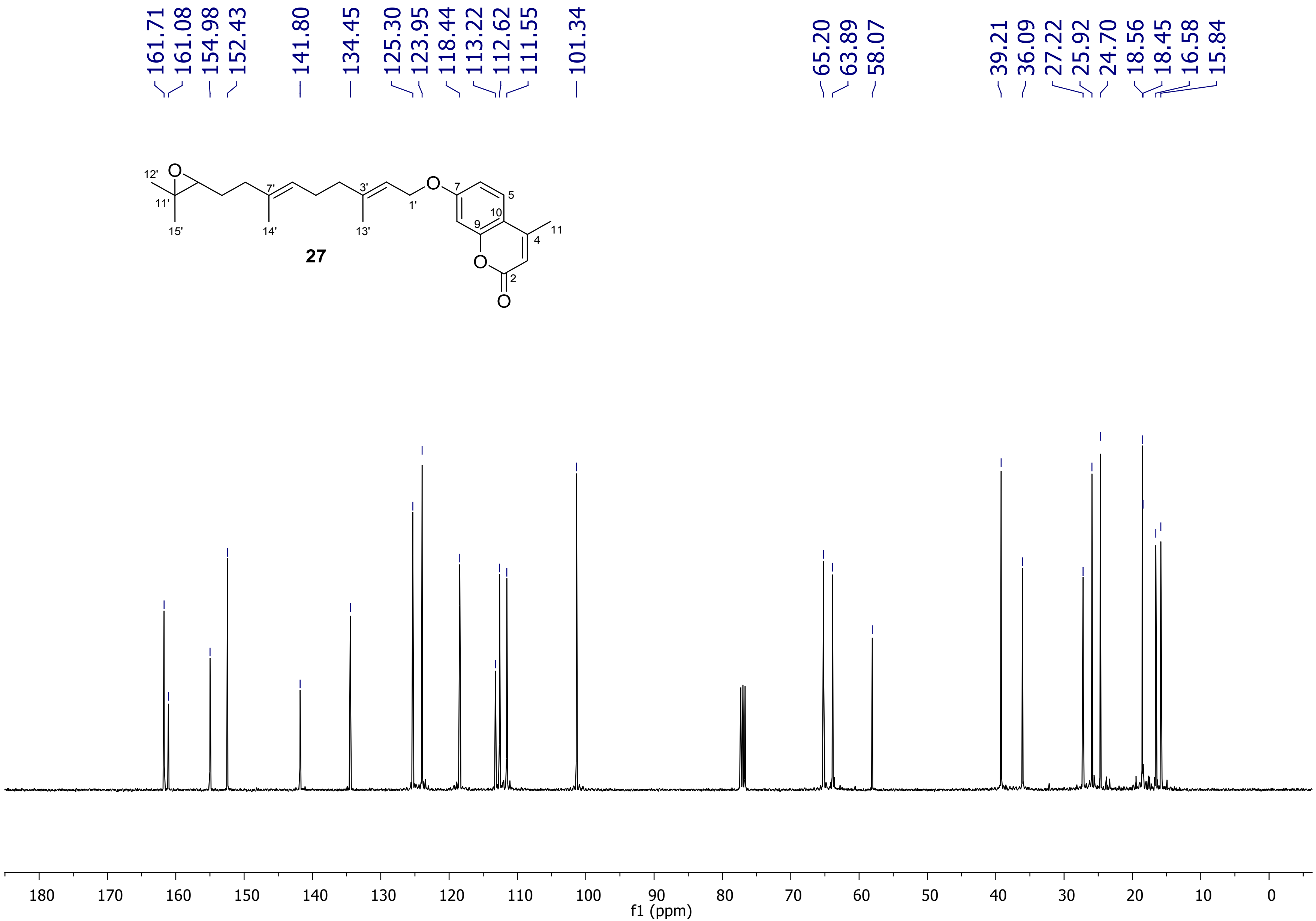

Figure S7b. ^{13}C NMR spectrum (100 MHz) of (±)-7-((2'*E*,6'*E*)-10,11-epoxy-3,7,11-trimethyldodeca-2,6-dien-1-yloxy)-4-methyl-2H-chromen-2-one ((±)-**27**) in $CDCl_3$.

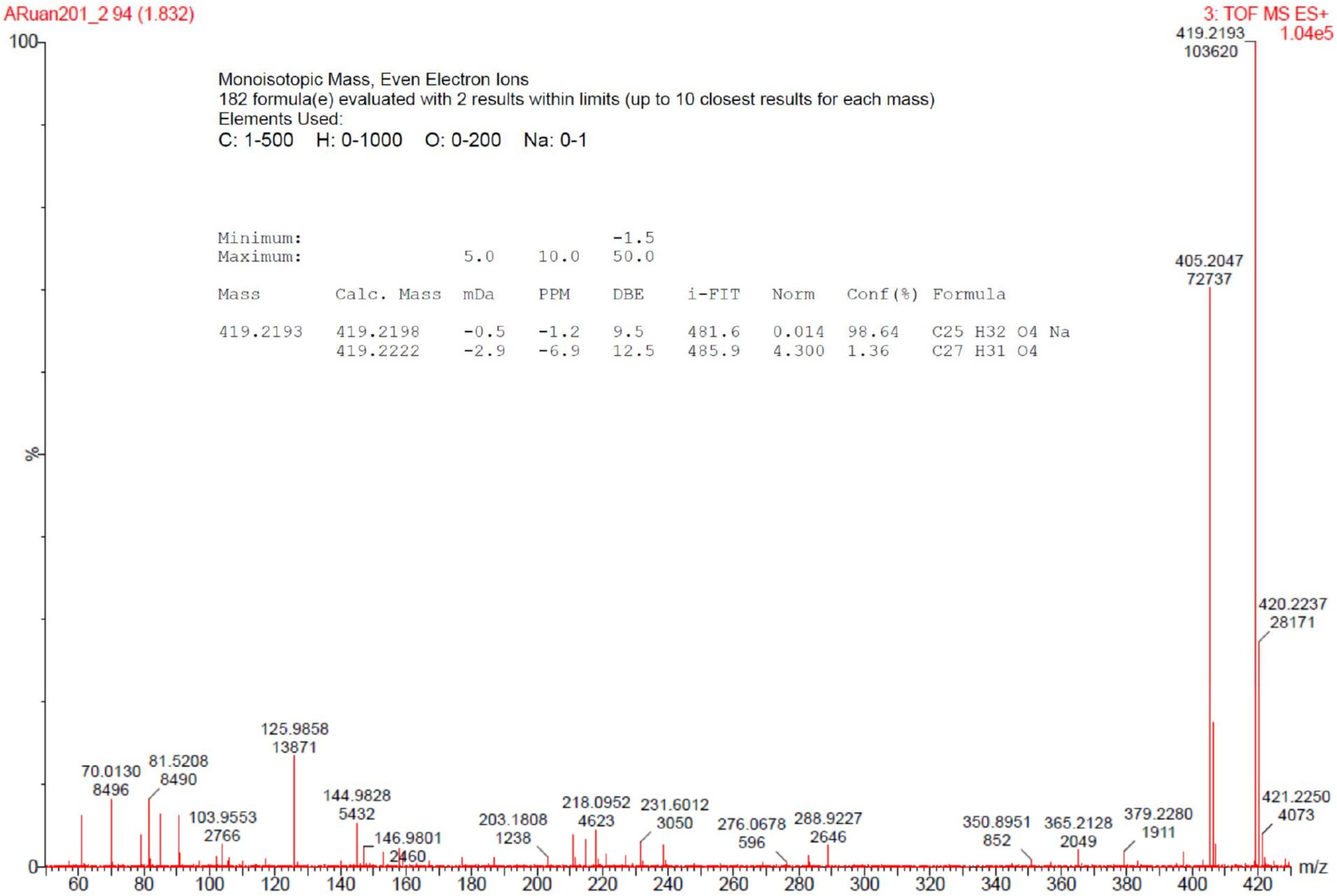

Figure S7c. HRESIMS of (±)-7-((2’*E*,6’*E*)-10,11-epoxy-3,7,11-trimethyldodeca-2,6-dien-1-yloxy)-4-methyl-2H-chromen-2-one ((±)-**27**).

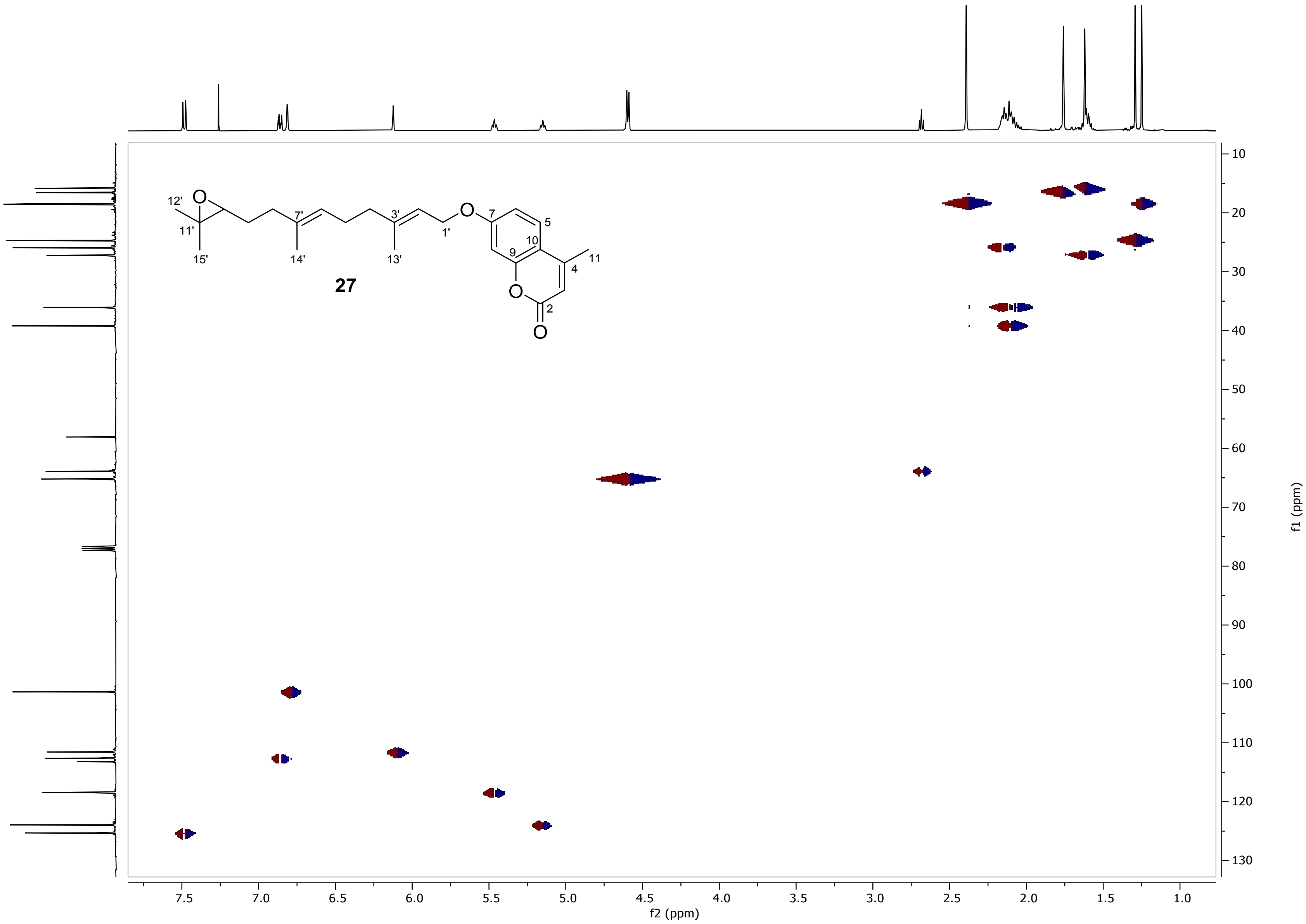

Figure S7d. gHSQC spectrum of (±)-7-((2'*E*,6'*E*)-10,11-epoxy-3,7,11-trimethyldodeca-2,6-dien-1-yloxy)-4-methyl-2H-chromen-2-one ((±)-**27**) in $CDCl_3$.

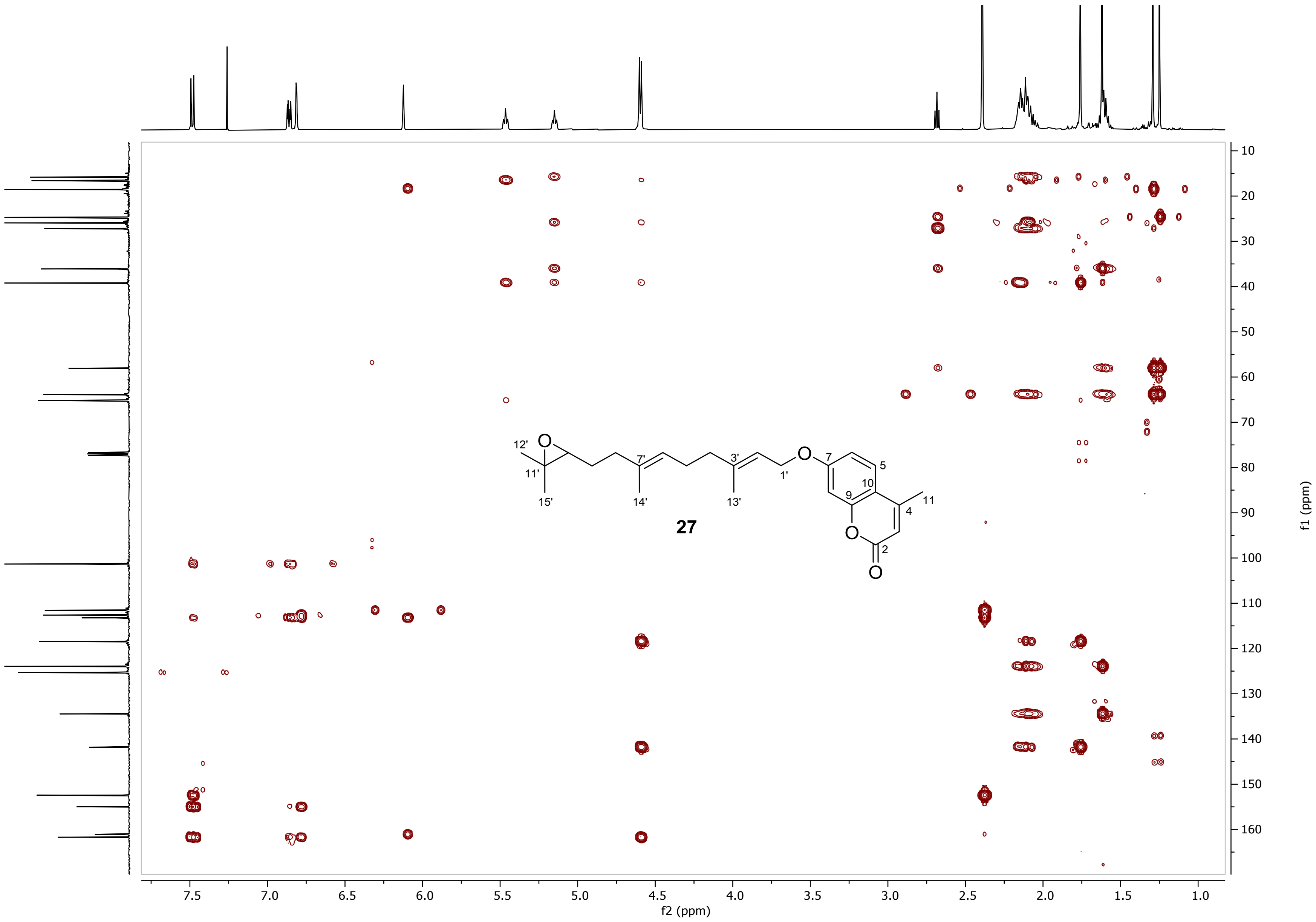

Figure S7e. gHMBC spectrum of (±)-7-((2’*E*,6’*E*)-10,11-epoxy-3,7,11-trimethyldodeca-2,6-dien-1-yloxy)-4-methyl-2H-chromen-2-one ((±)-**27**) in $CDCl_3$.

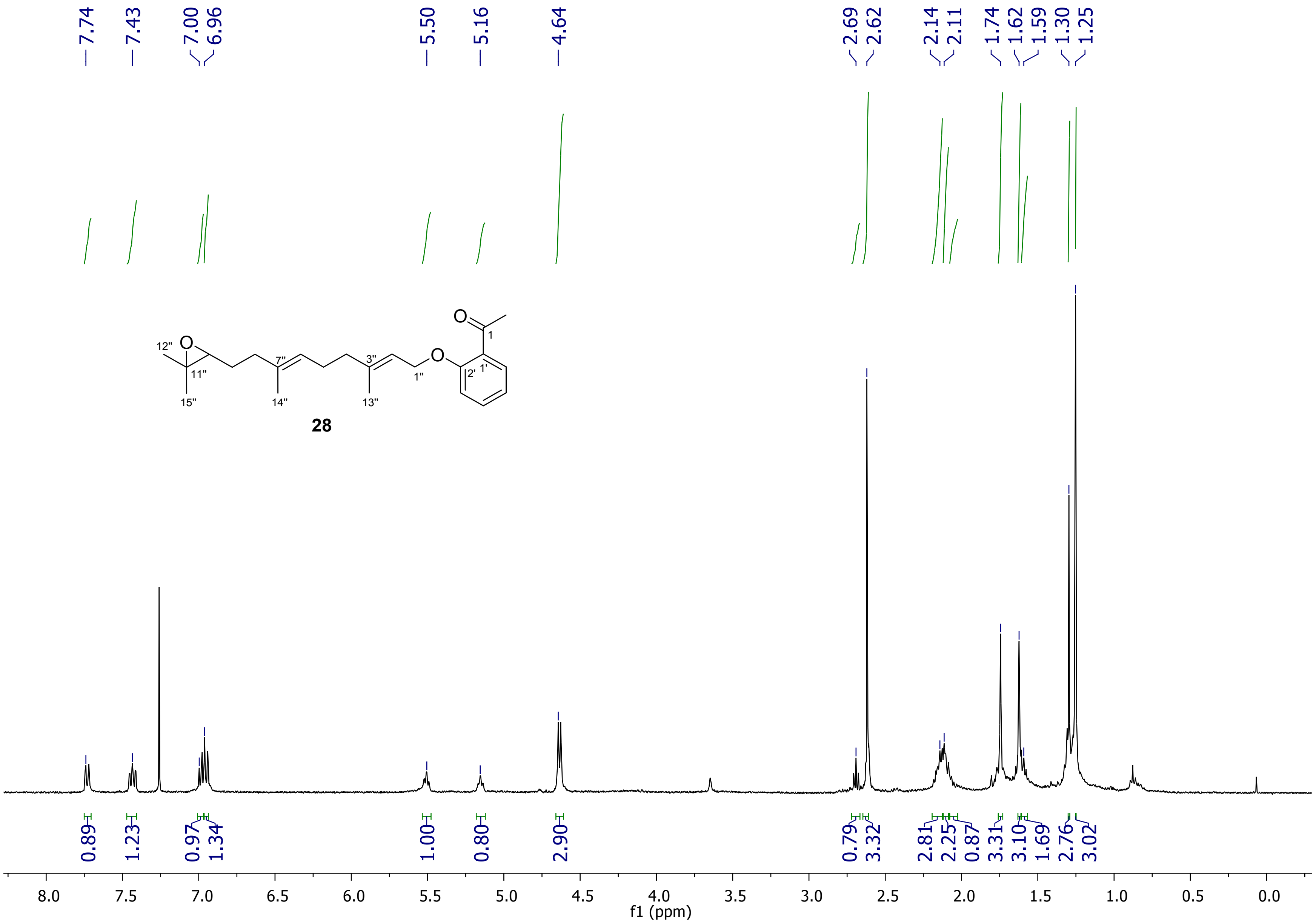

Figure S8a. ^{1}H NMR spectrum (400 MHz) of (±)-1-(2'-((2"*E*,6"*E*)-10,11-epoxy-3,7,11-trimethyldodeca-2,6-dien-1-yloxy)phenyl)ethanone ((±)-**28**) in $CDCl_3$.

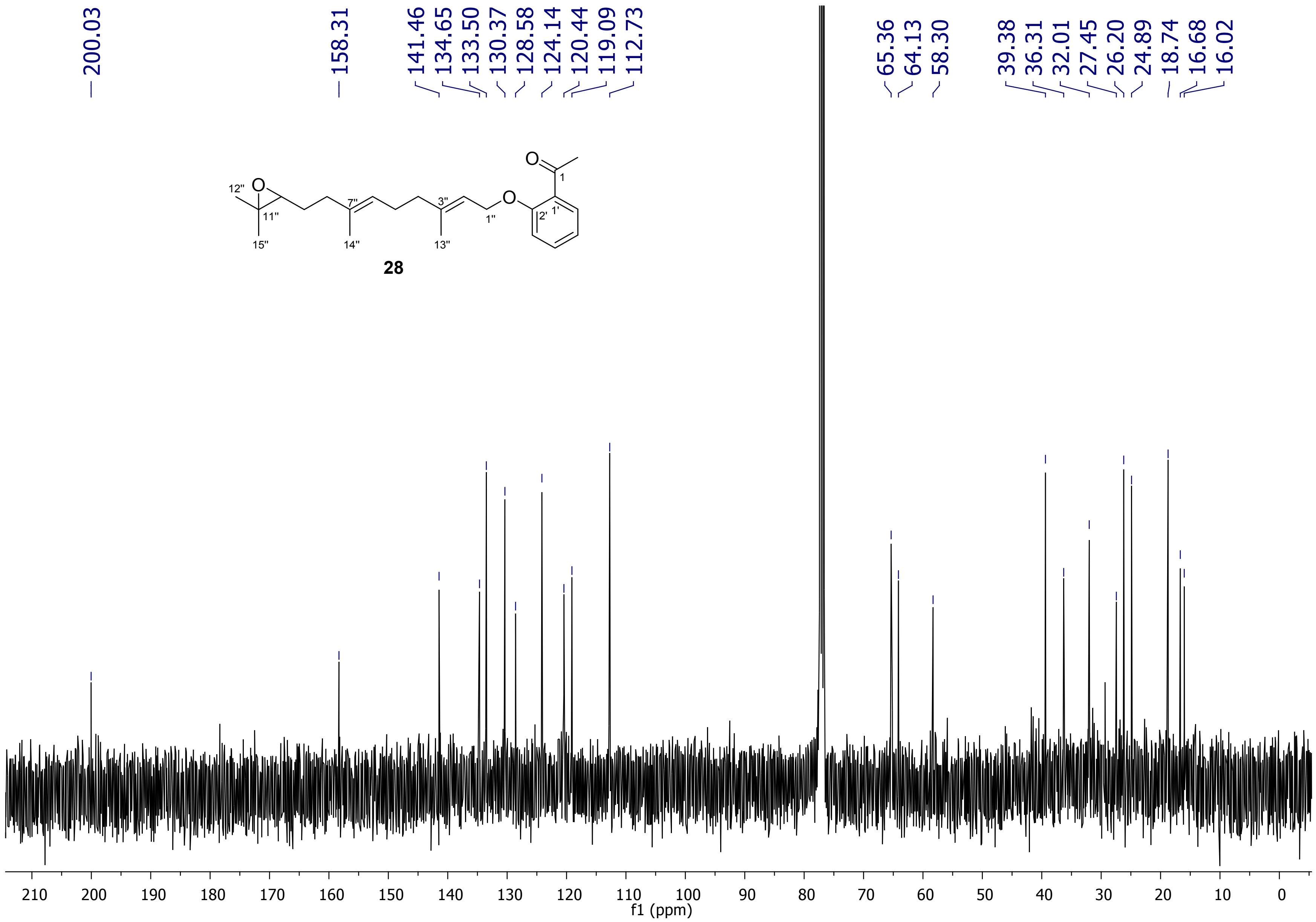

Figure S8b. ^{13}C NMR spectrum (100 MHz) of (±)-1-(2'-((2''*E*,6''*E*)-10,11-epoxy-3,7,11-trimethyldodeca-2,6-dien-1-yloxy)phenyl)ethanone ((±)-**28**) in $CDCl_3$.

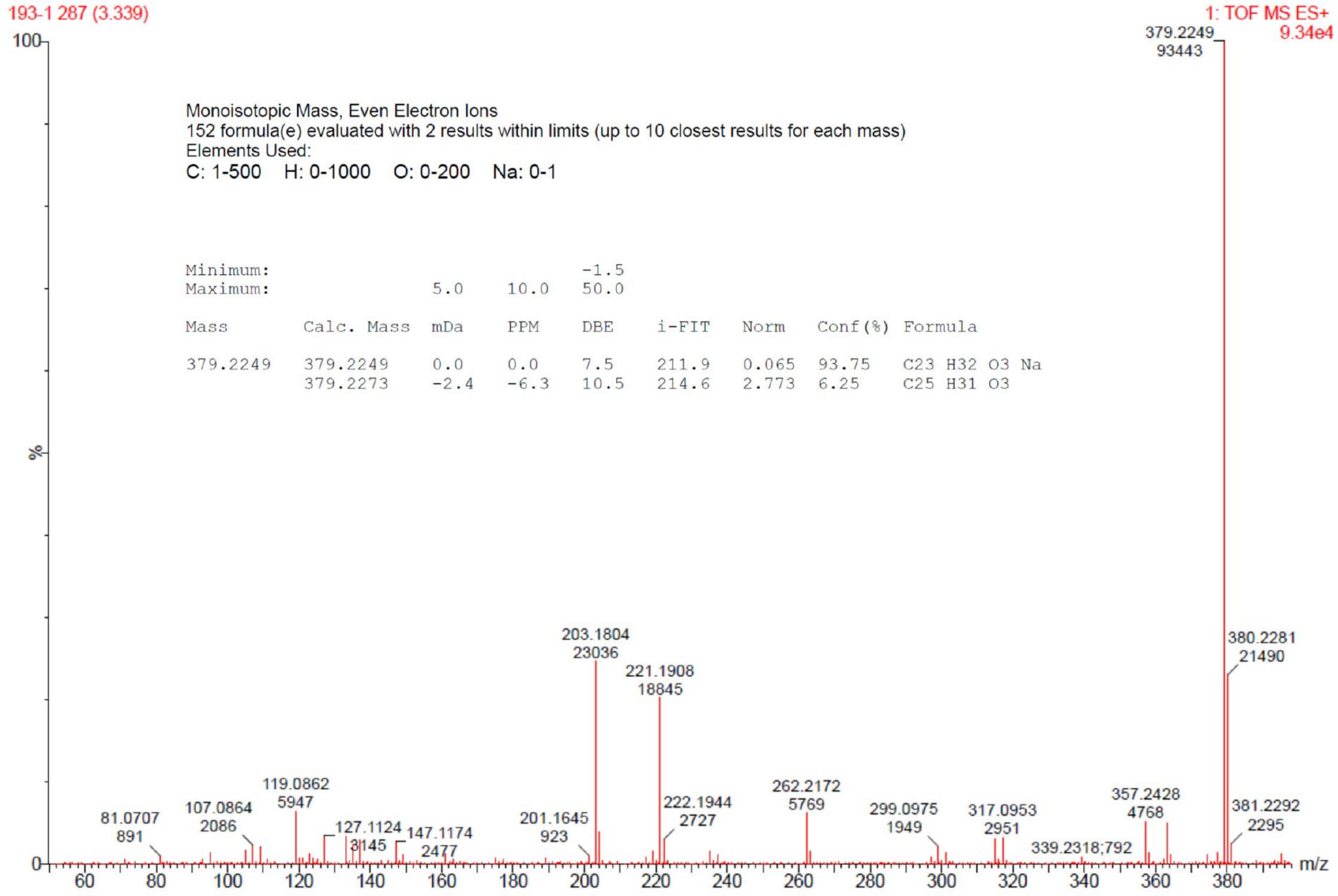

Figure S8c. HRESIMS of (±)-1-(2’-((2"*E*,6"*E*)-10,11-epoxy-3,7,11-trimethyldodeca-2,6-dien-1-yloxy)phenyl)ethanone ((±)-**28**).

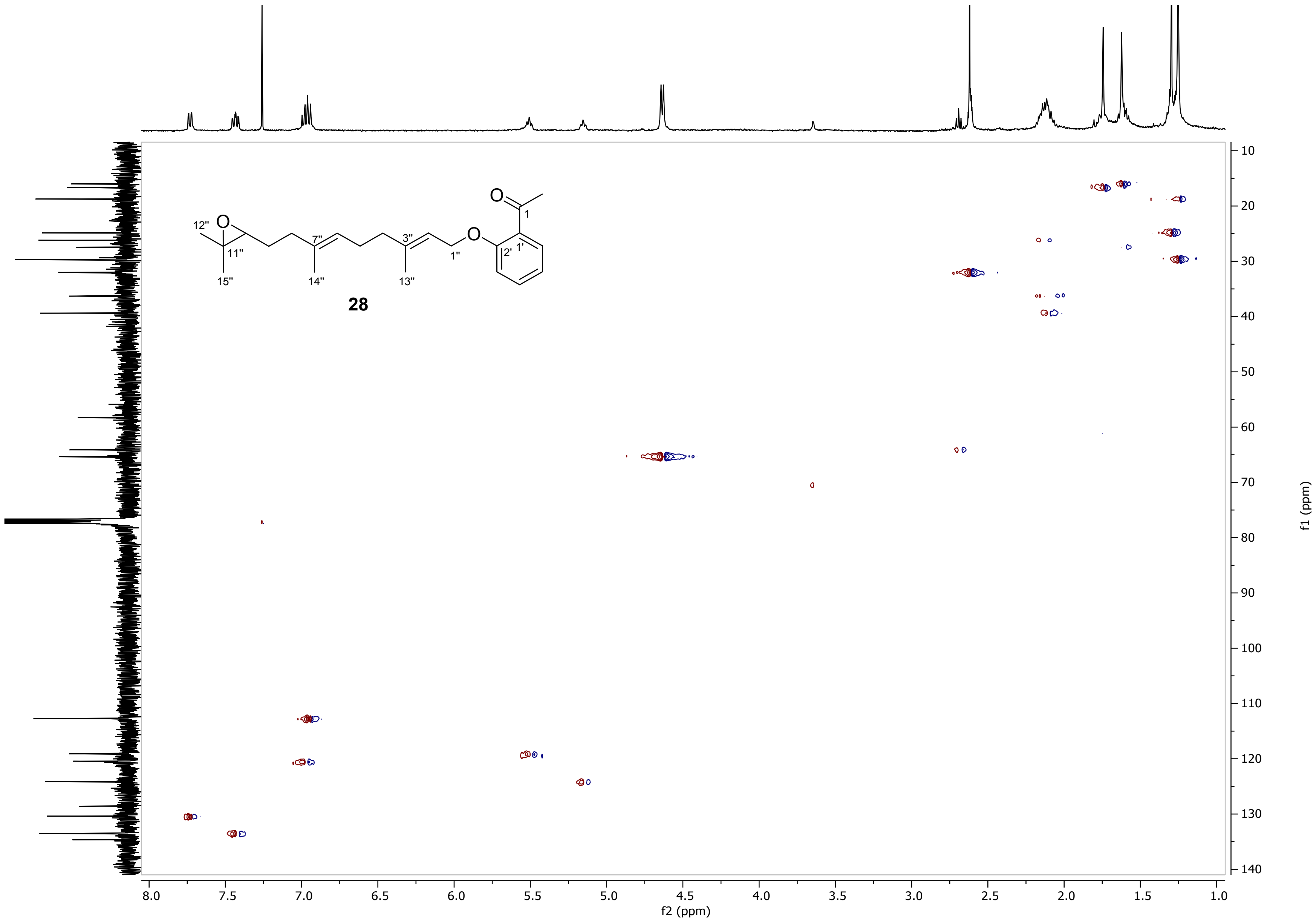

Figure S8d. gHSQC spectrum of (±)-1-(2'-((2"*E*,6"*E*)-10,11-epoxy-3,7,11-trimethyldodeca-2,6-dien-1-yloxy)phenyl)ethanone ((±)-**28**) in $CDCl_3$.

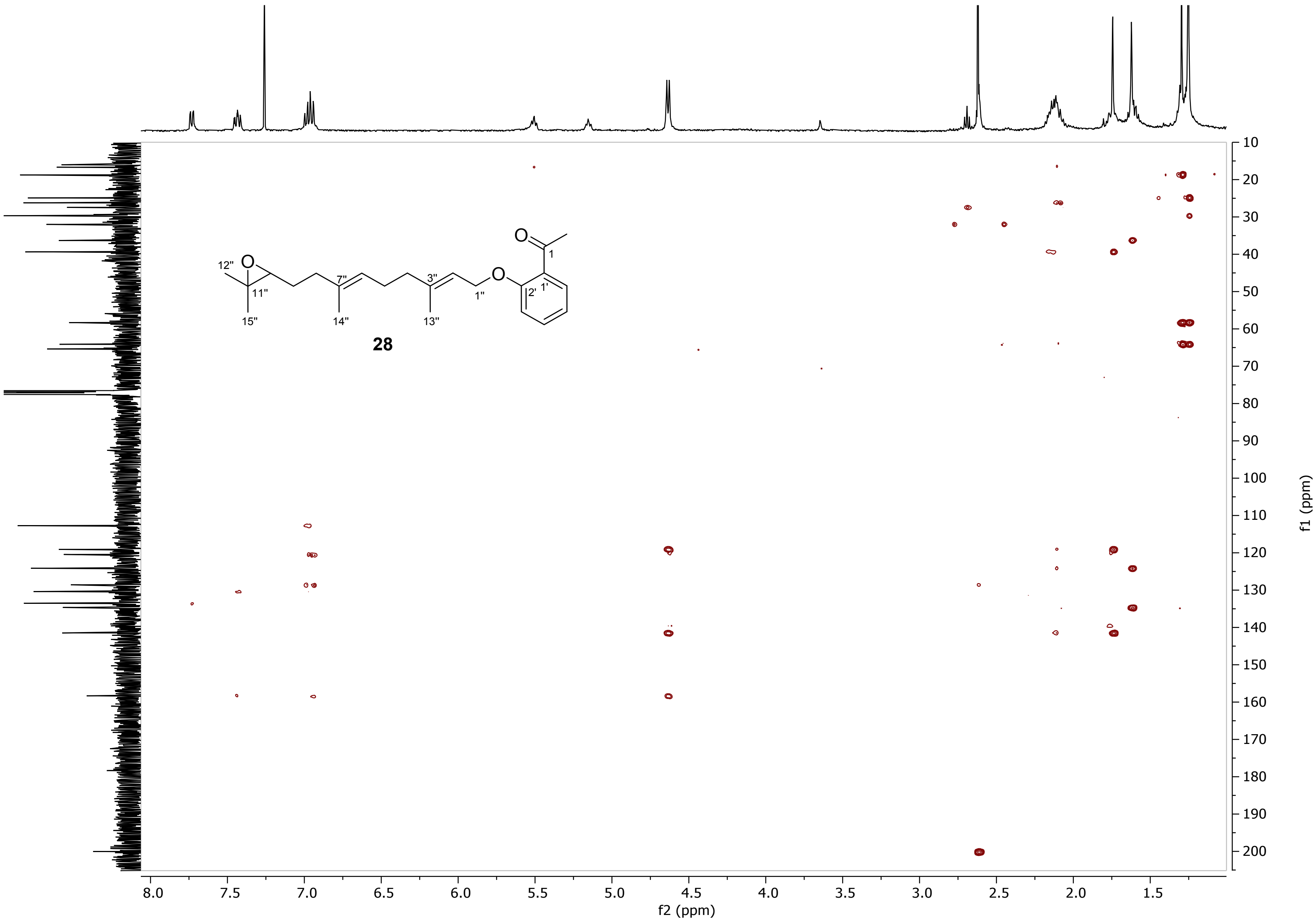

Figure S8e. gHMBC spectrum of (±)-1-(2'-((2''*E*,6''*E*)-10,11-epoxy-3,7,11-trimethyldodeca-2,6-dien-1-yloxy)phenyl)ethanone ((±)-**28**) in $CDCl_3$.

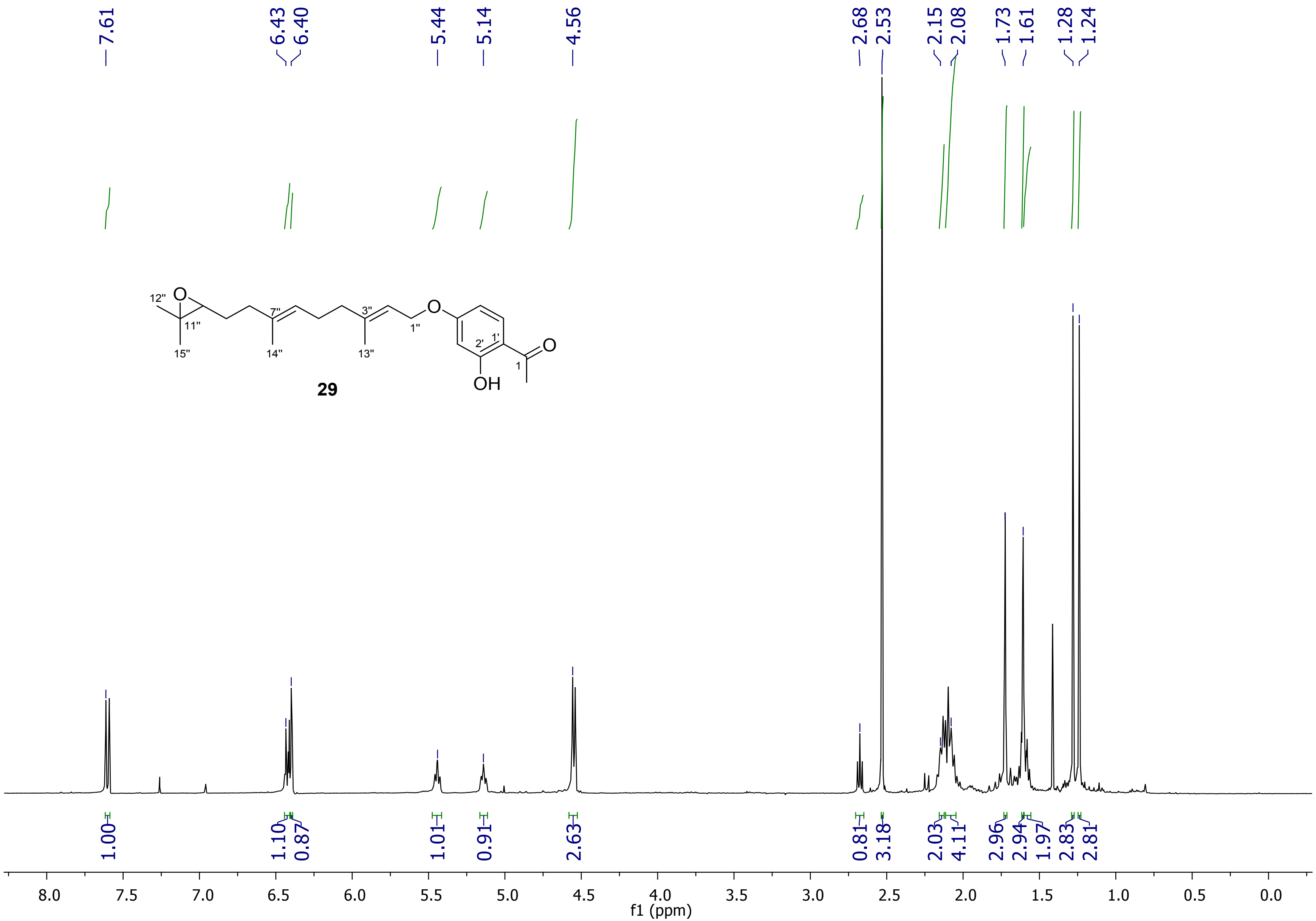

Figure S9a. ^{1}H NMR spectrum (400 MHz) of (±)-1-(4’-((2"*E*,6"*E*)-10,11-epoxy-3,7,11-trimethyldodeca-2,6-dien-1-yloxy)-2’-hydroxyphenyl)ethanone ((±)-**29**) in $CDCl_3$.

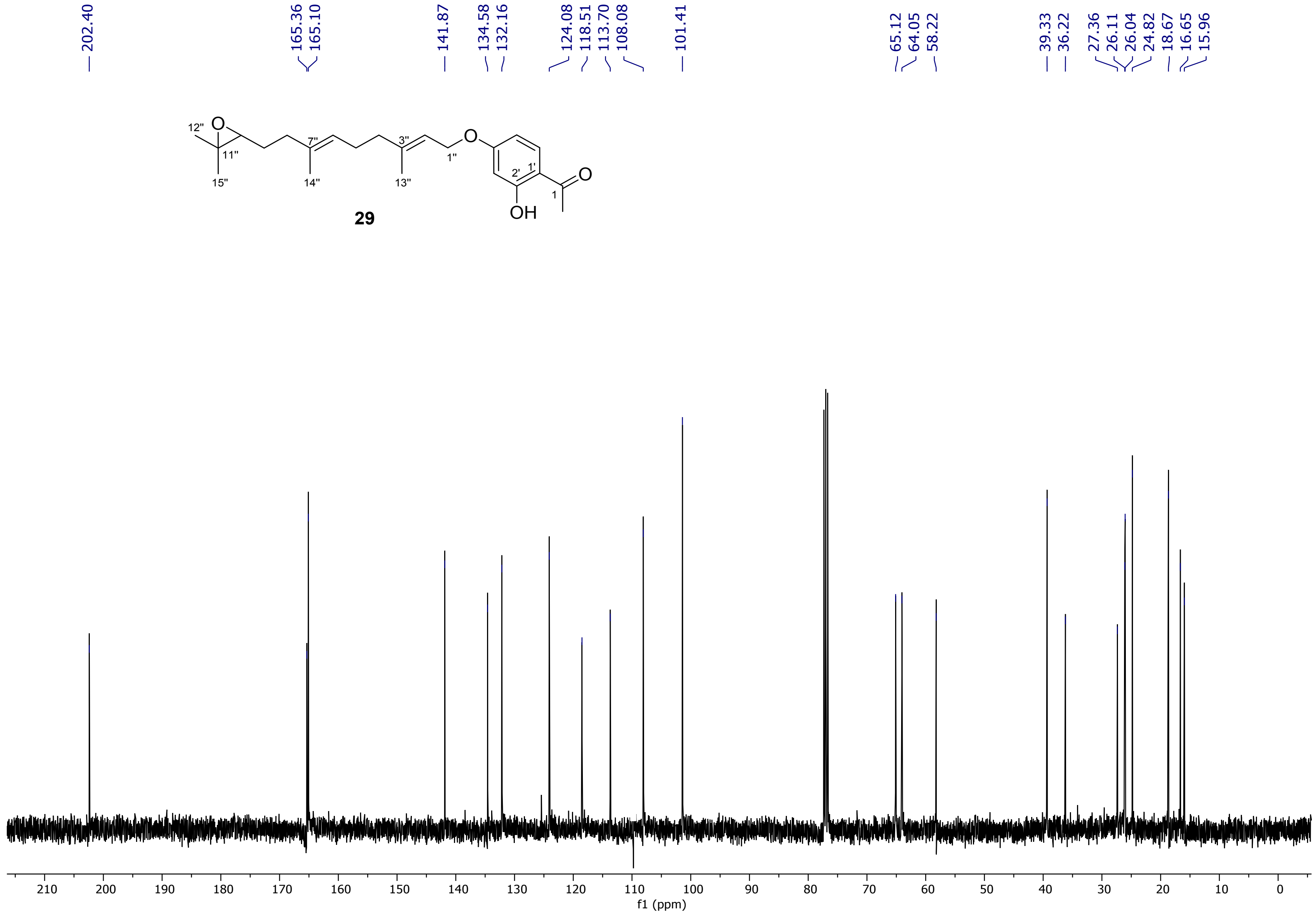

Figure S9b. ^{13}C NMR spectrum (100 MHz) of (±)-1-(4'-((2"*E*,6"*E*)-10,11-epoxy-3,7,11-trimethyldodeca-2,6-dien-1-yloxy)-2'-hydroxyphenyl)ethanone ((±)-**29**) in $CDCl_3$.

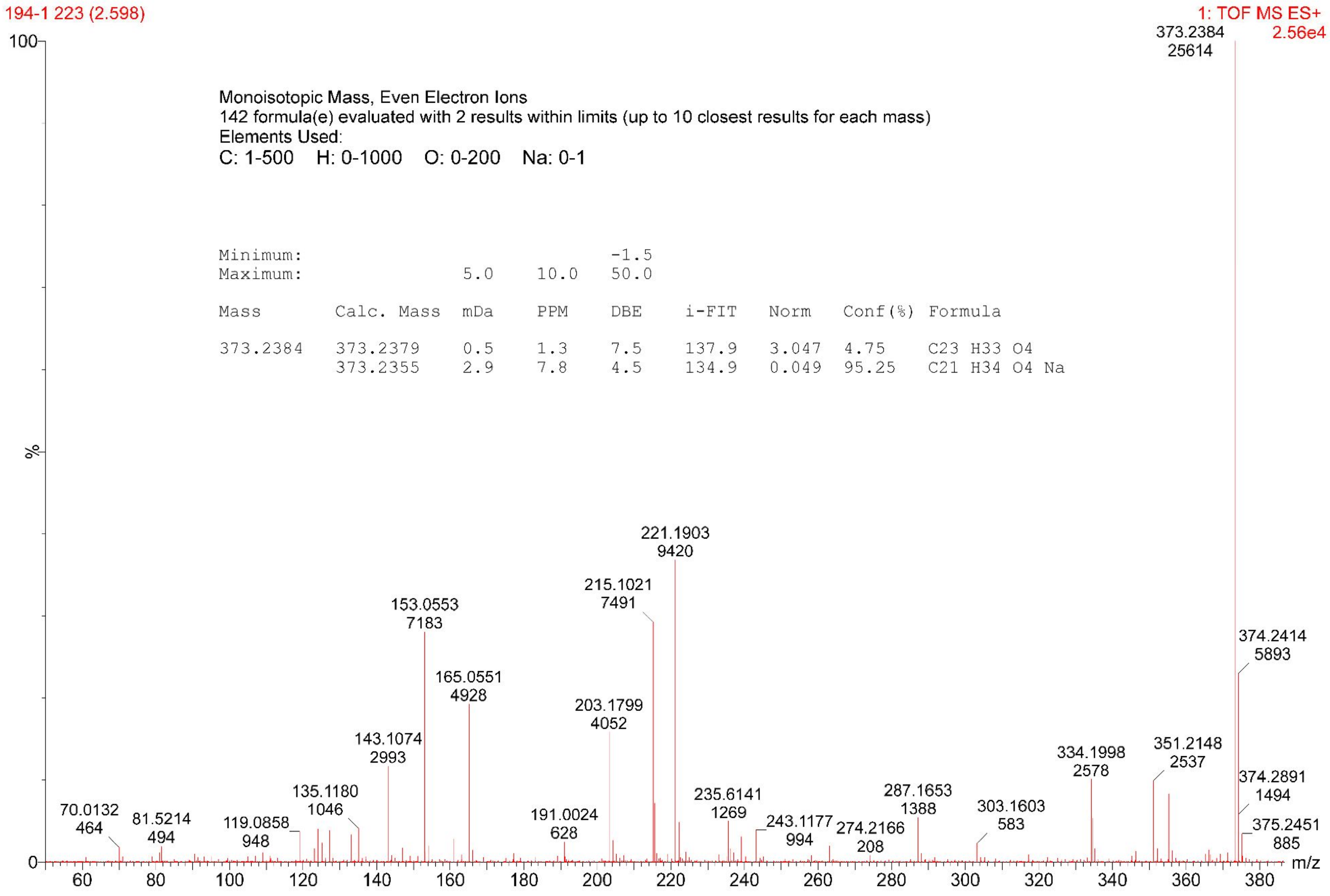

Figure S9c. HRESIMS of 13C NMR spectrum (100 MHz) of (±)-1-(4'-((2"*E*,6"*E*)-10,11-epoxy-3,7,11-trimethyldodeca-2,6-dien-1-yloxy)-2'-hydroxyphenyl)ethanone ((±)-**29**).

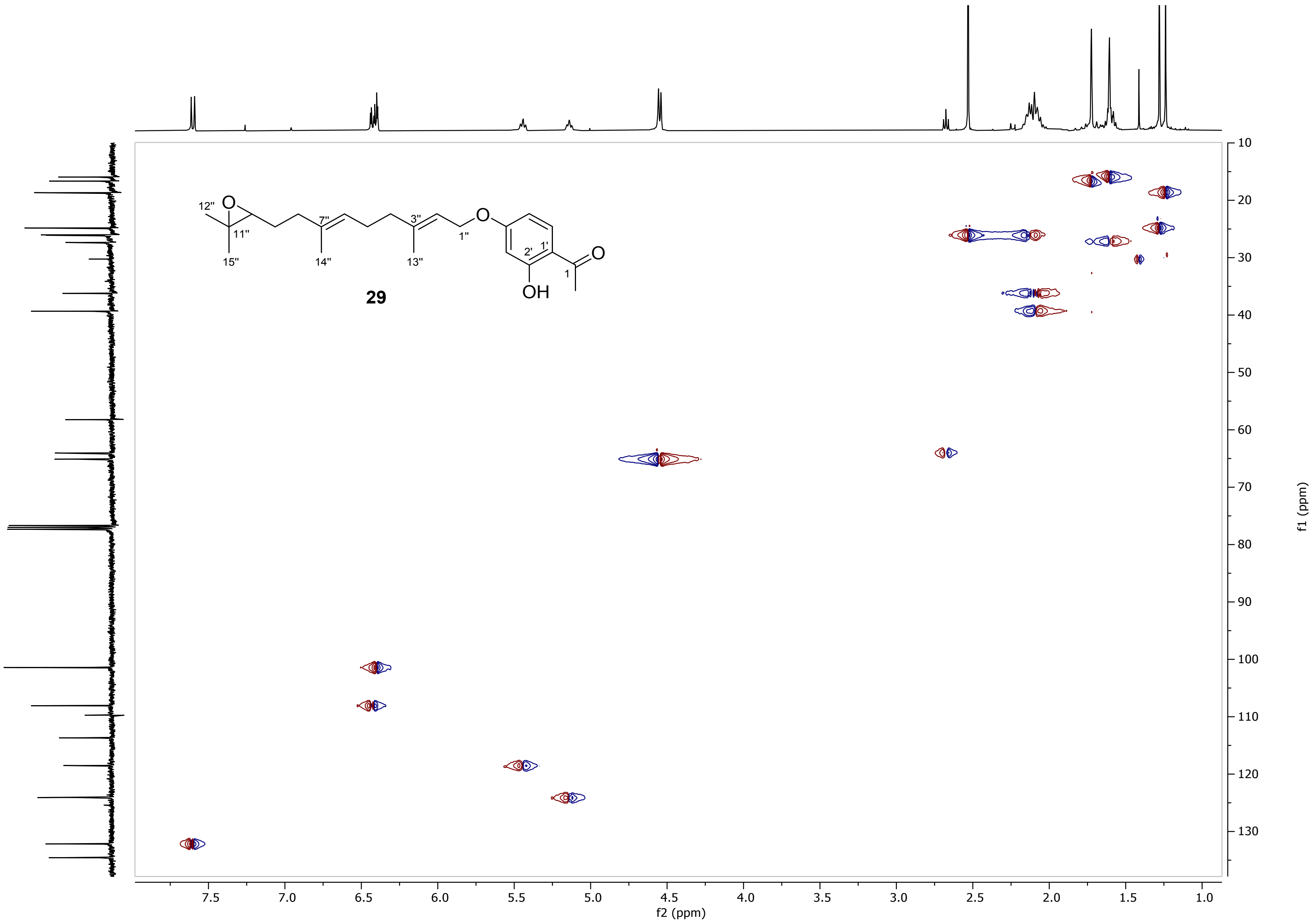

Figure S9d. gHSQC spectrum of (±)-1-(4’-((2"*E*,6"*E*)-10,11-epoxy-3,7,11-trimethyldodeca-2,6-dien-1-yloxy)-2’-hydroxyphenyl)ethanone ((±)-**29**) in $CDCl_3$.

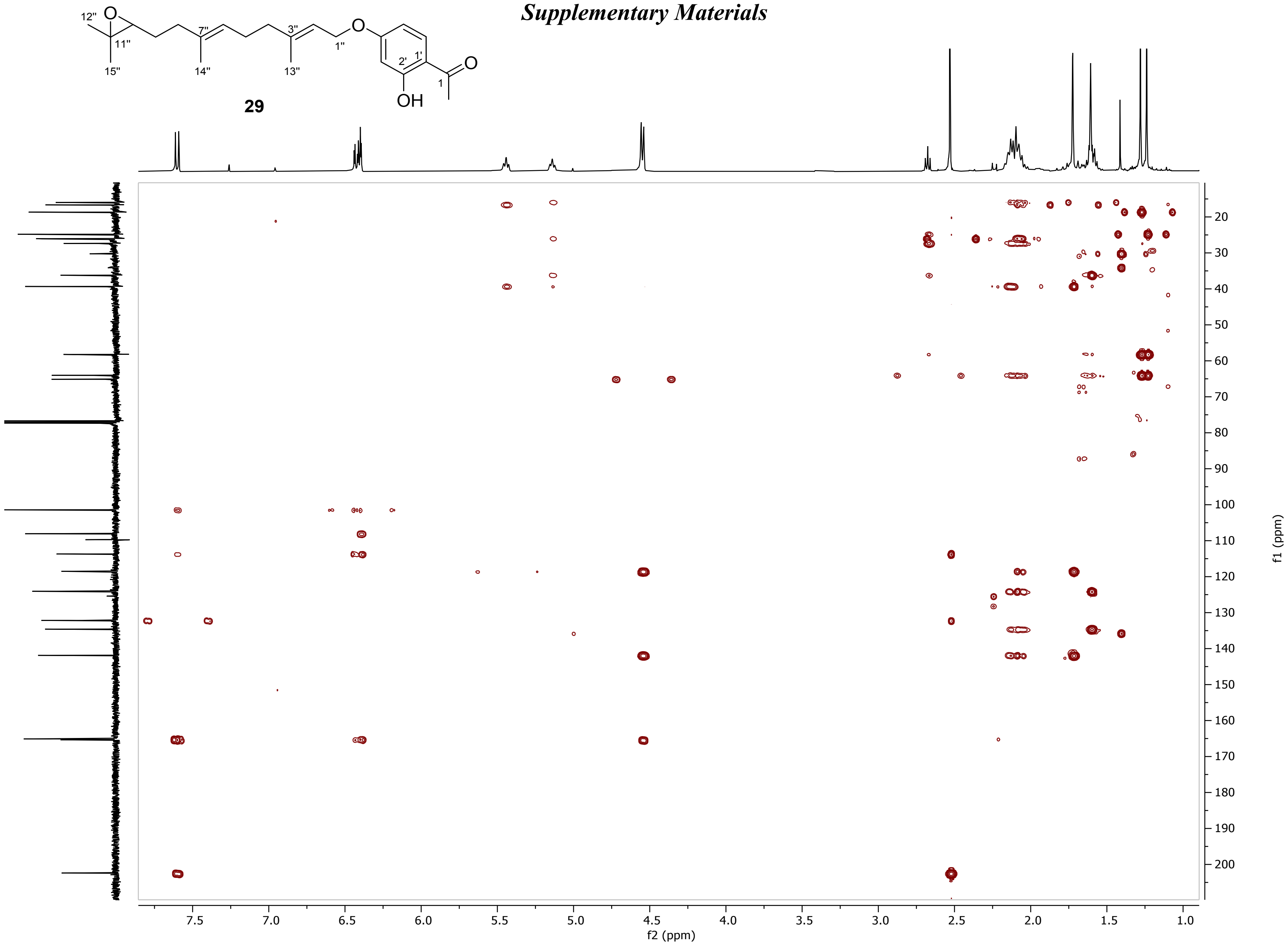

Figure S9e. gHMBC spectrum of (±)-1-(4'-((2"*E*,6"*E*)-10,11-epoxy-3,7,11-trimethyldodeca-2,6-dien-1-yloxy)-2'-hydroxyphenyl)ethanone ((±)-**29**) in $CDCl_3$.

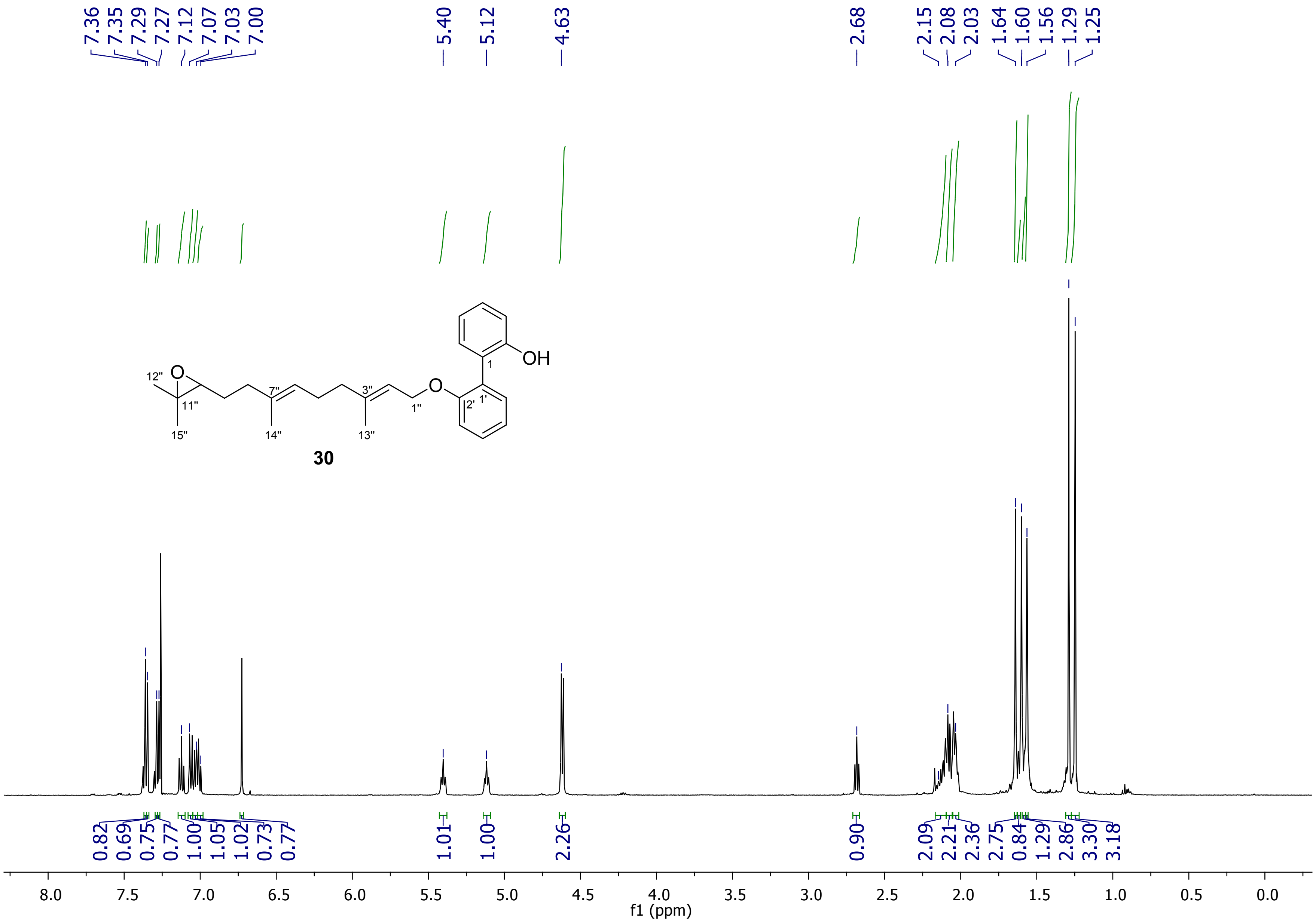

Figure S10a. ^{1}H NMR spectrum (500 MHz) of (±)-2'-((2"*E*,6"*E*)-10,11-epoxy-3,7,11-trimethyldodeca-2,6-dien-1-yloxy)-[1,1'-biphenyl]-2-ol ((±)-**30**) in $CDCl_3$.

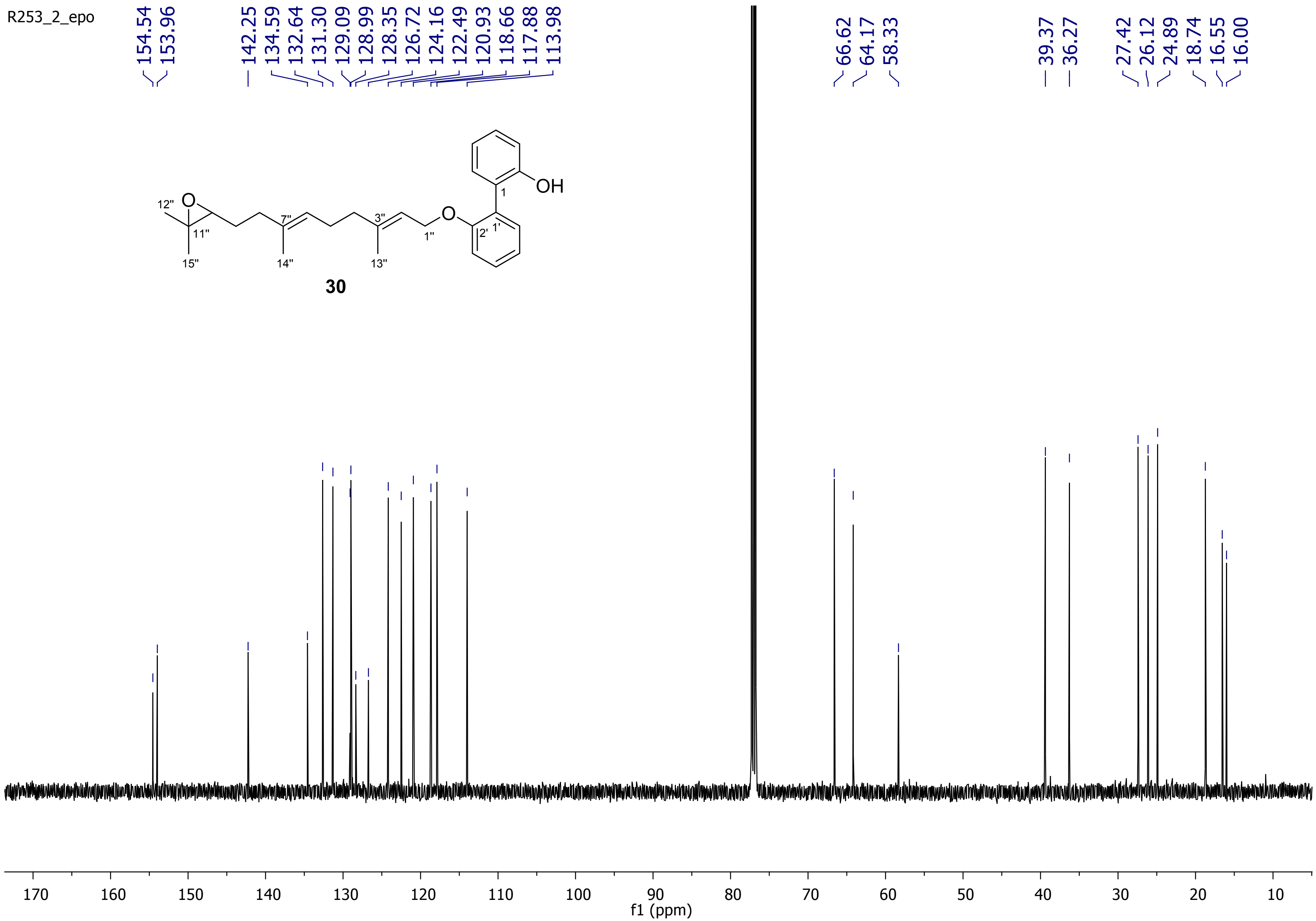

Figure S10b. ^{13}C NMR spectrum (125 MHz) of (±)-2'-((2"*E*,6"*E*)-10,11-epoxy-3,7,11-trimethyldodeca-2,6-dien-1-yloxy)-[1,1'-biphenyl]-2-ol ((±)-**30**) in $CDCl_3$.

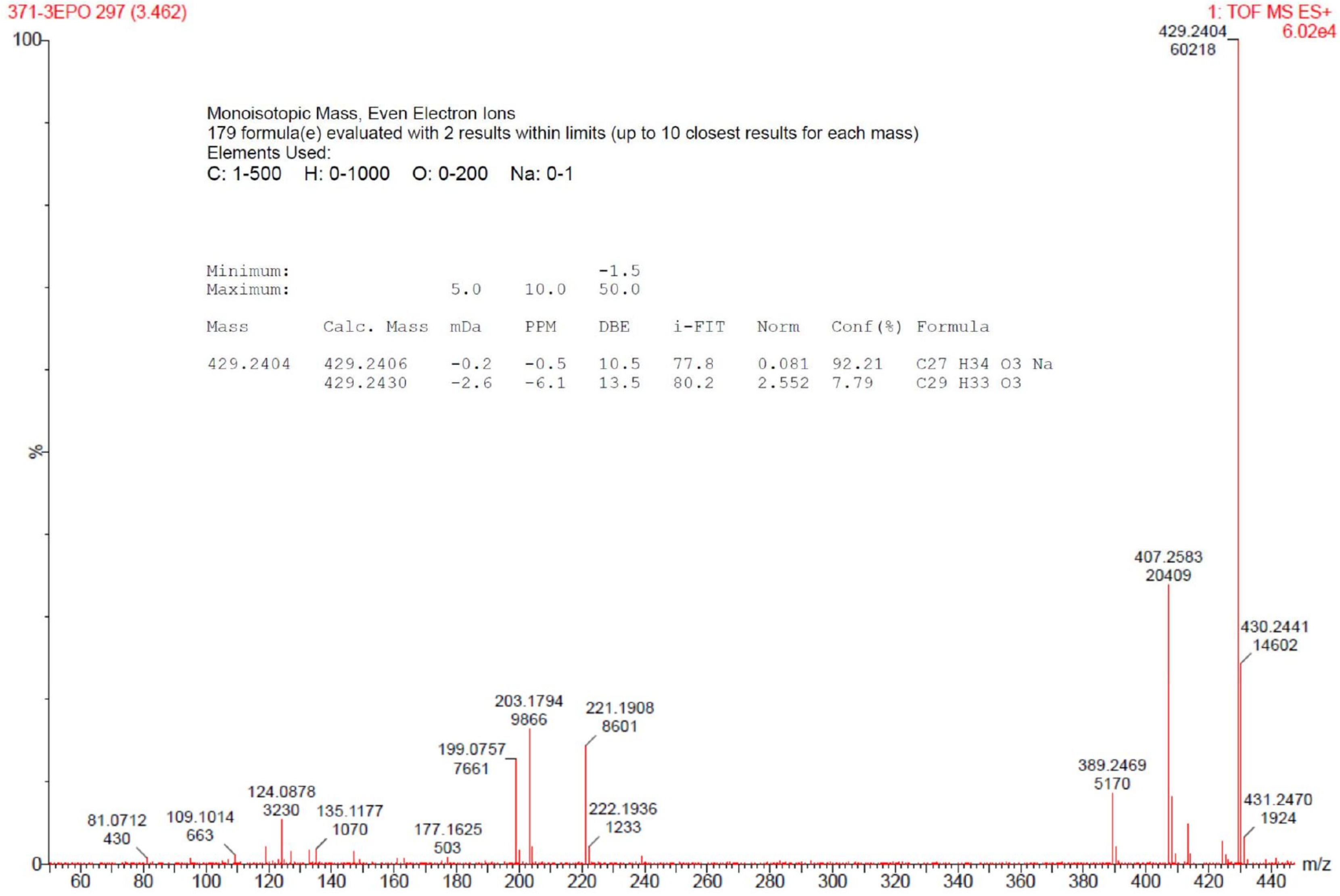

	Mass	Calc. Mass	mDa	PPM	DBE	i-FIT	Norm	Conf(%)	Formula
Minimum:					-1.5				
Maximum:			5.0	10.0	50.0				
	429.2404	429.2406	-0.2	-0.5	10.5	77.8	0.081	92.21	C27 H34 O3 Na
		429.2430	-2.6	-6.1	13.5	80.2	2.552	7.79	C29 H33 O3

Figure S10c. HRESIMS of (±)-2'-((2''*E*,6''*E*)-10,11-epoxy-3,7,11-trimethyldodeca-2,6-dien-1-yloxy)-[1,1'-biphenyl]-2-ol ((±)-**30**).

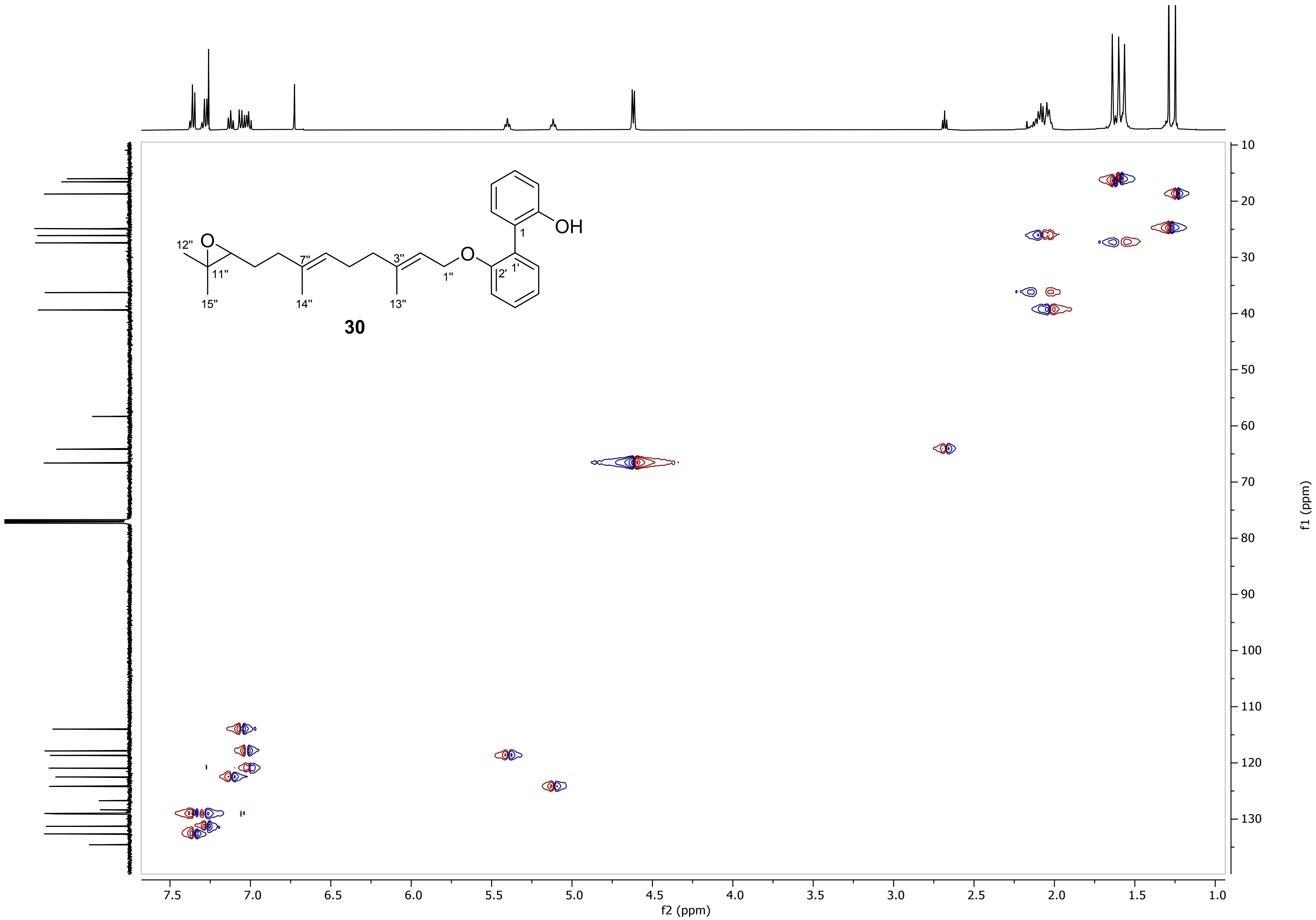

Figure S10d. gHSQC spectrum of (±)-2'-((2"*E*,6"*E*)-10,11-epoxy-3,7,11-trimethyldodeca-2,6-dien-1-yloxy)-[1,1'-biphenyl]-2-ol ((±)-**30**) in $CDCl_3$.

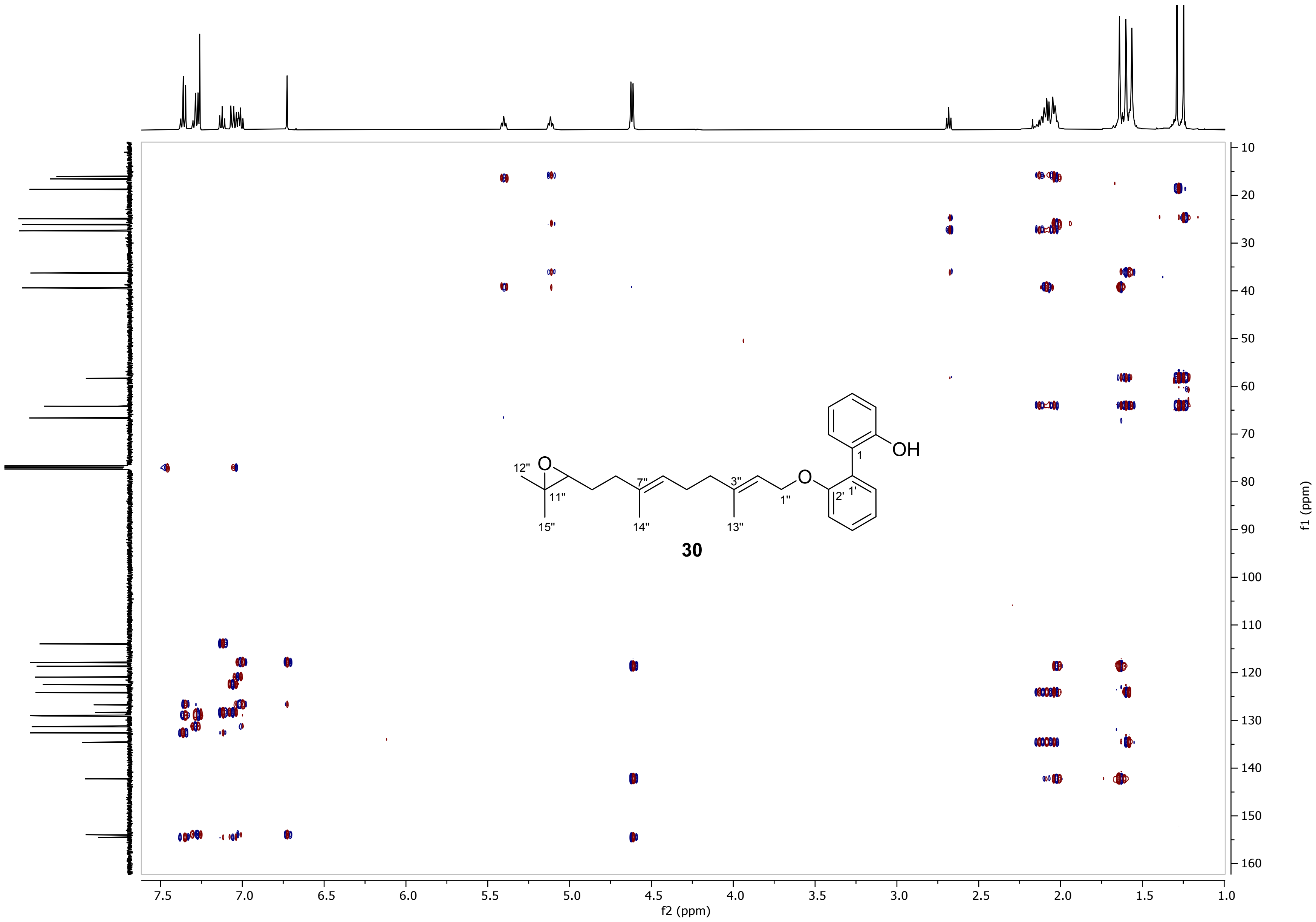

Figure S10e. gHMBC spectrum of (±)-2'-((2''*E*,6''*E*)-10,11-epoxy-3,7,11-trimethyldodeca-2,6-dien-1-yloxy)-[1,1'-biphenyl]-2-ol ((±)-**30**) in $CDCl_3$.

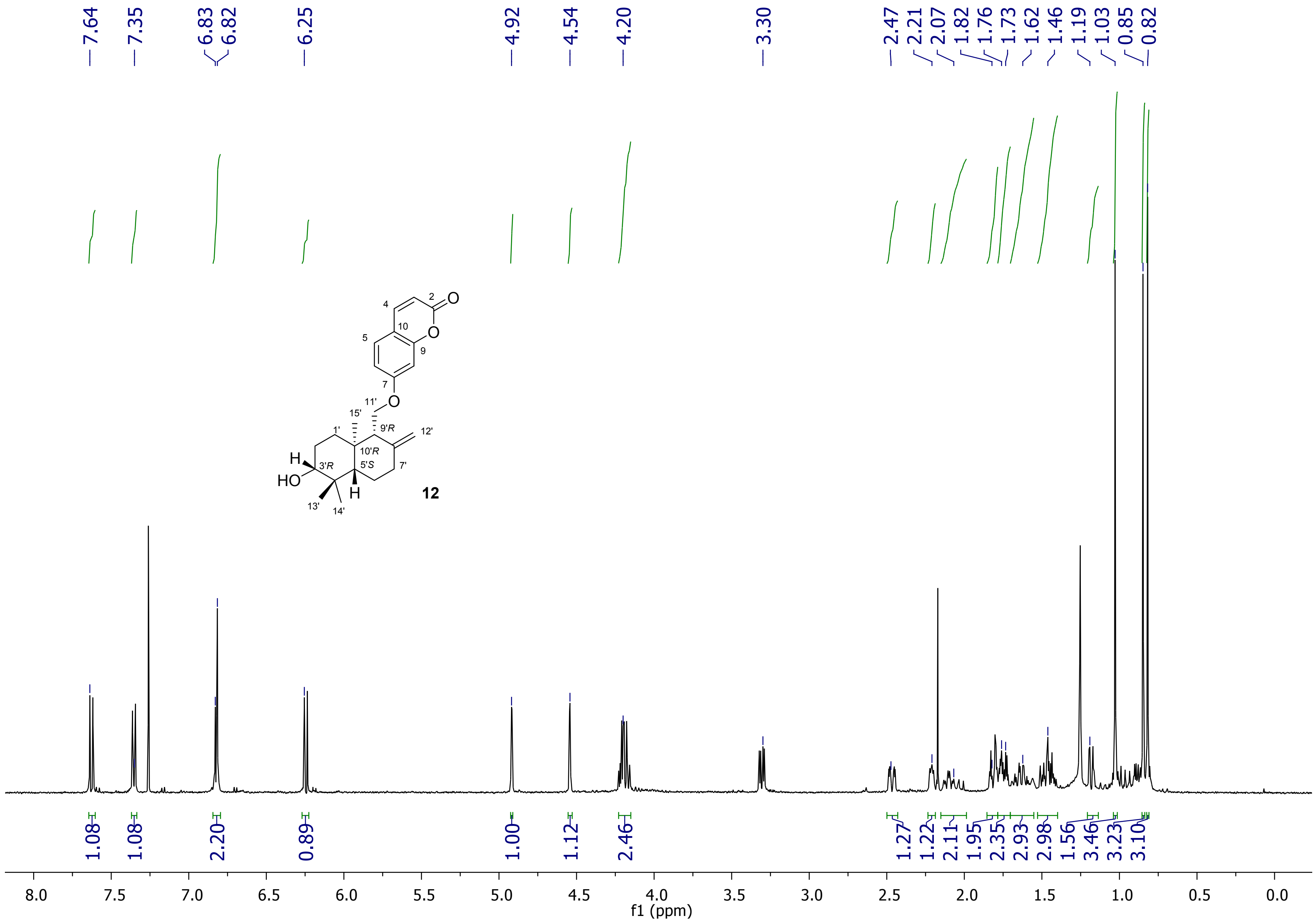

Figure S11a. ^{1}H NMR spectrum (500 MHz) of (±)-7-(3'*R*(*S*),5'*S*(*R*),9'*R*(*S*),10'*R*(*S*)-3'-hydroxydrim-8'(12')-en-11'-yloxy)-coumarin ((±)-coladonin ((±)-**12**)) in $CDCl_3$).

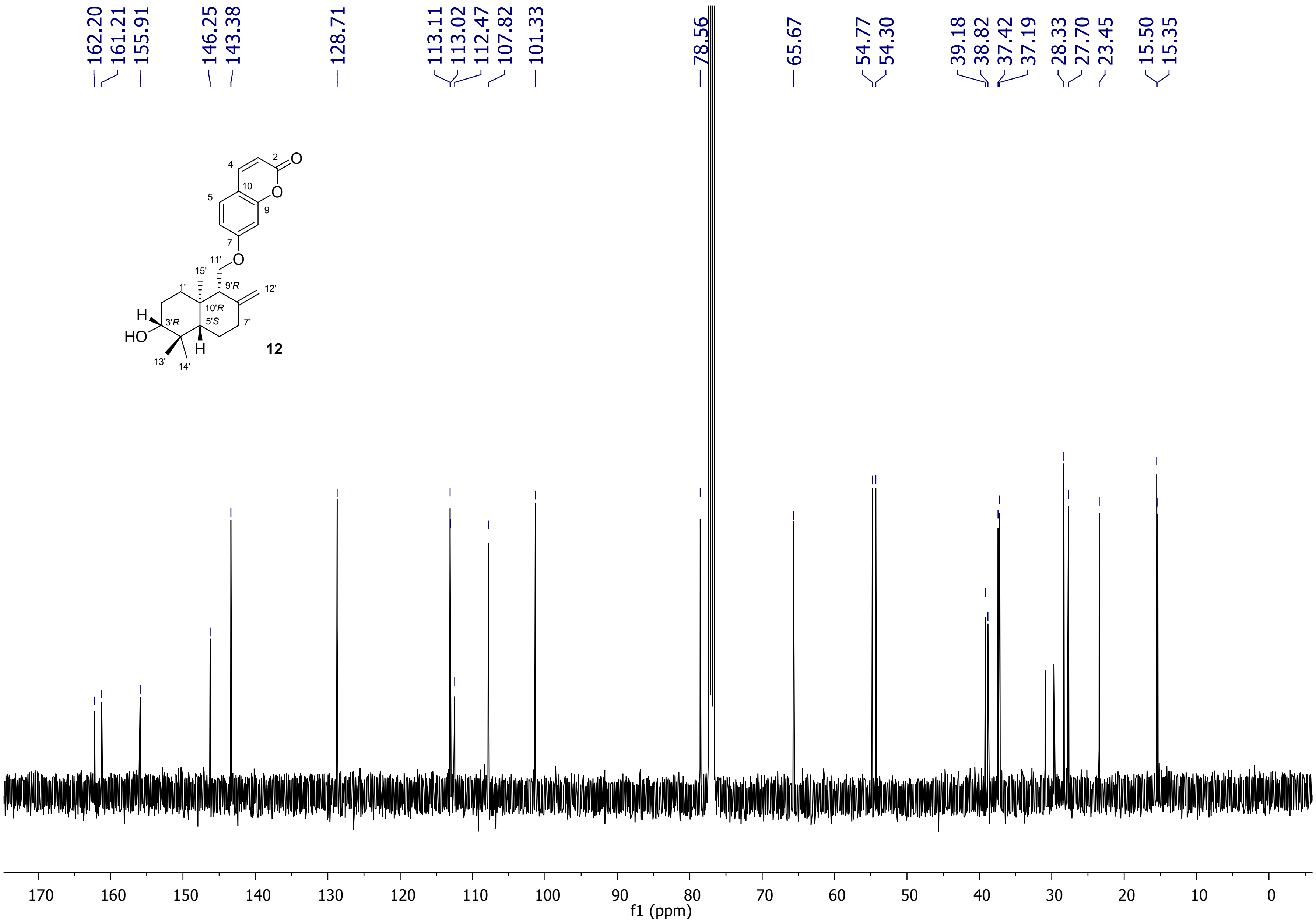

Figure S11b. ^{13}C NMR spectrum (125 MHz) of (±)-7-(3'*R*(*S*),5'*S*(*R*),9'*R*(*S*),10'*R*(*S*)-3'-hydroxydrim-8'(12')-en-11'-yloxy)-coumarin ((±)-coladonin) ((±)-**12**)) in $CDCl_3$).

Monoisotopic Mass, Odd and Even Electron Ions
83 formula(e) evaluated with 2 results within limits (up to 50 best isotopic matches for each mass)
Elements Used:
C: 0-500   H: 0-1000   O: 0-200

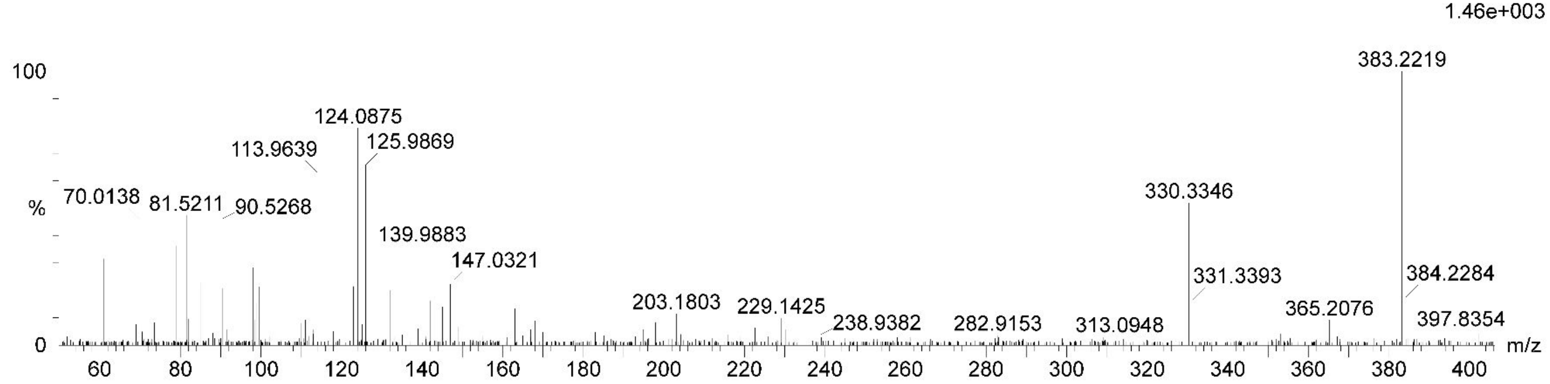

Minimum:	80.00				-1.5				
Maximum:	100.00		10.0	10.0	50.0				
Mass	RA	Calc. Mass	mDa	PPM	DBE	i-FIT	Norm	Conf(%)	Formula
383.2219	100.00	383.2281	-6.2	-16.2	0.5	36.5	0.067	93.48	C17 H35 O9
		383.2222	-0.3	-0.8	9.5	39.1	2.731	6.52	C24 H31 O4

Figure S11c. HRESIMS of (±)-7-(3'*R*(*S*),5'*S*(*R*),9'*R*(*S*),10'*R*(*S*)-3'-hydroxydrim-8'(12')-en-11'-yloxy)-coumarin ((±)-coladonin) ((±)-**12**).

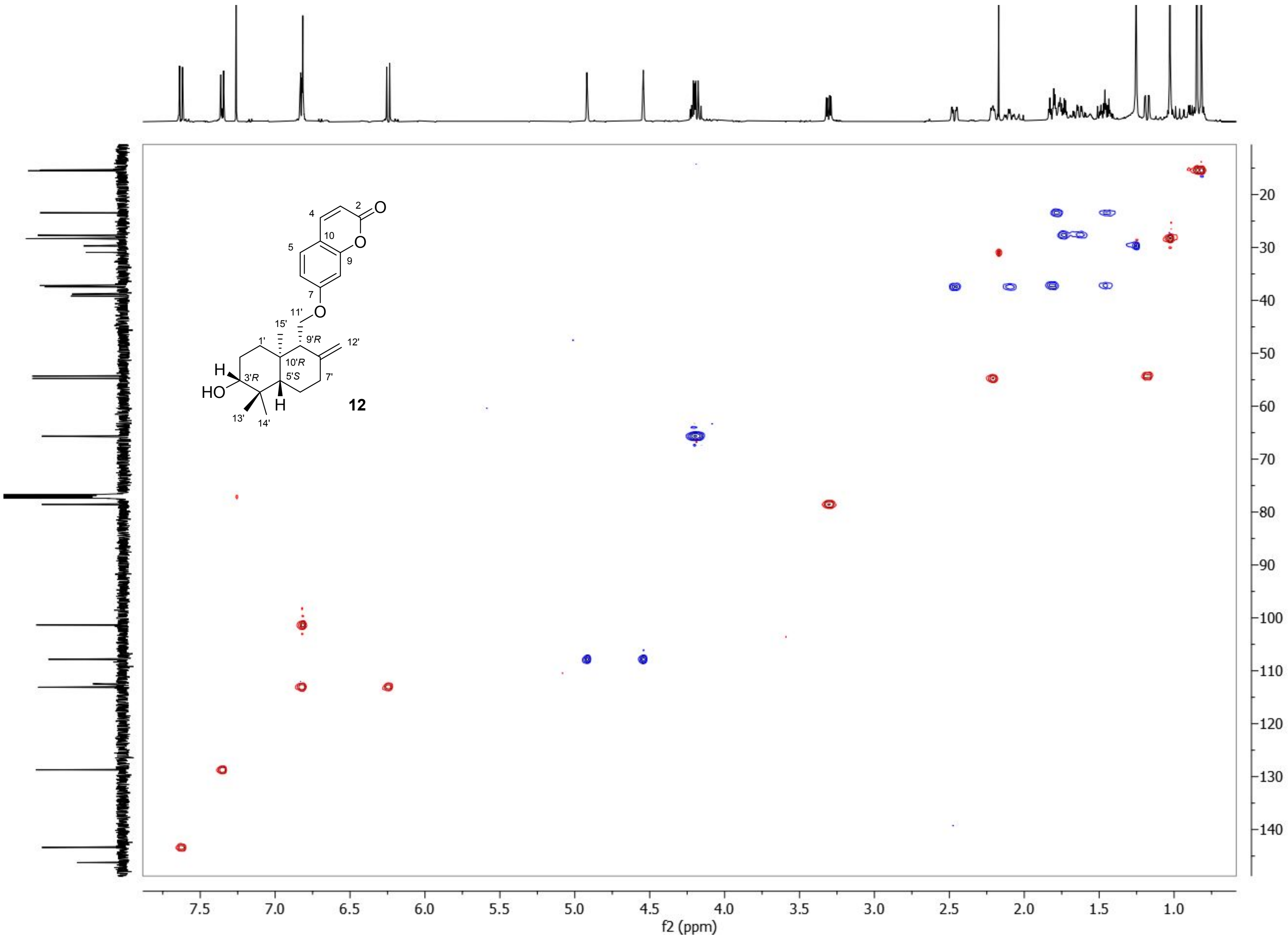

Figure S11d. gHSQC spectrum of (±)-7-(3'*R*(*S*),5'*S*(*R*),9'*R*(*S*),10'*R*(*S*)-3'-hydroxydrim-8'(12')-en-11'-yloxy)-coumarin ((±)-coladonin) ((±)-**12**)) in $CDCl_3$.

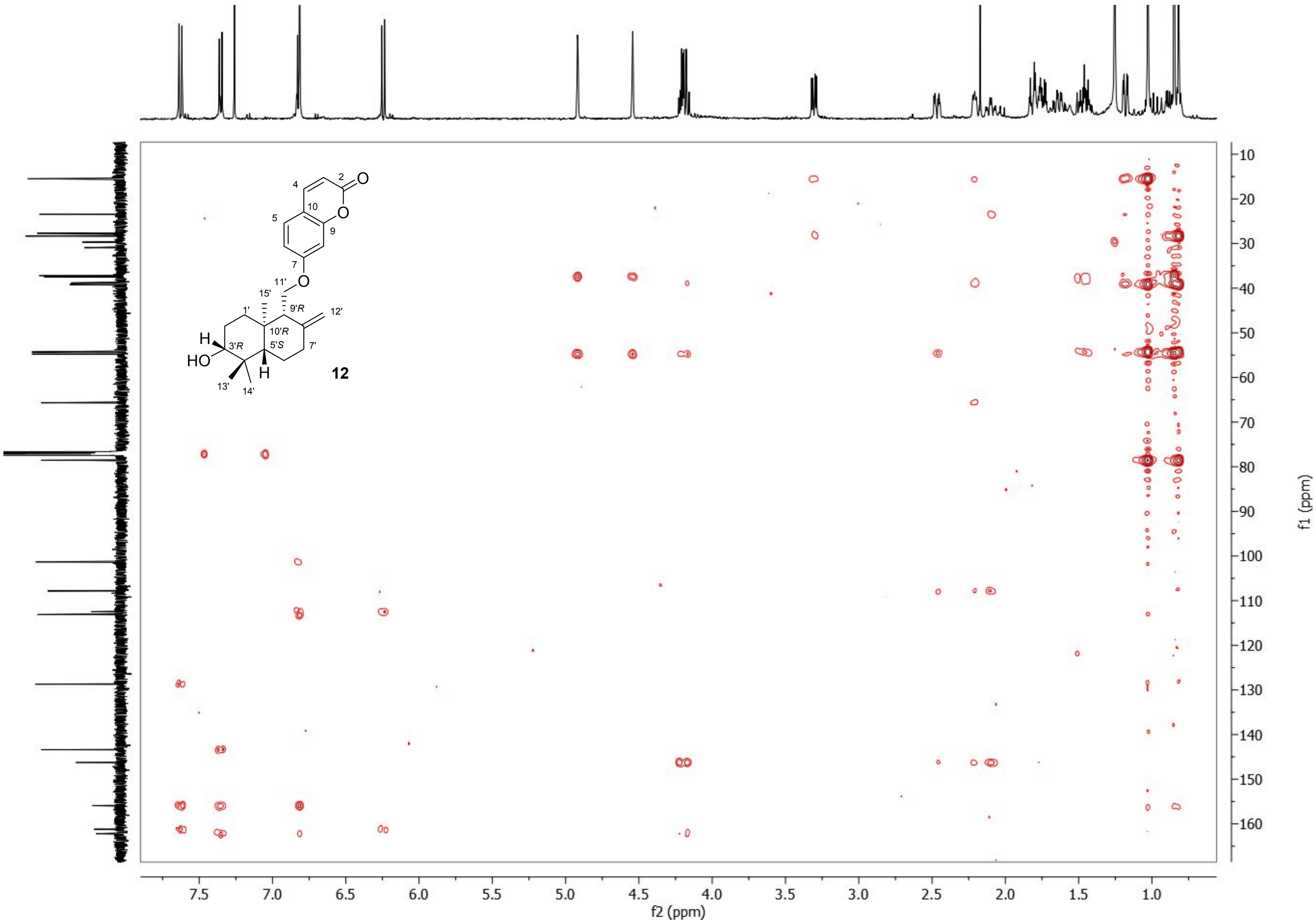

Figure S11e. gHMBC spectrum of (±)-7-(3'*R*(*S*),5'*S*(*R*),9'*R*(*S*),10'*R*(*S*)-3'-hydroxydrim-8'(12')-en-11'-yloxy)-coumarin ((±)-coladonin) ((±)-**12**)) in $CDCl_3$.

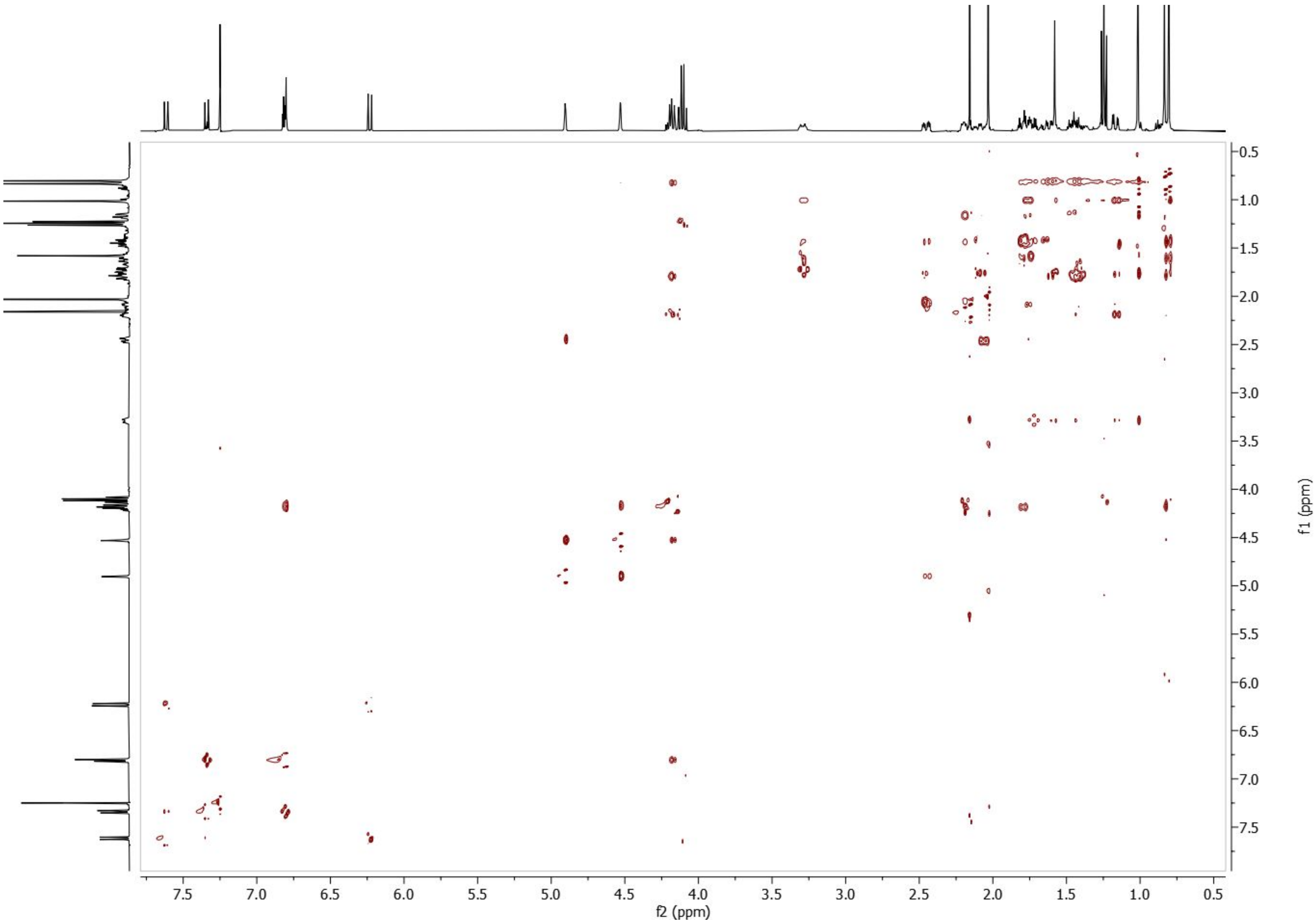

Figure S11f. NOESY2D spectrum of (±)-7-(3'*R*(*S*),5'*S*(*R*),9'*R*(*S*),10'*R*(*S*)-3'-hydroxydrim-8'(12')-en-11'-yloxy)-coumarin ((±)-coladonin) ((±)-**12**)) in $CDCl_3$.

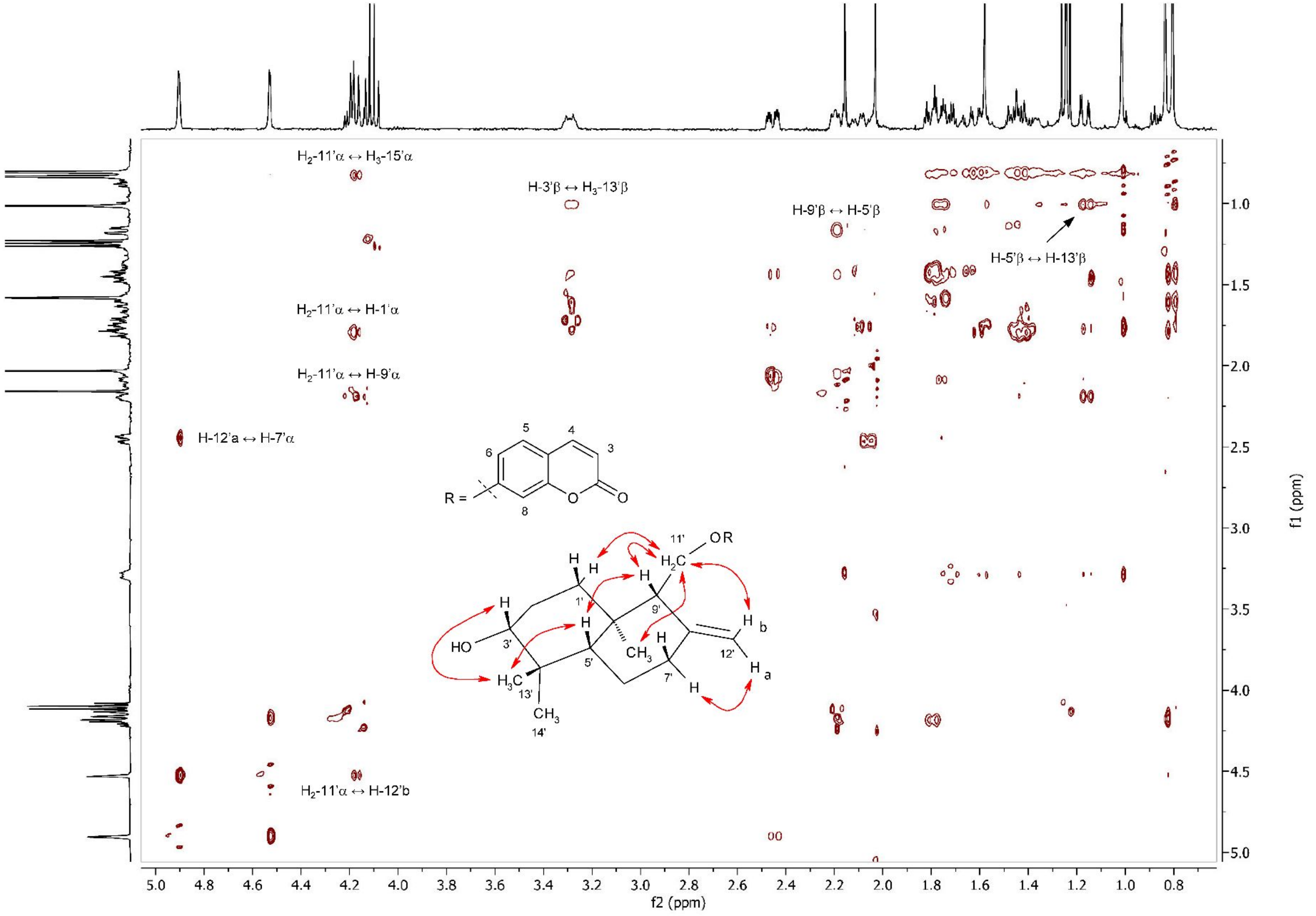

Figure S11g. Expansion of NOESY2D spectrum of (±)-7-(3'*R*(*S*),5'*S*(*R*),9'*R*(*S*),10'*R*(*S*)-3'-hydroxydrim-8'(12')-en-11'-yloxy)-coumarin ((±)-coladonin) ((±)-**12**)) in $CDCl_3$. Selected NOESY2D correlations are annotated and highlighted on (±)-coladonin ((±)-**12**) structure.

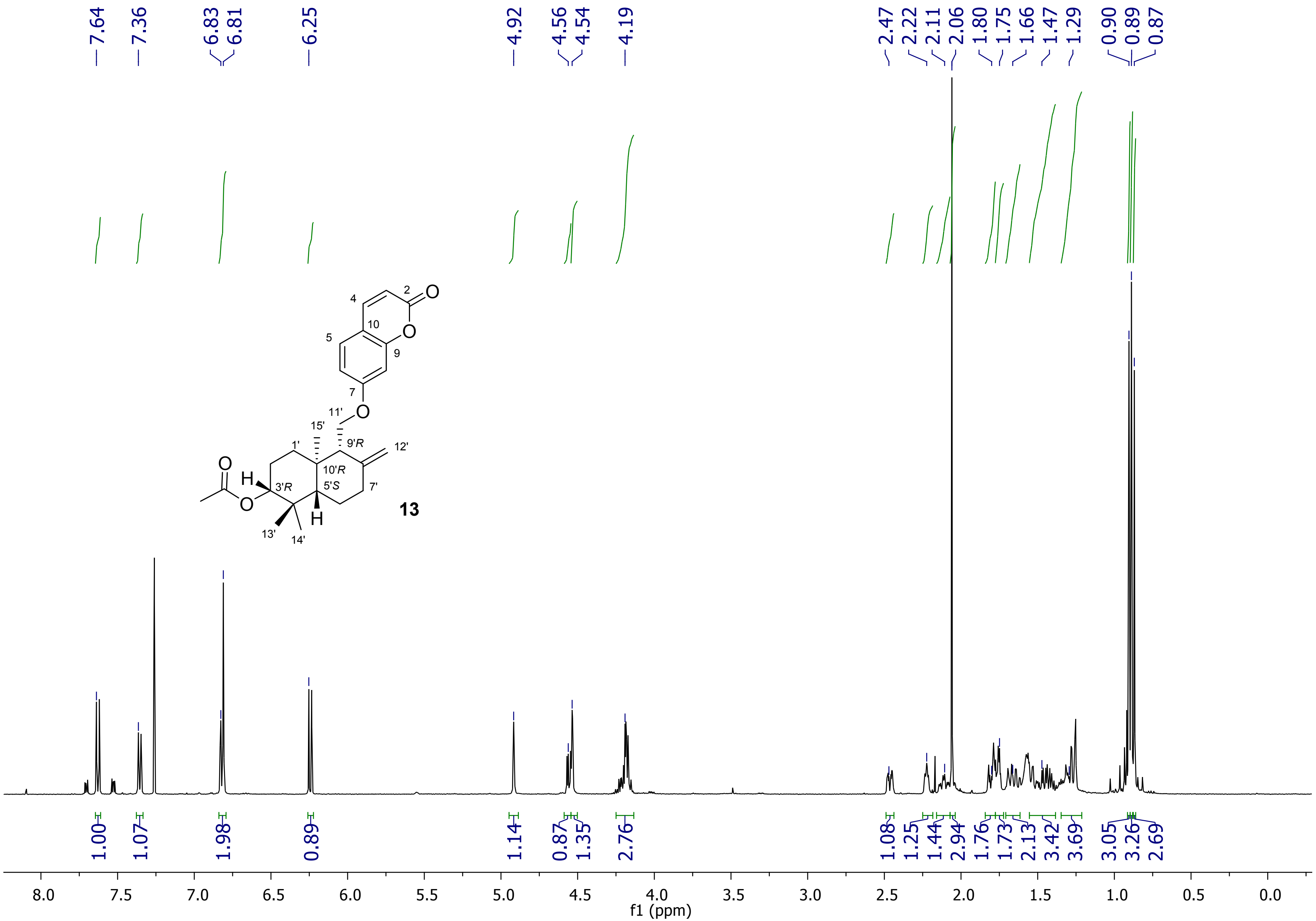

Figure S12a. ^{1}H NMR spectrum (500 MHz) of (±)-7-(3'*R*(*S*),5'*S*(*R*),9'*R*(*S*),10'*R*(*S*)-3'-acetoxydrim-8'(12')-en-11'-yloxy)-coumarin ((±)-coladin) ((±)-**13**)) in $CDCl_3$.

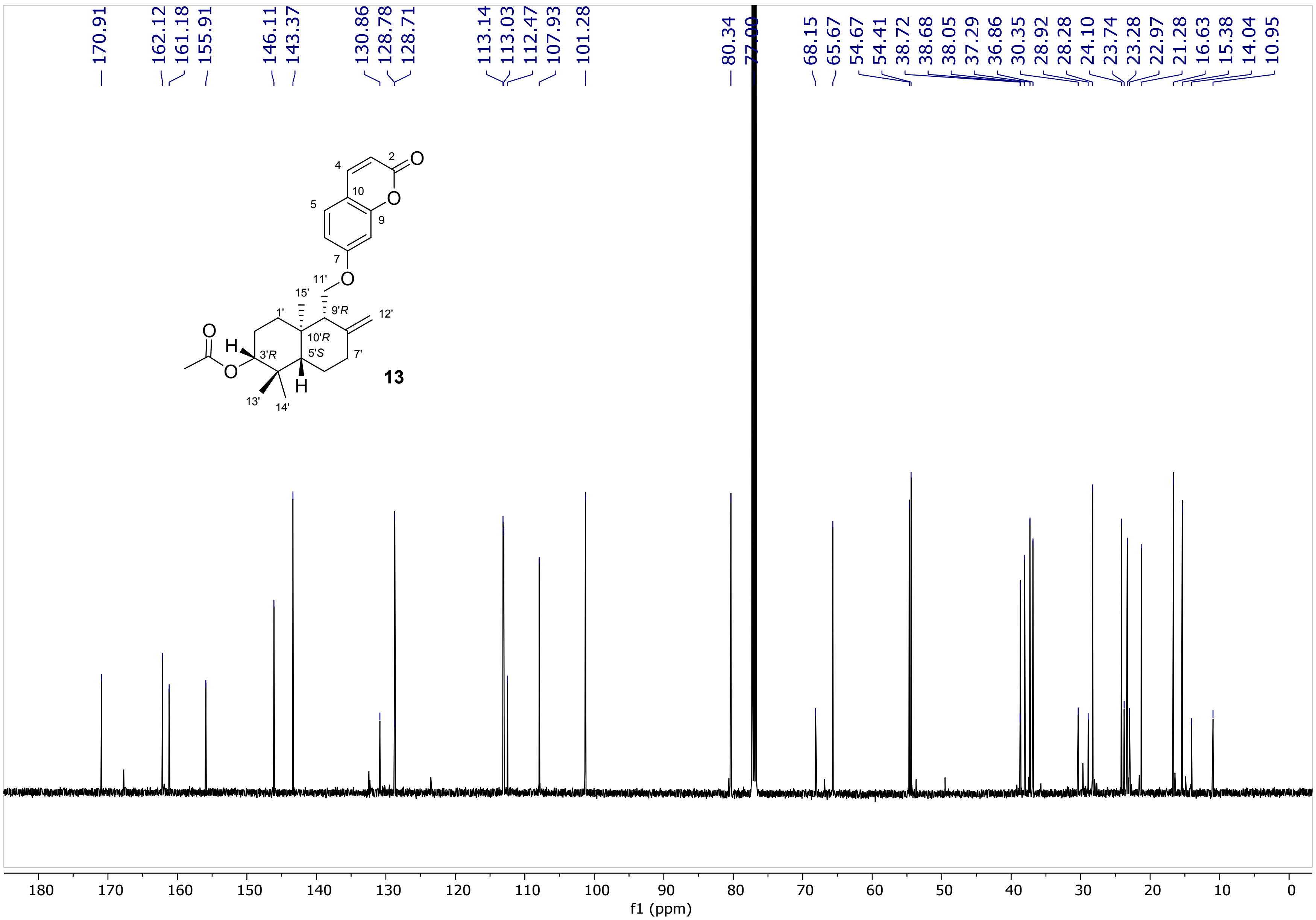

Figure S12b. ^{13}C NMR spectrum (125 MHz) of (±)-7-(3'*R*(*S*),5'*S*(*R*),9'*R*(*S*),10'*R*(*S*)-3'-acetoxydrim-8'(12')-en-11'-yloxy)-coumarin ((±)-coladin) ((±)-**13**)) in CDCl$_3$.

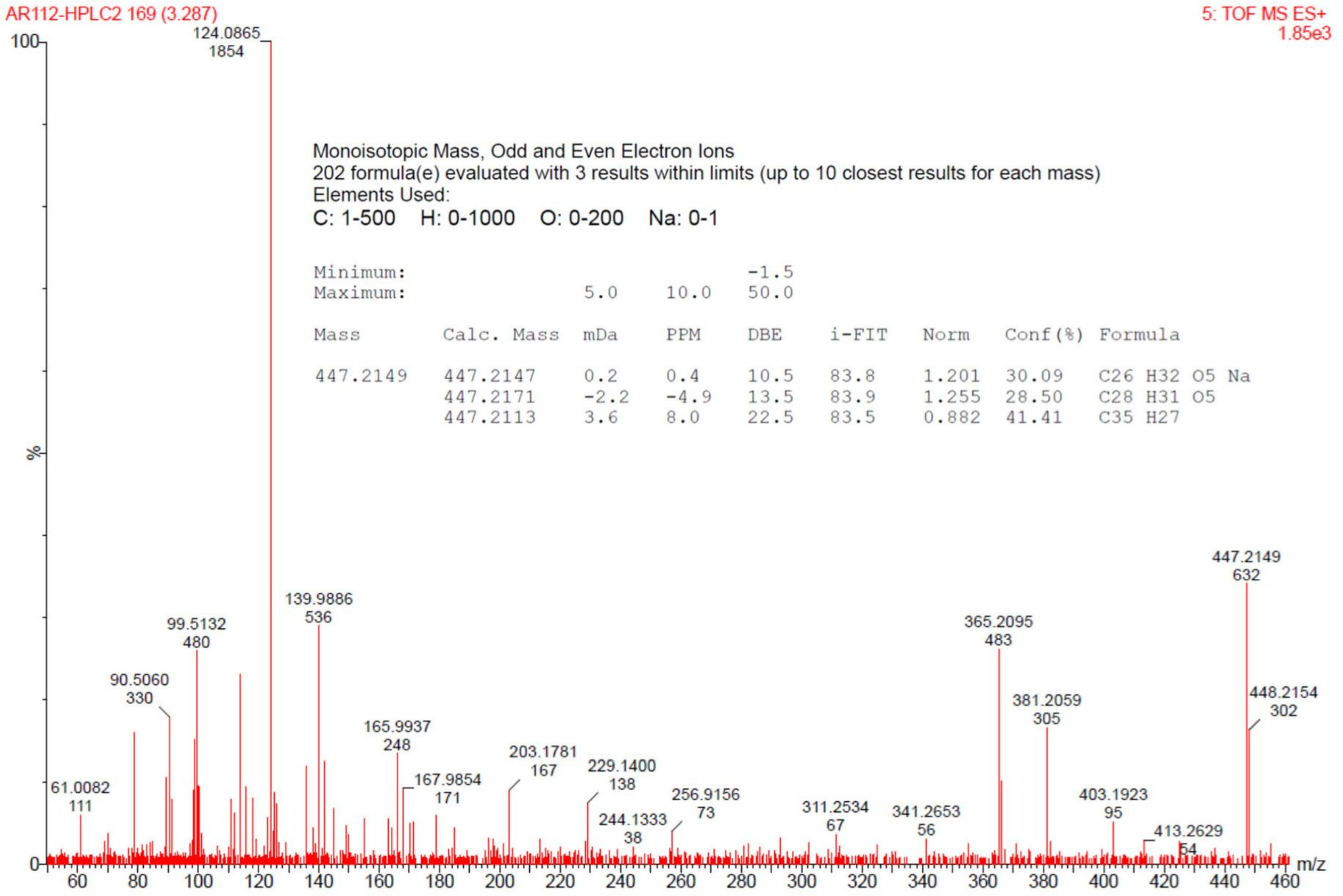

Figure S12c. HRESIMS of (±)-7-(3'*R*(*S*),5'*S*(*R*),9'*R*(*S*),10'*R*(*S*)-3'-acetoxydrim-8'(12')-en-11'-yloxy)-coumarin ((±)-coladin ((±)-**13**)).

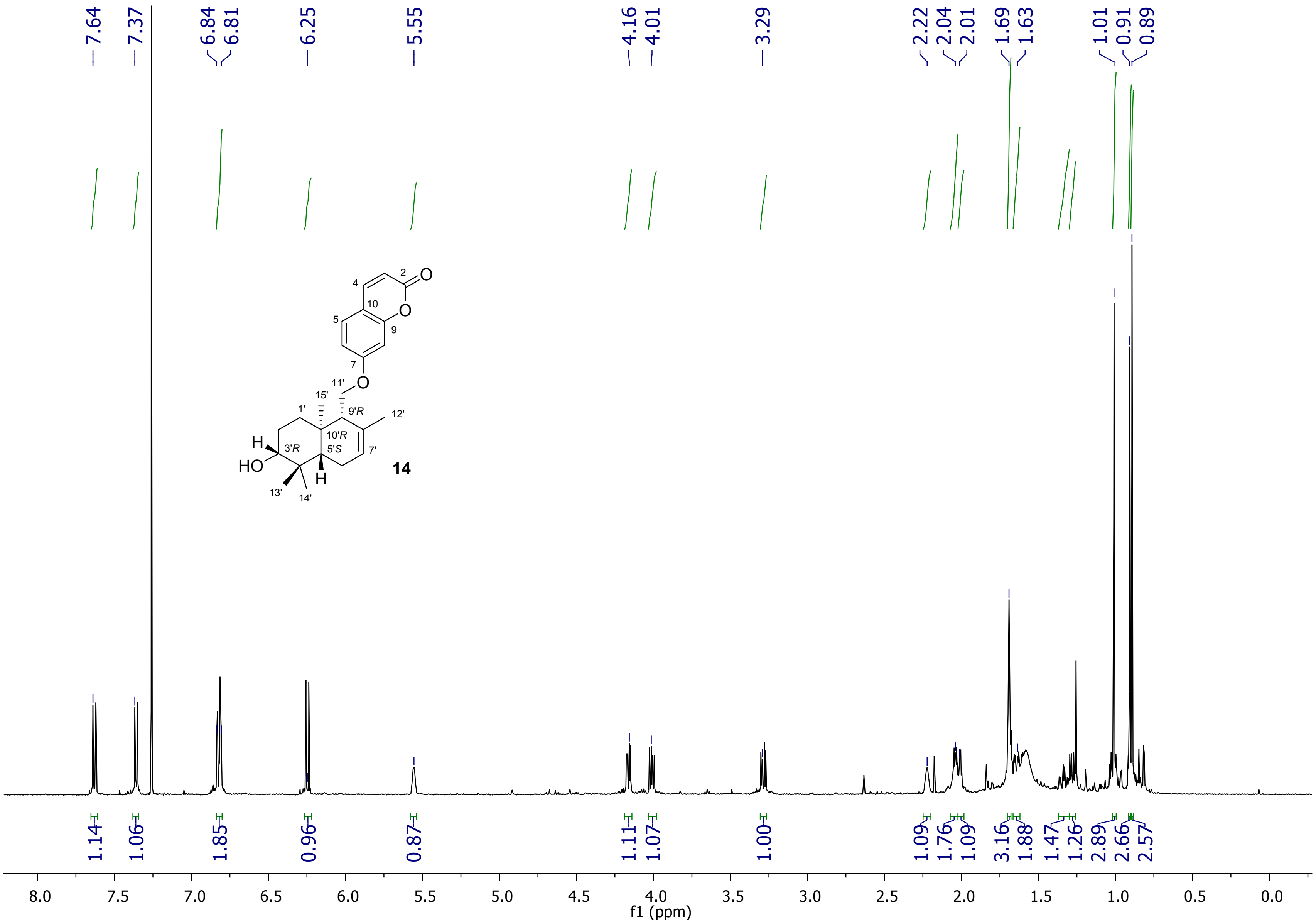

Figure S13a. ^{1}H NMR spectrum (500 MHz) of (±)-7-(3'*R*(*S*),5'*S*(*R*),9'*R*(*S*),10'*R*(*S*)-3'-hydroxydrim-7'-en-11'-yloxy)-coumarin ((±)-feselol) ((±)-**14**)) in $CDCl_3$.

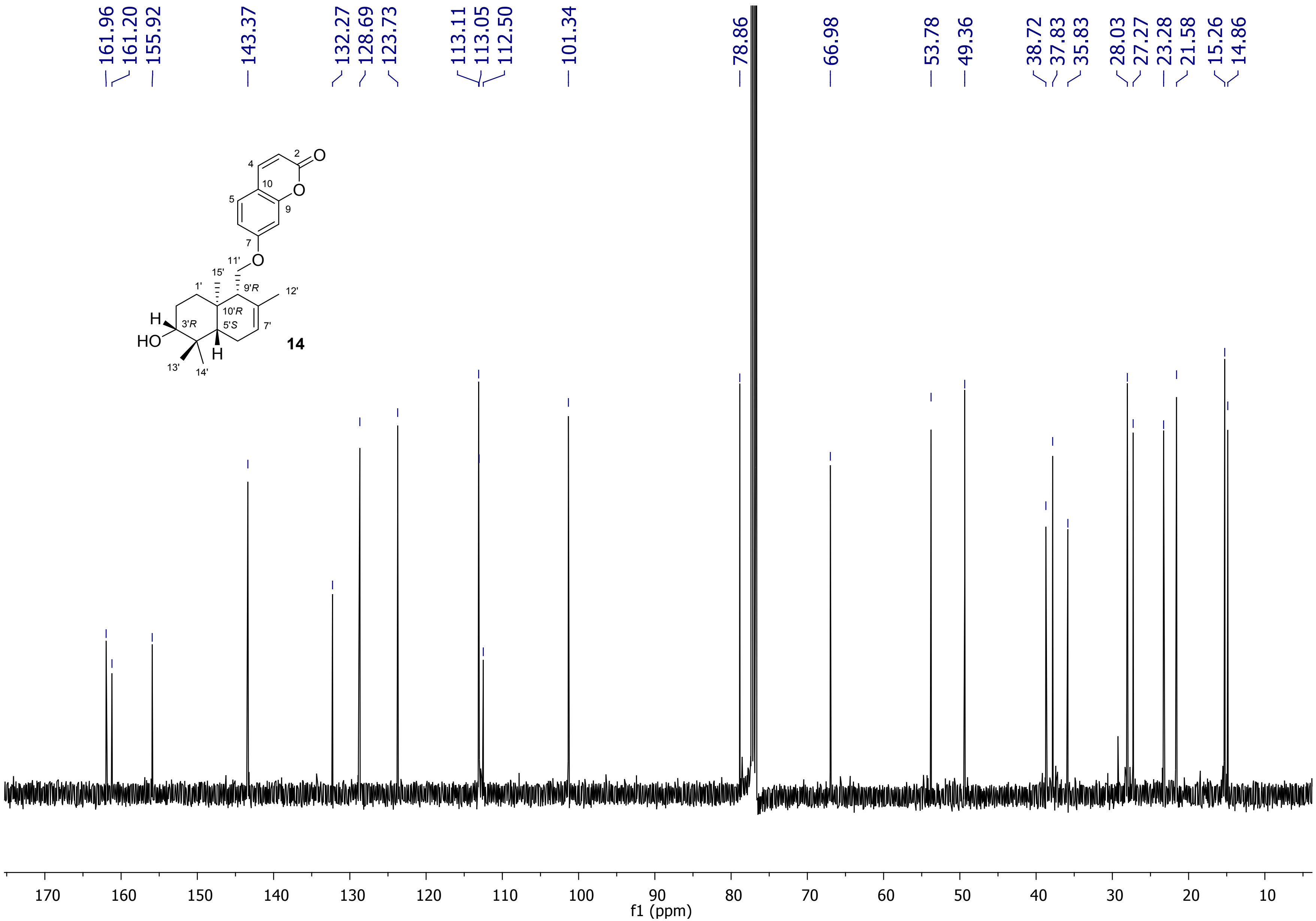

Figure S13b. ^{13}C NMR spectrum (125 MHz) of (±)-7-(3'*R*(*S*),5'*S*(*R*),9'*R*(*S*),10'*R*(*S*)-3'-hydroyidrim-7'-en-11'-yloxy)-coumarin ((±)-feselol) ((±)-**14**)) in $CDCl_3$.

Monoisotopic Mass, Odd and Even Electron Ions
83 formula(e) evaluated with 2 results within limits (up to 50 best isotopic matches for each mass)
Elements Used:
C: 0-500 H: 0-1000 O: 0-200

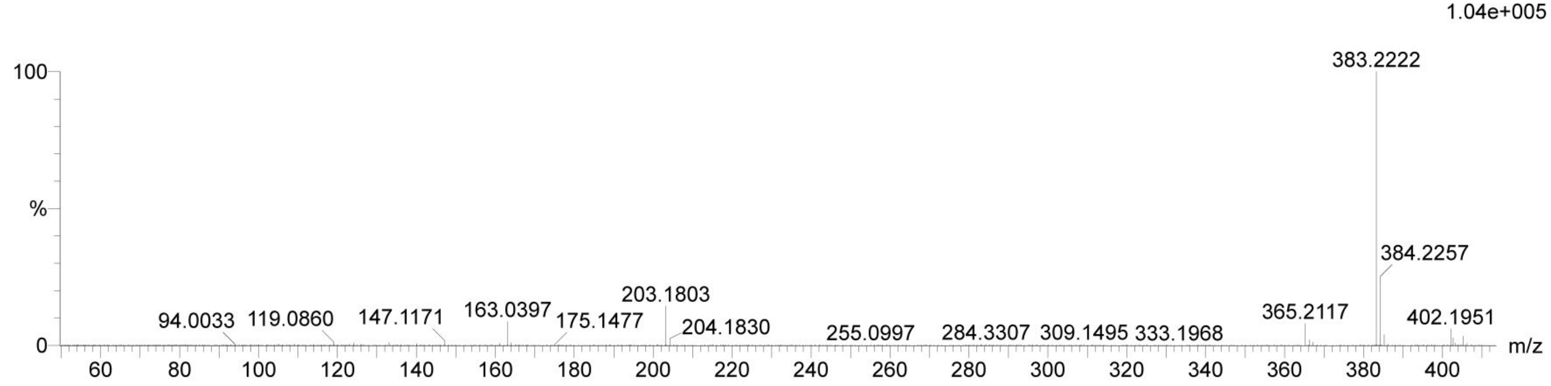

Minimum:	80.00				-1.5				
Maximum:	100.00		10.0	10.0	50.0				
Mass	RA	Calc. Mass	mDa	PPM	DBE	i-FIT	Norm	Conf(%)	Formula
383.2222	100.00	383.2222	0.0	0.0	9.5	115.1	0.000	99.98	C24 H31 O4
		383.2281	-5.9	-15.4	0.5	123.7	8.549	0.02	C17 H35 O9

Figure S13c. HRESIMS of (±)-7-(3'*R*(*S*),5'*S*(*R*),9'*R*(*S*),10'*R*(*S*)-3'-hydroyidrim-7'-en-11'-yloxy)-coumarin ((±)-feselol) ((±)-**14**).

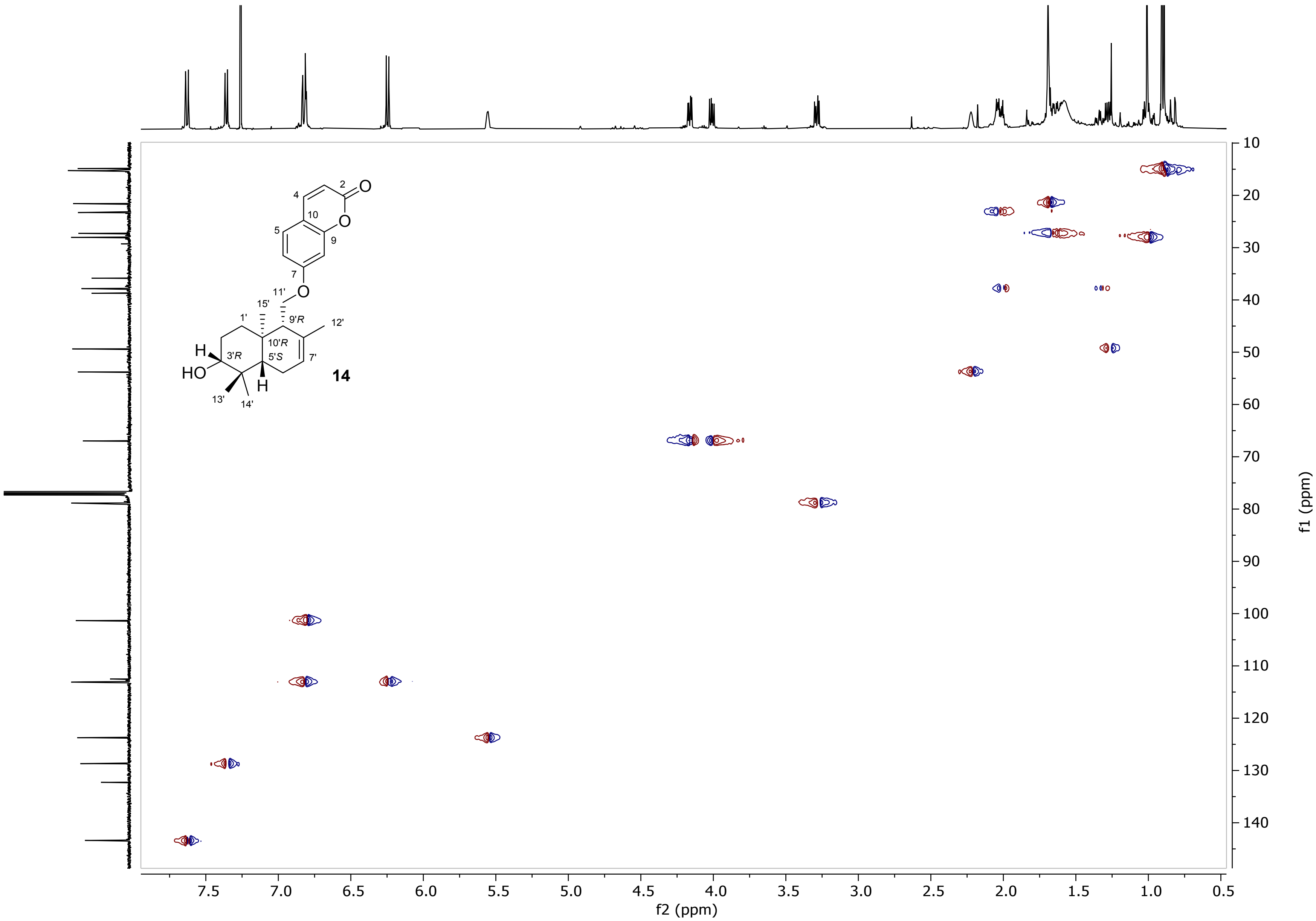

Figure S13d. gHSQC spectrum of (±)-7-(3'*R*(*S*),5'*S*(*R*),9'*R*(*S*),10'*R*(*S*)-3'-hydroxydrim-7'-en-11'-yloxy)-coumarin ((±)-feselol) ((±)-**14**)) in $CDCl_3$.

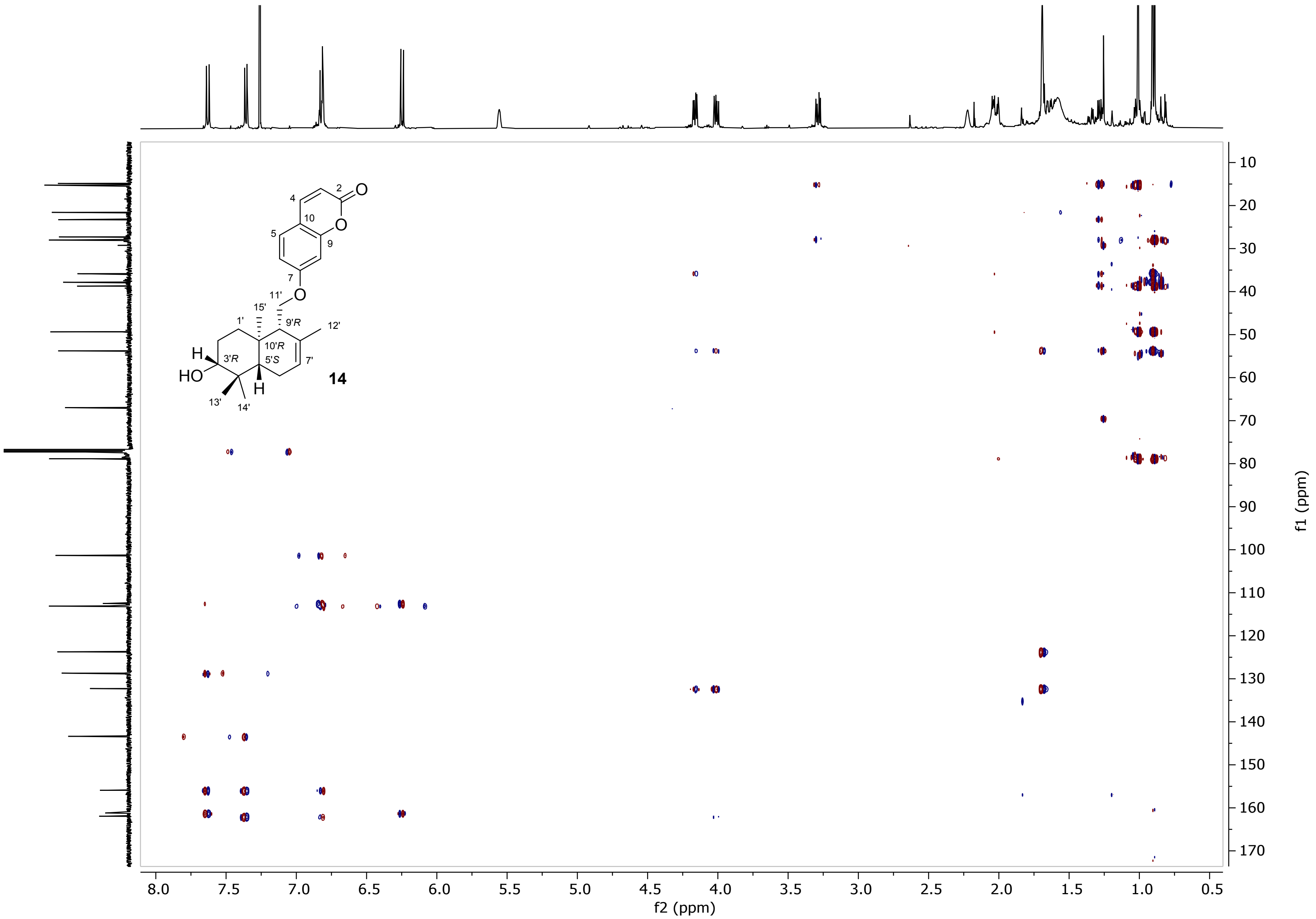

Figure S13e. gHMBC spectrum of (±)-7-(3'*R*(*S*),5'*S*(*R*),9'*R*(*S*),10'*R*(*S*)-3'-hydroxydrim-7'-en-11'-yloxy)-coumarin ((±)-feselol) ((±)-**14**)) in $CDCl_3$.

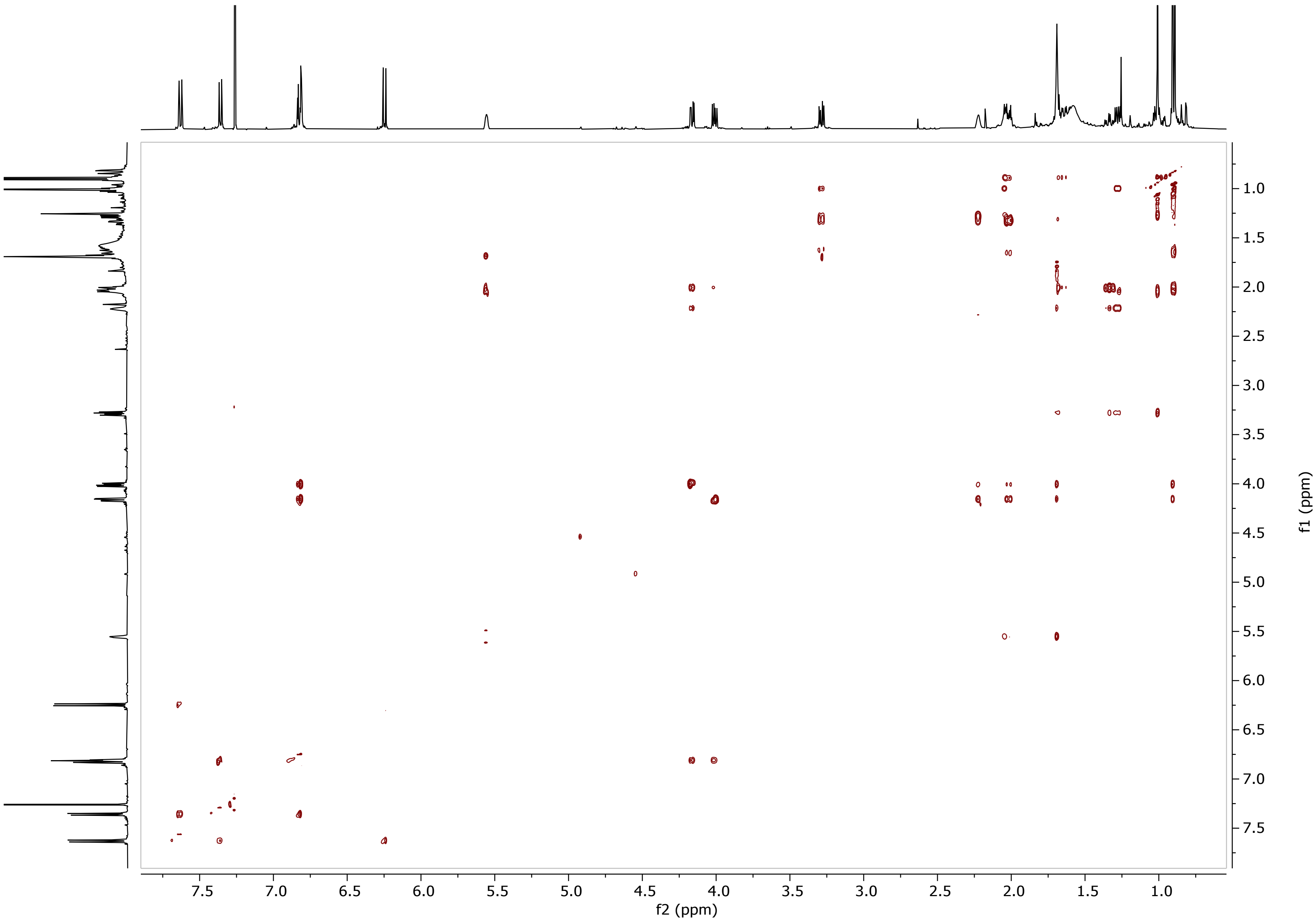

Figure S13f. NOESY2D spectrum of (±)-7-(3'*R*(*S*),5'*S*(*R*),9'*R*(*S*),10'*R*(*S*)-3'-hydroxydrim-7'-en-11'-yloxy)-coumarin ((±)-feselol) ((±)-**14**)) in $CDCl_3$.

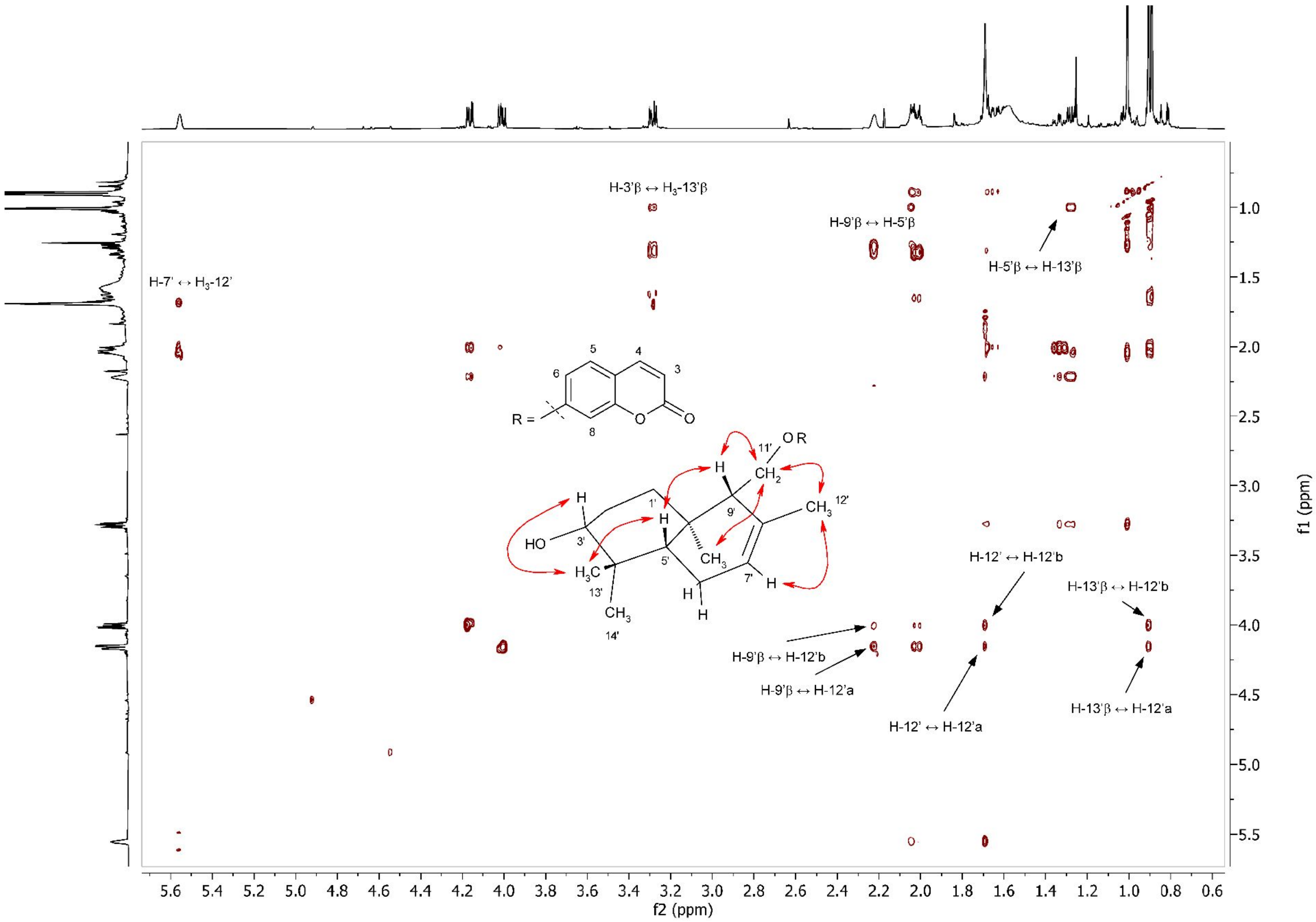

Figure S13g. Expansion of NOESY2D spectrum of (±)-7-(3'*R*(*S*),5'*S*(*R*),9'*R*(*S*),10'*R*(*S*)-3'-hydroxydrim-7'-en-11'-yloxy)-coumarin ((±)-feselol) ((±)-**14**)) in $CDCl_3$. Selected NOESY2D correlations are annotated and highlighted on (±)-feselol ((±)-**14**) structure.

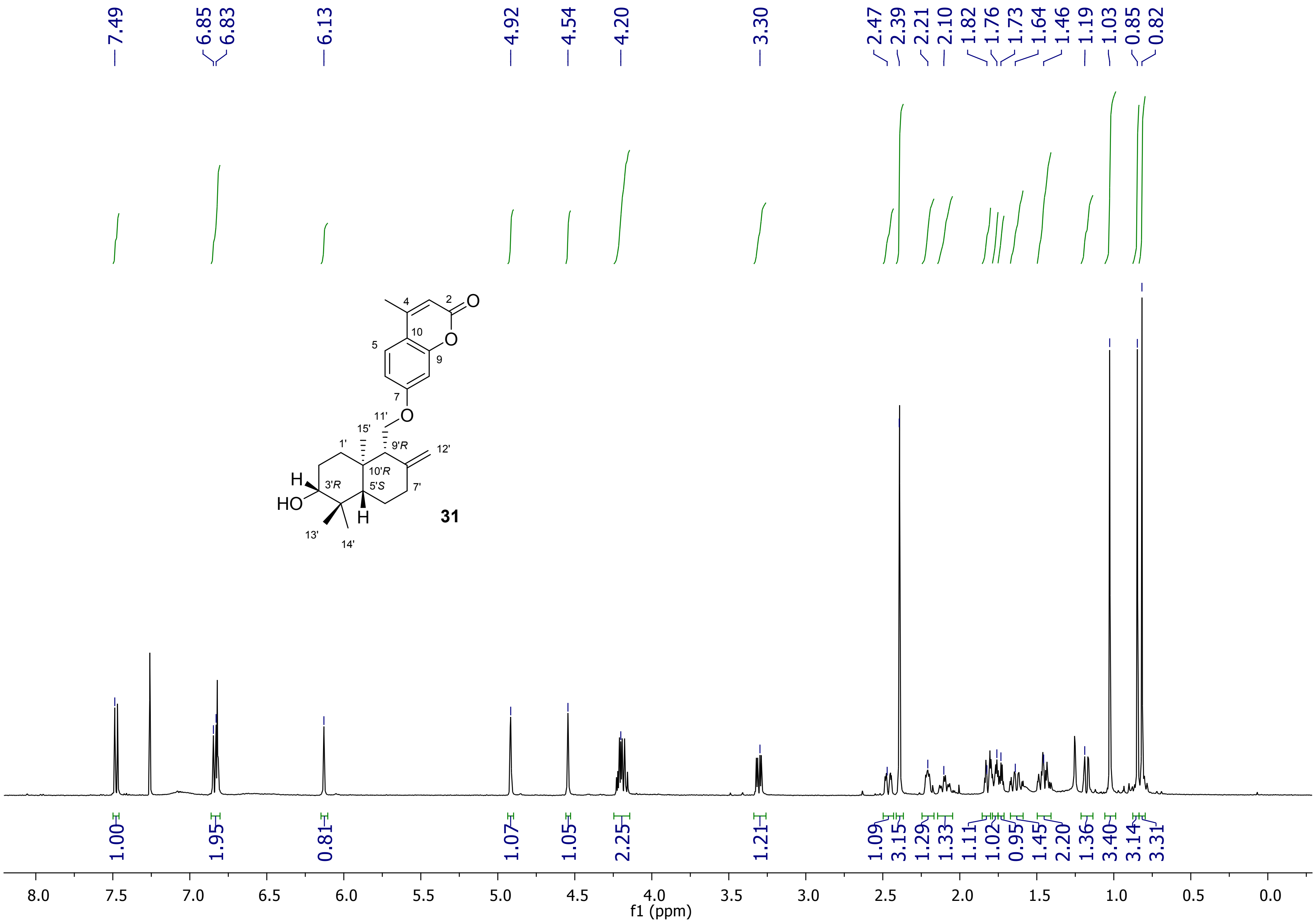

Figure S14a. ^{1}H NMR spectrum (500 MHz) of 7-(3'*R*(*S*),5'*S*(*R*),9'*R*(*S*),10'*R*(*S*)-3'-hydroxydrim-8'(12')-en-11'-yloxy)-4-methycoumarin ((±)-**31**) in $CDCl_3$.

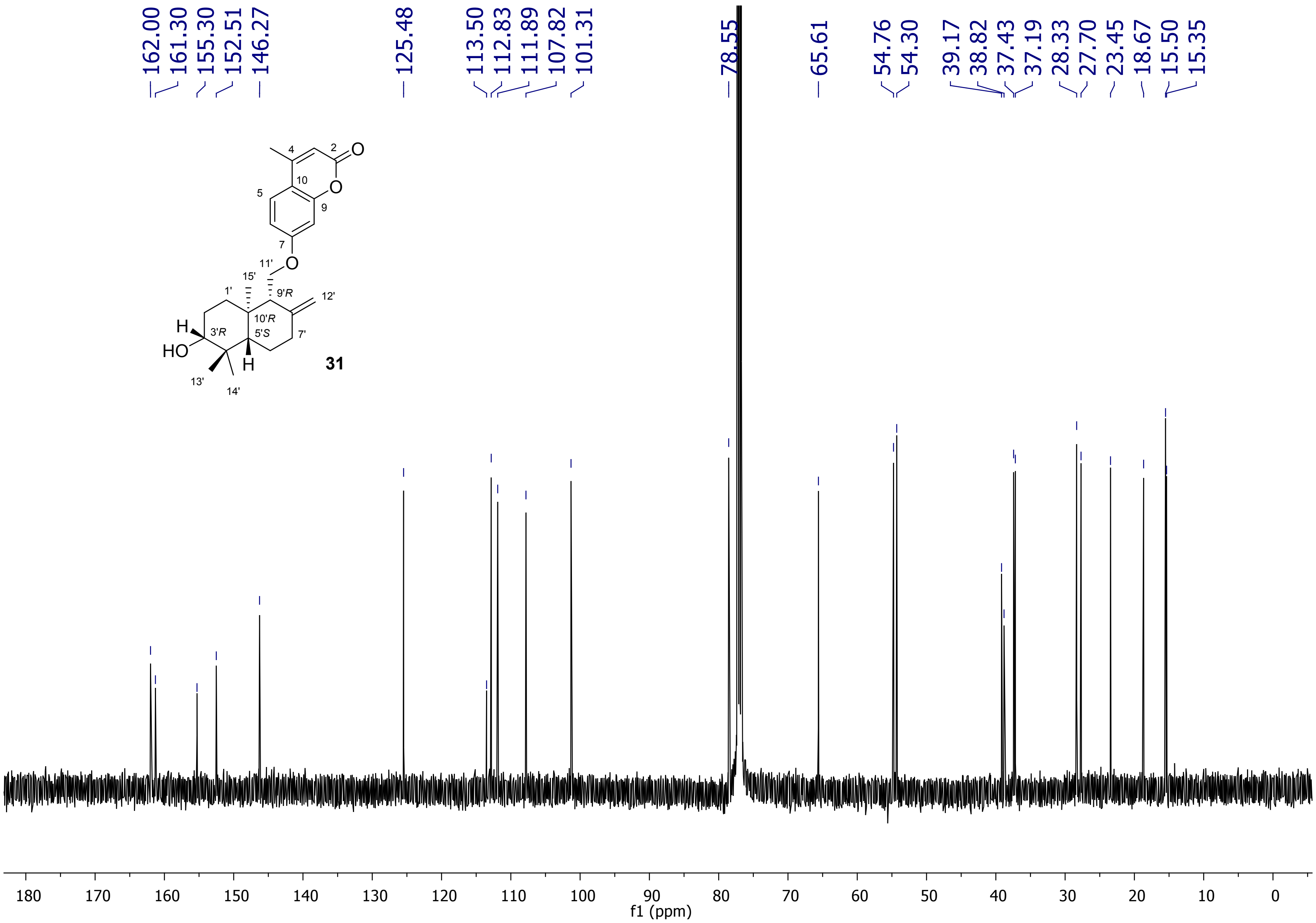

Figure S14b. ^{13}C NMR spectrum (125 MHz) of 7-(3'*R*(*S*),5'*S*(*R*),9'*R*(*S*),10'*R*(*S*)-3'-hydroxydrim-8'(12')-en-11'-yloxy)-4-methycoumarin ((±)-**31**) in $CDCl_3$.

Monoisotopic Mass, Odd and Even Electron Ions
84 formula(e) evaluated with 2 results within limits (up to 50 best isotopic matches for each mass)
Elements Used:
C: 0-500 H: 0-1000 O: 0-200

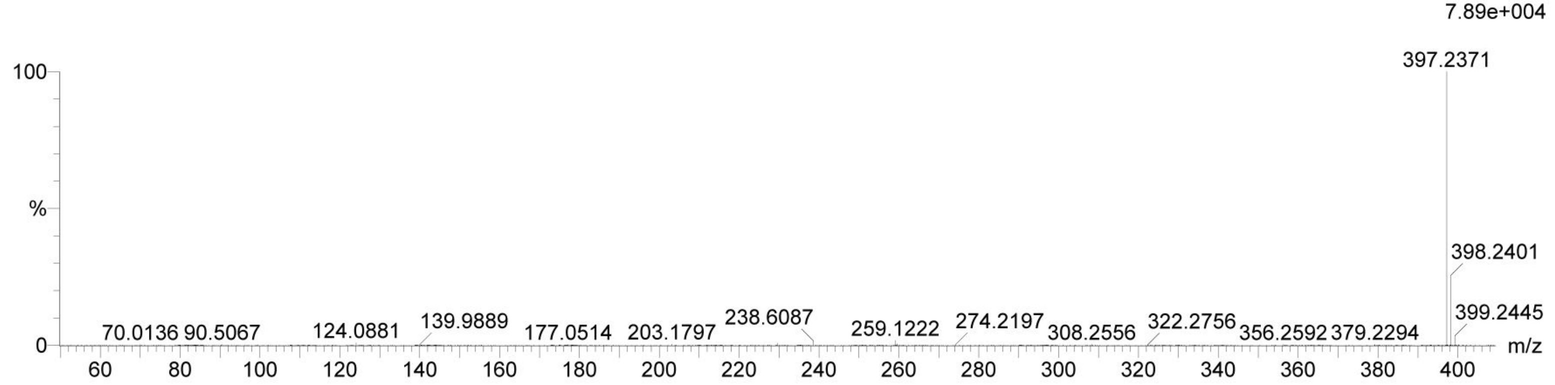

Minimum:	80.00				-1.5				
Maximum:	100.00		10.0	10.0	50.0				
Mass	RA	Calc. Mass	mDa	PPM	DBE	i-FIT	Norm	Conf(%)	Formula
397.2371	100.00	397.2379	-0.8	-2.0	9.5	151.5	0.018	98.23	C25 H33 O4
		397.2438	-6.7	-16.9	0.5	155.5	4.035	1.77	C18 H37 O9

Figure S14c. HRESIMS of 7-(3'*R*(*S*),5'*S*(*R*),9'*R*(*S*),10'*R*(*S*)-3'-hydroxydrim-8'(12')-en-11'-yloxy)-4-methycoumarin ((±)-**31**).

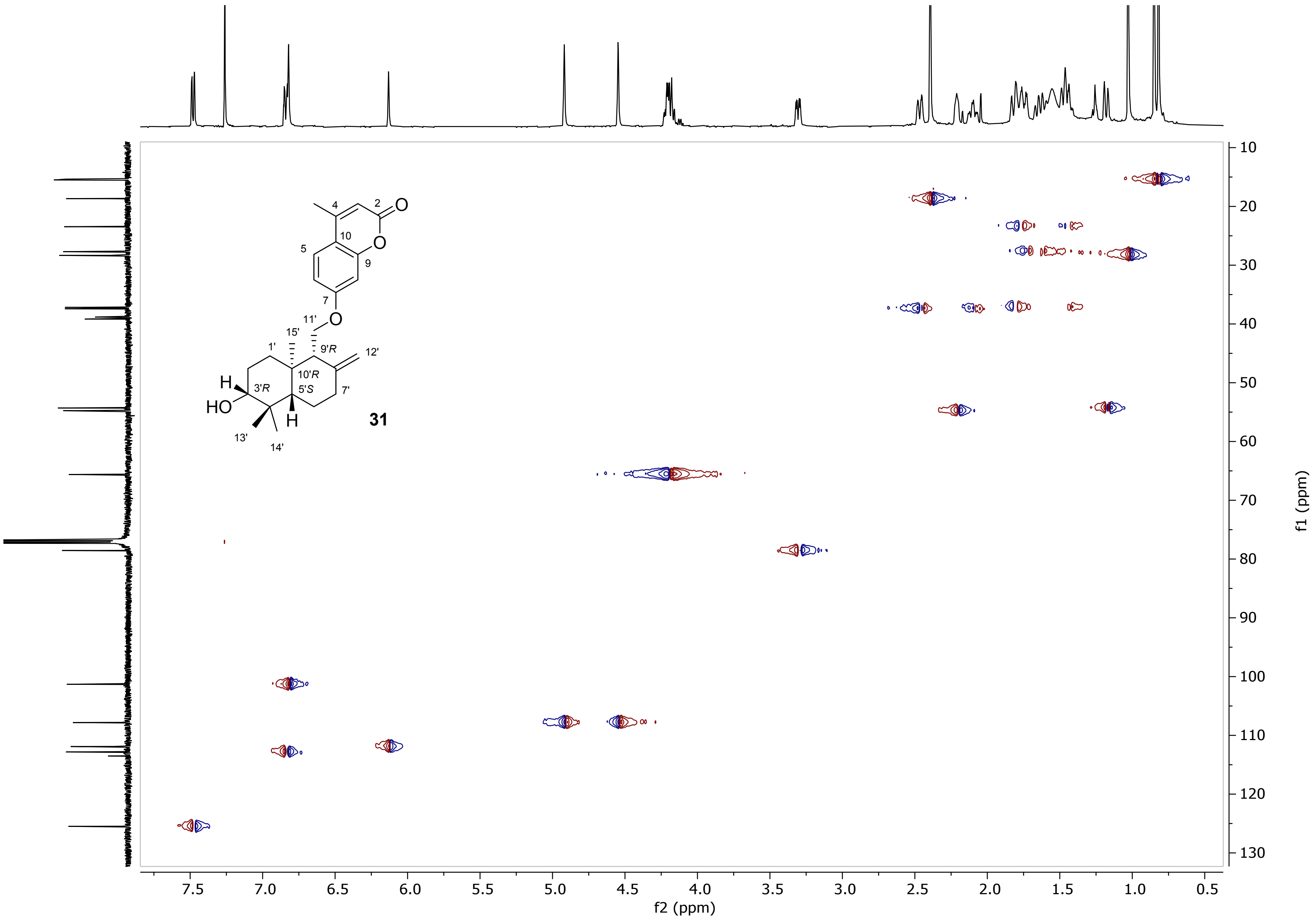

Figure S14d. gHSQC spectrum of 7-(3'*R*(*S*),5'*S*(*R*),9'*R*(*S*),10'*R*(*S*)-3'-hydroxydrim-8'(12')-en-11'-yloxy)-4-methycoumarin ((±)-**31**) in $CDCl_3$.

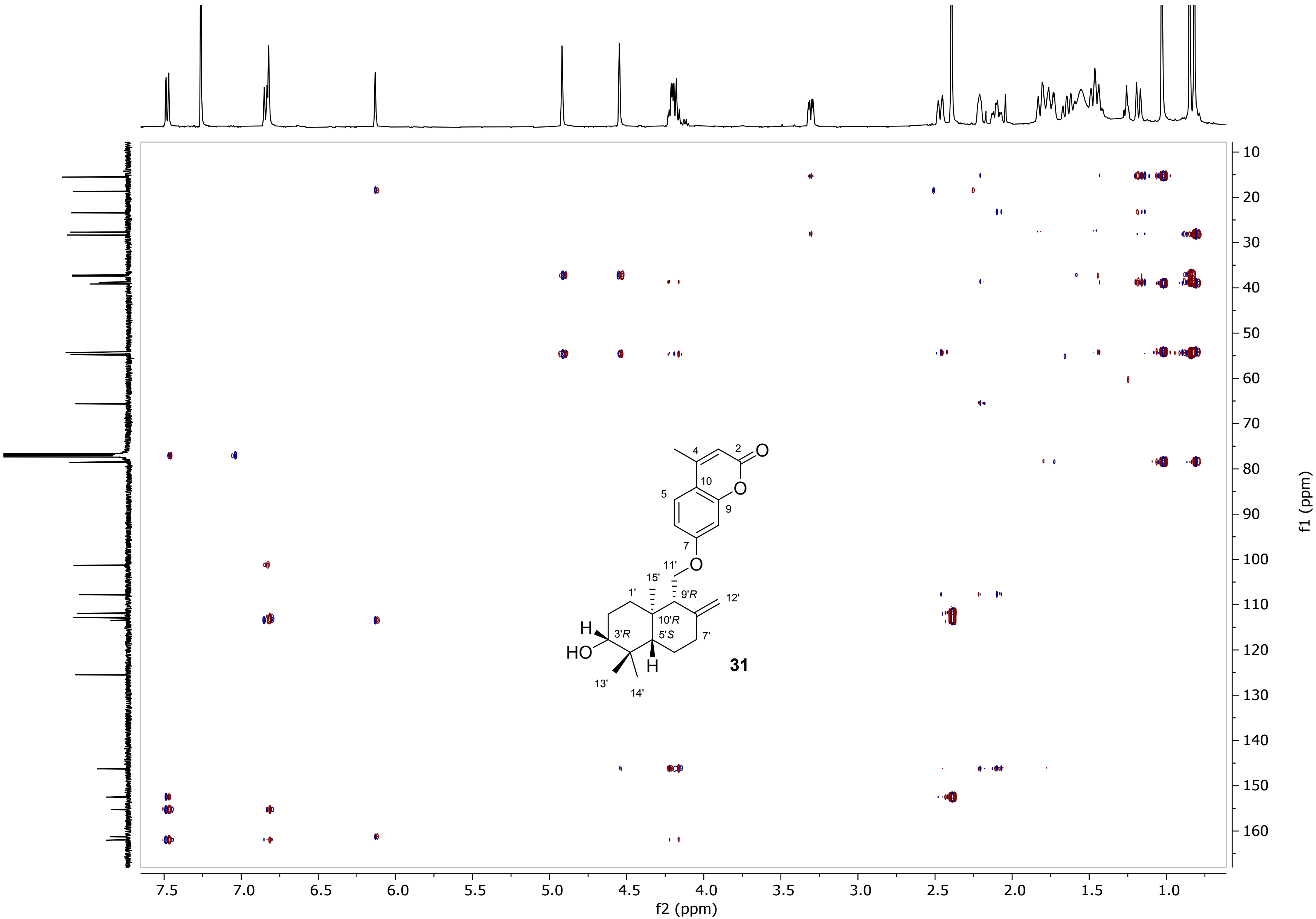

Figure S14e. gHMBC spectrum of 7-(3'*R*(*S*),5'*S*(*R*),9'*R*(*S*),10'*R*(*S*)-3'-hydroxydrim-8'(12')-en-11'-yloxy)-4-methycoumarin ((±)-**31**) in $CDCl_3$.

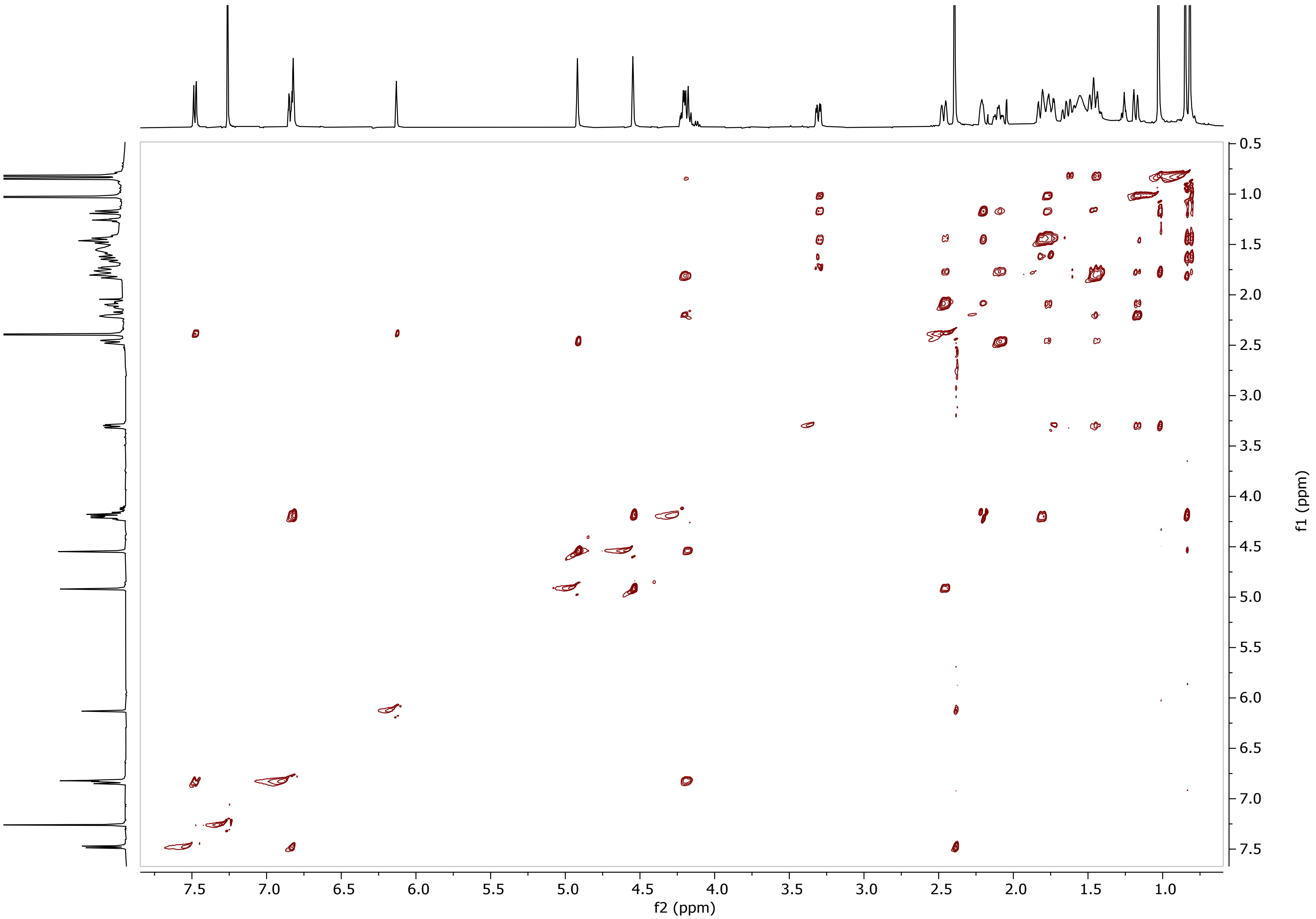

Figure S14f. NOESY2D spectrum of 7-(3'*R*(*S*),5'*S*(*R*),9'*R*(*S*),10'*R*(*S*)-3'-hydroxydrim-8'(12')-en-11'-yloxy)-4-methycoumarin ((±)-**31**) in $CDCl_3$.

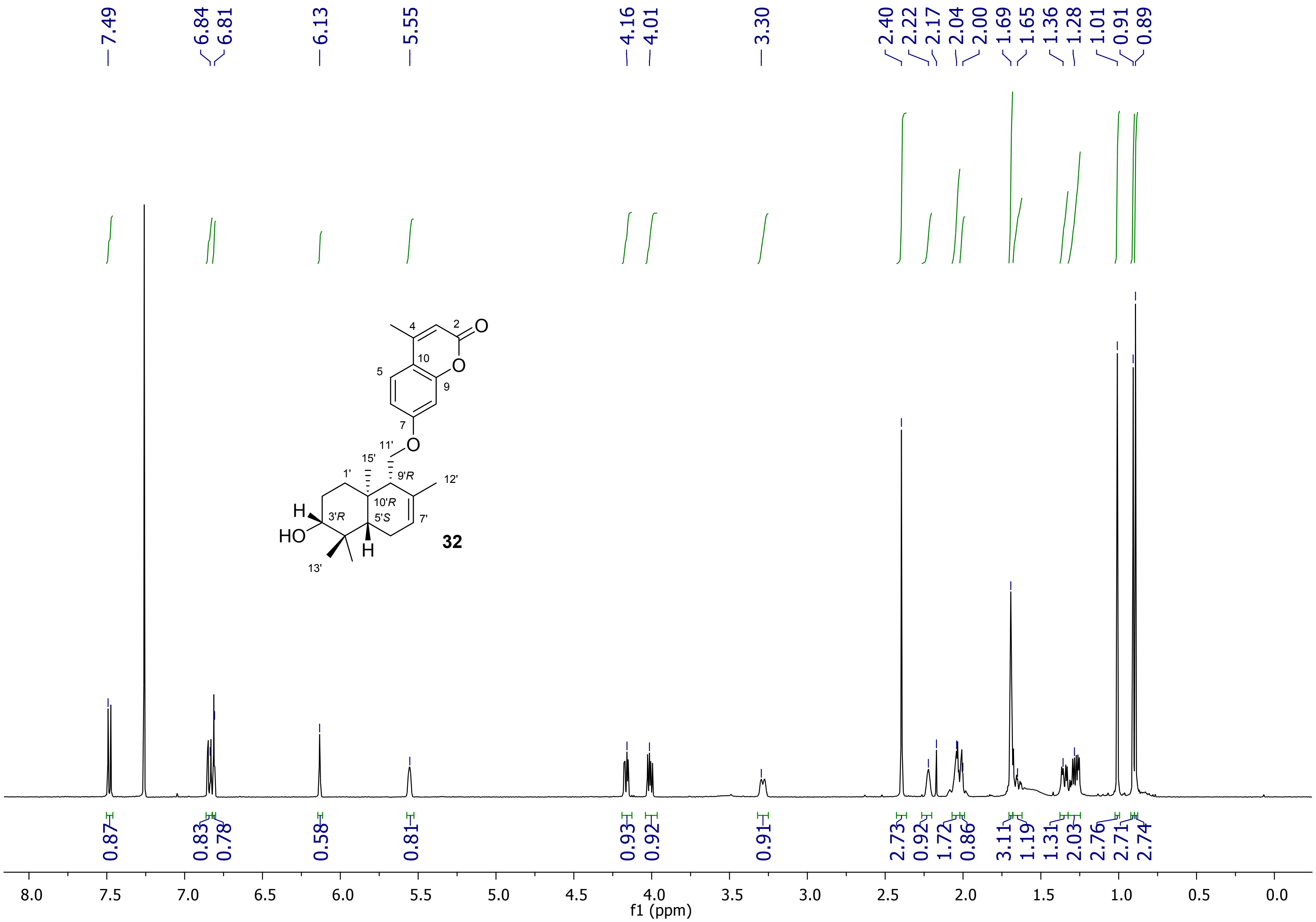

Figure S15a. ^{1}H NMR spectrum (500 MHz) of 7-(3'*R*(*S*),5'*S*(*R*),9'*R*(*S*),10'*R*(*S*)-3'-hydroxydrim-7'-en-11'-yloxy)-4-methylcoumarin ((±)-**32**) in $CDCl_3$.

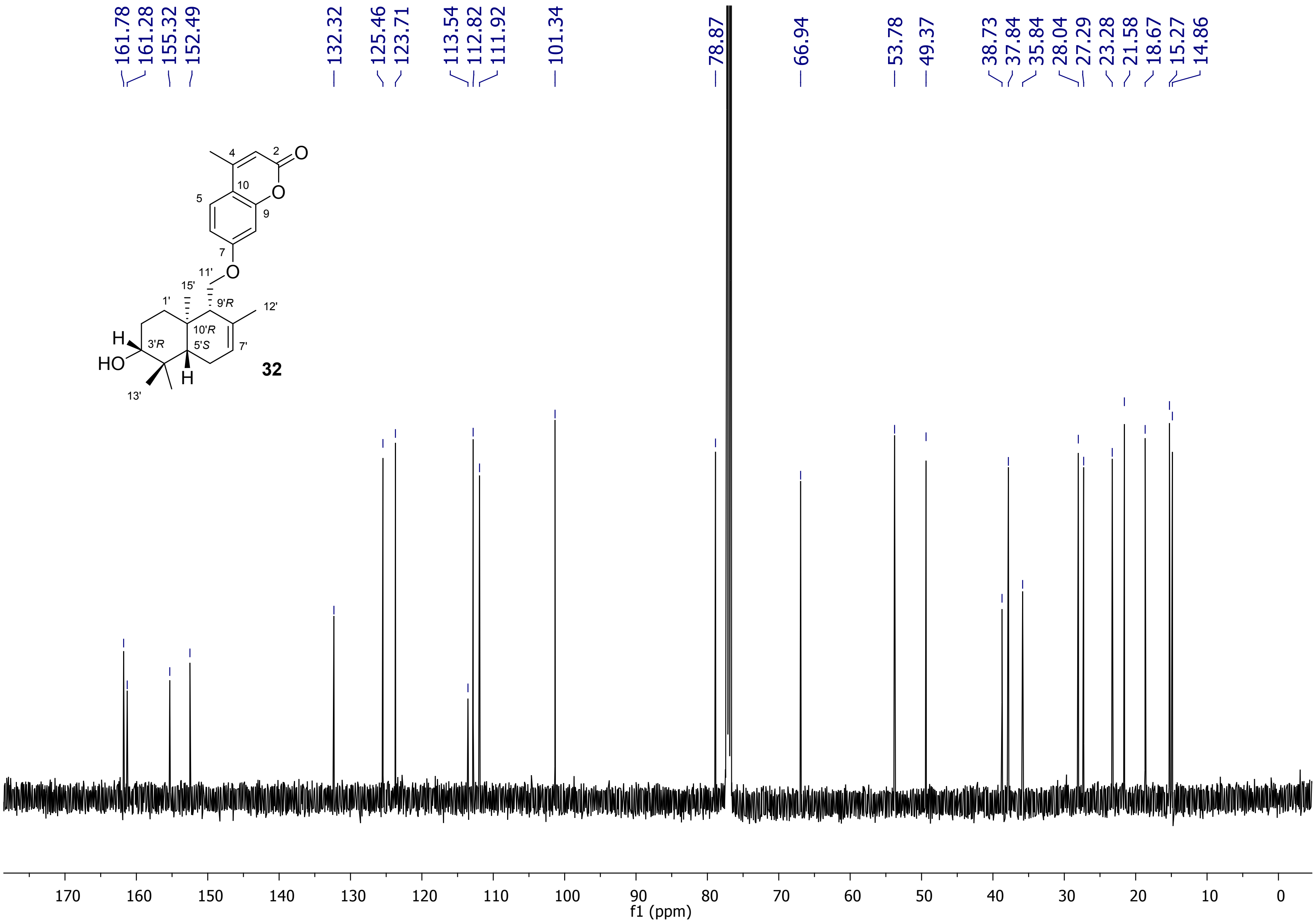

Figure S15b. ^{13}C NMR spectrum (125 MHz) of 7-(3'*R*(*S*),5'*S*(*R*),9'*R*(*S*),10'*R*(*S*)-3'-hydroxydrim-7'-en-11'-yloxy)-4-methylcoumarin ((±)-**32**) in $CDCl_3$.

Monoisotopic Mass, Odd and Even Electron Ions
84 formula(e) evaluated with 2 results within limits (up to 50 best isotopic matches for each mass)
Elements Used:
C: 0-500 H: 0-1000 O: 0-200

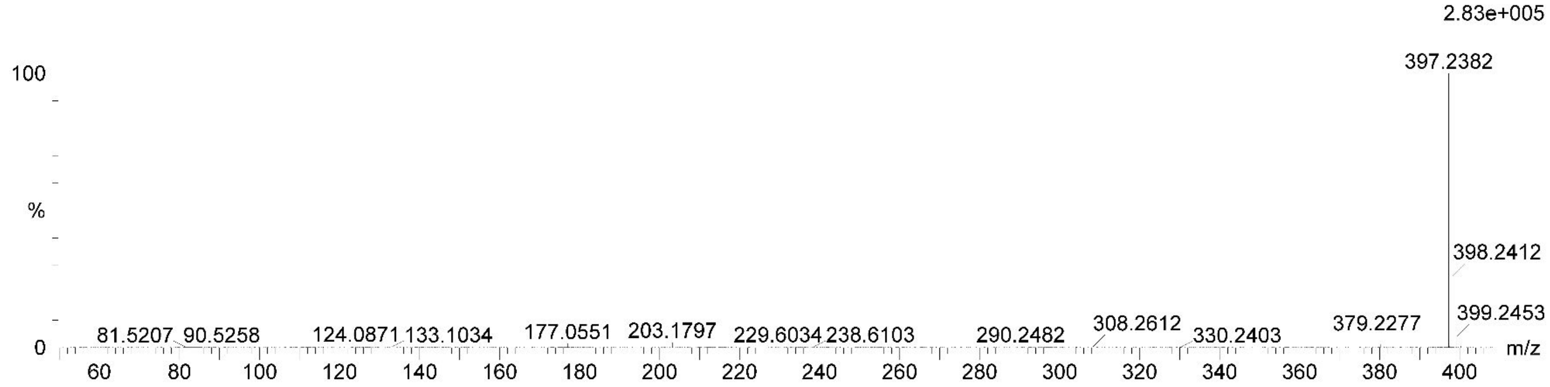

Minimum:	80.00				-1.5				
Maximum:	100.00		10.0	10.0	50.0				
Mass	RA	Calc. Mass	mDa	PPM	DBE	i-FIT	Norm	Conf(%)	Formula
397.2382	100.00	397.2379	0.3	0.8	9.5	265.3	0.001	99.93	C25 H33 O4
		397.2438	-5.6	-14.1	0.5	272.5	7.212	0.07	C18 H37 O9

Figure S15c. HRESIMS of 7-(3'*R*(*S*),5'*S*(*R*),9'*R*(*S*),10'*R*(*S*)-3'-hydroxydrim-7'-en-11'-yloxy)-4-methylcoumarin ((±)-**32**).

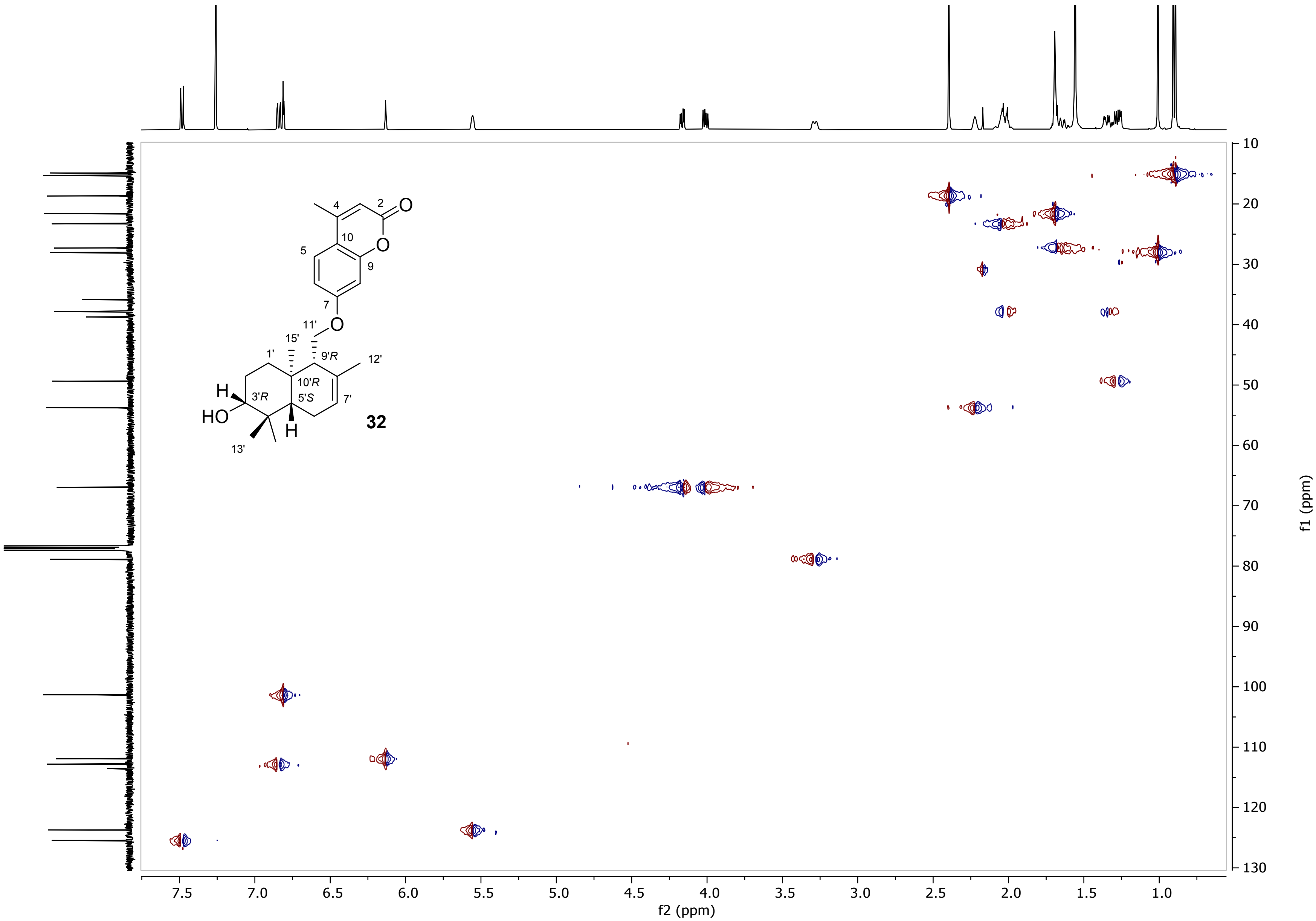

Figure S15d. gHSQC spectrum of 7-(3'*R*(*S*),5'*S*(*R*),9'*R*(*S*),10'*R*(*S*)-3'-hydroxydrim-7'-en-11'-yloxy)-4-methylcoumarin ((±)-**32**) in $CDCl_3$.

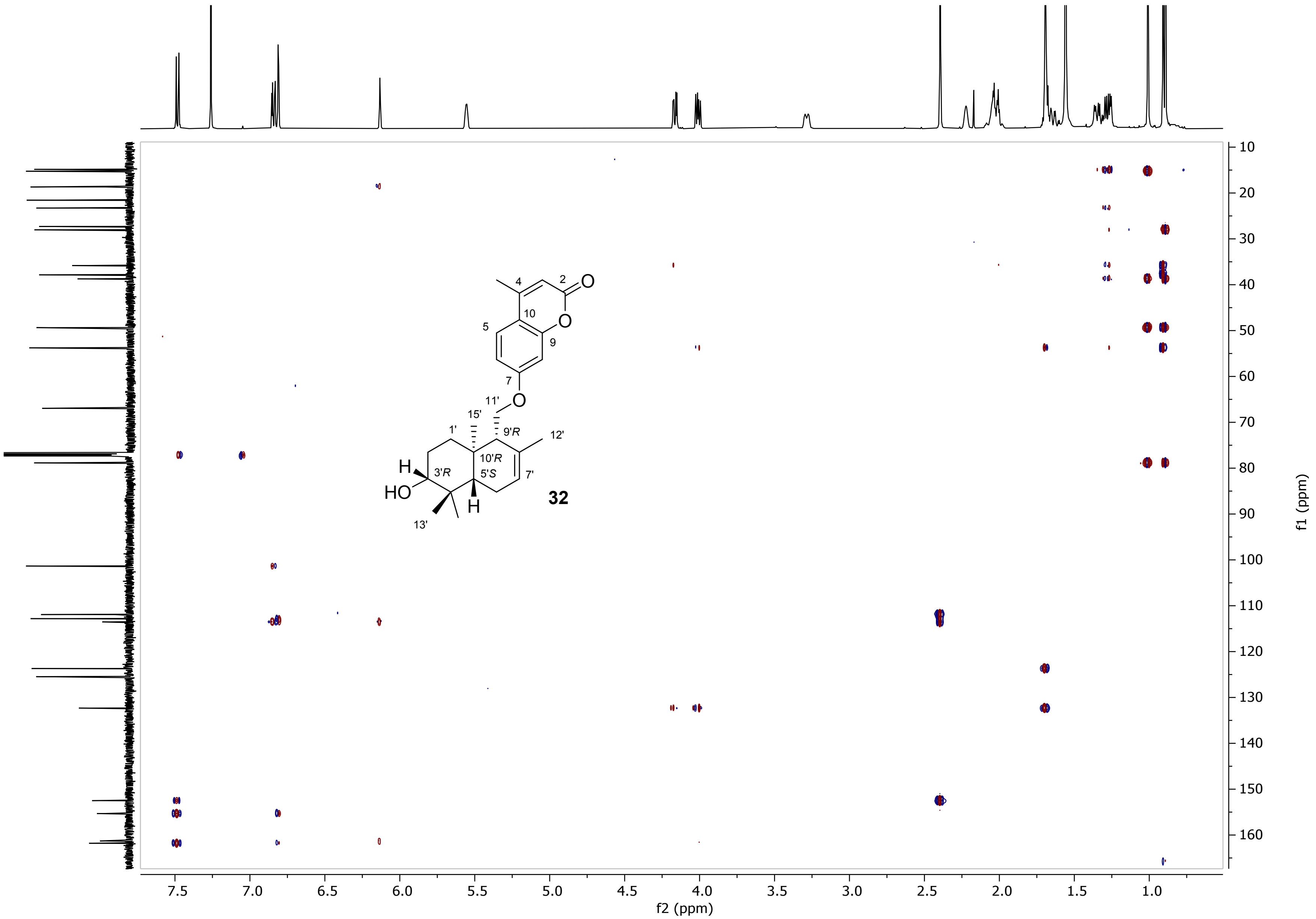

Figure S15e. gHMBC spectrum of 7-(3'*R*(*S*),5'*S*(*R*),9'*R*(*S*),10'*R*(*S*)-3'-hydroxydrim-7'-en-11'-yloxy)-4-methylcoumarin ((±)-**32**) in $CDCl_3$.

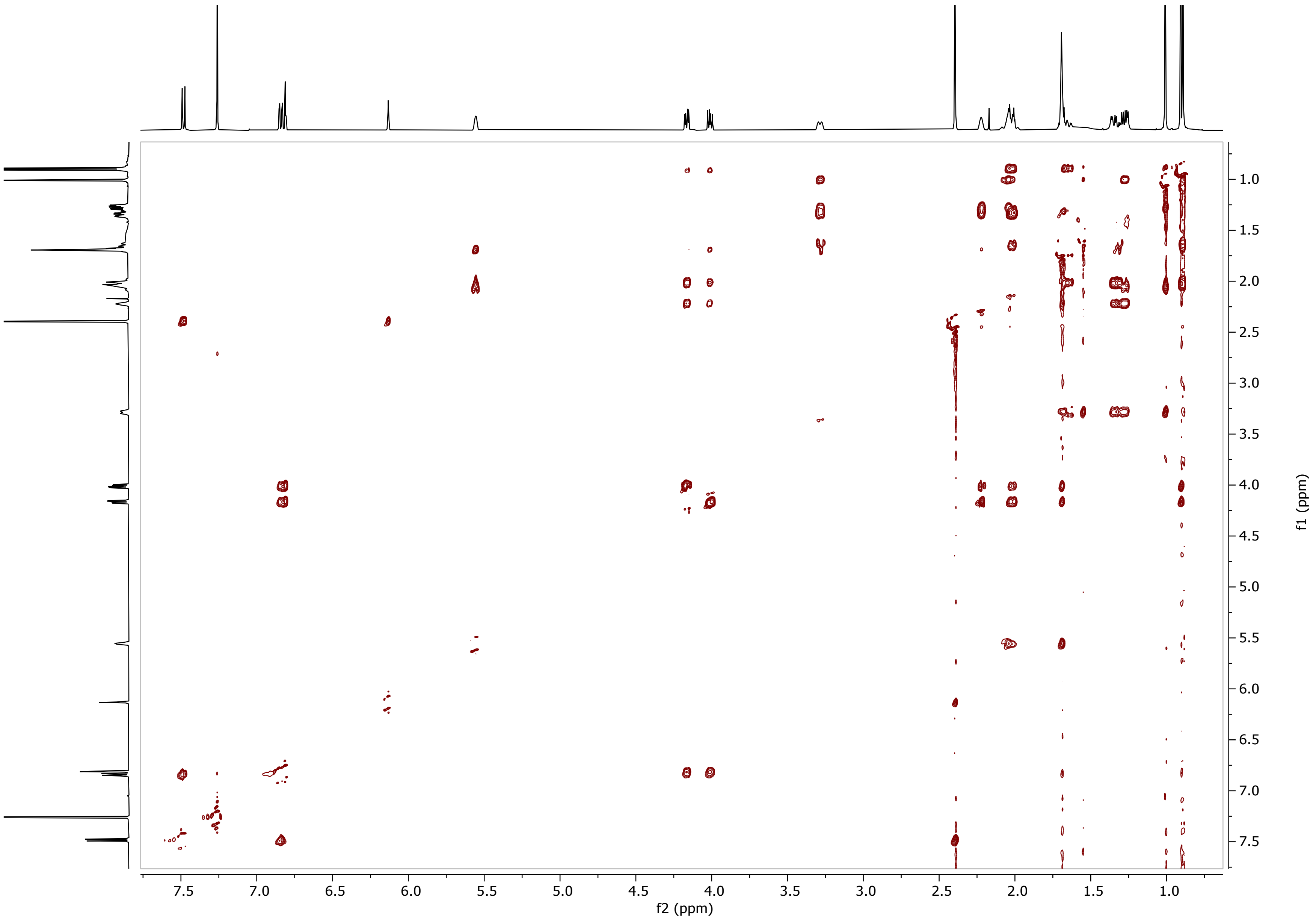

Figure S15f. NOESY2D spectrum of 7-(3'*R*(*S*),5'*S*(*R*),9'*R*(*S*),10'*R*(*S*)-3'-hydroxydrim-7'-en-11'-yloxy)-4-methylcoumarin ((±)-**32**) in $CDCl_3$.

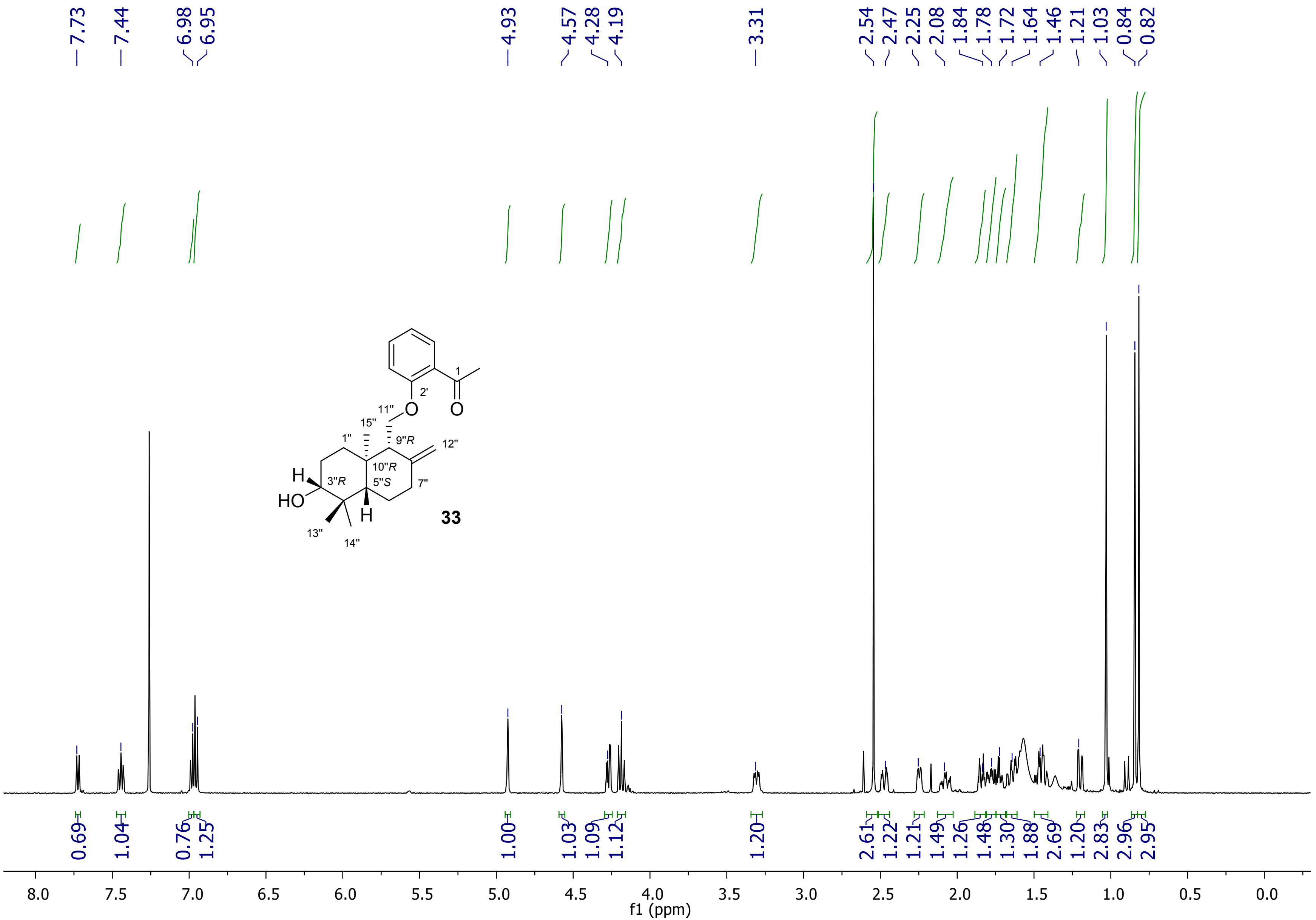

Figure S16a. ^{1}H NMR spectrum (500 MHz) of 1-(2'-(3''*R*(*S*),5''*S*(*R*),9''*R*(*S*),10''*R*(*S*)-3''-hydroxydrim-8''(12'')-en-11''-yloxy)phenyl)ethanone ((±)-**33**) in $CDCl_3$.

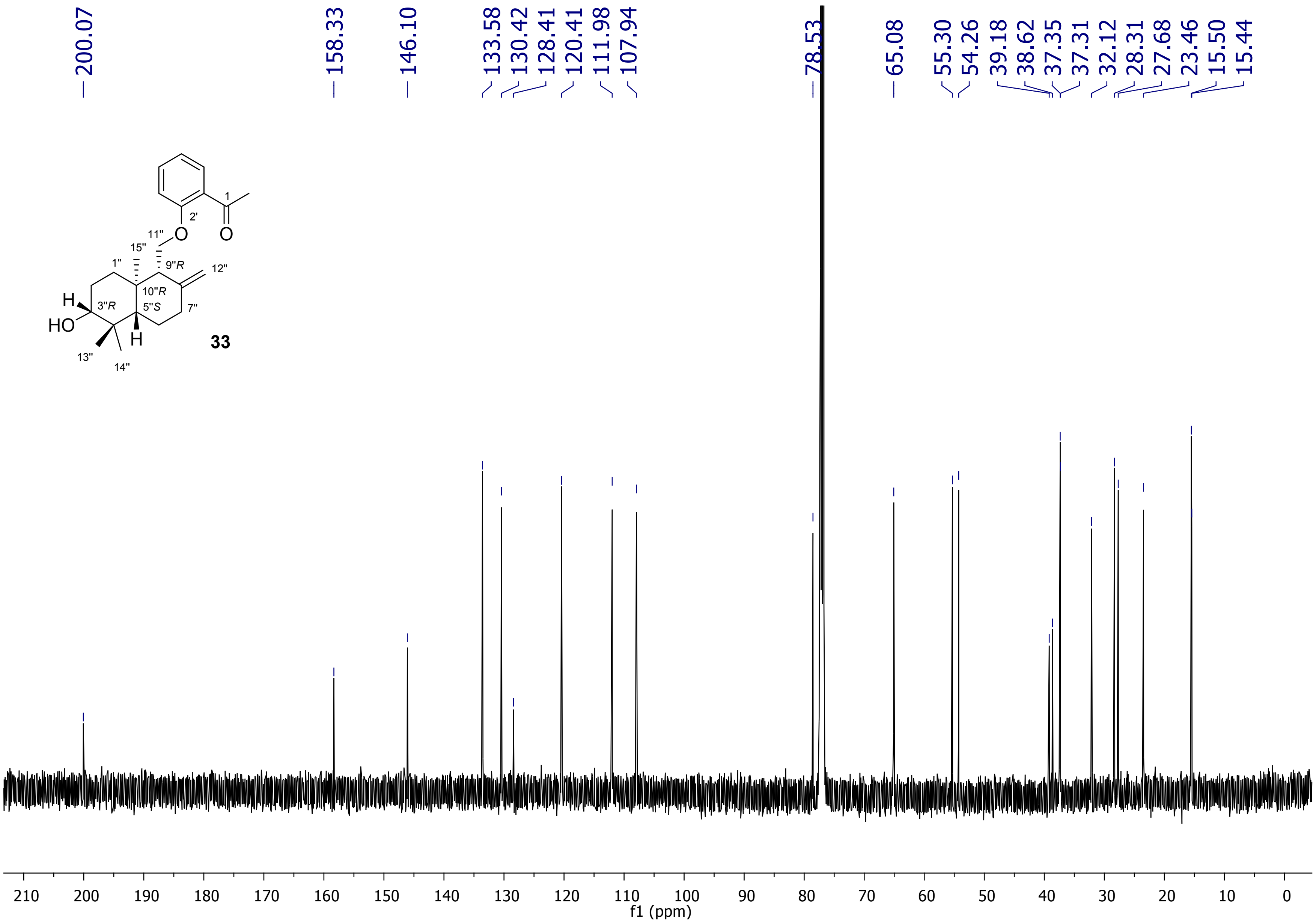

Figure S16b. ^{13}C NMR spectrum (125 MHz) of 1-(2'-(3''*R*(*S*),5''*S*(*R*),9''*R*(*S*),10''*R*(*S*)-3''-hydroxydrim-8''(12'')-en-11''-yloxy)phenyl)ethanone ((±)-**33**) in $CDCl_3$.

Monoisotopic Mass, Even Electron Ions
131 formula(e) evaluated with 2 results within limits (up to 10 closest results for each mass)
Elements Used:
C: 1-500 H: 0-1000 O: 0-200 Na: 0-1

Minimum:				-1.5				
Maximum:		5.0	10.0	50.0				
Mass	Calc. Mass	mDa	PPM	DBE	i-FIT	Norm	Conf(%)	Formula
357.2435	357.2430	0.5	1.4	7.5	108.7	1.229	29.25	C23 H33 O3
	357.2406	2.9	8.1	4.5	107.8	0.346	70.75	C21 H34 O3 Na

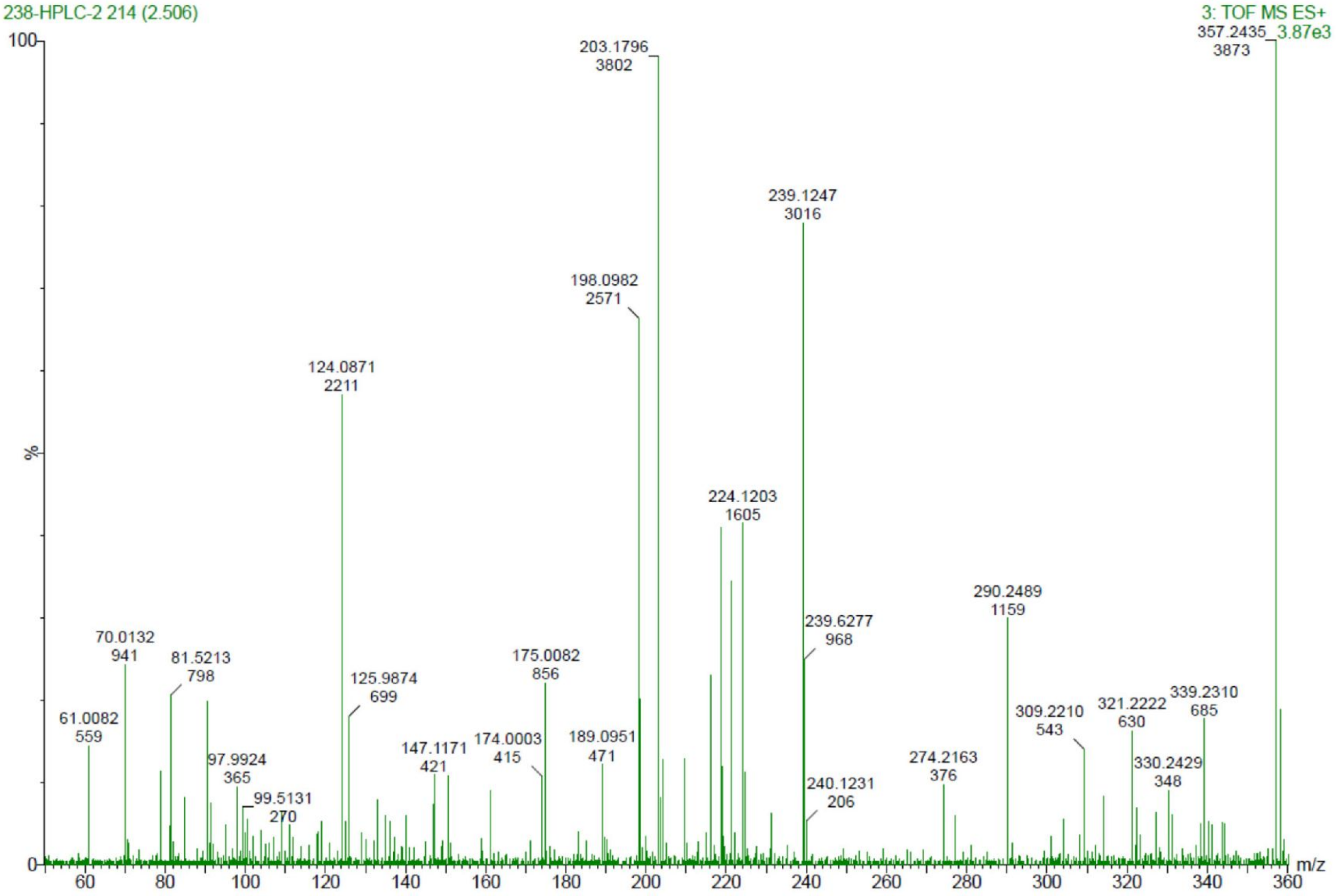

Figure S16c. HRESIMS of 1-(2'-(3''*R*(*S*),5''*S*(*R*),9''*R*(*S*),10''*R*(*S*)-3''-hydroxydrim-8''(12'')-en-11''-yloxy)phenyl)ethanone ((±)-**33**) in $CDCl_3$.

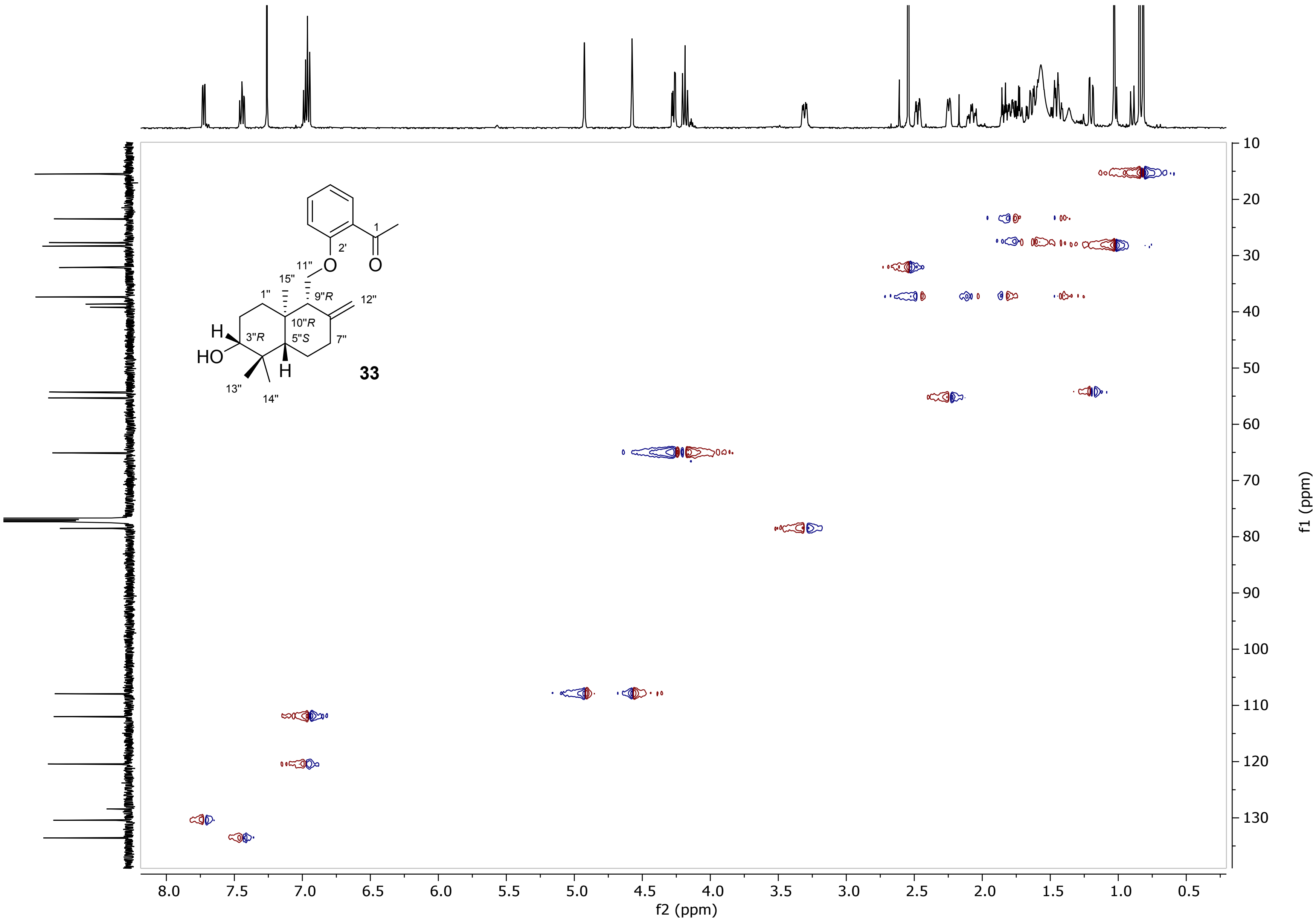

Figure S16d. gHSQC spectrum of 1-(2'-(3''*R*(*S*),5''*S*(*R*),9''*R*(*S*),10''*R*(*S*)-3''-hydroxydrim-8''(12'')-en-11''-yloxy)phenyl)ethanone ((±)-**33**) in $CDCl_3$.

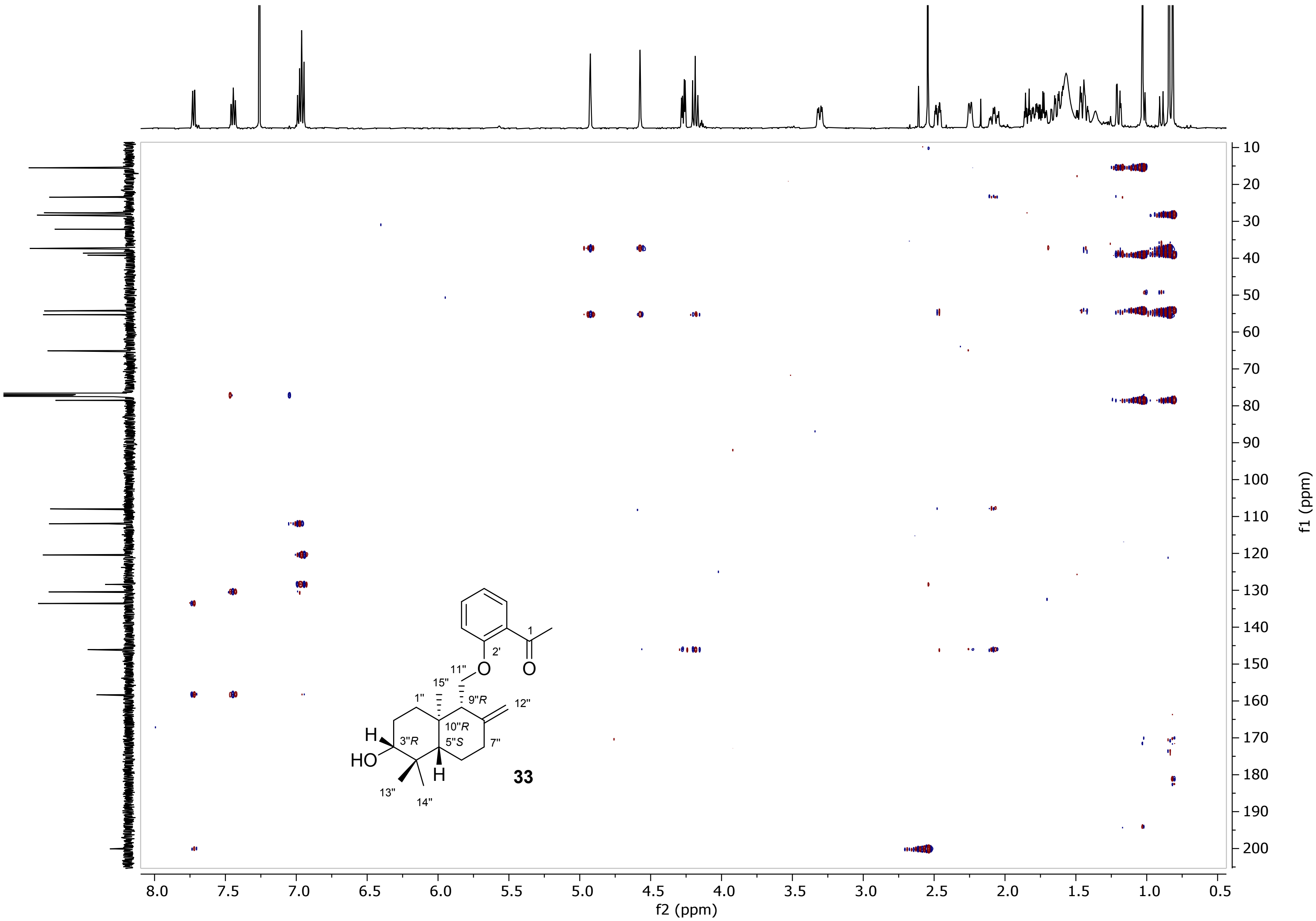

Figure S16e. gHMBC spectrum of 1-(2'-(3"*R*(*S*),5"*S*(*R*),9"*R*(*S*),10"*R*(*S*)-3"-hydroxydrim-8"(12")-en-11"-yloxy)phenyl)ethanone ((±)-**33**) in $CDCl_3$.

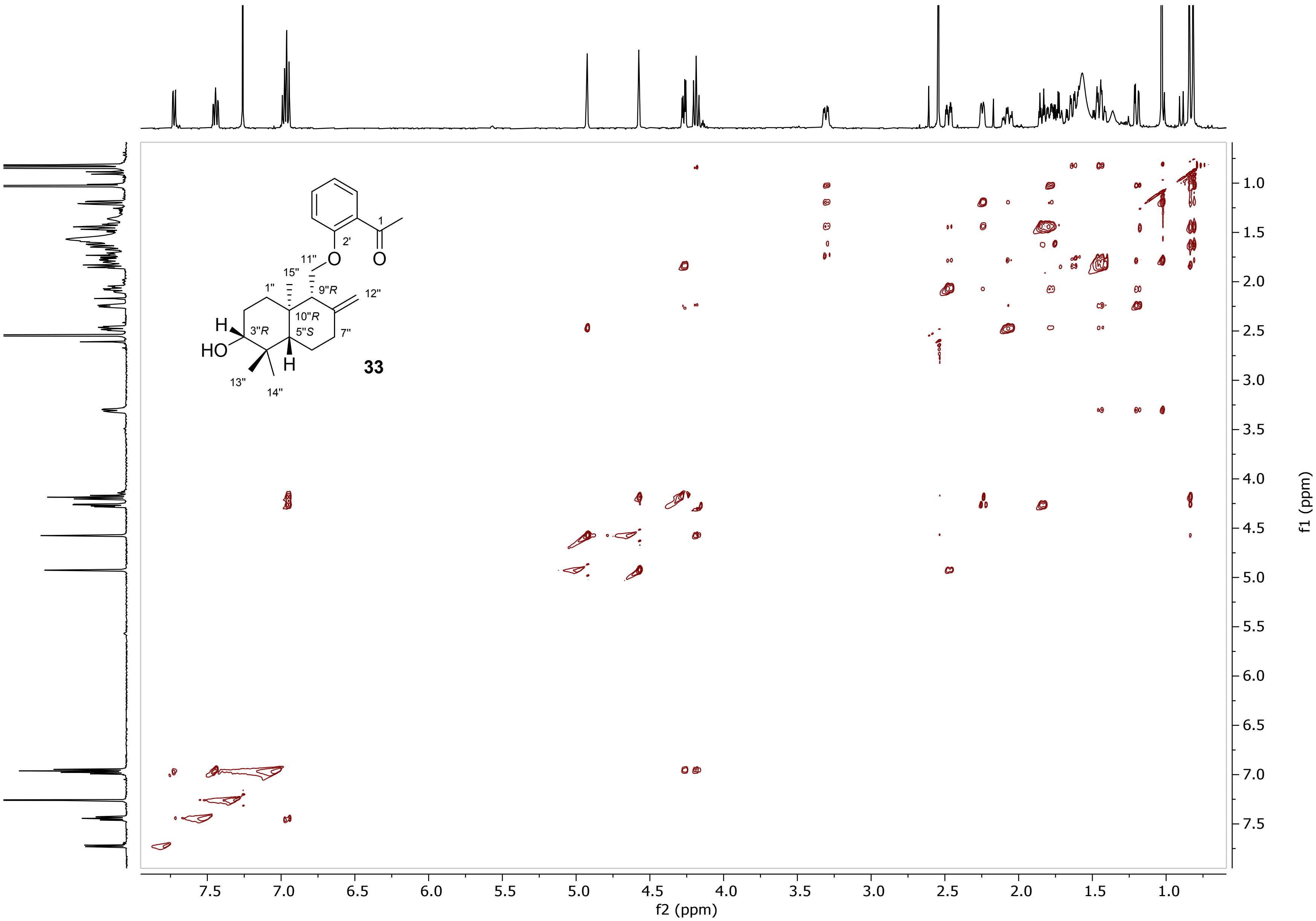

Figure S16f. NOESY2D spectrum of 1-(2'-(3''*R*(*S*),5''*S*(*R*),9''*R*(*S*),10''*R*(*S*)-3''-hydroxydrim-8''(12'')-en-11''-yloxy)phenyl)ethanone ((±)-**33**) in $CDCl_3$.

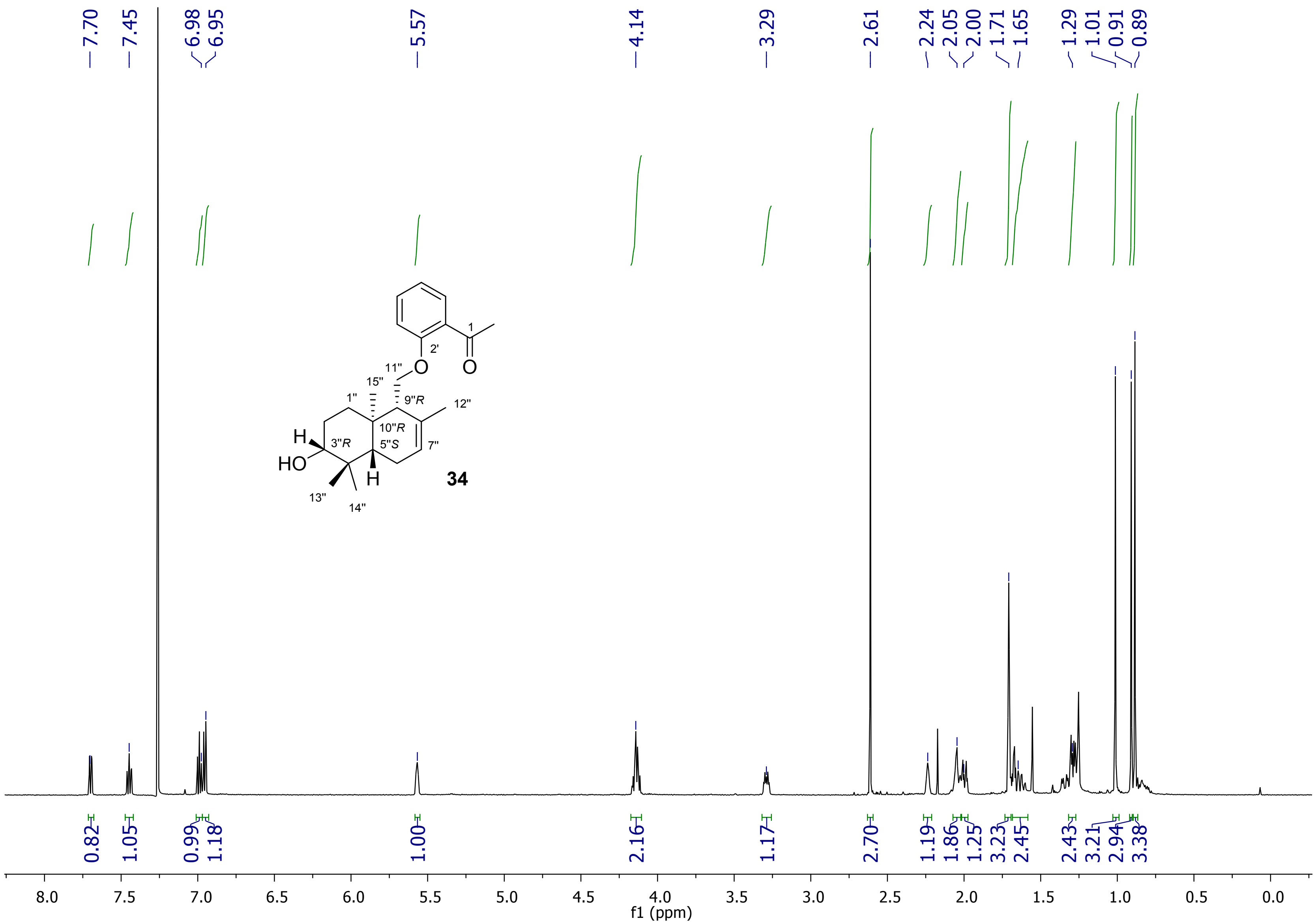

Figure S17a. ^{1}H NMR spectrum (500 MHz) of 1-(2’-(3"*R*(*S*),5"*S*(*R*),9"*R*(*S*),10"*R*(*S*)-3"-hydroxydrim-7"-en-11"-yloxy)phenyl)ethanone ((±)-**34**) in $CDCl_3$.

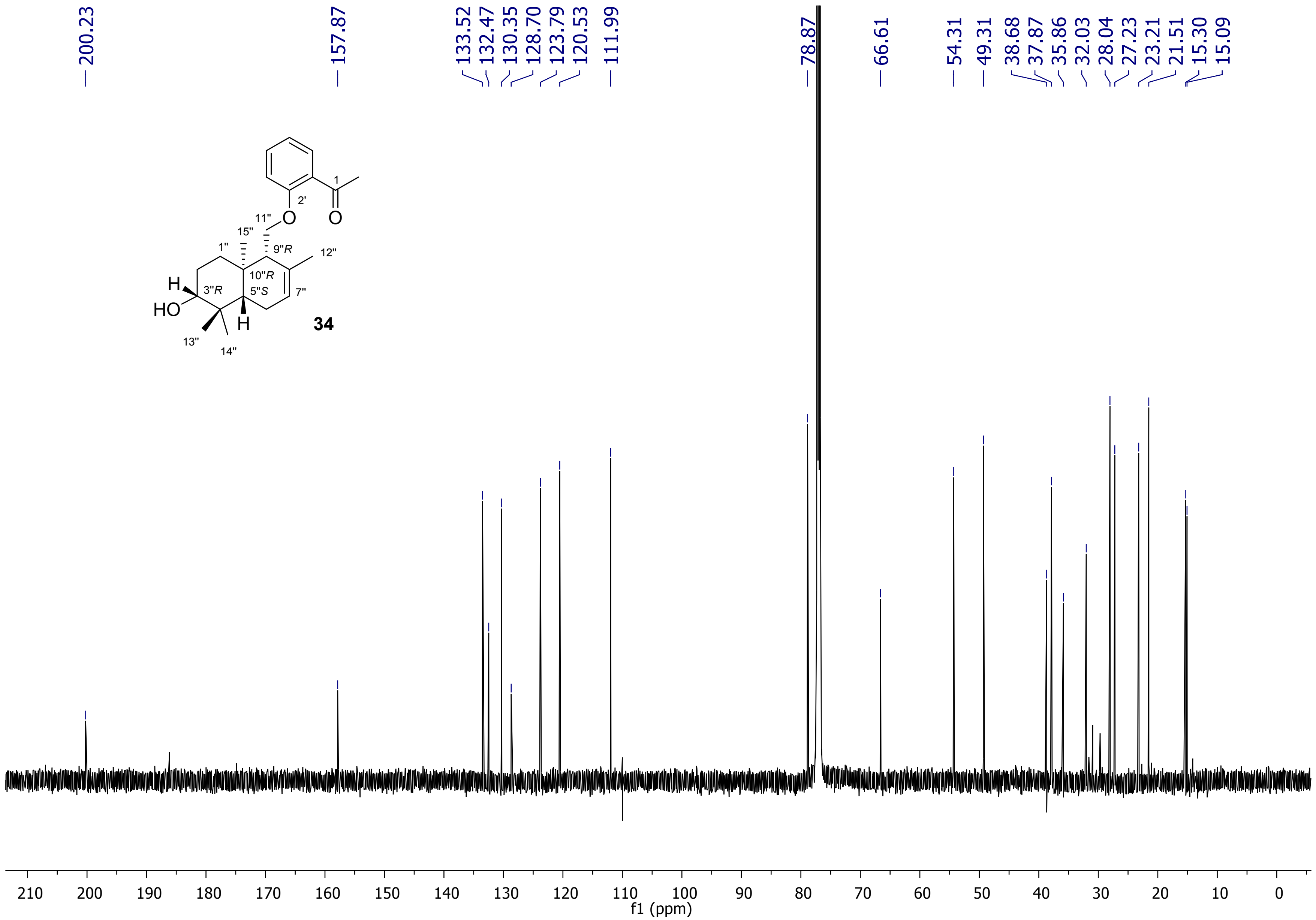

Figure S17b. ^{13}C NMR spectrum (125 MHz) of 1-(2'-(3"*R*(*S*),5"*S*(*R*),9"*R*(*S*),10"*R*(*S*)-3"-hydroxydrim-7"-en-11"-yloxy)phenyl)ethanone ((±)-**34**) in $CDCl_3$.

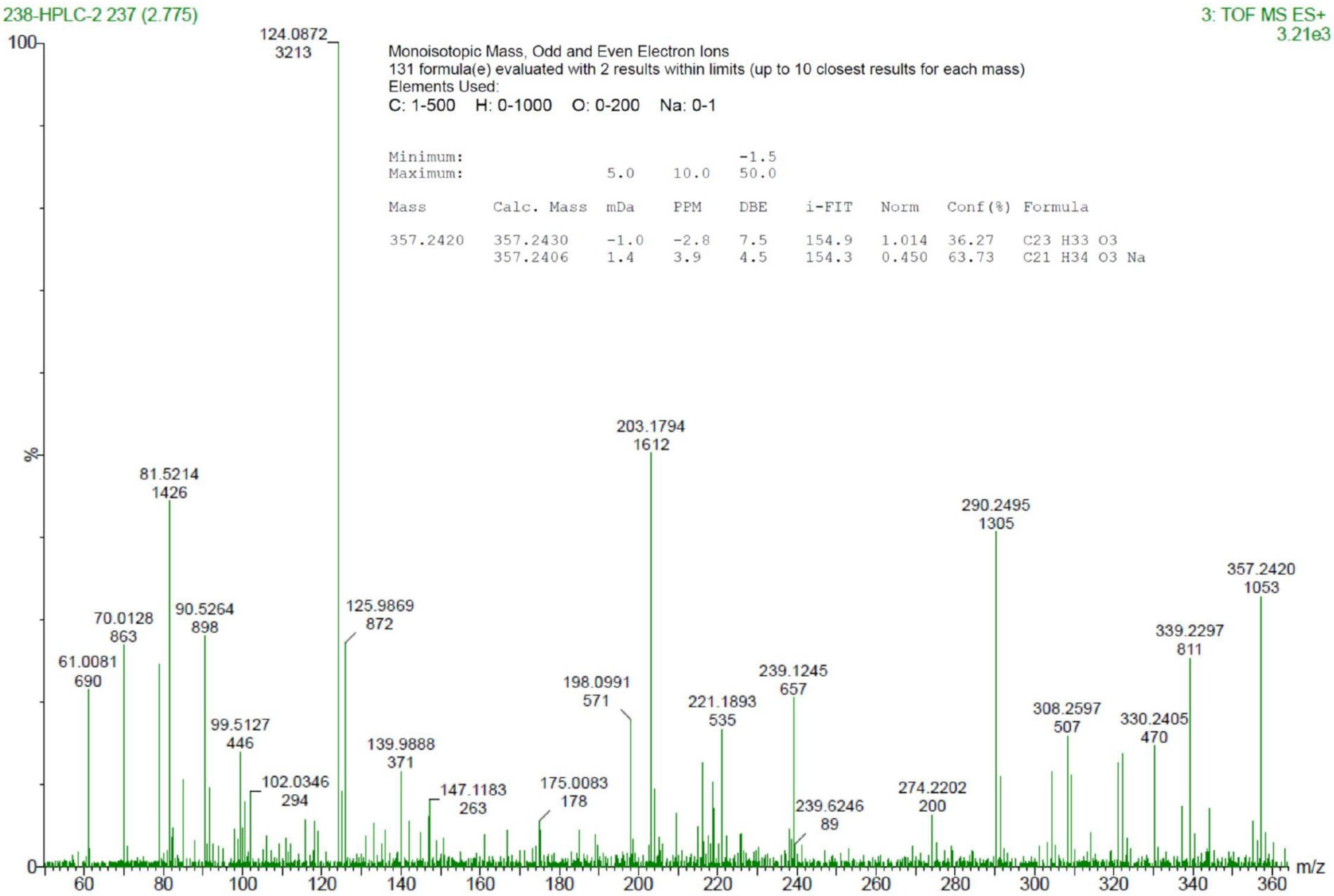

Figure S17c. HRESIMS of 1-(2'-(3"*R*(*S*),5"*S*(*R*),9"*R*(*S*),10"*R*(*S*)-3"-hydroxydrim-7"-en-11"-yloxy)phenyl)ethanone ((±)-**34**).

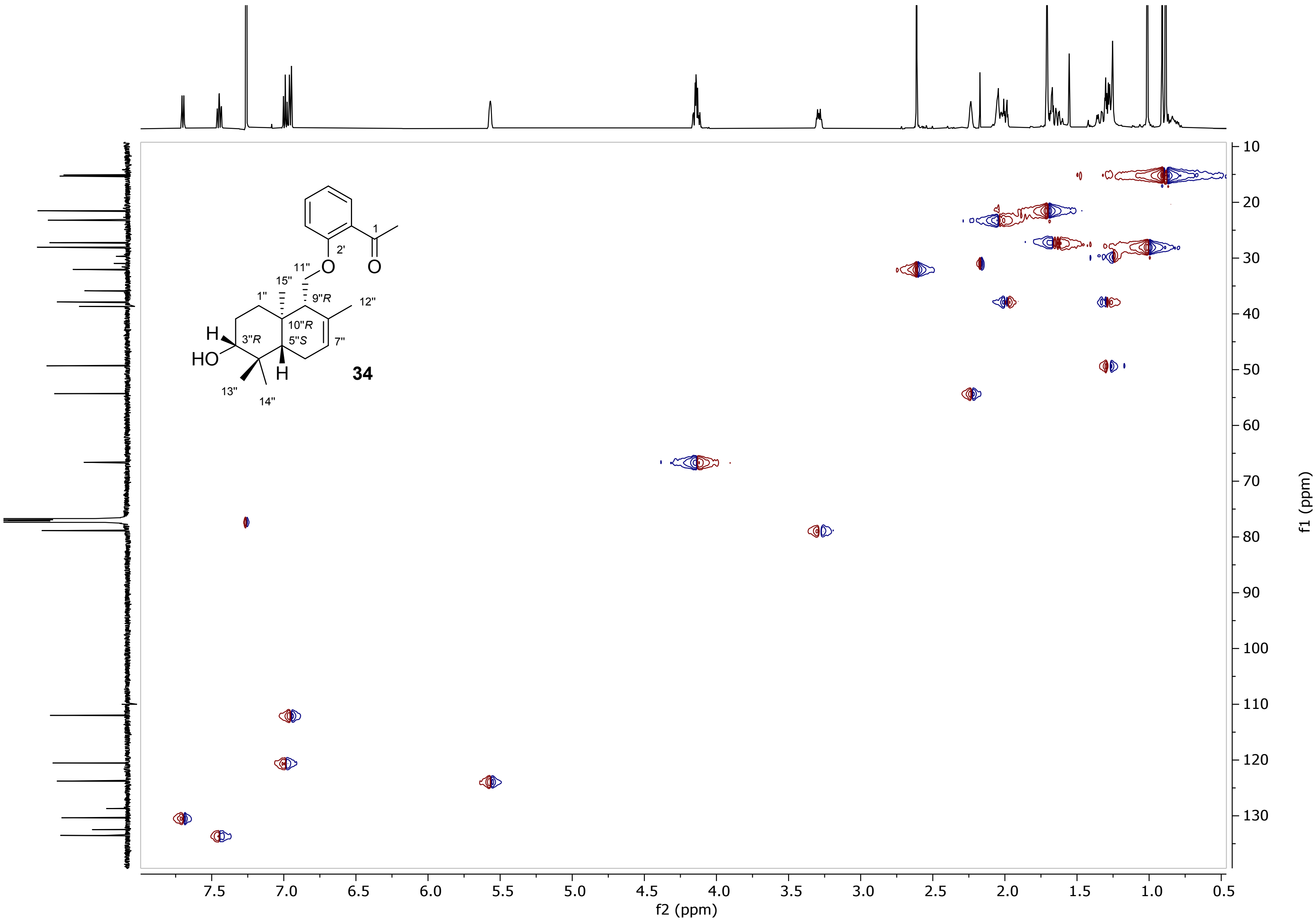

Figure S17d. gHSQC spectrum of 1-(2'-(3"*R*(*S*),5"*S*(*R*),9"*R*(*S*),10"*R*(*S*)-3"-hydroxydrim-7"-en-11"-yloxy)phenyl)ethanone ((±)-**34**) in $CDCl_3$.

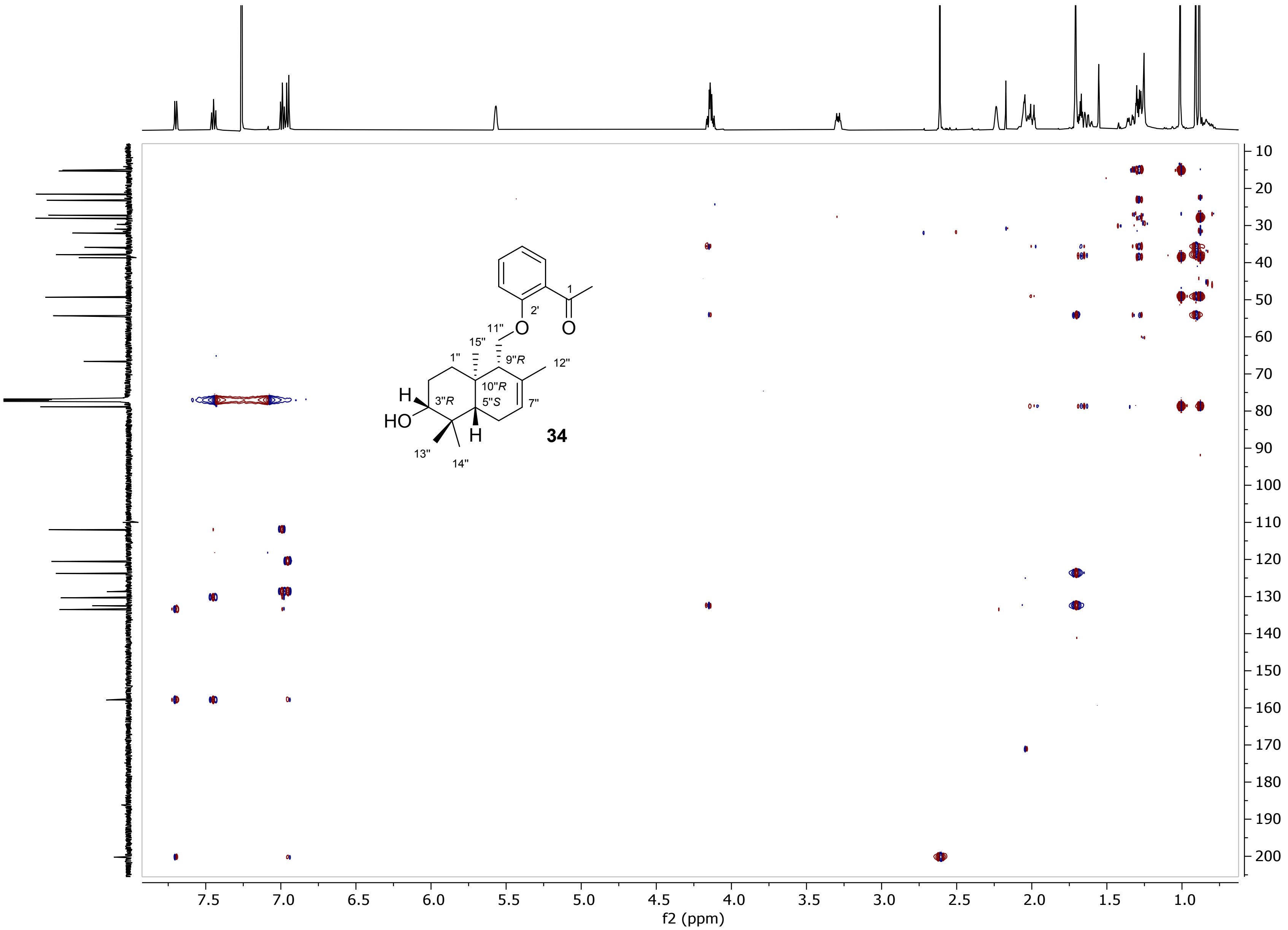

Figure S17e. gHMBC spectrum of 1-(2'-(3"*R*(*S*),5"*S*(*R*),9"*R*(*S*),10"*R*(*S*)-3"-hydroxydrim-7"-en-11"-yloxy)phenyl)ethanone ((±)-**34**) in $CDCl_3$.

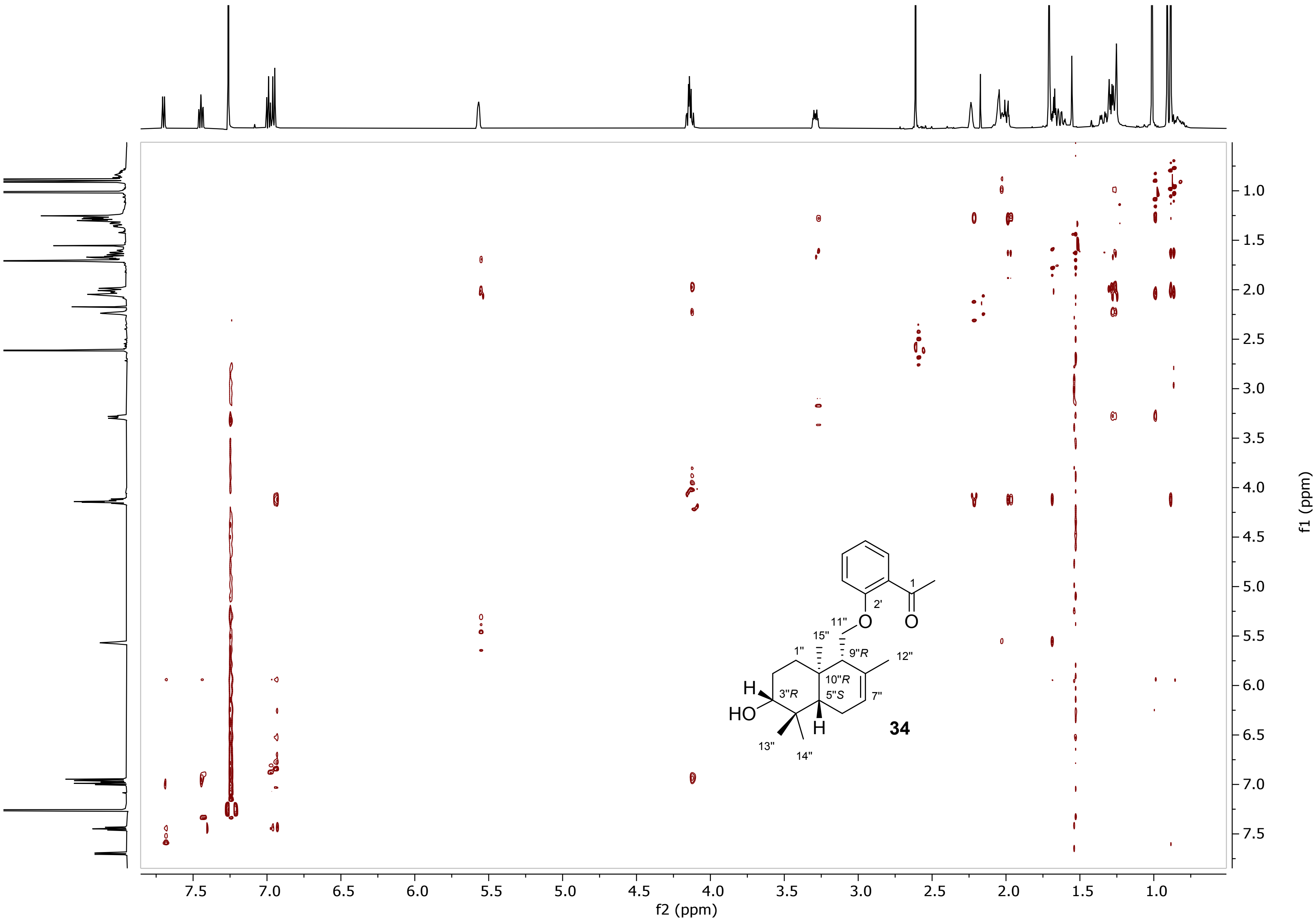

Figure S17f. NOESY2D spectrum of 1-(2'-(3"*R*(*S*),5"*S*(*R*),9"*R*(*S*),10"*R*(*S*)-3"-hydroxydrim-7"-en-11"-yloxy)phenyl)ethanone ((±)-**34**) in $CDCl_3$.

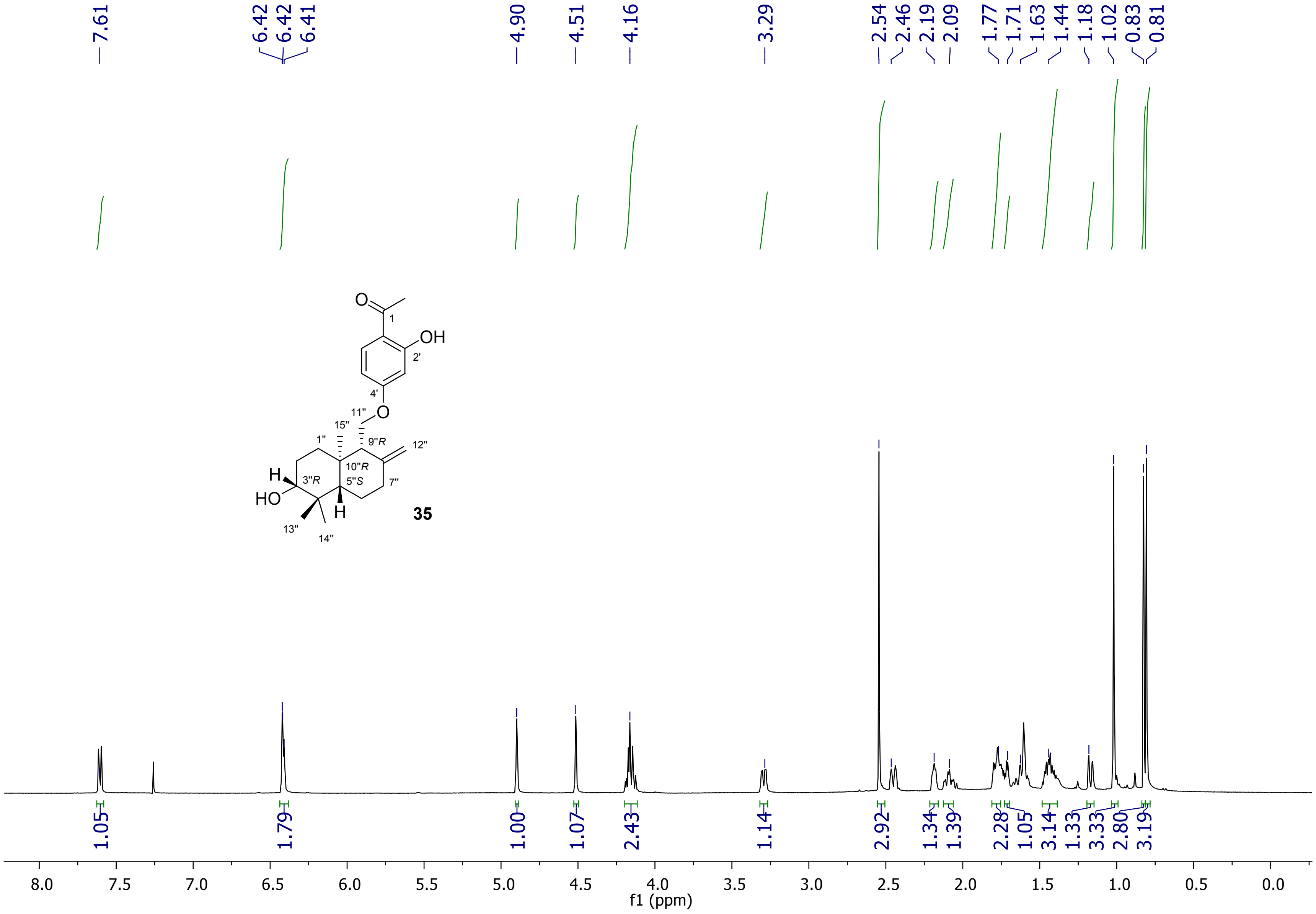

35

Figure S18a. ^{1}H NMR spectrum (500 MHz) of 1-(2'-hydroxy-4'-(3"*R*(*S*),5"*S*(*R*),9"*R*(*S*),10"*R*(*S*)-3"-hydroxydrim-8"(12")-en-11"-yloxy)phenyl)ethanone ((±)-**35**) in $CDCl_3$.

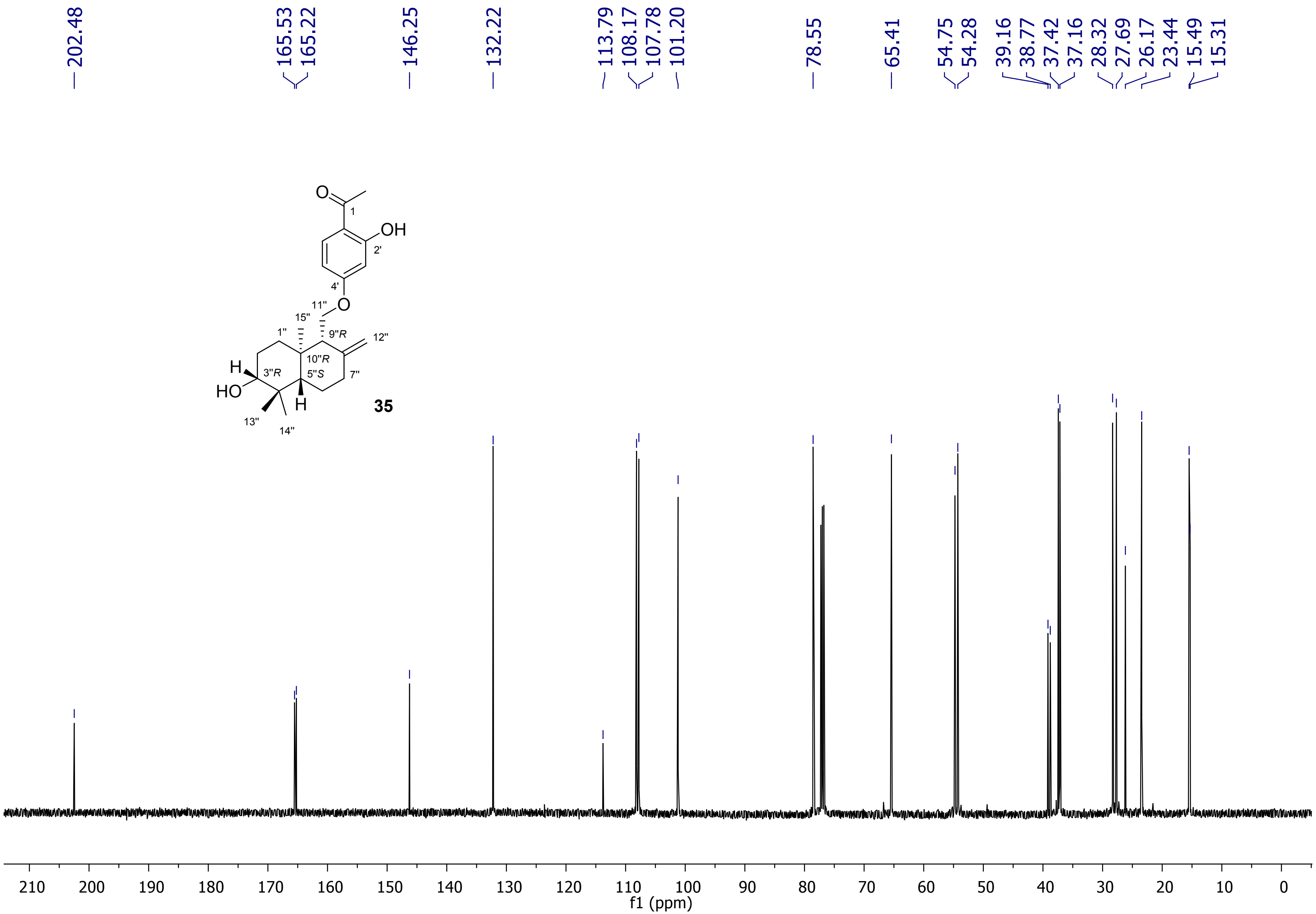

Figure S18b. ^{13}C NMR spectrum (125 MHz) of 1-(2’-hydroxy-4’-(3"*R*(*S*),5"*S*(*R*),9"*R*(*S*),10"*R*(*S*)-3"-hydroxydrim-8"(12")-en-11"-yloxy)phenyl)ethanone ((±)-**35**) in $CDCl_3$.

Monoisotopic Mass, Odd and Even Electron Ions
77 formula(e) evaluated with 1 results within limits (up to 50 best isotopic matches for each mass)
Elements Used:
C: 0-500 H: 0-1000 O: 0-200

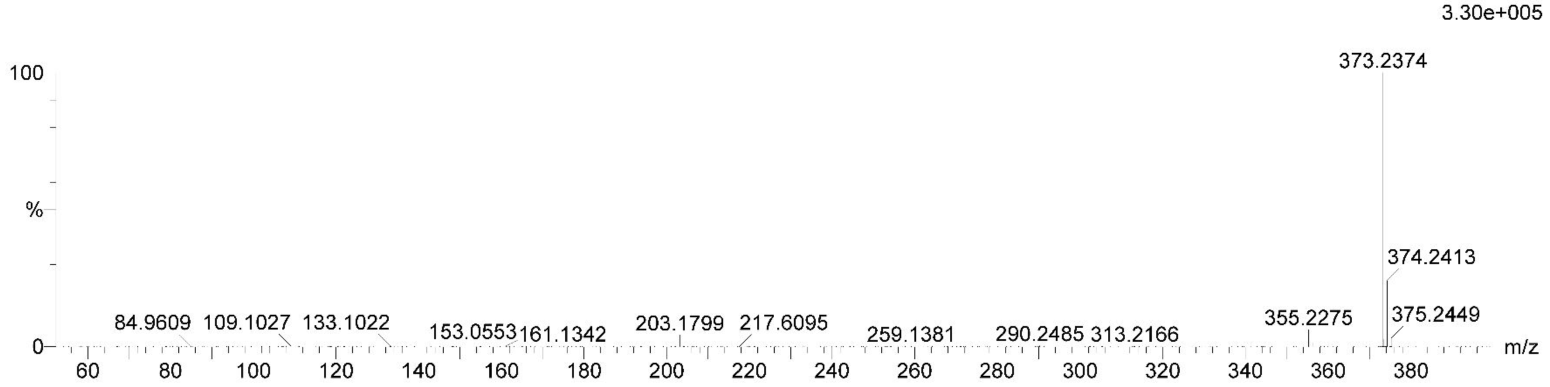

Minimum:	80.00				-1.5				
Maximum:	100.00		3.0	10.0	50.0				
Mass	RA	Calc. Mass	mDa	PPM	DBE	i-FIT	Norm	Conf(%)	Formula
373.2374	100.00	373.2379	-0.5	-1.3	7.5	205.2	n/a	n/a	C23 H33 O4

Figure S18c. HRESIMS of 1-(2'-hydroxy-4'-(3"*R*(*S*),5"*S*(*R*),9"*R*(*S*),10"*R*(*S*)-3"-hydroxydrim-8"(12")-en-11"-yloxy)phenyl)ethanone ((±)-**35**).

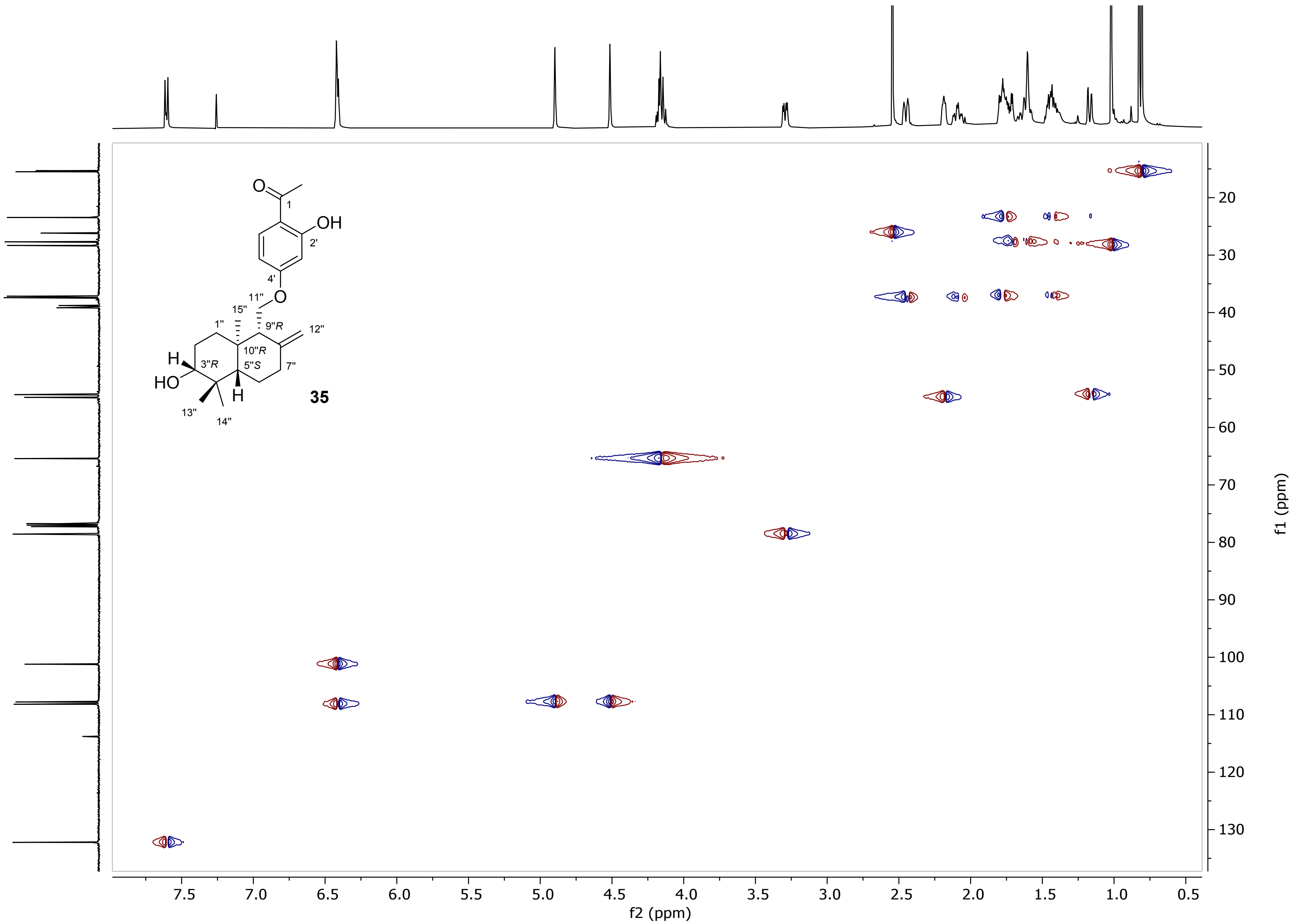

Figure S18d. gHSQC spectrum of 1-(2'-hydroxy-4'-(3"*R*(*S*),5"*S*(*R*),9"*R*(*S*),10"*R*(*S*)-3"-hydroxydrim-8"(12")-en-11"-yloxy)phenyl)ethanone ((±)-**35**) in $CDCl_3$.

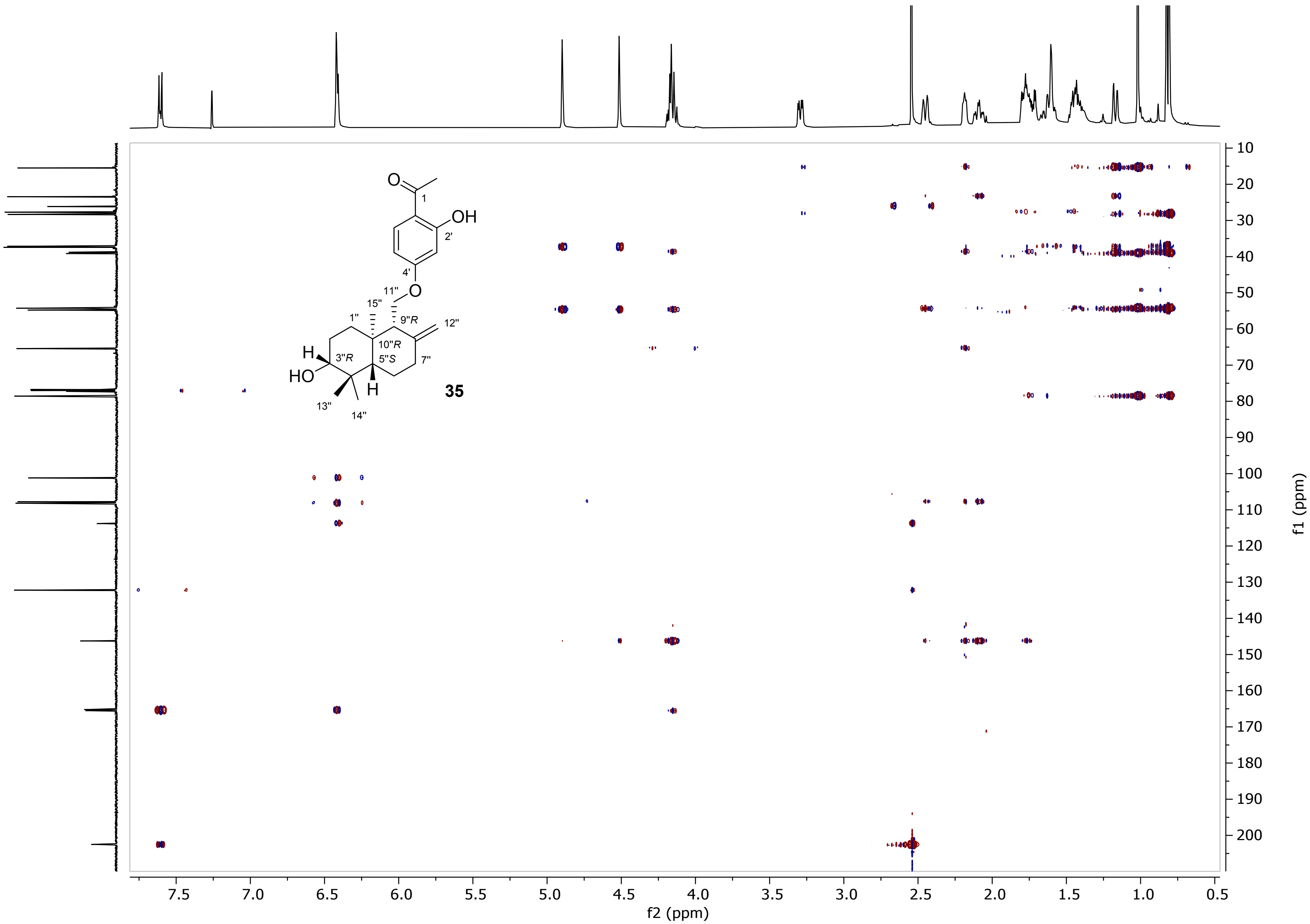

Figure S18e. gHMBC spectrum of 1-(2'-hydroxy-4'-(3"*R*(*S*),5"*S*(*R*),9"*R*(*S*),10"*R*(*S*)-3"-hydroxydrim-8"(12")-en-11"-yloxy)phenyl)ethanone ((±)-**35**) in $CDCl_3$.

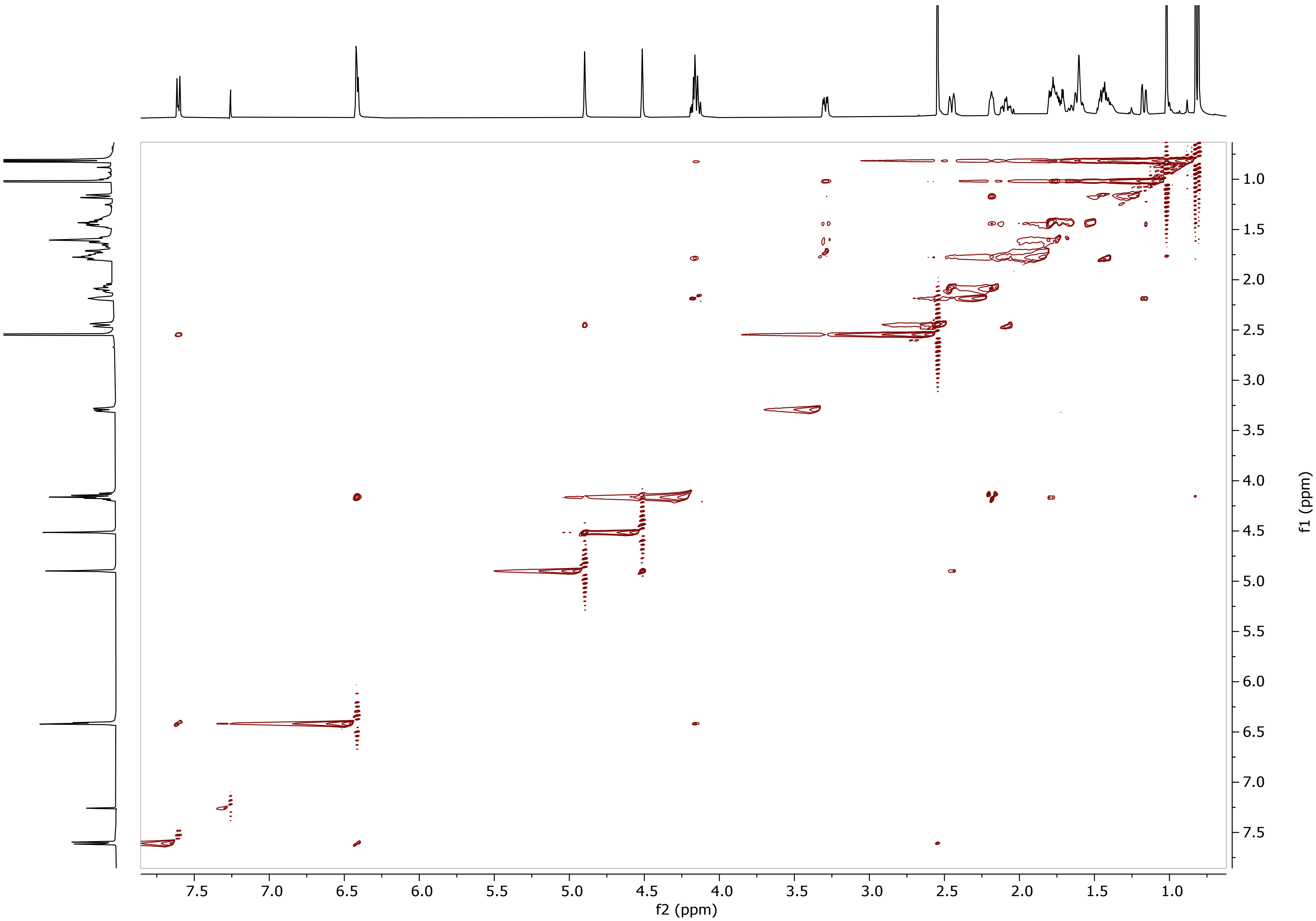

Figure S18f. NOESY2D spectrum of 1-(2'-hydroxy-4'-(3"*R*(*S*),5"*S*(*R*),9"*R*(*S*),10"*R*(*S*)-3"-hydroxydrim-8"(12")-en-11"-yloxy)phenyl)ethanone ((±)-**35**) in $CDCl_3$.

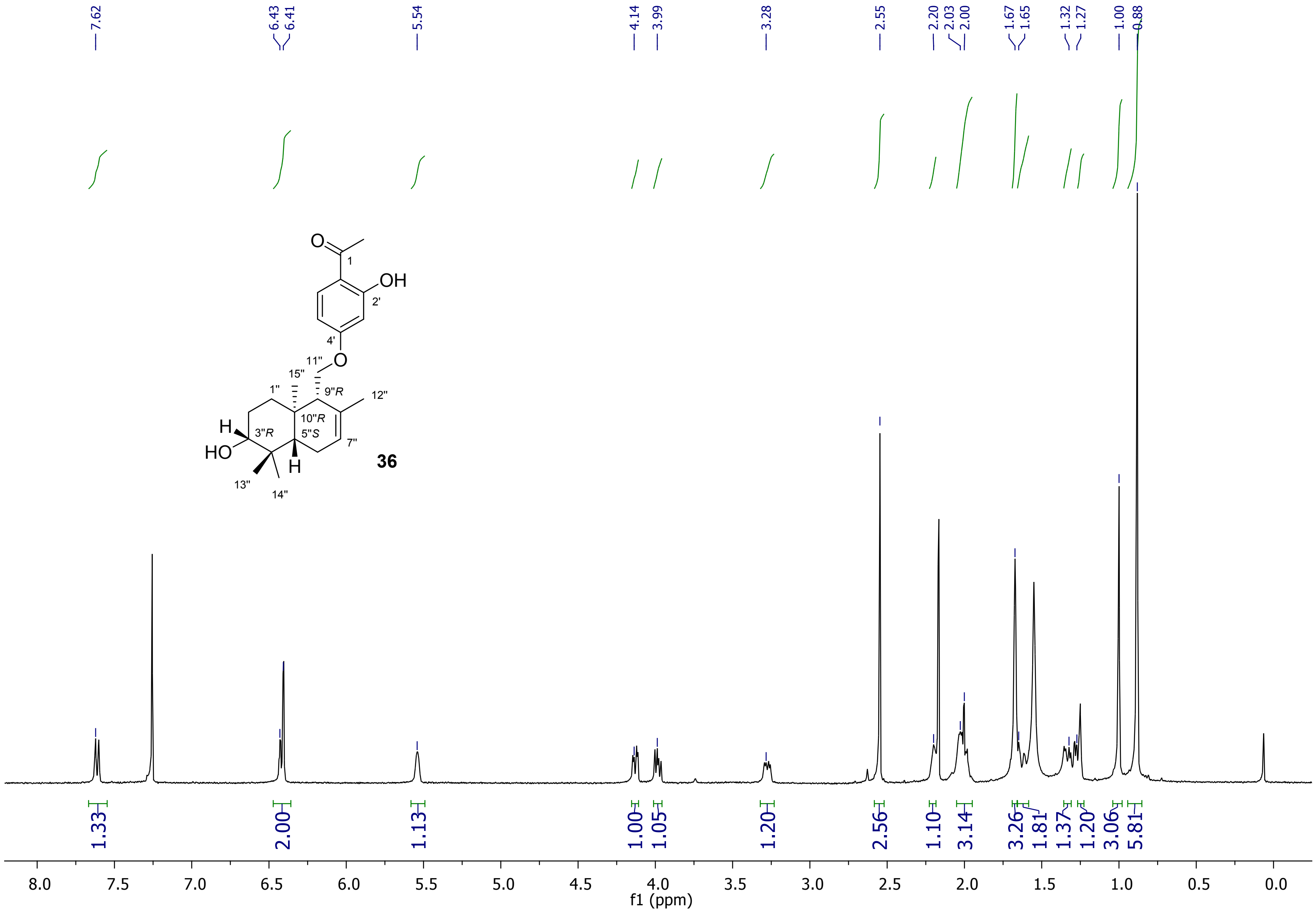

Figure S19a. ^{1}H NMR spectrum (400 MHz) of 1-(2'-hydroxy-4'-(3"*R*(*S*),5"*S*(*R*),9"*R*(*S*),10"*R*(*S*)-3"-hydroxydrim-7"-en-11"-yloxy)phenyl)ethanone ((±)-**36**) in $CDCl_3$.

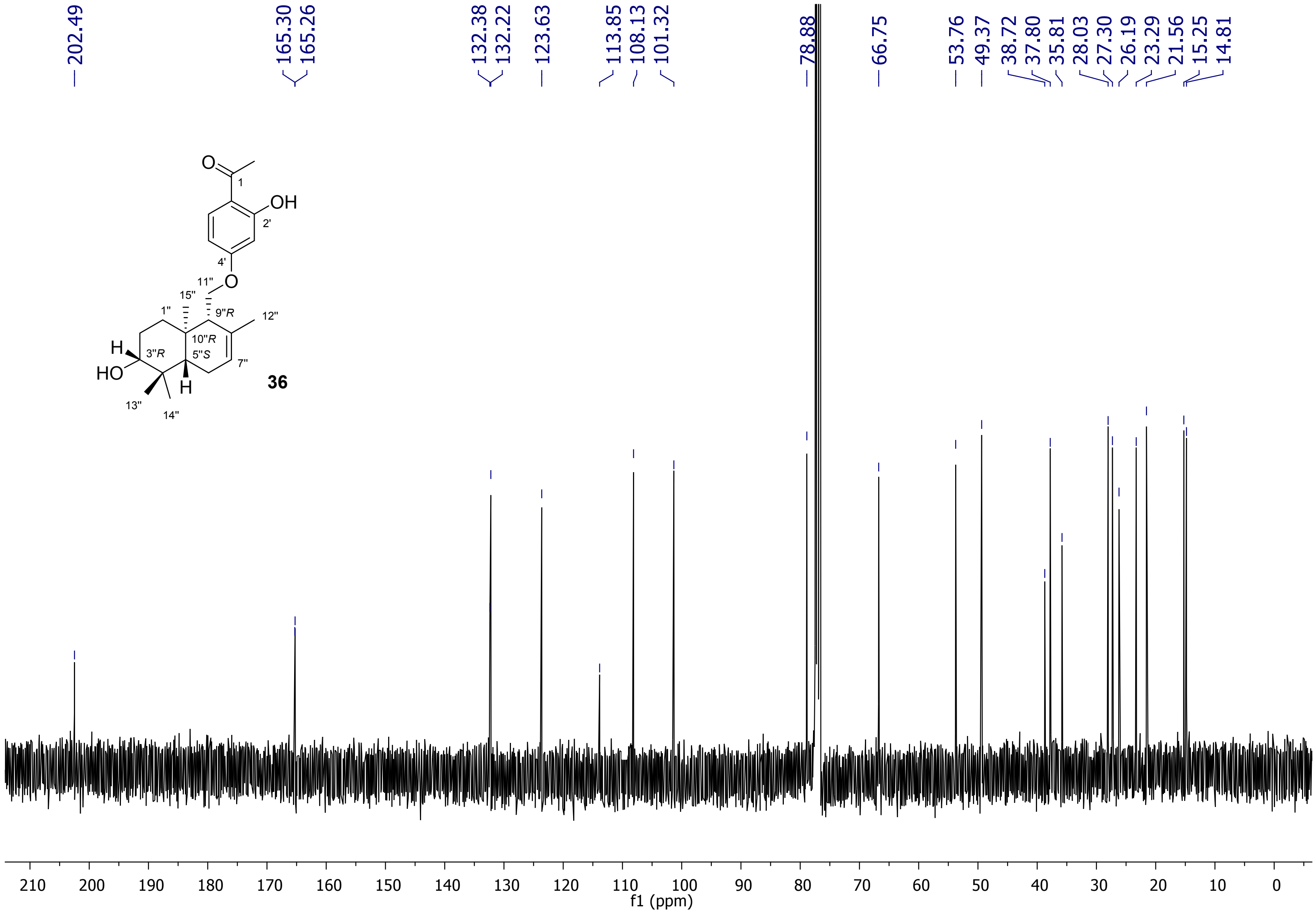

Figure S19b. ^{13}C NMR spectrum (100 MHz) of 1-(2'-hydroxy-4'-(3"*R*(*S*),5"*S*(*R*),9"*R*(*S*),10"*R*(*S*)-3"-hydroxydrim-7"-en-11"-yloxy)phenyl)ethanone ((±)-**36**) in $CDCl_3$.

Monoisotopic Mass, Even Electron Ions
137 formula(e) evaluated with 2 results within limits (up to 10 best isotopic matches for each mass)
Elements Used:
C: 1-500 H: 0-1000 O: 0-200 K: 0-1
246-HPLC-9 285 (3.316)
1: TOF MS ES+

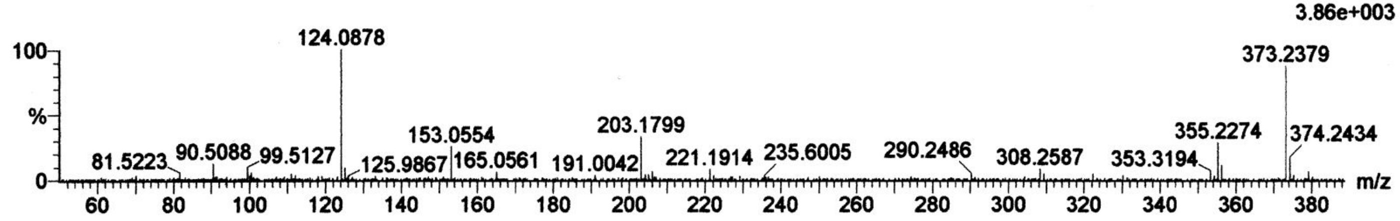

Mass	Calc. Mass	mDa	PPM	DBE	i-FIT	Norm	Conf(%)	Formula
Minimum:				-1.5				
Maximum:		5.0	10.0	50.0				
373.2379	373.2379	0.0	0.0	7.5	35.2	0.000	99.98	C23 H33 O4
	373.2356	2.3	6.2	-0.5	43.7	8.461	0.02	C18 H38 O5 K

Figure S19c. HRESIMS of 1-(2’-hydroxy-4’-(3"*R*(*S*),5"*S*(*R*),9"*R*(*S*),10"*R*(*S*)-3"-hydroxydrim-7"-en-11"-yloxy)phenyl)ethanone ((±)-**36**).

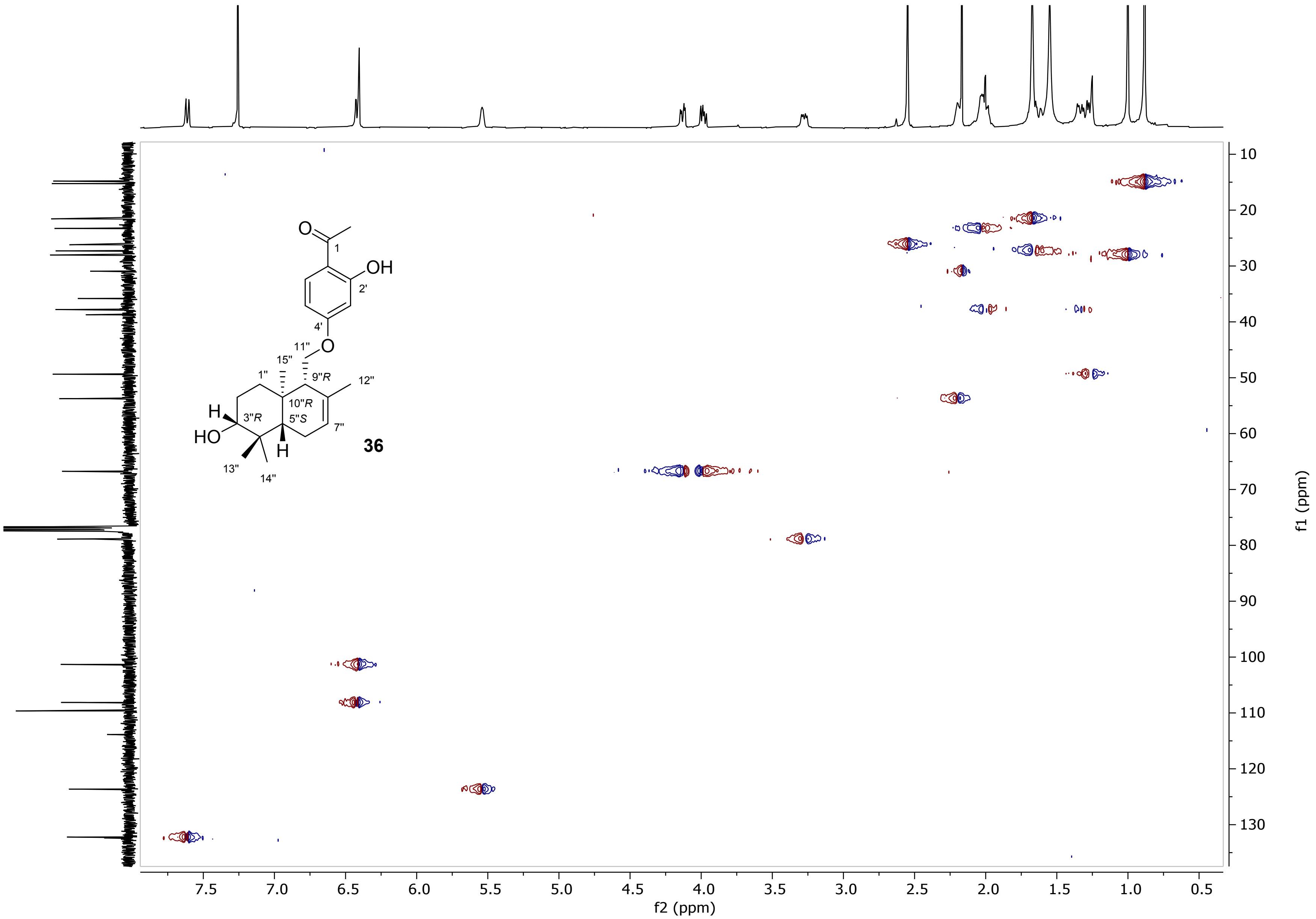

Figure S19d. gHSQC spectrum of 1-(2'-hydroxy-4'-(3"*R*(*S*),5"*S*(*R*),9"*R*(*S*),10"*R*(*S*)-3"-hydroxydrim-7"-en-11"-yloxy)phenyl)ethanone ((±)-**36**) in $CDCl_3$.

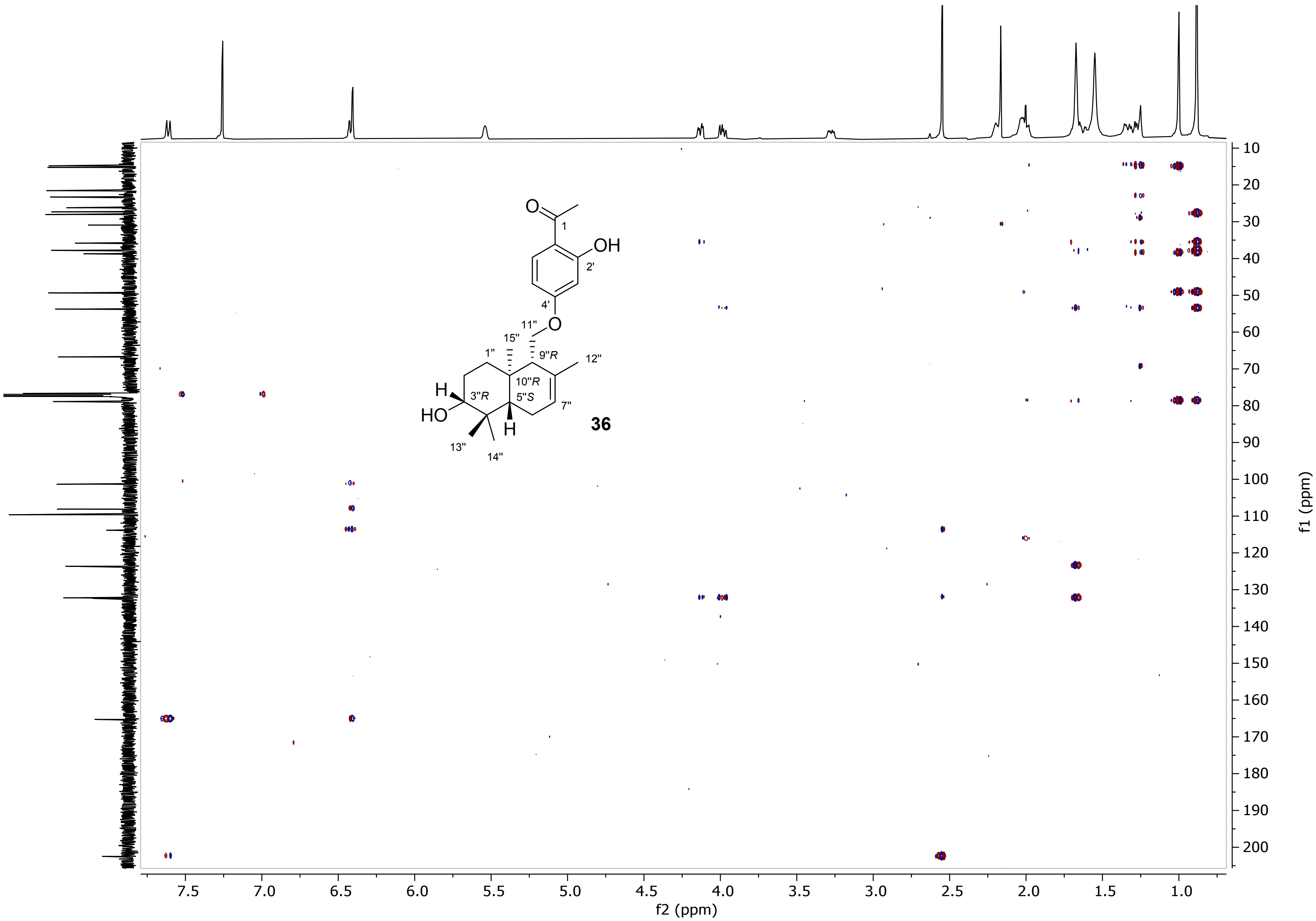

Figure S19e. gHMBC spectrum of 1-(2'-hydroxy-4'-(3"*R*(*S*),5"*S*(*R*),9"*R*(*S*),10"*R*(*S*)-3"-hydroxydrim-7"-en-11"-yloxy)phenyl)ethanone ((±)-**36**) in $CDCl_3$.

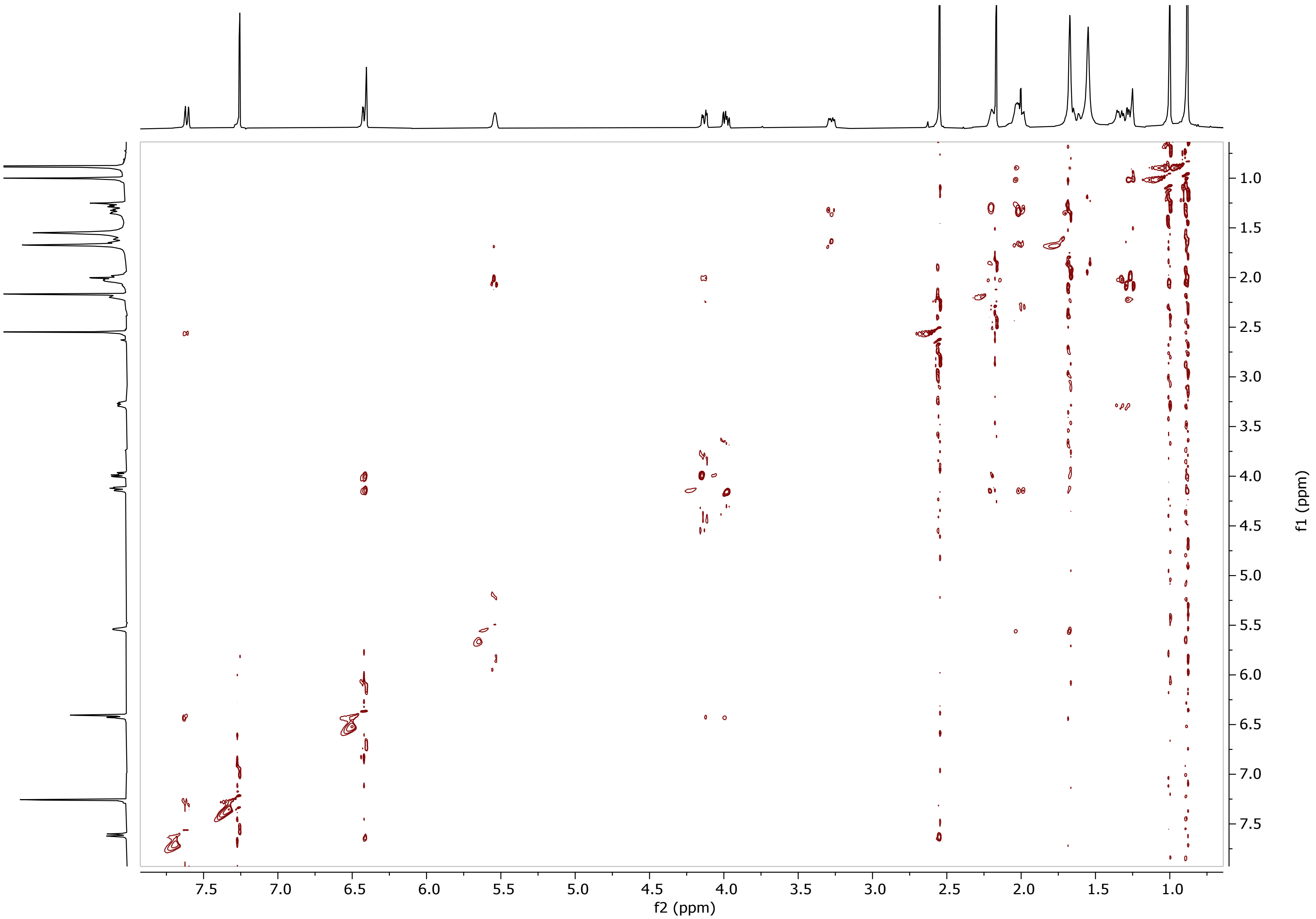

Figure S19f. NOESY2D spectrum of 1-(2’-hydroxy-4’-(3"*R*(*S*),5"*S*(*R*),9"*R*(*S*),10"*R*(*S*)-3"-hydroxydrim-7"-en-11"-yloxy)phenyl)ethanone ((±)-**36**) in $CDCl_3$.

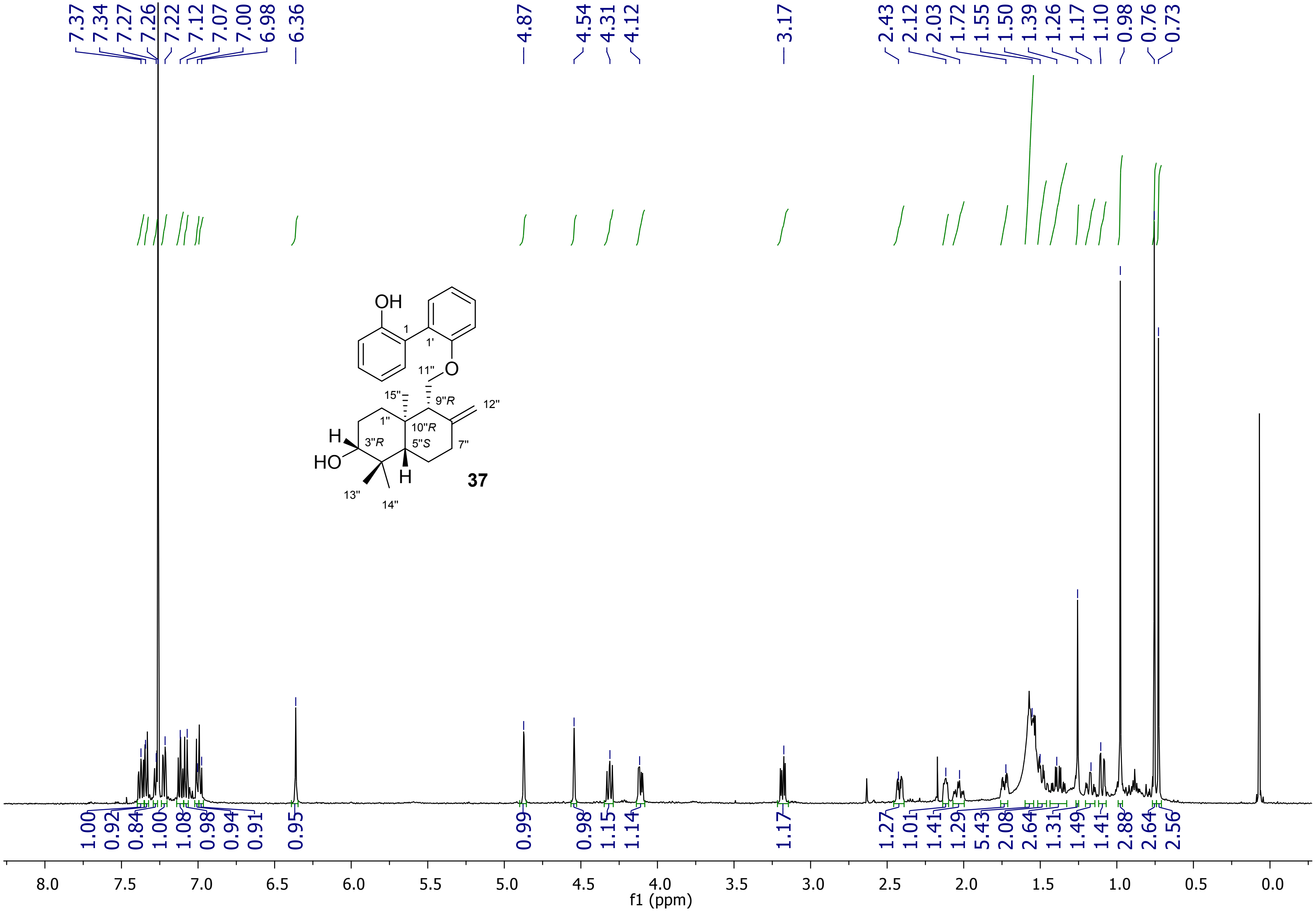

Figure S20a. ^{1}H NMR spectrum (500 MHz) of 2'-(3''*R*(*S*),5''*S*(*R*),9''*R*(*S*),10''*R*(*S*)-3''-hydroxydrim-8''(12'')-en-11''-yloxy)-[1,1'-biphenyl]-2-ol ((±)-**37**) in $CDCl_3$.

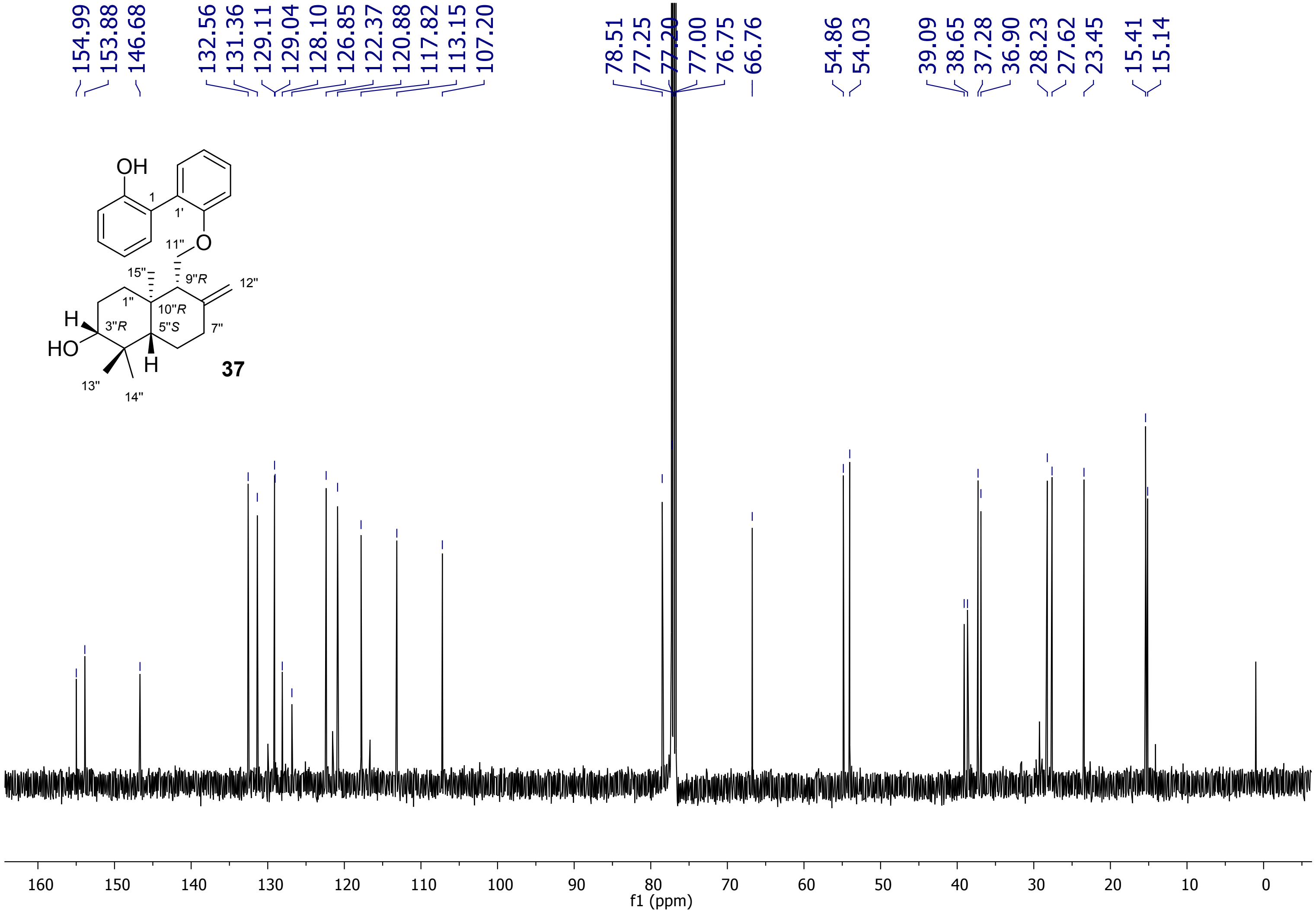

Figure S20b. ^{13}C NMR spectrum (125 MHz) of 2'-(3"*R*(*S*),5"*S*(*R*),9"*R*(*S*),10"*R*(*S*)-3"-hydroxydrim-8"(12")-en-11"-yloxy)-[1,1'-biphenyl]-2-ol ((±)-**37**) in $CDCl_3$.

Monoisotopic Mass, Odd and Even Electron Ions
180 formula(e) evaluated with 1 results within limits (up to 50 best isotopic matches for each mass)
Elements Used:
C: 0-500 H: 0-1000 O: 0-200 Na: 0-1

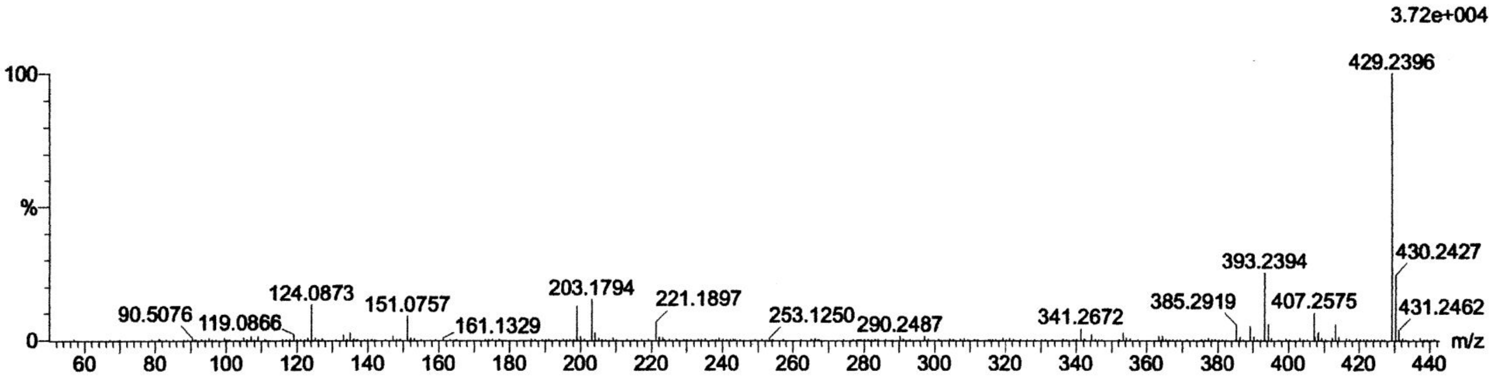

Minimum:	80.00				-1.5				
Maximum:	100.00		3.0	10.0	50.0				
Mass	RA	Calc. Mass	mDa	PPM	DBE	i-FIT	Norm	Conf(%)	Formula
429.2396	100.00	429.2406	-1.0	-2.3	10.5	94.0	n/a	n/a	C27 H34 O3 Na

Figure S20c. HRESIMS of 2'-(3"*R*(*S*),5"*S*(*R*),9"*R*(*S*),10"*R*(*S*)-3"-hydroxydrim-8"(12")-en-11"-yloxy)-[1,1'-biphenyl]-2-ol ((±)-**37**).

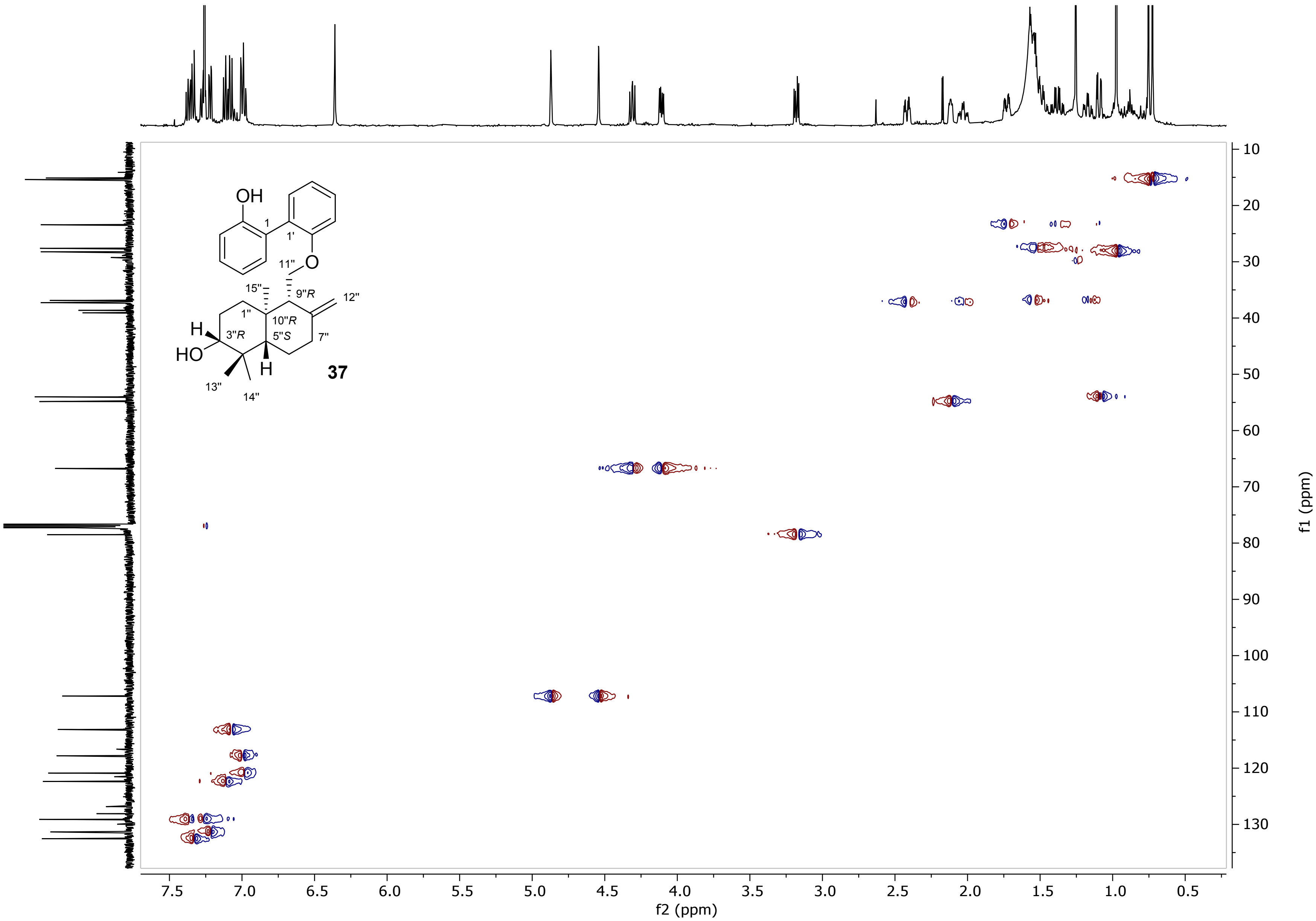

Figure S20d. gHSQC spectrum of 2'-(3''*R*(*S*),5''*S*(*R*),9''*R*(*S*),10''*R*(*S*)-3''-hydroxydrim-8''(12'')-en-11''-yloxy)-[1,1'-biphenyl]-2-ol ((±)-**37**) in $CDCl_3$.

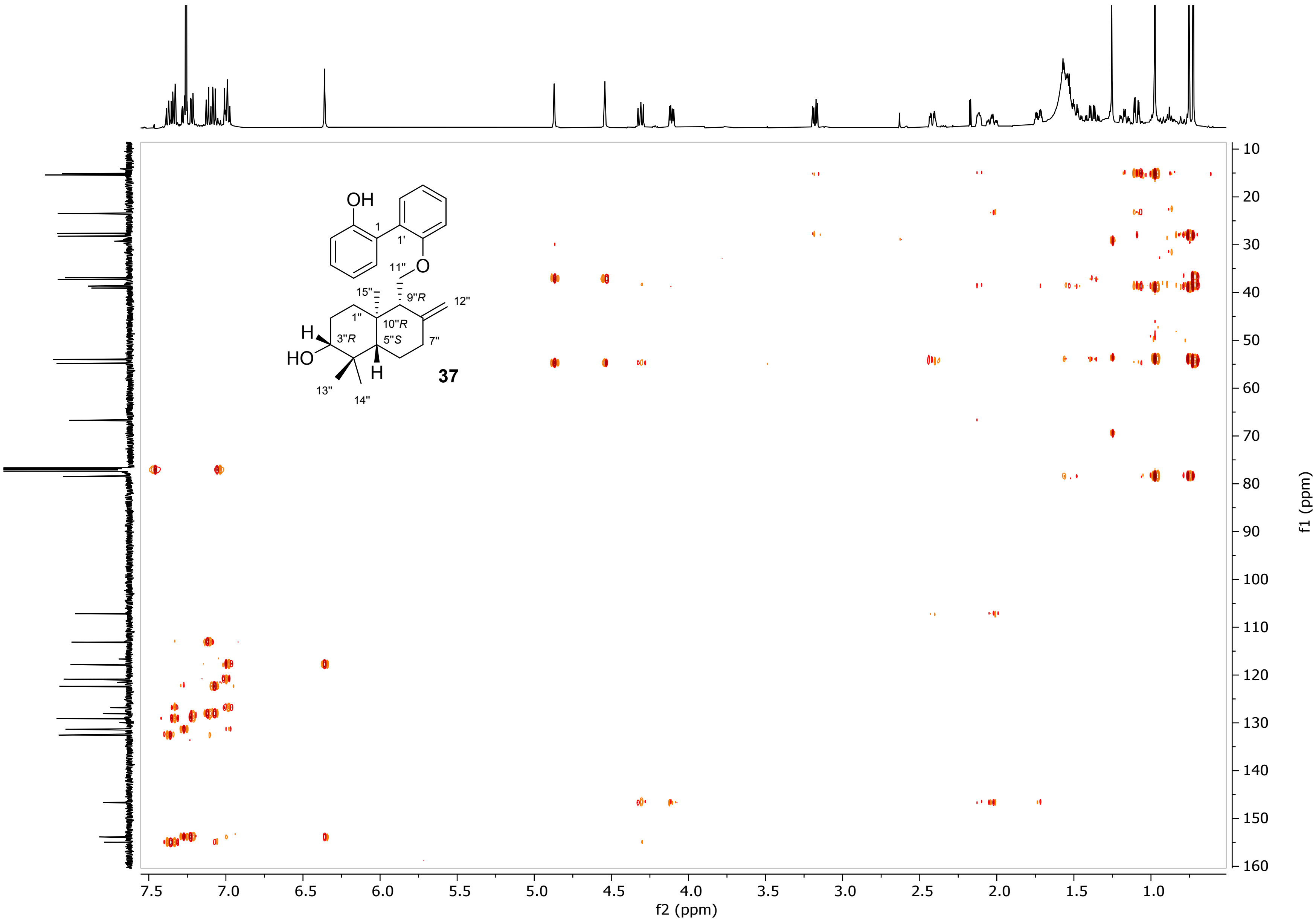

Figure S20e. gHMBC spectrum of 2'-(3"*R*(*S*),5"*S*(*R*),9"*R*(*S*),10"*R*(*S*)-3"-hydroxydrim-8"(12")-en-11"-yloxy)-[1,1'-biphenyl]-2-ol ((±)-**37**) in $CDCl_3$.

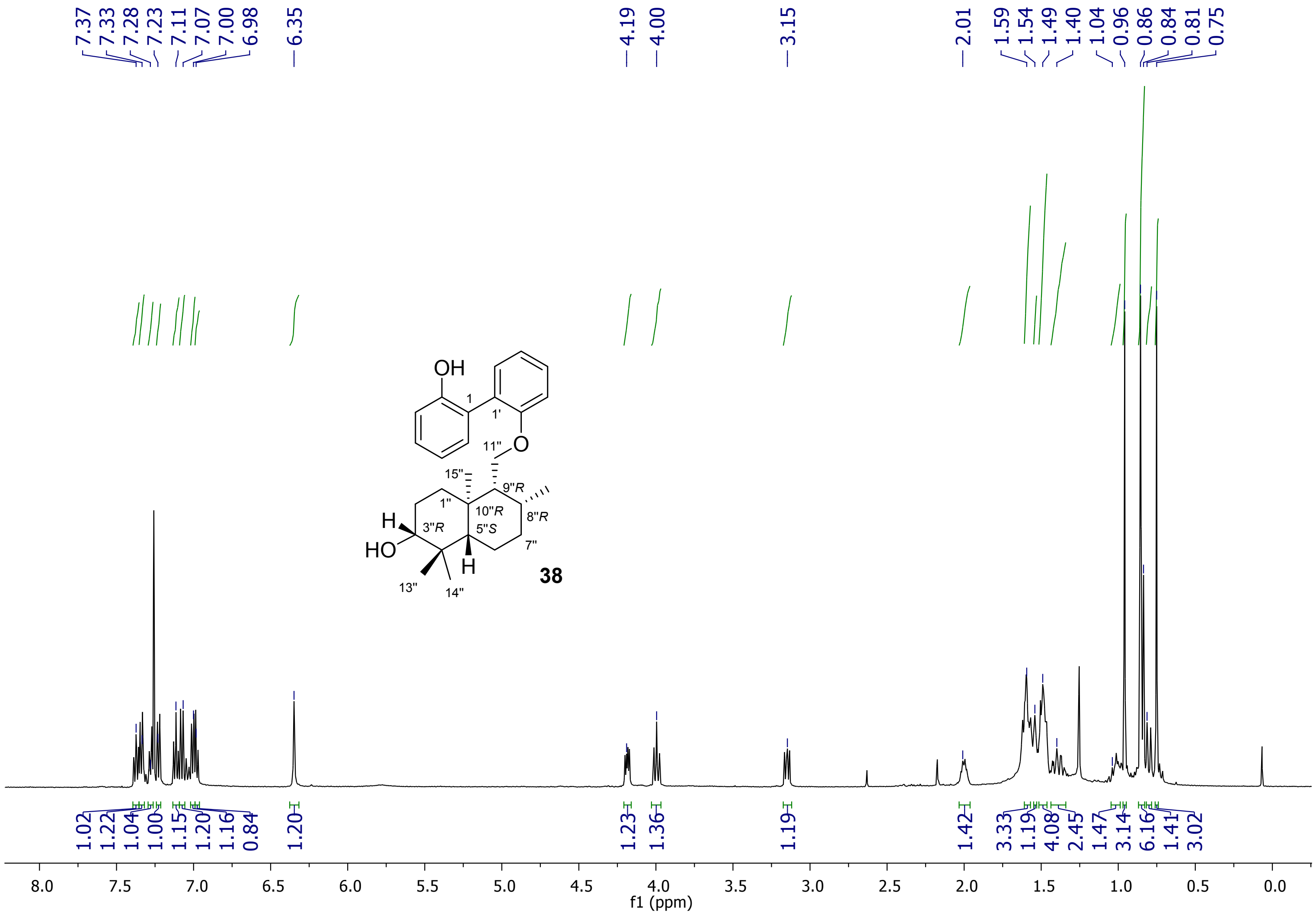

Figure S21a. ^{1}H NMR spectrum (500 MHz) of 2'-(3''*R*(*S*),5''*S*(*R*), 8''*R*(*S*),9''*R*(*S*),10''*R*(*S*)-3''-hydroxydriman-11''-yloxy)-[1,1'-biphenyl]-2-ol ((±)-**38**) in $CDCl_3$.

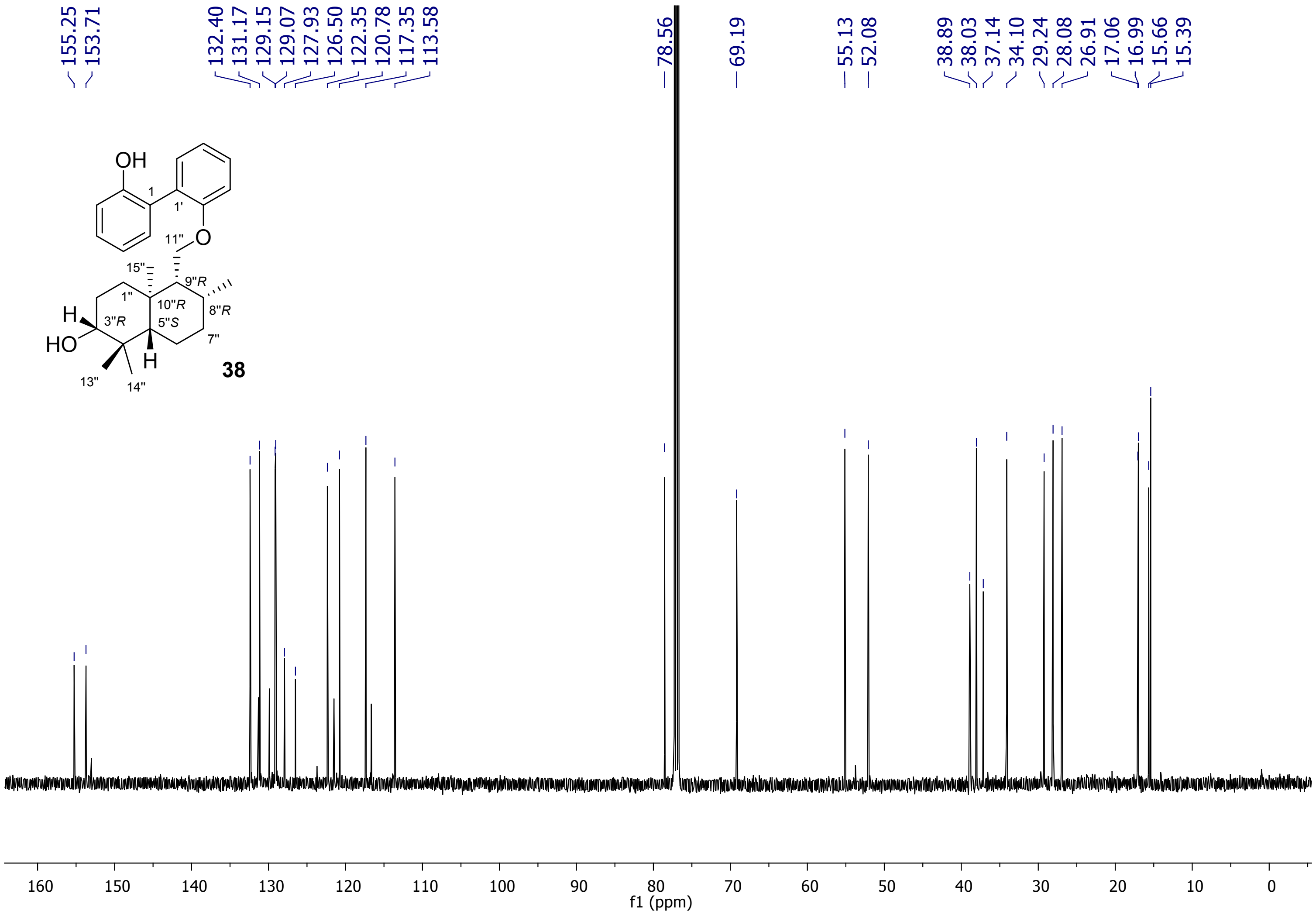

Figure S21b. ^{13}C NMR spectrum (125 MHz) of 2'-(3"*R*(*S*),5"*S*(*R*), 8"*R*(*S*),9"*R*(*S*),10"*R*(*S*)-3"-hydroxydriman-11"-yloxy)-[1,1'-biphenyl]-2-ol ((±)-**38**) in $CDCl_3$.

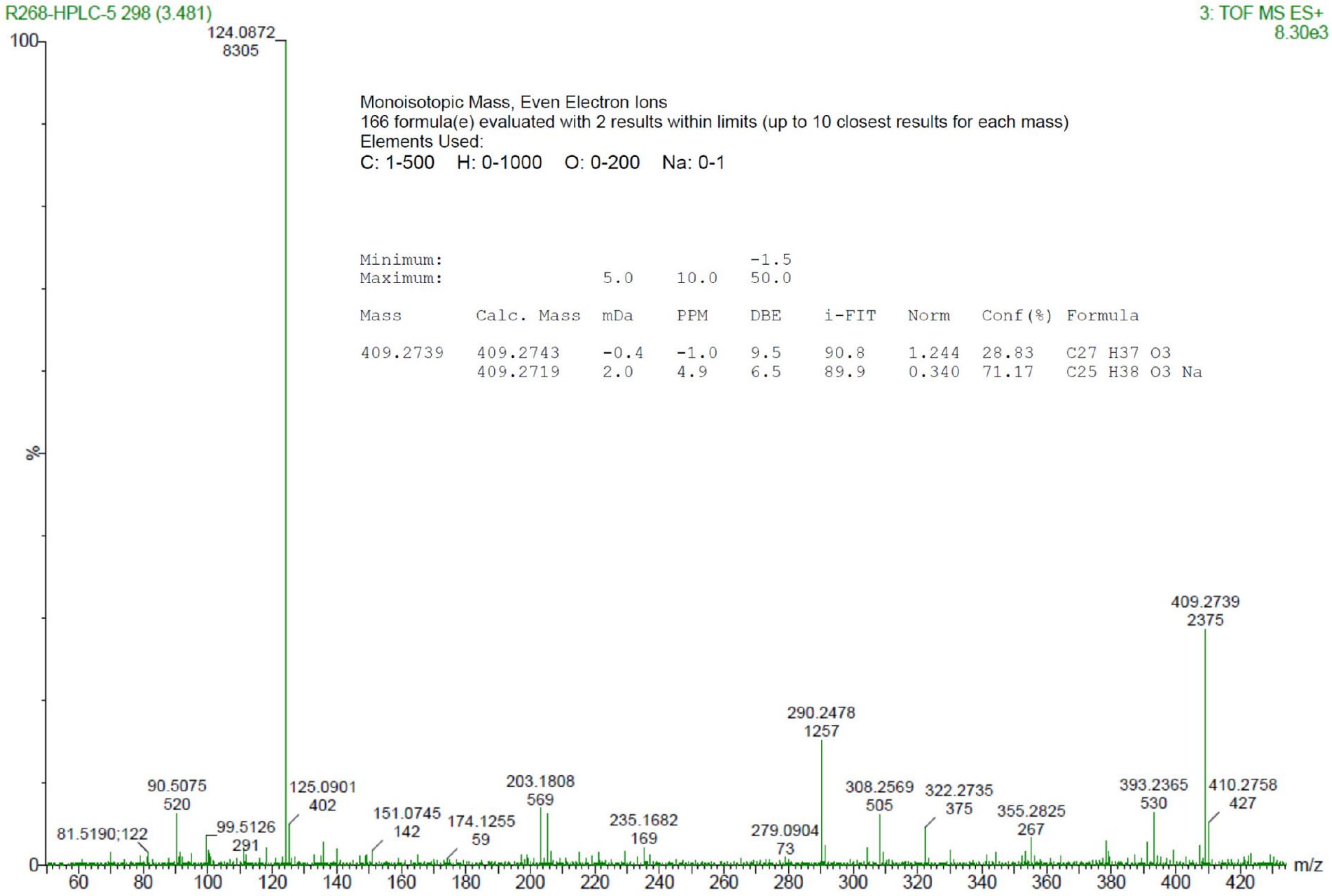

Figure S21c. HRESIMS of 2'-(3"*R*(*S*),5"*S*(*R*), 8"*R*(*S*),9"*R*(*S*),10"*R*(*S*)-3"-hydroxydriman-11"-yloxy)-[1,1'-biphenyl]-2-ol ((±)-**38**).

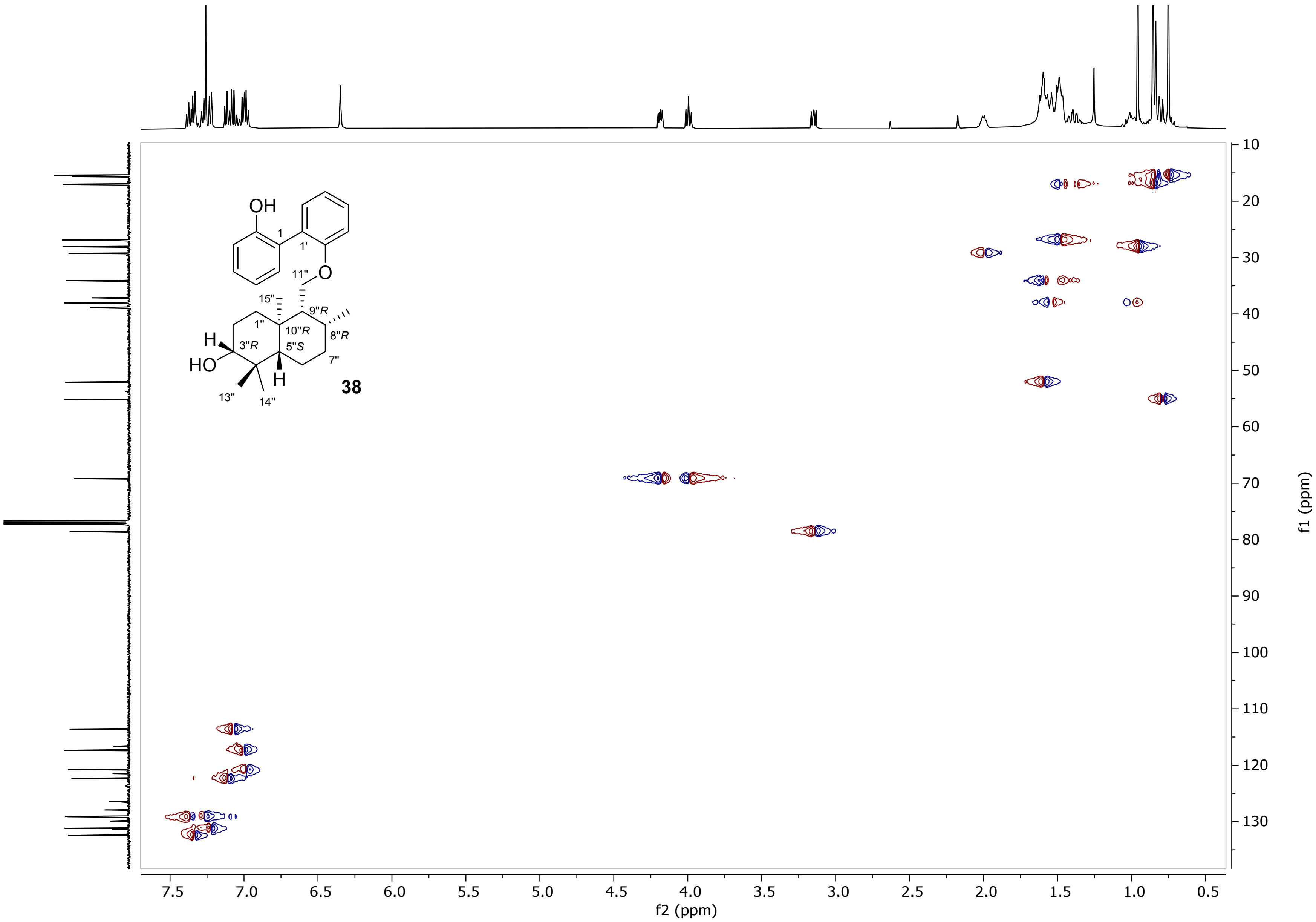

Figure S21d. gHSQC spectrum of 2'-(3''*R*(*S*),5''*S*(*R*), 8''*R*(*S*),9''*R*(*S*),10''*R*(*S*)-3''-hydroxydriman-11''-yloxy)-[1,1'-biphenyl]-2-ol ((±)-**38**) in $CDCl_3$.

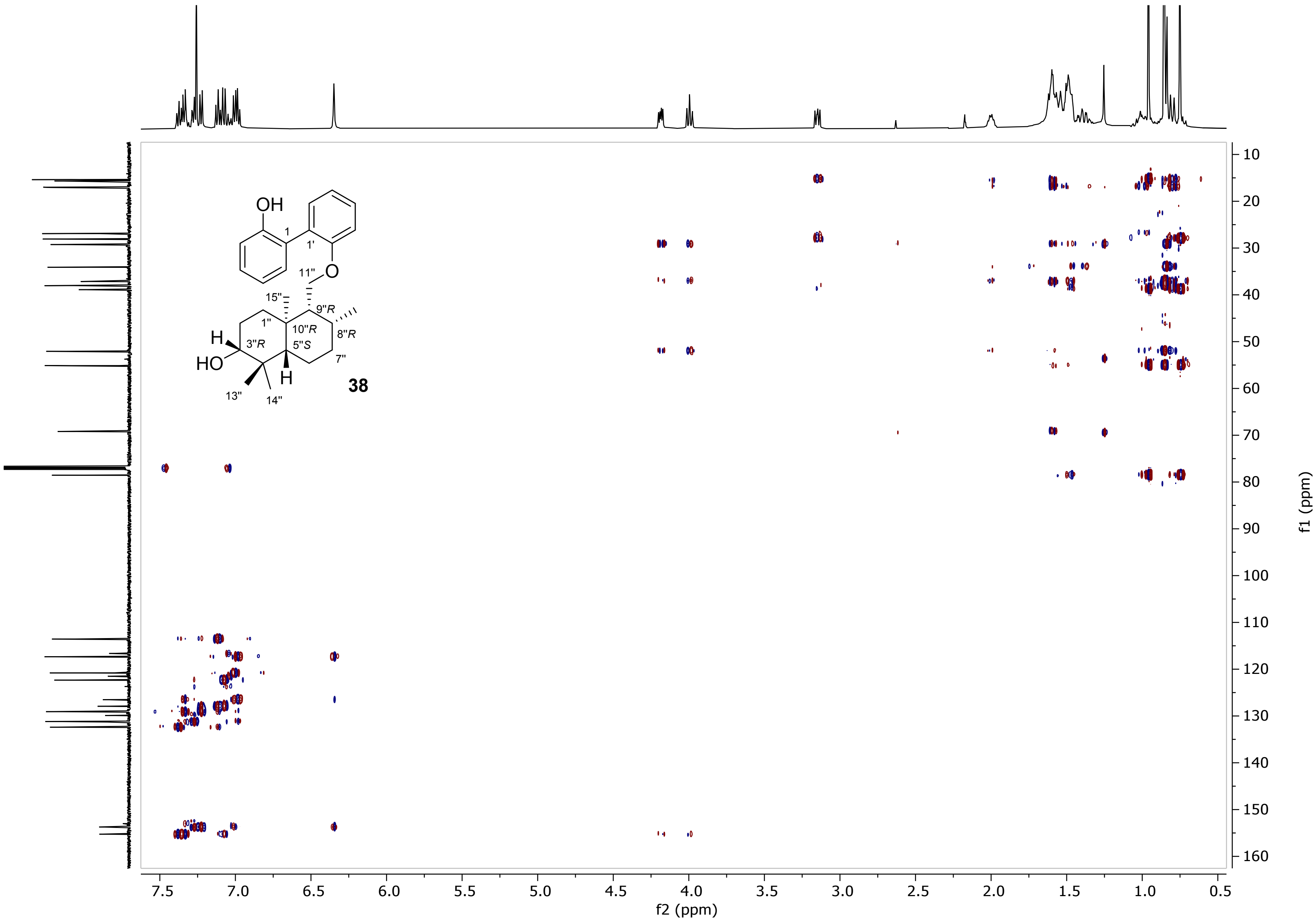

Figure S21e. gHMBC spectrum of 2'-(3"*R*(*S*),5"*S*(*R*), 8"*R*(*S*),9"*R*(*S*),10"*R*(*S*)-3"-hydroxydriman-11"-yloxy)-[1,1'-biphenyl]-2-ol ((±)-**38**) in $CDCl_3$.

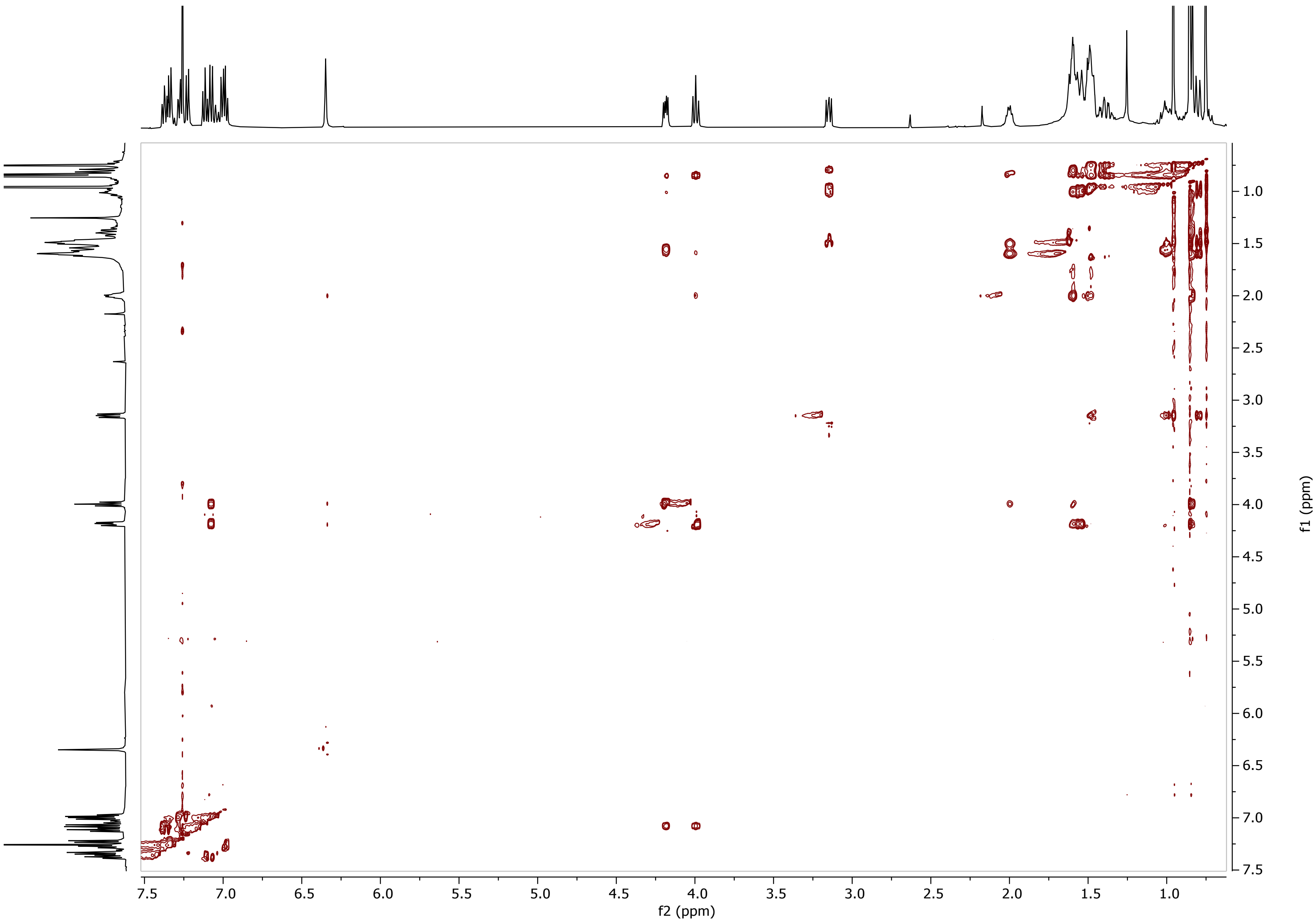

Figure S21f. NOESY2D spectrum of 2'-(3"*R*(*S*),5"*S*(*R*), 8"*R*(*S*),9"*R*(*S*),10"*R*(*S*)-3"-hydroxydriman-11"-yloxy)-[1,1'-biphenyl]-2-ol ((±)-**38**) in $CDCl_3$.

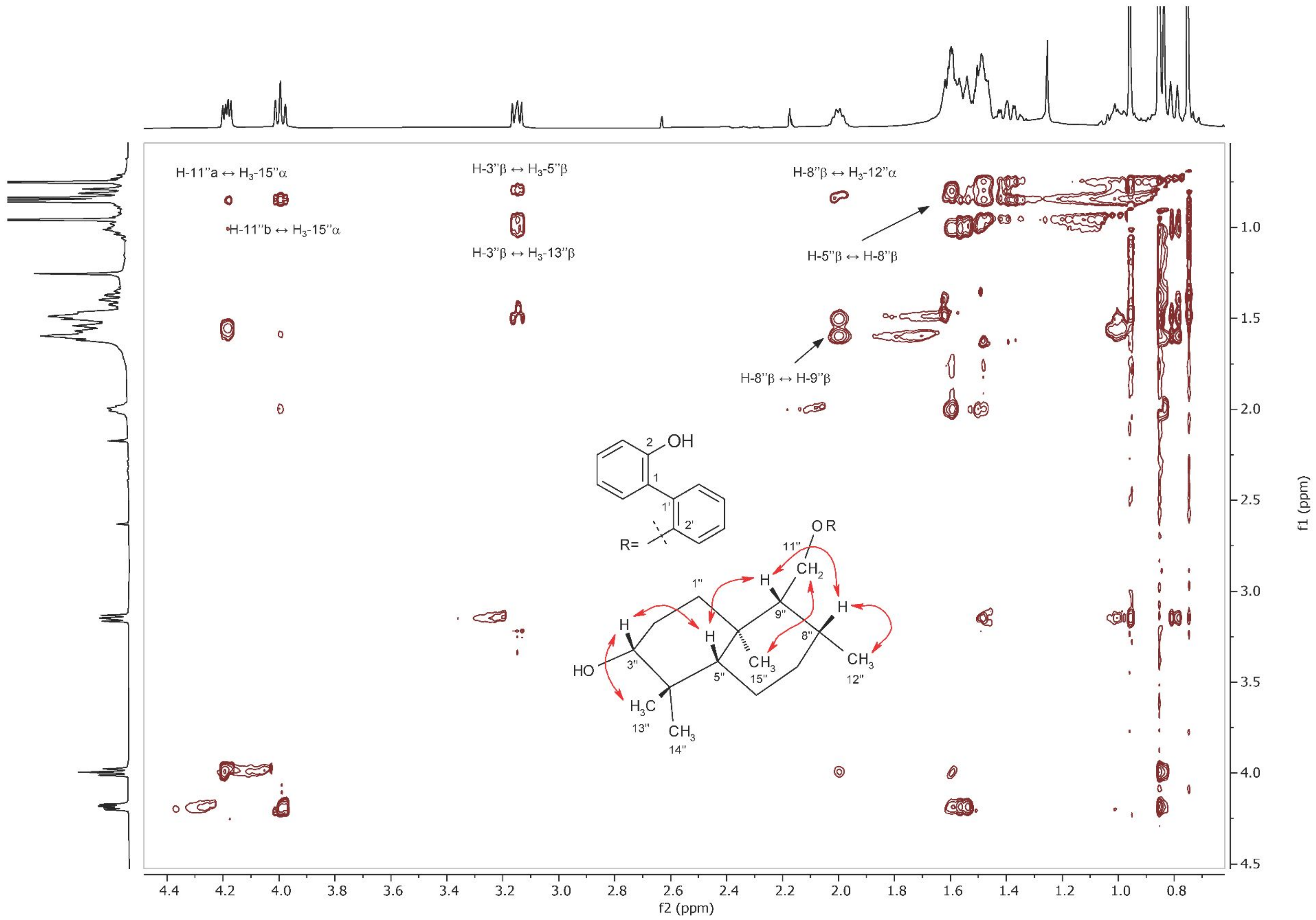

Figure S21g. Expansion of NOESY2D spectrum of 2'-(3"*R*(*S*),5"*S*(*R*), 8"*R*(*S*),9"*R*(*S*),10"*R*(*S*)-3"-hydroxydriman-11"-yloxy)-[1,1'-biphenyl]-2-ol ((±)-**38**) in $CDCl_3$. Selected NOESY2D correlations are annotated and highlighted on (±)-**38** structure.

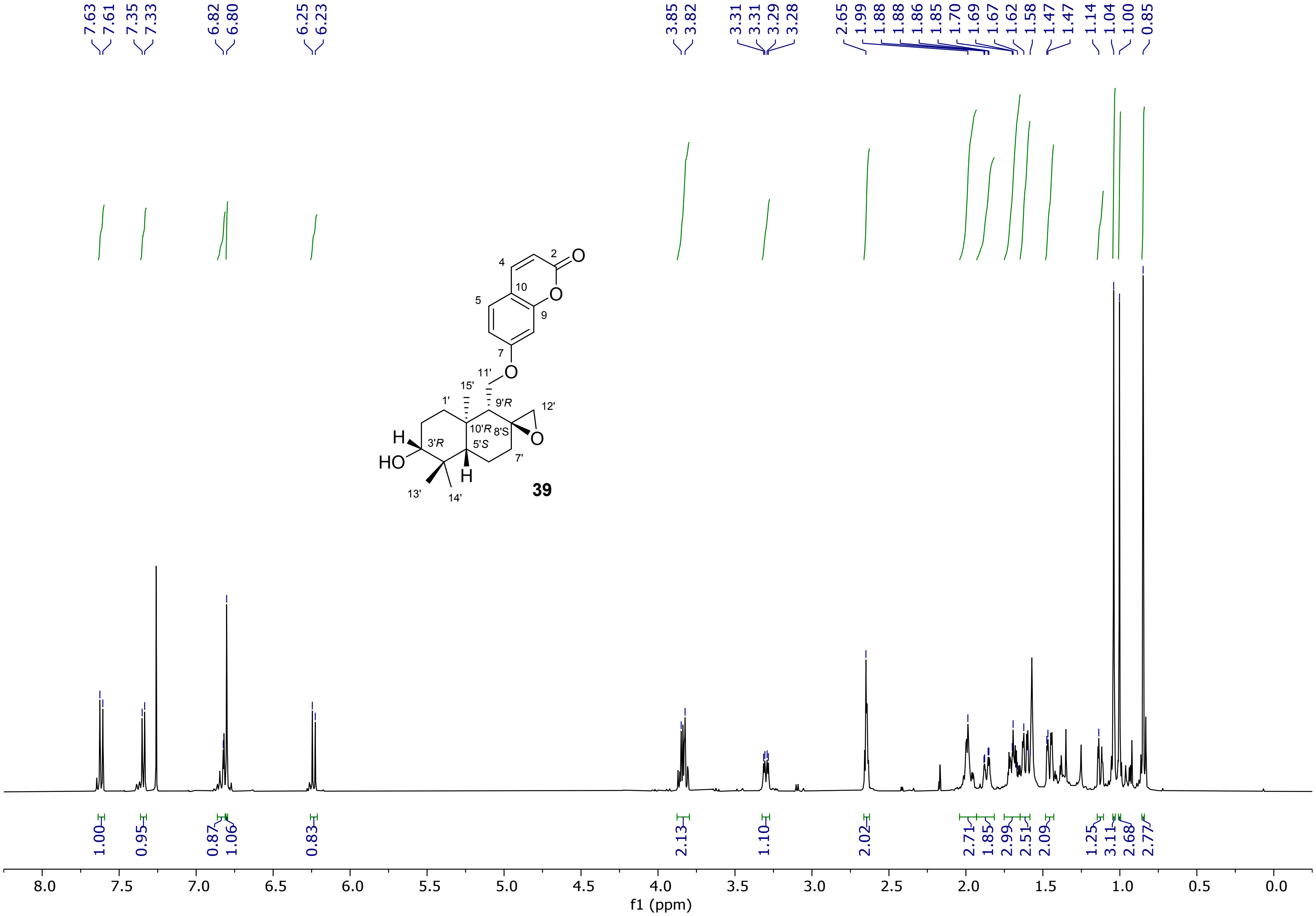

Figure S22a. ^{1}H NMR spectrum (500 MHz) of 7-(3'*R*(*S*),5'*S*(*R*), 8'*S*(*R*),9'*R*(*S*),10'*R*(*S*)-8',12'-epoxy-3'-hydroxydriman-11'-yloxy)-coumarin ((±)-**39**) in $CDCl_3$.

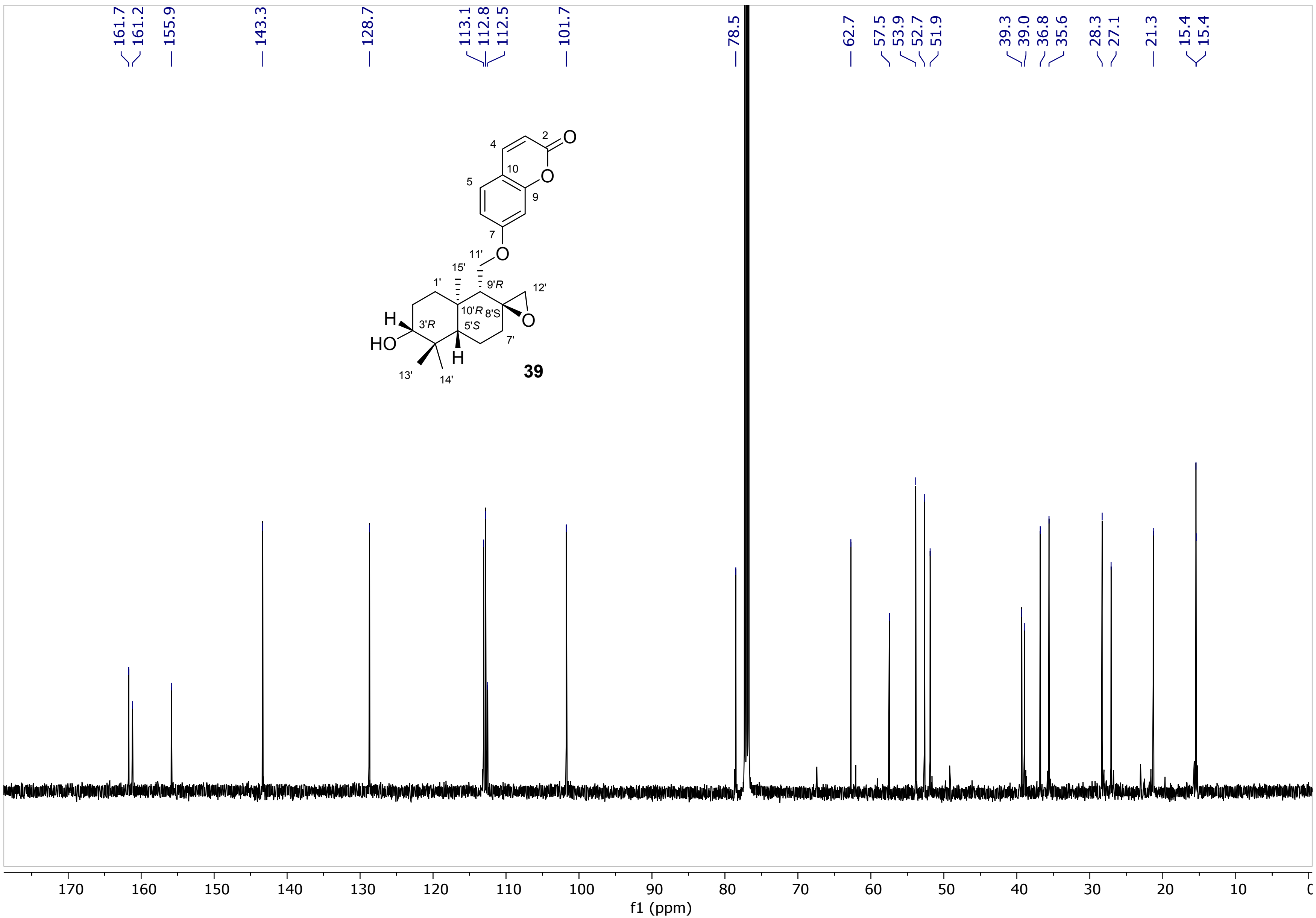

Figure S22b. ^{13}C NMR spectrum (125 MHz) of 7-(3'*R*(*S*),5'*S*(*R*),8'*S*(*R*),9'*R*(*S*),10'*R*(*S*)-8',12'-epoxy-3'-hydroxydriman-11'-yloxy)-coumarin ((±)-**39**) in CDCl$_3$.

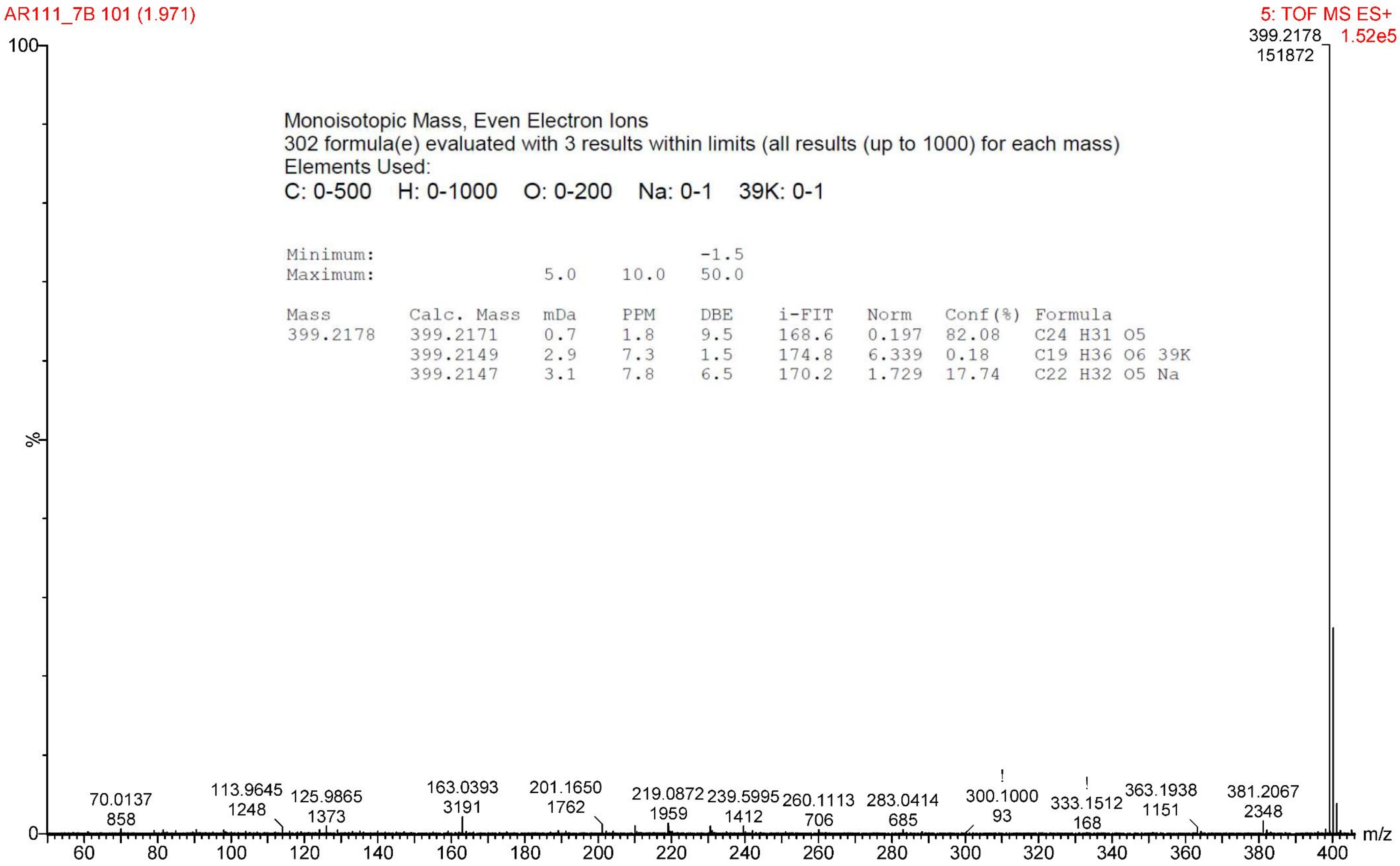

Figure S22c. HRESIMS of 7-(3'*R*(*S*),5'*S*(*R*), 8'*S*(*R*),9'*R*(*S*),10'*R*(*S*)-8',12'-epoxy-3'-hydroxydriman-11'-yloxy)-coumarin ((±)-**39**).

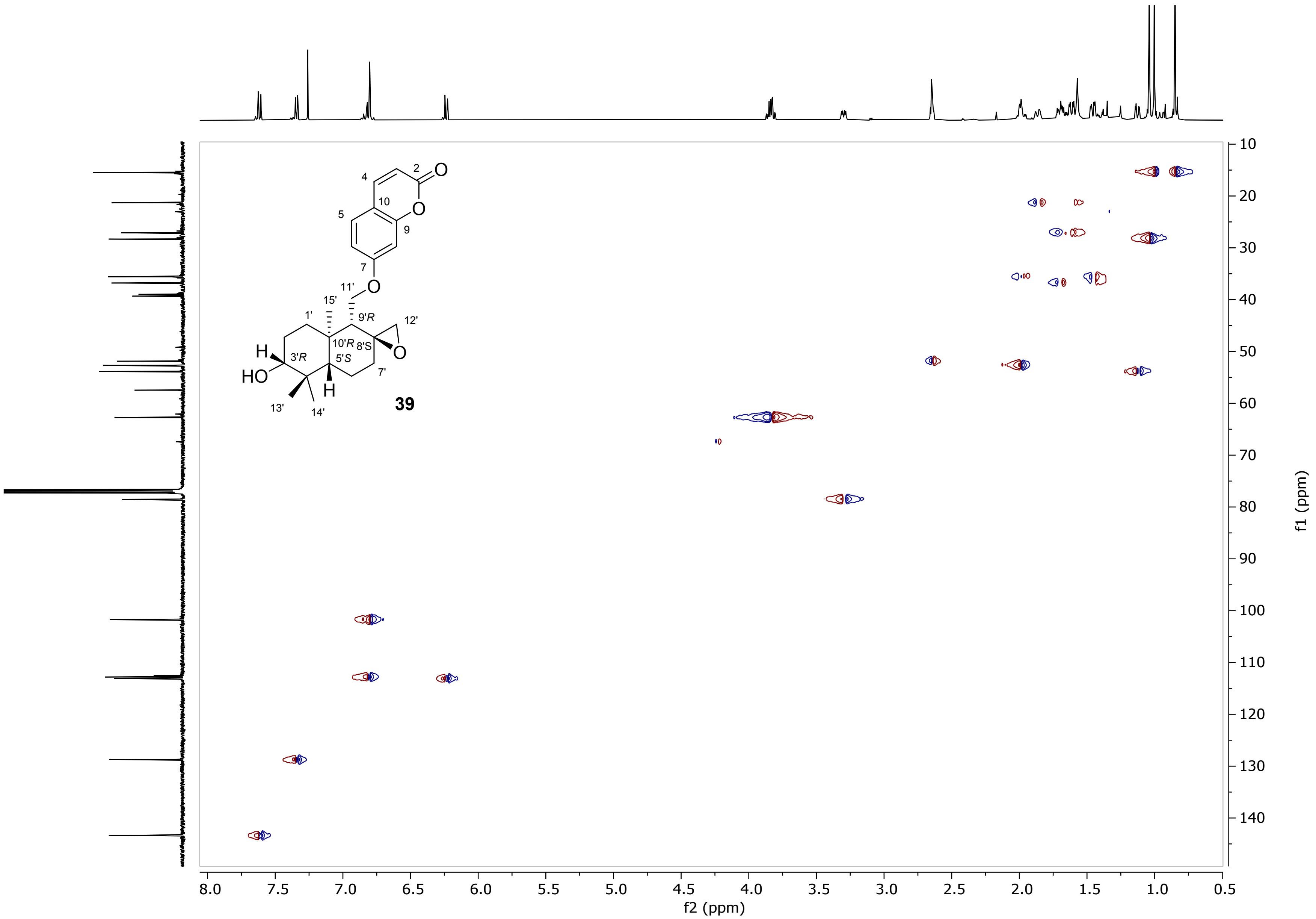

Figure S22d. gHSQC spectrum of 7-(3'*R*(*S*),5'*S*(*R*),8'*S*(*R*),9'*R*(*S*),10'*R*(*S*)-8',12'-epoxy-3'-hydroxydriman-11'-yloxy)-coumarin ((±)-**39**) in $CDCl_3$.

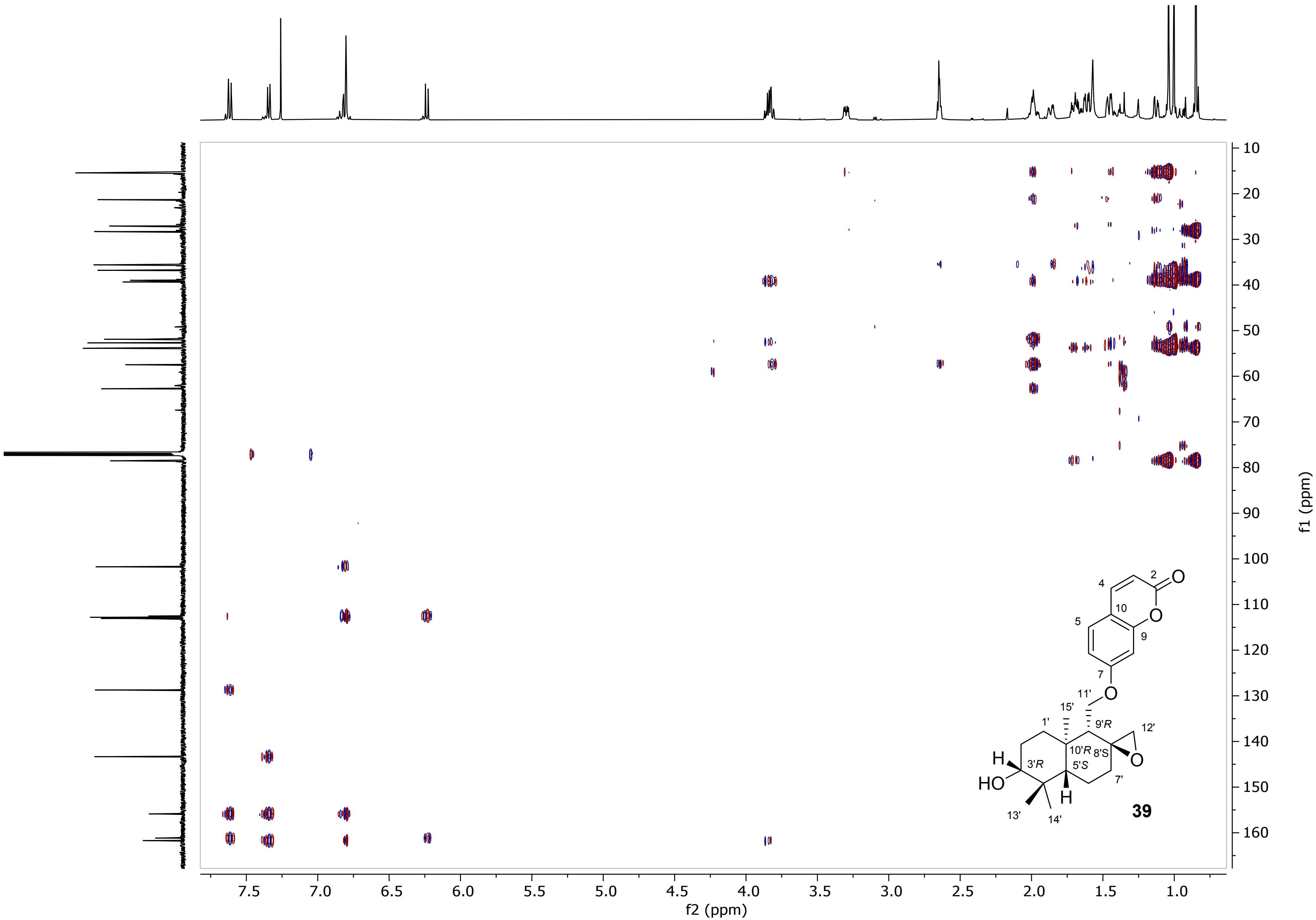

Figure S22e. gHMBC spectrum of 7-(3'*R*(*S*),5'*S*(*R*),8'*S*(*R*),9'*R*(*S*),10'*R*(*S*)-8',12'-epoxy-3'-hydroxydriman-11'-yloxy)-coumarin ((±)-**39**) in $CDCl_3$.

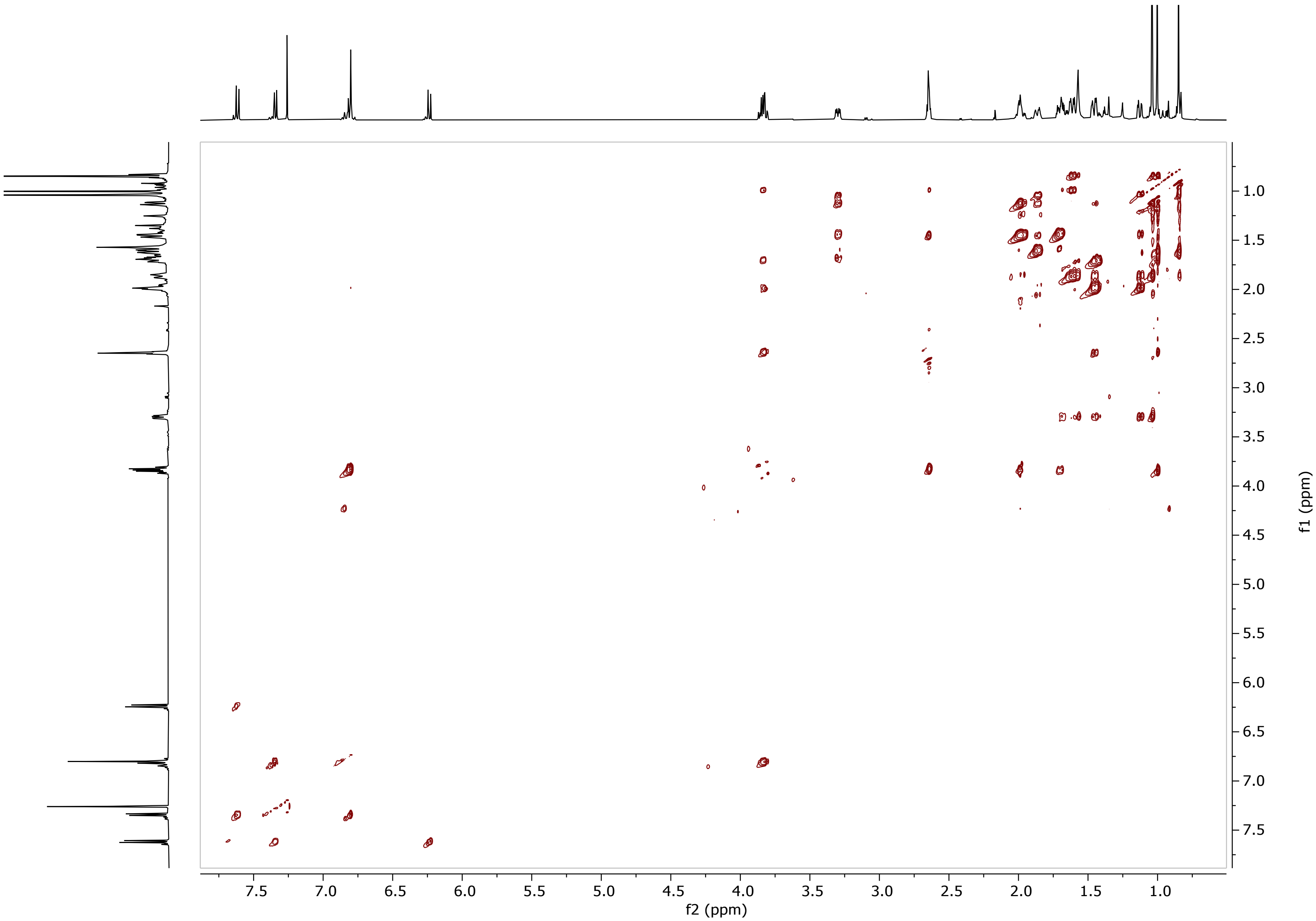

Figure S22f. NOESY2D spectrum of 7-(3'*R*(*S*),5'*S*(*R*),8'*S*(*R*),9'*R*(*S*),10'*R*(*S*)-8',12'-epoxy-3'-hydroxydriman-11'-yloxy)-coumarin ((±)-**39**) in $CDCl_3$.

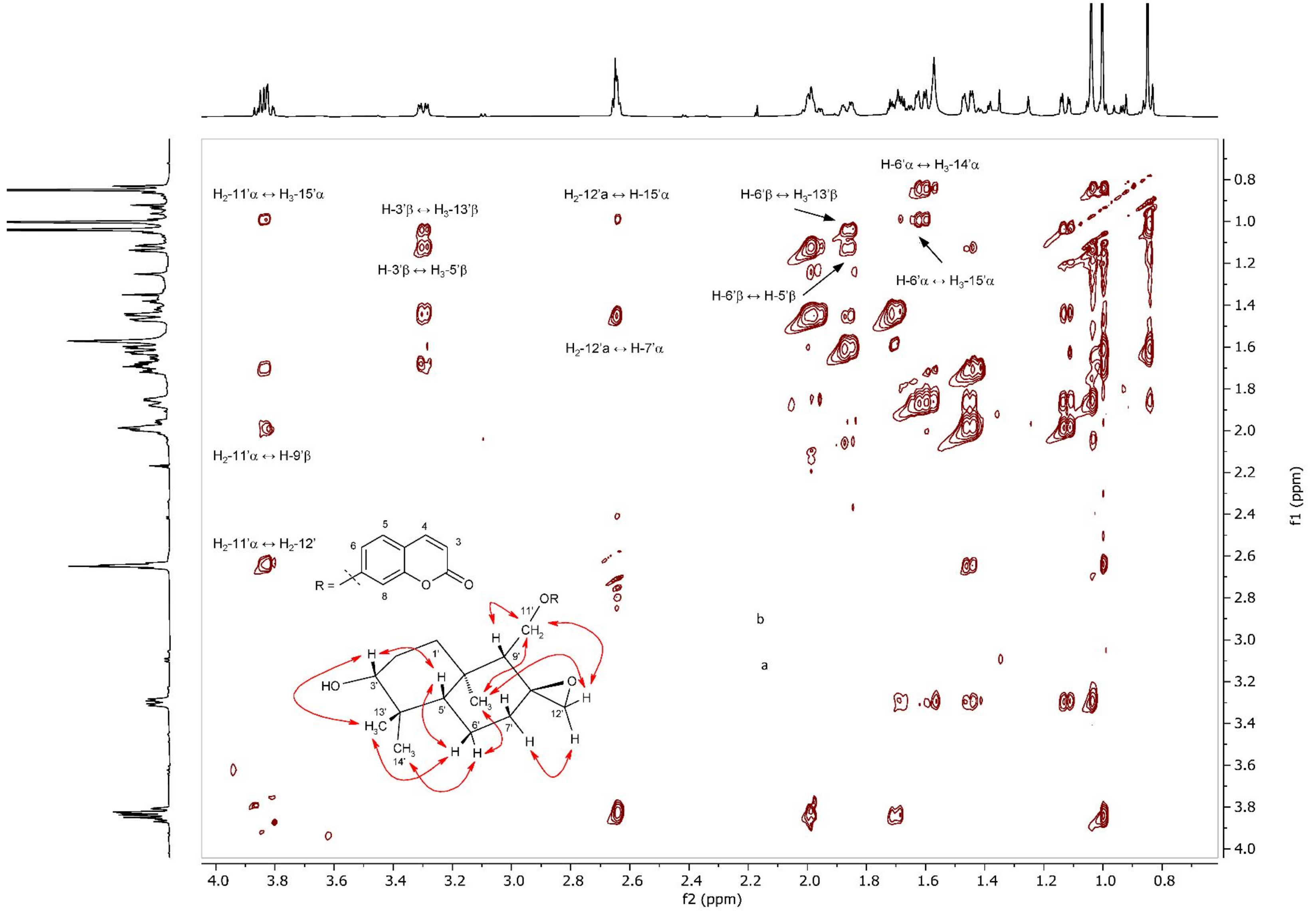

Figure S22g. Expansion of NOESY2D spectrum of 7-(3'*R*(*S*),5'*S*(*R*),8'*S*(*R*),9'*R*(*S*),10'*R*(*S*)-8',12'-Epoxy-3'-hydroxydriman-11'-yloxy)-coumarin ((±)-**39**) in $CDCl_3$. Selected NOESY2D correlations are annotated and highlighted on (±)-**39** structure.

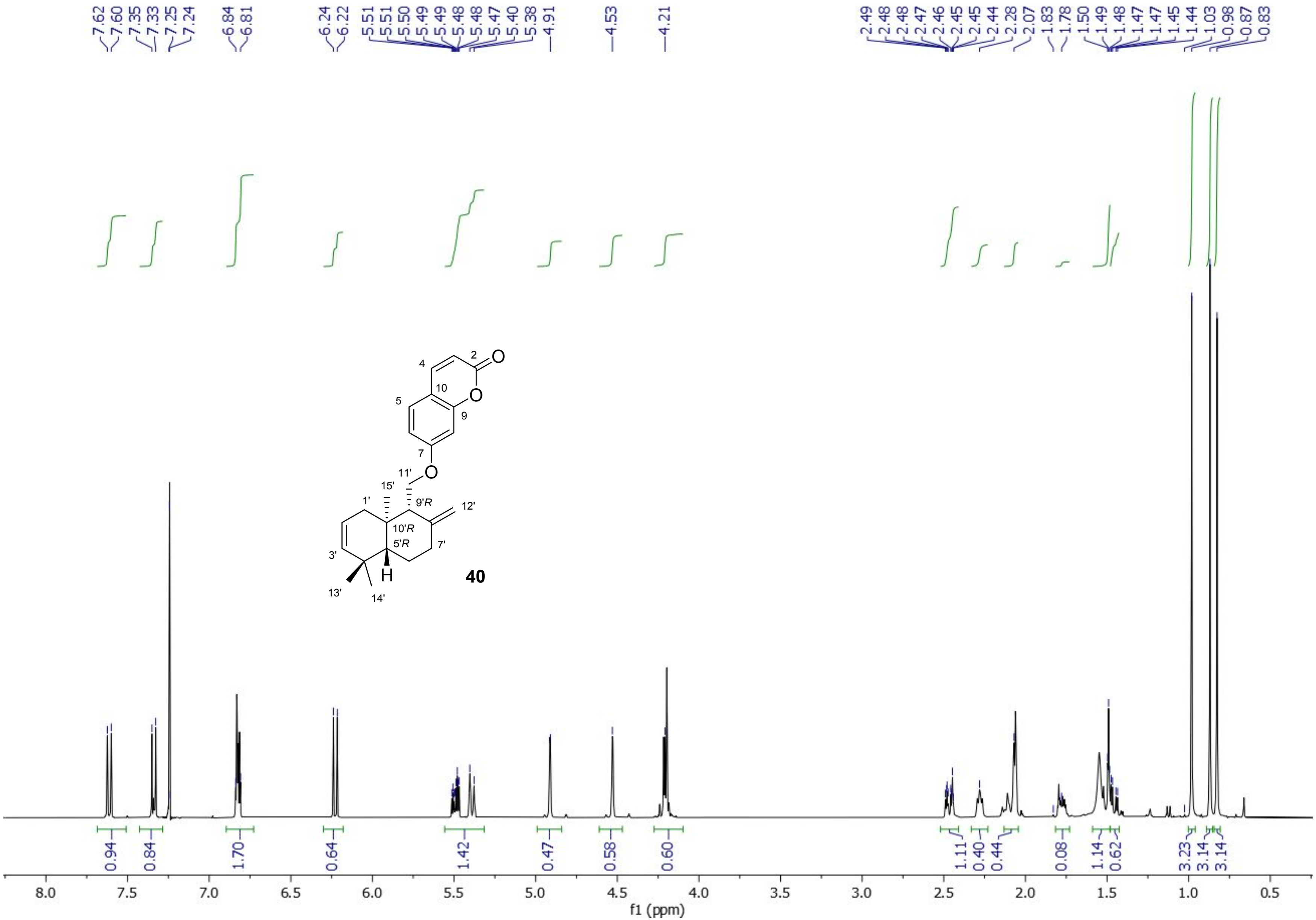

Figure S23a. ^{1}H NMR spectrum (400 MHz) of 7-(5'*R*(*S*),9'*R*(*S*),10'*R*(*S*)-drima-3',8'(12')-dien-11'-iloxy)-coumarin ((±)-**40**) in $CDCl_3$.

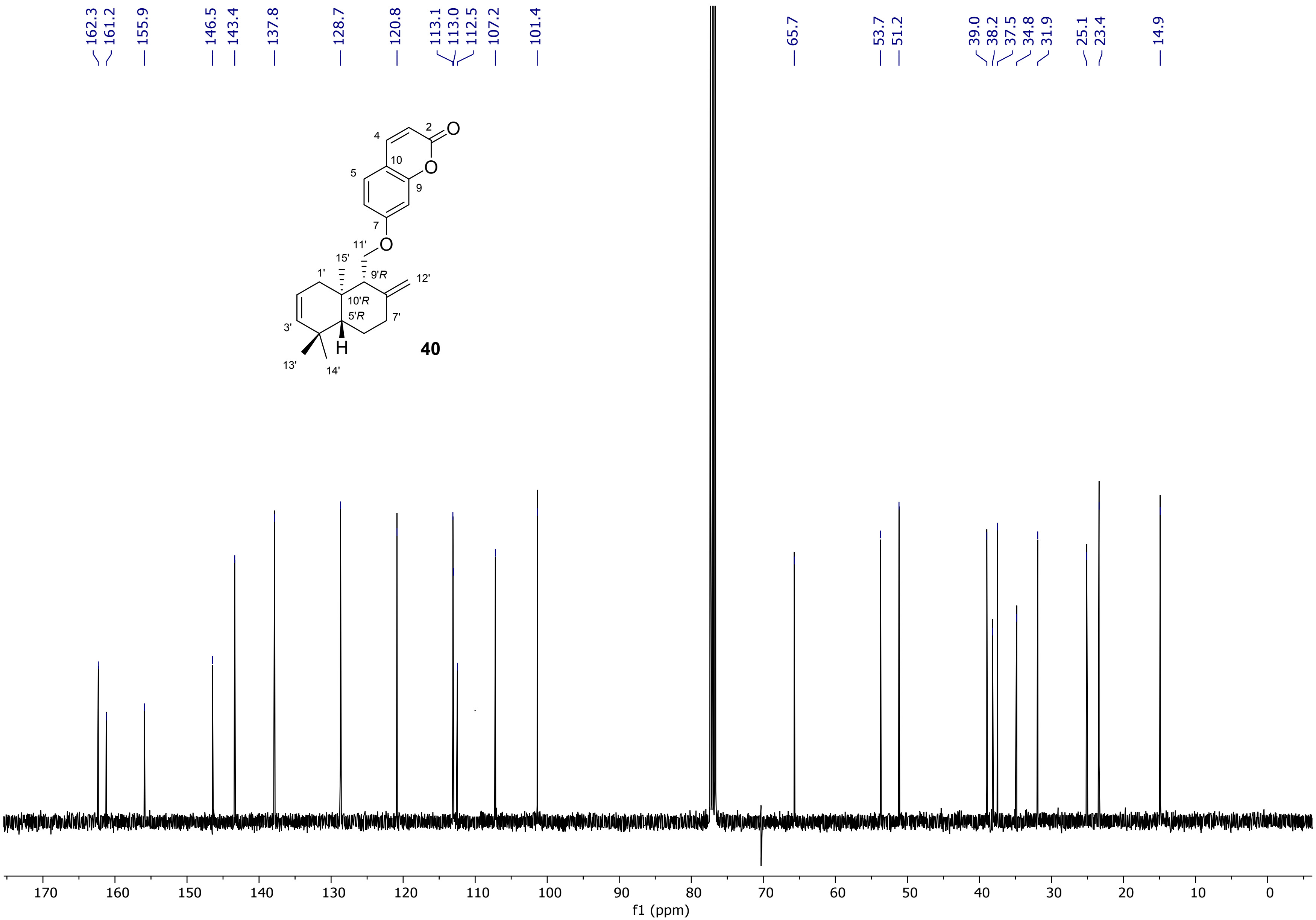

Figure S23b. ^{13}C NMR spectrum (100 MHz) of 7-(5'*R*(*S*),9'*R*(*S*),10'*R*(*S*)-drima-3',8'(12')-dien-11'-iloxy)-coumarin ((±)-**40**) in $CDCl_3$.

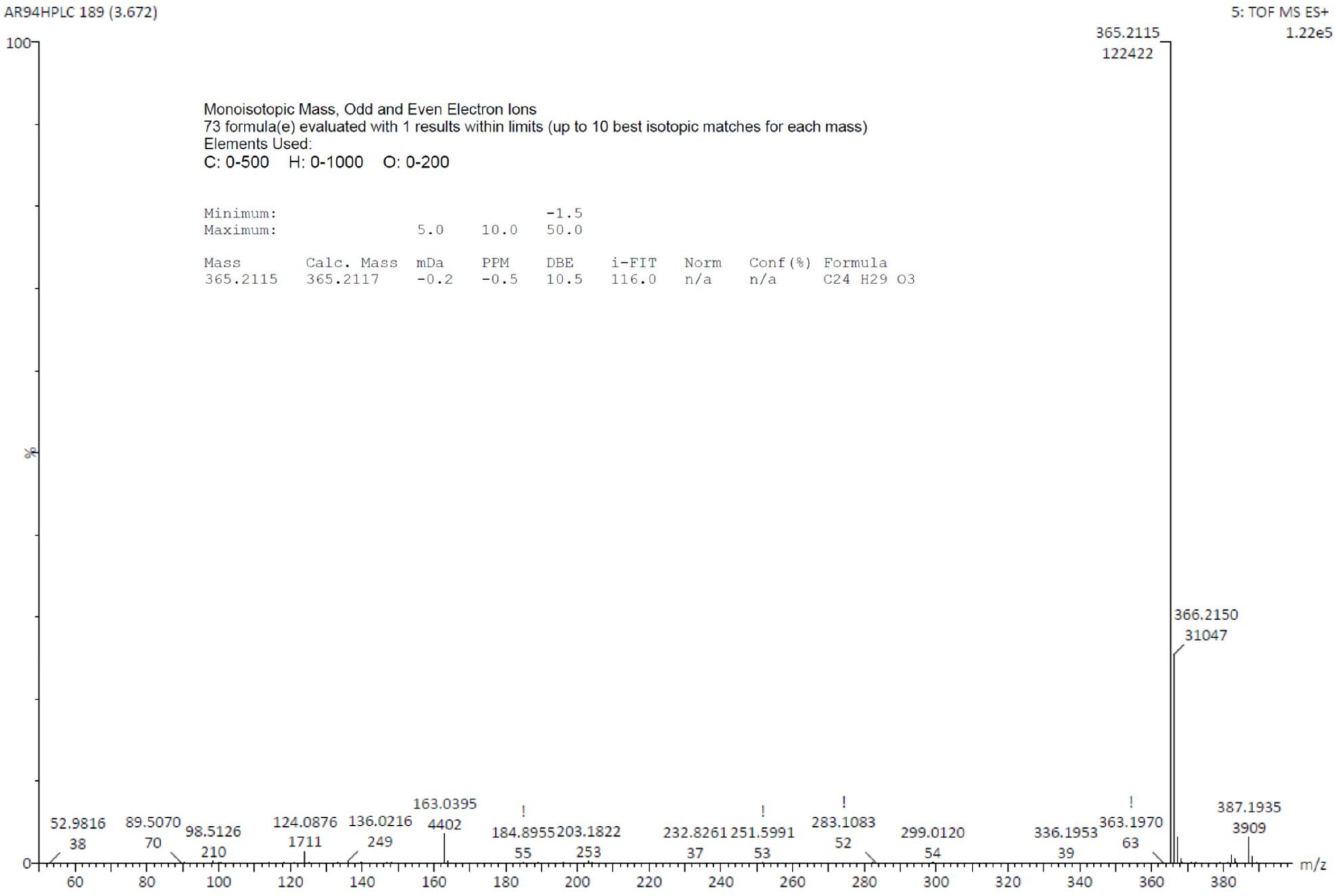

Figure S23c. HRESIMS of 7-(5'*R*(*S*),9'*R*(*S*),10'*R*(*S*)-drima-3',8'(12')-dien-11'-iloxy)-coumarin ((±)-**40**).

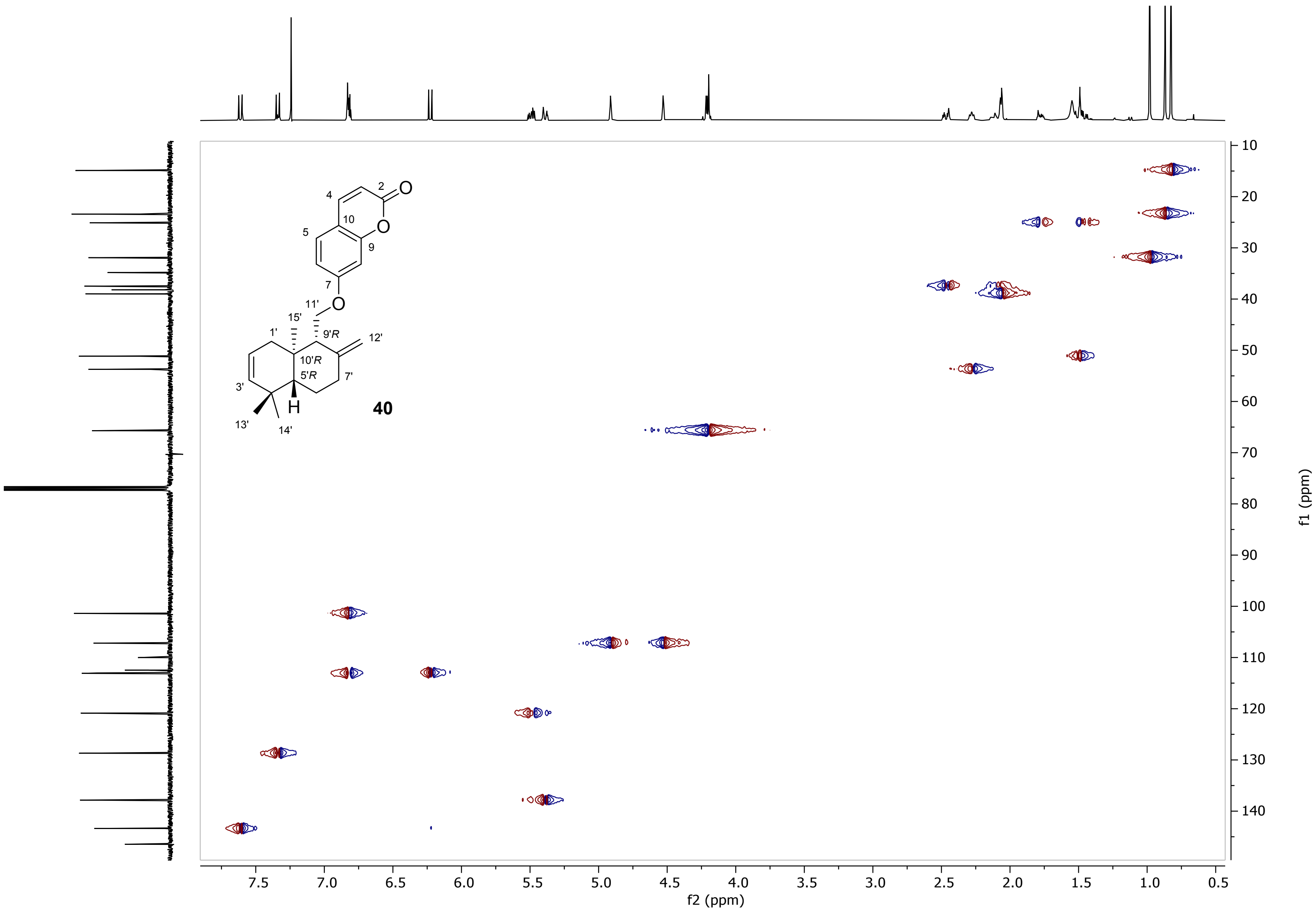

Figure S23d. gHSQC spectrum of 7-(5'*R*(*S*),9'*R*(*S*),10'*R*(*S*)-drima-3',8'(12')-dien-11'-iloxy)-coumarin ((±)-**40**) in $CDCl_3$.

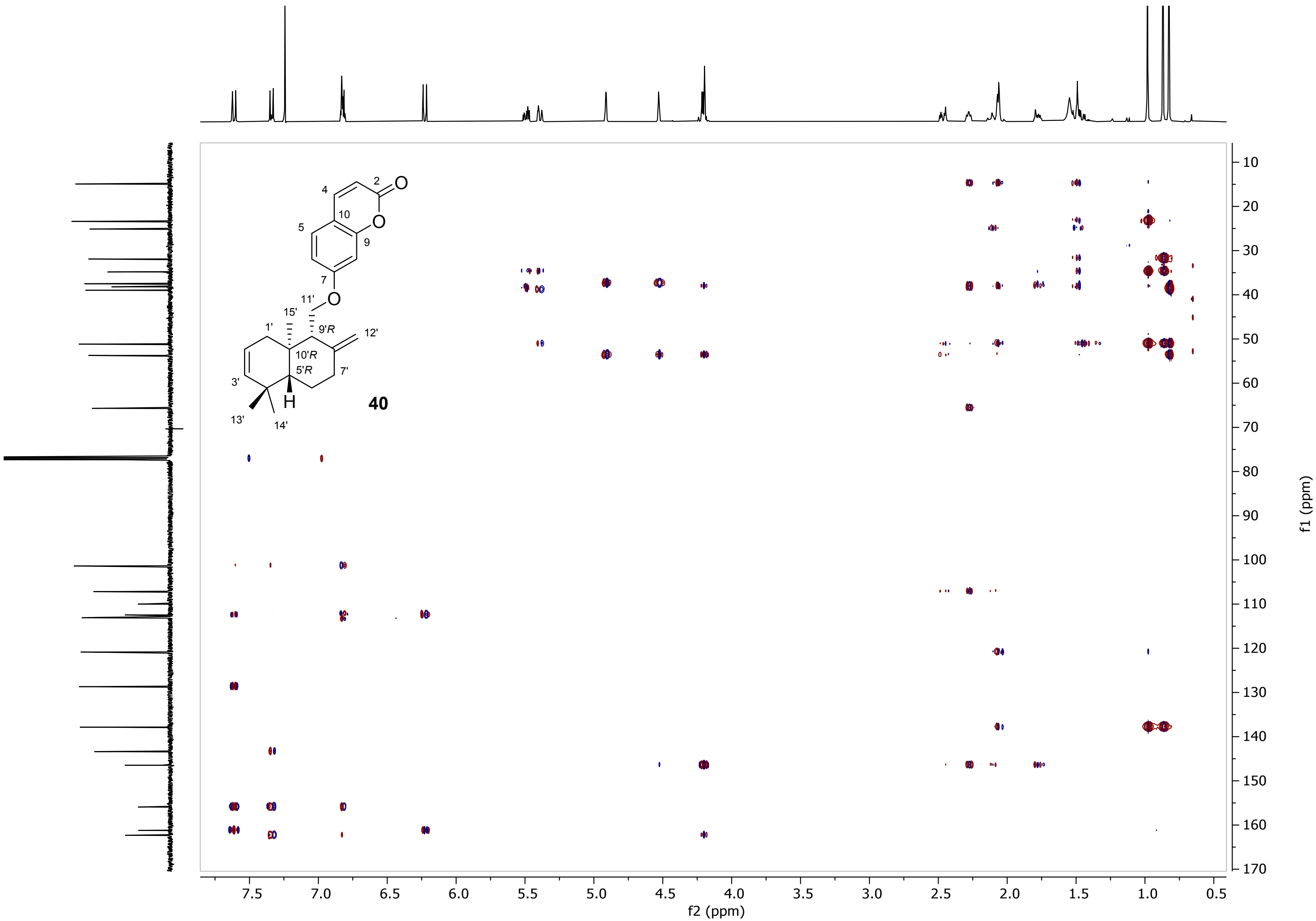

Figure S23e. gHMBC spectrum of 7-(5'*R*(*S*),9'*R*(*S*),10'*R*(*S*)-drima-3',8'(12')-dien-11'-iloxy)-coumarin ((±)-**40**) in $CDCl_3$.

Table S1-^{1}H. Comparison of ^{1}H-NMR spectroscopic data for (±)-**12** versus (-)-**12**[1] and (±)-**14** versus (+)-**14**[2].

H	(±)-**12**<br>δ_H[b] mult.<br>(*J* (Hz))	(-)-**12**[1]<br>δ_H[a] mult.<br>(*J* (Hz))	(±)-**14**<br>δ_H[b] mult.<br>(*J* (Hz))	(+)-**14**[2]<br>δ_H[c] mult.<br>(*J* (Hz))
1	-		-	-
2	-		-	-
3	6.25, d (9.6)	6.24, d, (9.6)	6.25, d (9.4)	6.27, d (9.5)
4	7.63, d (9.6)	7.62, d (9.6)	7.63, d (9.4)	7.66, d (9.5)
5	7.36, d (9.4)	7.35, d (8.8)	7.36, d (8.4)	7.39, d (8.4)
6	6.82, m	6.83, dd (8.8, 2.8)	6.84, dd (8.4, 2.5)	6.88, dd (8.4, 2.3)
7	-		-	
8	6.81, m	6.81, d (2.8)	6.81, d (2.5)	6.85, d (2.3)
9	-		-	-
10	-		-	-
1′	α: 1.82, m<br>β: 1.46, m	eq: 1.68, m<br>axi: 1.48, m	a: 2.01, m<br>b: 1.33, td (13.3, 4.7)	α: 2.05, m<br>β: 1.37, dd (13.2, 4.2)
2′	a: 1.73, m<br>b: 1.63, m	eq: 1.68, m<br>axi: 1.58, m	a, b: 1.63	a, b: 1.67
3′	β: 3.31, dd (11.8, 4.4)	axi: 3.30, dd (11.0, 4.2)	β: 3.29 dd (11.2, 4.6)	β: 3.31 d (br) (10.8)
4′	-		-	
5′	β: 1.18, dd (12.6, 2.7)	axi: 1.22, dd, (10.5, 6.2)	β: 1.28, dd (11.4, 5.3)	β: 1.30, dd (11.4, 6.0)
6′	a: 1.76, m<br>b: 1.46, m	eq: 1.68, m<br>axi: 1.38, m	a: 2.04, m<br>b: 1.67, m	a, b: 2.01
7′	α: 2.47 ddd (13.4, 4.3, 2.4)<br>β: 2.10 td (13.6, 5.2)	eq: 2.49, m<br>axi: 2.12, m	5.55, s (br)	5.60, s (br)
8′	-		-	
9′	β: 2.21, m	axi: 2.31, t (4.2)	β: 2.22, s(br)	β: 2.26, s(br)
10′	-		-	
11′	a, b: 4.20	a: 4.35, dd (9.6, 4.2)<br>b: 4.27, dd (9.6, 4.1)	a: 4.16, dd (9.7, 3.4)<br>b: 4.01, dd (9.7, 5.9)	a: 4.19, dd (9.6, 3.0)<br>b: 4.03, dd (9.6, 5.4)
12′	a: 4.92, s<br>b: 4.54, s	a: 4.89, br s<br>b: 4.81, br s	1.69, s	1.73 s
13′	β: 1.03, s[3]	1.04, s	β: 1.01, s	β: 1.05, s
14′	α: 0.82, s[3]	0.88, s	α: 0.89, s	α: 0.93, s
15′	α: 0.85, s[3]	0.83, s	α: 0.91, s	α: 0.95, s

[a] $CDCl_3$ (400 MHz); [b] $CDCl_3$ (500 MHz); [c] $CDCl_3$ (600 MHz).

.

[1] Abd El-Razek, M.H.; Wu, Y.-C.; Chang, F.-R. Sesquiterpene Coumarins from Ferula Foetida. *J. Chinese Chem. Soc.* **2007**, *54*, 235–238, doi: 10.1002/jccs.200700035.

[2] Iranshahi, M.; Rezaee, R.; Sahebkar, A.; Bassarello, C.; Piacente, S.; Pizza, C. Sesquiterpene coumarins from the fruits of Ferula badrakema. *Pharm. Biol.* **2009**, *47*, 344–347, doi:10.1080/13880200902752884.

[3] Structural assignment based on NOESY2D experiment (see Figure S11g).

Table S1-^{13}C. Comparison of ^{13}C-NMR spectroscopic data for (±)-**12** versus (-)-**12**[1] and (±)-**14** versus (+)-**14**[2].

	(±)-**12**	(-)-**12**[1]	(±)-**14**	(+)-**14**[2]
**C**	δ_C[b], mult.	δ_C[a], mult.	δ_C[b], mult.	δ_C[c], mult.
1	-		-	-
2	161.2, C	161.0	161.2, C	161.6
3	113.0, CH	112.6	113.1, CH	113.4
4	143.4, CH	143.3	143.4, CH	143.8
5	128.7, CH	128.6	128.7, CH	129.1
6	113.1, CH	113.2	113.0, CH	113.5
7	162.2, C	162.0	162.00, C	162.4
8	101.3, CH	101.2	101.3, CH	101.7
9	155.9, C	155.6	155.9, C	156.3
10	112.5, C	118.3	112.5, C	112.9
1′	37.2, CH_2	36.7	37.8, CH_2	38.2
2′	27.7, CH_2	27.4	27.3, CH_2	27.7
3′	78.5, CH	78.0	78.9, CH	79.2
4′	39.2, C	38.8	38.7, C	39.1
5′	54.8, CH	54.7	49.4, CH	49.7
6′	23.5, CH_2	23.3	23.3, CH_2	23.7
7′	37.4, CH_2	37.1	123.7, CH	124.1
8′	146.2, C	146.1	132.3, C	132.7
9′	54.3, CH	54.0	53.8, CH	54.2
10′	38.8, C	38.5	35.9, C	36.2
11′	65.7, CH_2	65.3	67.0, CH_2	67.4
12′	107.8, CH_2	107.5	21.6, CH_3	22.0
13′	28.3, CH_3 [3]	15.4	28.0, CH_3	28.4
14′	15.3, CH_3 [3]	28.2	15.3, CH_3	15.3
15′	15.35, CH_3 [3]	15.1	14.9, CH_3	15.2

[a] $CDCl_3$ (100 MHz); [d] $CDCl_3$ (125 MHz); [c] $CDCl_3$ (600 MHz).

Table S2. Molar doses for broth microdilution assays against *B. cinerea* for compounds (±)**-12**-(±)**-14**, **21**-**25**, (±)-**26**-(±)**-33**, (±)**-35**-(±)**-40**, triclosan and azoxystrobin (10^{-5} mg/mL versus 10^{-6} M).

	Doses		
**Compound**	6250 [a]	391 [a,b]	49 [a,c]
(±)**-12**	163.4 [d]	10.2 [d]	1.3 [d]
(±)**-13**	147.2 [d]	9.2 [d]	1.2 [d]
(±)**-14**	163.4 [d]	10.2 [d]	1.3 [d]
**21**	170.5 [d]	10.7 [d]	1.3 [d]
**22**	164.2 [d]	10.3 [d]	1.3 [d]
**23**	183.5 [d]	11.5 [d]	1.4
**24**	175.3 [d]	11.0 [d]	1.4 [d]
**25**	160.0	10.0	1.3
(±)**-26**	163.4 [d]	10.2 [d]	1.3 [d]
(±)**-27**	157.6 [d]	9.9 [d]	1.2 [d]
(±)**-28**	175.3 [d]	11.0 [d]	1.4 [d]
(±)**-29**	167.8 [d]	10.5 [d]	1.3 [d]
(±)**-30**	153.7 [d]	9.6 [d]	1.2 [d]
(±)**-31**	157.6 [d]	9.9 [d]	1.2 [d]
(±)**-32**	157.6 [d]	9.9 [d]	1.2 [d]
(±)**-33**	175.3 [d]	11.0 [d]	1.4 [d]
(±)**-35**	167.8 [d]	10.5 [d]	1.3 [d]
(±)**-36**	167.8 [d]	10.5 [d]	1.3 [d]
(±)**-37**	153.7 [d]	9.6 [d]	1.2 [d]
(±)**-38**	153.7 [d]	9.6 [d]	1.2 [d]
(±)**-39**	156.8 [d]	9.8 [d]	1.2 [d]
(±)**-40**	171.5 [d]	10.7 [d]	1.3 [d]
Triclosan (C1+)	215.9 [d]	13.5 [d]	1.7 [d]
Azoxystrobin (C2+)	154.9 [d]	9.7 [d]	1.2 [d]

[a] 10^{-5} mg/mL; [b] 16-fold dilution; [b] 128-fold dilution; [d] 10^{-6} M; concentration range for 6250 10^{-5} mg/mL dose = 215.9 10^{-6} M– 147.2 10^{-6} M; concentration range for 391 10^{-5} mg/mL dose = 13.5 10^{-6} M– 9.2 10^{-6} M; concentration range for 49 10^{-5} mg/mL dose = 1.7 10^{-6} M– 1.2 10^{-6} M.

Supplementary Materials

Table S3. Inhibition of fungal growth percentage (IFG%) against *B. cinerea* for compounds (±)-**12**-(±)-**14**, **21**-**25**, (±)-**26**-(±)-**33**, (±)-**35**-(±)-**40**, triclosan (**C1+**) and azoxystrobin (**C2+**). (6250 10^{-5} mg/mL, 391 10^{-5} mg/mL and 49 10^{-5} mg/mL doses; see molar doses for every compound in Table S2).

Inhibition of fungal growth percentage (IFG%)	**Doses**		
**Compound**	6250 [a]	391 [a,b]	49 [a,c]
(±)-**12**	92.2 ± 3.7	83.2 ± 3.2	23.3 ± 0.7
(±)-**13**	47.5 ± 1.3	22.6 ± 1.3	12.8 ±
(±)-**14**	81.0 ± 1.3	38.6 ± 1.3	9.0 ± 0.1
**21**	78.1 ± 5.5	11.5 ± 2.4	0.0
**22**	62.1 ± 5.2	3.5 ± 1.8	0.0
**23**	91.6 ± 4.8	6.0 ± 3.9	0.0
**24**	57.8 ± 0.5	5.9 ± 1.1	0.0
**25**	47.5 ± 4.6	0.0	0.0
(±)-**26**	72.0 ± 3.2	11.8 ± 8.4	0.0
(±)-**27**	67.5 ± 3.3	14.0 ± 0.9	0.0
(±)-**28**	42.3 ± 3.6	5.6 ± 2.9	0.0
(±)-**29**	38.4 ± 3.0	2.9 ± 2.8	0.0
(±)-**30**	73.8 ± 5.2	17.8 ± 4.4	0.0
(±)-**31**	84.0 ± 3.7	75.1 ± 3.1	19.9 ± 2.9
(±)-**32**	57.3 ± 1.2	36.4 ± 1.5	0.0
(±)-**33**	43.1 ± 7.3	6.3 ± 3.7	0.0
(±)-**35**	98.8 ± 0.7	77.2 ± 4.1	39.5 ± 0.1
(±)-**36**	76.1 ± 1.8	54.2 ± 3.4	7.9 ± 0.9
(±)-**37**	61.6 ± 2.7	17.2 ± 1.8	8.5 ± 4.1
(±)-**38**	29.5 ± 2.2	13.6 ± 6.6	5.6 ± 5.6
(±)-**39**	75.3 ± 2.7	23.3 ± 1.5	7.2 ± 5.0
(±)-**40**	20.9 ± 2.6	0.0	0.0
Triclosan (C1+)	100.0 ± 0.0	83.2 ± 4.7	13.5 ± 7.0
Azoxystrobin (C2+)	73.8 ± 6.2	59.7 ± 5.6	40.4 ± 2.0

Inhibition of fungal growth percentage (IFG%): means ± standard deviation (SD). [a] 10^{-5} mg/mL; [b] 16-fold dilution; [b] 128-fold dilution; [d] 10^{-6} M.

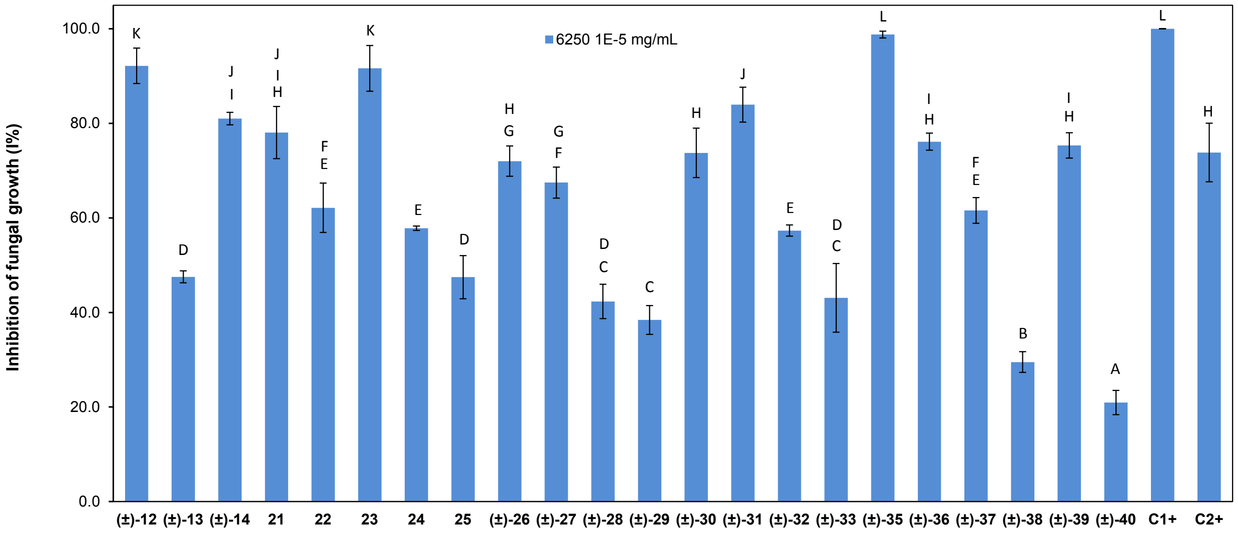

Figure S24. Comparison of inhibition of fungal growth percentage (IFG%) among compounds (±)**-12**-(±)**-14**, **21**-**25**, (±)**-26**-(±)**-33**, (±)**-35**-(±)**-40**, triclosan (**C1+**) and azoxystrobin (**C2+**) (*B. cinerea*, 6250 10^{-5} mg/mL dose; see molar doses for every compound in Table S2). Data are presented as mean ± standard deviation. Differences in IFG% between compounds are presented in uppercase Latin: different letters represent statistically significant differences between compounds; p<0.05.

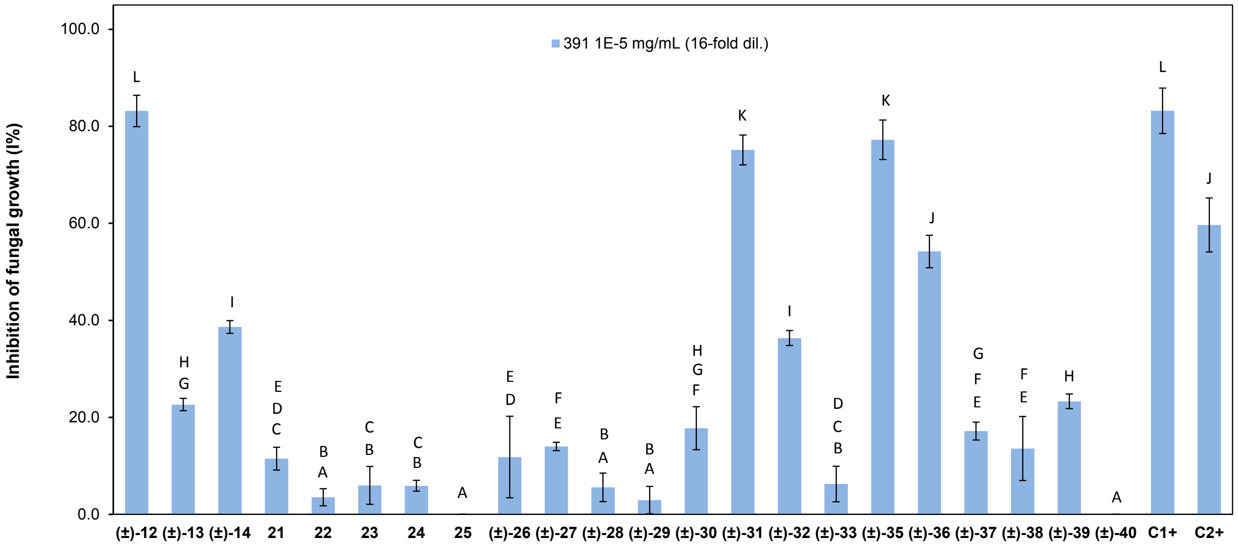

Figure S25. Comparison of inhibition of fungal growth percentage (IFG%) among compounds (±)**-12**-(±)**-14**, **21**-**25**, (±)**-26**-(±)**-33**, (±)**-35**-(±)**-40**, triclosan (**C1+**) and azoxystrobin (**C2+**) (*B. cinerea*, 391 10^{-5} mg/mL dose - 16-fold dilution from 6250 10^{-5} mg/mL dose-); see molar doses for every compound in Table S2). Data are presented as mean ± standard deviation. Differences in IFG% between compounds are presented in uppercase Latin: different letters represent statistically significant differences between compounds; p<0.05.

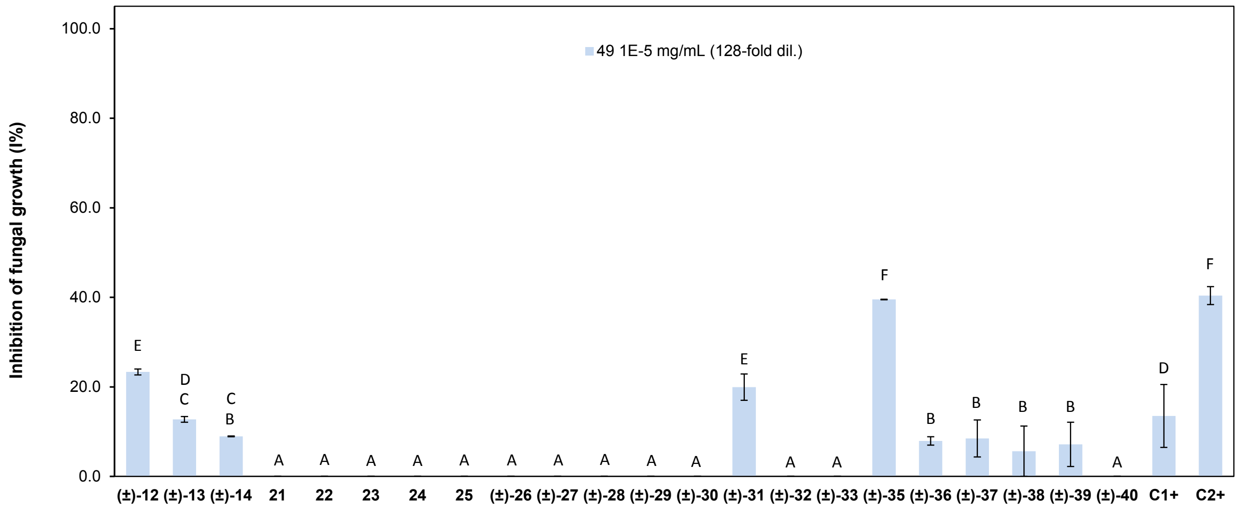

Figure S26. Comparison of inhibition of fungal growth percentage (IFG%) among compounds (±)**-12**-(±)**-14**, **21-25**, (±)**-26**-(±)**-33**, (±)**-35**-(±)**-40**, triclosan (**C1+**) and azoxystrobin (**C2+**) (*B. cinerea*, 49 10^{-5} mg/mL dose - 128-fold dilution from 6250 10^{-5} mg/mL dose-); see molar doses for every compound in Table S2). Data are presented as mean ± standard deviation. Differences in IFG% between compounds are presented in uppercase Latin: different letters represent statistically significant differences between compounds; $p<0.05$.

Table S4. Calculated (www.molinspiration.com) logP, total polar surface area (TPSA)[4], hydrogen bond donor atoms (HBA) and hydroxyl groups (OH) for compounds (±)**-12**-(±)**-14**, (±)**-31** **-** (±)**-33**, (±)**-35** **-** (±)**-40**.

**Compound**	LogP	TPSA	HBA	OH
(±)**-12**	5.30	59.67	4	1
(±)**-13**	6.01	65.75	5	0
(±)**-14**	5.52	59.67	4	1
(±)**-31**	5.68	59.67	4	1
(±)**-32**	5.89	59.67	4	1
(±)**-33**	5.10	46.53	3	1
(±)**-35**	5.07	66.76	4	2
(±)**-36**	5.28	66.76	4	2
(±)**-37**	6.73	49.69	3	2
(±)**-38**	6.94	49.69	3	2
(±)**-39**	4.62	72.20	5	1
(±)**-40**	6.20	39.45	3	0

[4] Ertl, P.; Rohde, B.; Selzer, P. Fast Calculation of Molecular Polar Surface Area as a Sum of Fragment-Based Contributions and Its Application to the Prediction of Drug Transport Properties. *J. Med. Chem.* **2000**, *43*, 3714–3717, doi:10.1021/jm000942e.

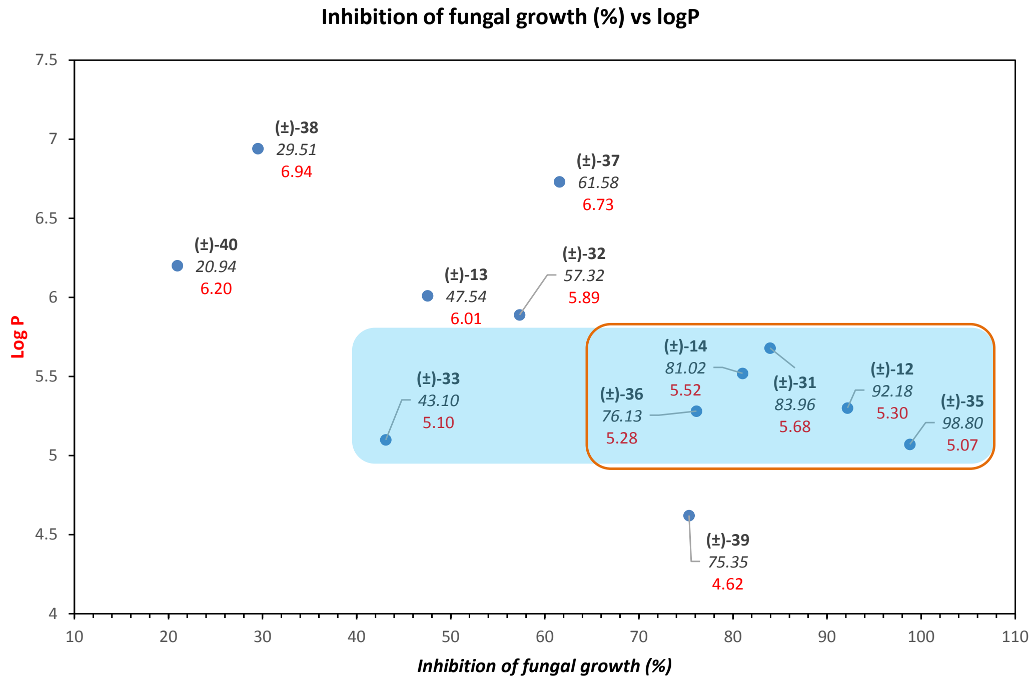

Figure S27. Comparison of inhibition of fungal growth percentage (IFG%) (*B. cinerea*, 6250 10^{-5} mg/mL dose; see molar doses for every compound in Table S2) versus Log P for compounds (±)-**12**-(±)-**14**, (±)-**31**-(±)-**33**, (±)-**35**-(±)-**40**. In shaded blue, compounds falling in the logP range 5.68-5.07; circled in orange, compounds also displaying TPSA values within range 66.76 -59.67 Å^2 (see Figure S28).

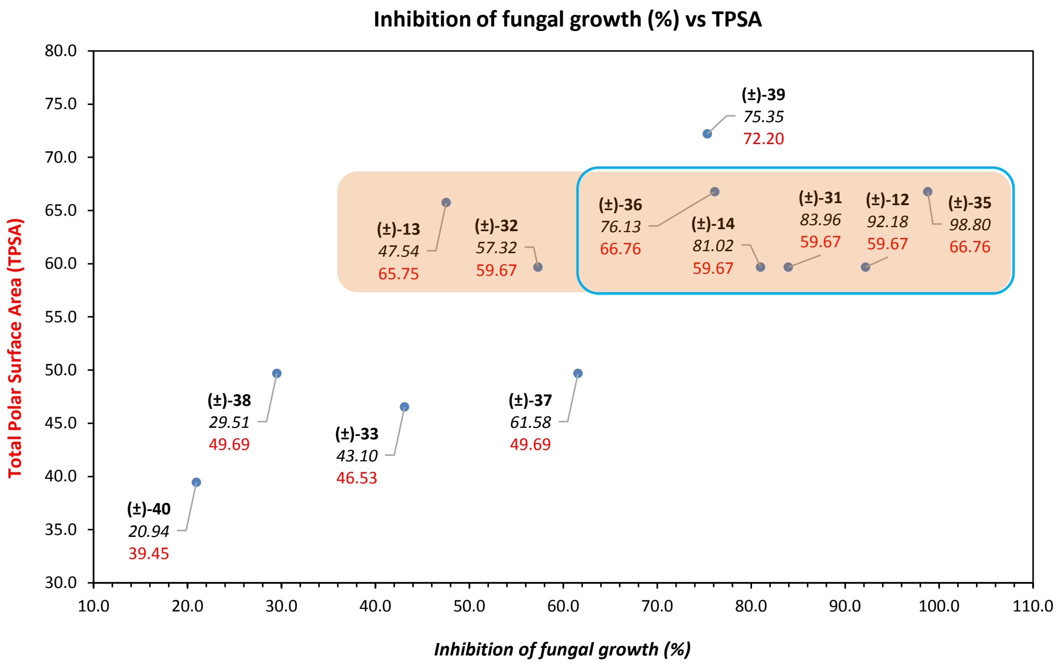

Figure S28. Comparison of inhibition of fungal growth percentage (IFG%) (*B. cinerea*, 6250 10^{-5} mg/mL dose; see molar doses for every compound in Table S2) versus total polar surface area (TPSA)[4] for compounds (±)-**12**-(±)-**14**, (±)-**31**-(±)-**33**, (±)-**35**-(±)-**40**. In shaded orange, compounds falling in the TPSA range 66.76 -59.67 Å^2; circled in blue, compounds also displaying logP values within range 5.68-5.07 (see Figure S27).
